# Total synthesis of the putative structure of the proposed Banyasin A

**DOI:** 10.3389/fchem.2015.00019

**Published:** 2015-03-17

**Authors:** Xuguang Gao, Qi Ren, Sun Choi, Zhengshuang Xu, Tao Ye

**Affiliations:** ^1^Laboratory of Chemical Genomics, School of Chemical Biology and Biotechnology, Peking University Shenzhen Graduate SchoolShenzhen, China; ^2^National Leading Research Laboratory of Molecular Modeling and Drug Design, College of Pharmacy, Graduate School of Pharmaceutical Sciences, Ewha Womans UniversitySeoul, South Korea; ^3^Department of Applied Biology and Chemical Technology, The Hong Kong Polytechnic UniversityHong Kong, China

**Keywords:** Banyasin A, total synthesis, stereoselective, cyclopeptide, chitinase inhibitors

## Abstract

The first total synthesis of four possible isomers of a molecule possessing the configuration proposed for Banyasin A is described. The structure synthesized appears to be different from that of the natural product.

## Introduction

Secondary metabolites produced by cyanobacteria are key synthetic targets in the quest for new leads in the pharmaceutical industry (Singh et al., 2011). One particular species, *Nostoc* sp., (Rawat and Bhargava, 2011; Niedermeyer, 2014) is responsible for the production of a considerable of bioactive cyclopeptide and cyclodepsipeptides with a variety of significant associated biological activities, including antiproliferative activities (Kang et al., 2012) antitoxin against Microcystins (Jokela et al., 2010), inhibition of chymotrypsin (Ploutno and Carmeli, 2002) antimicrobial activities (Ploutno and Carmeli, 2000), selective cytotoxicity (Trimurtulu et al., 1994). Some of the metabolic signatures of cyanobacterial peptides include a high degree of *N*-methylated amino acids, D-amino acids, and β-amino acids. We have been interested in marine secondary metabolites, especially the cyclopeptides and cyclodepsipeptides, and view their syntheses as playing a key role in structural confirmation, structural modification, and subsequent activity control. Previous work in our group led to the assignment/revision of a number of natural products including mandelalide A (Lei et al., 2014), lagunamide A (Dai et al., 2012), nocardioazine B (Wang et al., 2012), burkholdac A (Liu et al., 2012), bisebromoamide (Gao et al., 2010), and yanucamide A (Xu et al., 2003). Thus, we were encouraged to consider the synthesis of other natural products of uncertain configuration.

**Figure 1:**
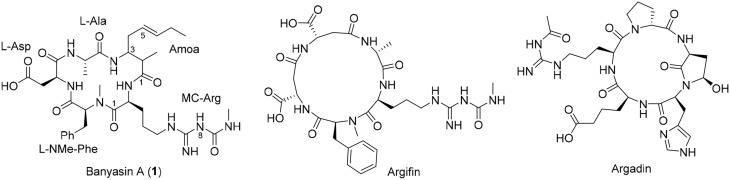
**The Structures of banyasin A (1), argifin and argadin**.

**Figure 2:**
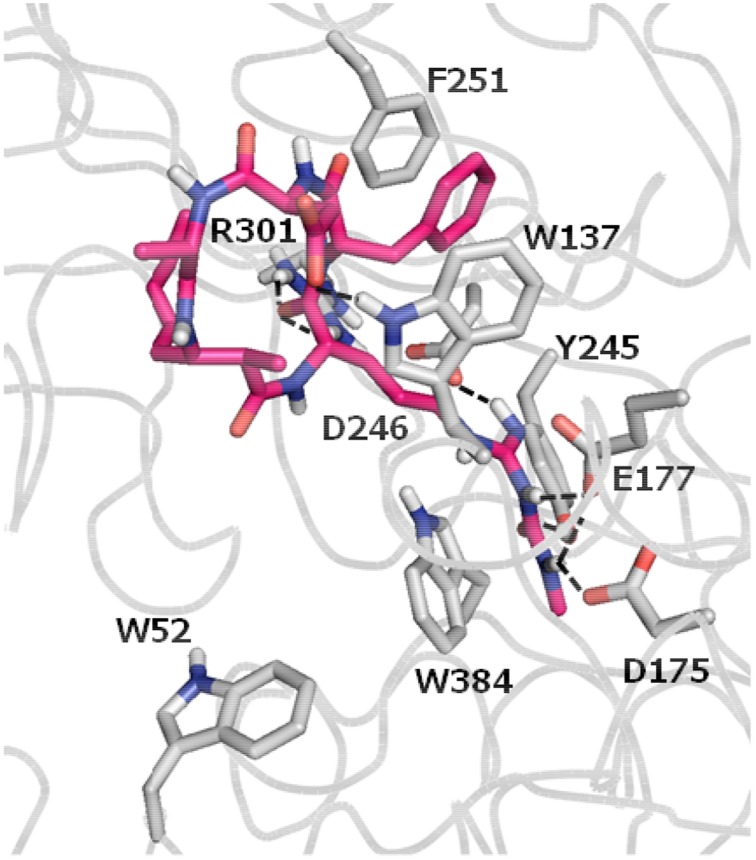
**Binding mode of banyasin A in *AfChiB1* active site**. Banyasin A **(1b)** (magenta carbon atoms) and the key interacting residues (gray carbon atoms) are shown in capped-sticks. Hydrogen bonds are displayed as black dashed lines, and non-polar hydrogens are removed for clarity.

In 2005, Carmeli and Pluotno reported the isolation of banyasin A (**1**) (Figure 1), from cyanobacterium *Nostoc* sp. (Pluotno and Carmeli, 2005). Structurally, banyasin A possesses a 16-membered macrocyclic peptide backbone which is composed of the unique, non-proteinogenic amino acids residues such as 3-amino-2-methyl-5*E*-octenoic acid and L-*N*_8_-(N-methylcarboxyaminde)arginine. Both the relative stereochemistry and absolute configuration of 3-amino-2-methyl-5*E*-octeneoic acid were unassigned in the isolation paper. The final structural determination had to await the total synthesis of the four possible diastereomeric structures proposed for the natural product. Banyasin A was isolated followed by a serine protease inhibition-guided protocol, however, pure banyasin A was found to be inactive toward trypsin inhibition. Banyasin A is structurally closely related to the known chitinase inhibitors argifin and argadin (Figure 1) (Arai et al., 2000; Omura et al., 2000). All these three natural products contained a highly modified arginine at the guanidine moiety. It is known that the dimethylguanylurea fragment of argifin was found to harbor all significant interactions with the chitinase and binds with unusually high efficiency. (Andersen et al., 2008). L-*N*_8_-(N-methylcarboxyaminde)arginine moiety played the critical role in binding of argifin to its cognate target (Dixon et al., 2009). The 16-membered macrolactam ring of banyasin A resemble the macrocyclic core of the known chitinase inhibitor argifin. Both banyasin A and argifin are comprised of L-*N*_8_-(N-methylcarboxyaminde)arginine residue. Modeling studies based on conformational analysis and manual docking yielded a binding model of banyasin A to a fungal chitinase (AfChiB1) active site, which has the most similar binding mode of argifin to AfChiB1 (Rao et al., 2005). As a representative compound, **1b** was displayed in Figure 2. The rest binding modes of banyasin's diastereomers (**1a**, **1c**, and **1d**) in AfChiB1 active site were included in support information. Taken together, these studies implied that banyasin A may serve as a potential lead compound for the development of antifungals and pesticides. The limited supply of banyasin A from its natural source has prevented its full biological characterization. In order to obtain sufficient material for more extensive biological evaluation as well as to determine the absolute configuration of banyasin A, we undertook research to develop a total synthesis of banyasin A with flexibility to enable future SAR development. Herein, we describe our efforts toward the total synthesis of banyasin A.

**Figure 3:**
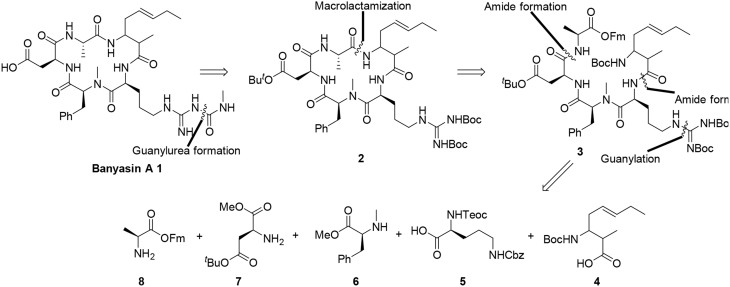
**Retrosynthetic analysis of banyasin A (1)**.

The retrosynthetic analysis of banyasin A (1) is illustrated in Figure 3. The *N*-methyl carbamoyl modification of the Arg residue will be conducted as the final step of the synthesis. There are a number of possible positions to close the macrocyclic portion (**2**) of the molecule (Figure 2). We chose to close the cyclopeptide via macrolactamization between the alanine and amoa residues because it allowed versatility in the construction of the cyclization precursor (**3**). Because the absolute configuration of the 3-amino-2-methyl-5*E*-octenoic acid residue was not established, the incorporation of four possible diastereomers of the 3-amino-2-methyl-5*E*-octenoic acid residue to afford linear precursor **3** could be exerted in the late stage. Further disconnection of the linear peptide **3** revealed amoa unit (**4**) and a tetrapeptide equivalent, the latter intermediate being traced to the protected amino acids **5**, **6**, **7**, and **8**. (Figure 3).

## Materials and methods

Experimental procedure and compound characterization data are furnished in the Supplementary Material.

## Results and discussion

**Figure 4:**
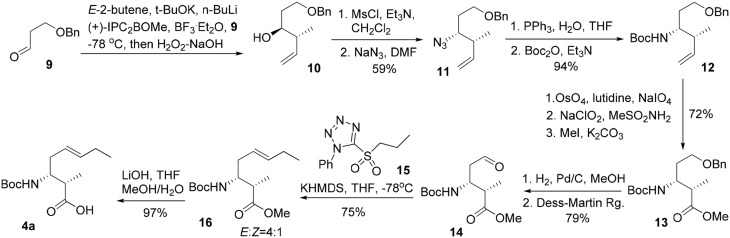
**Initial route for the synthesis of 3-amino-2-methyl-5*E*-octenoic acid 4**.

**Figure 5:**
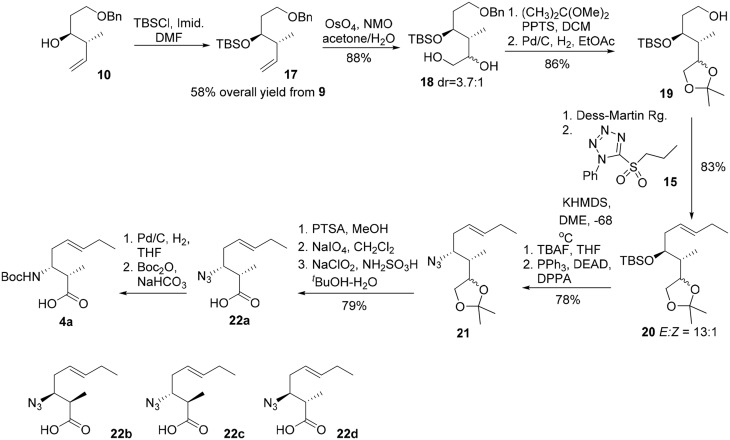
**Revised route for the synthesis of 3-amino-2-methyl-5*E*-octenoic acid 4**.

The synthesis commenced from crotylation of 3-benzyloxy-propionaldehyde (**9**) with Brown's (*E*)-(+)-crotyldiisopinocampheylborane, prepared from (+)-diisopinocampheyl(methoxy)borane, and yielded the *anti*-homoallylic alcohol **10** as a single diastereomer after silica gel chromatography (Figure 4). (Brown and Bhat, 1986) The homoallylic alcohol was then converted to its mesylate derivative, followed by exposure of the resulting mesylate to sodium azide in DMF furnished, with inversion of configuration, the corresponding azido derivative **11** in 59% overall yield from aldehyde **9**. The azide moiety of **11** was reduced by the action of triphenylphosphine to afford the corresponding primary amine, which was then protected as a Boc carbamate (**12**) in 94% yield over two steps. Oxidative cleavage of the olefin of **12** using OsO_4_–NaIO_4_ in the presence of 2,6-lutidine afforded an aldehyde, which was further oxidized to the corresponding carboxylic acid by Pinnick oxidation. A subsequent methylation of the carboxylic group using iodomethane and potassium carbonate gave the methyl ester **13** in 72% yield over the three steps. Subsequent removal of the benzyl ether under hydrogenative conditions followed by Dess-Martin oxidation of the resultant primary alcohol gave rise to aldehyde **14** in 79% yield. The Kocienski-modified Julia olefination of aldehyde **14** with sulfone **15** in THF at −78°C delivered olefin **16** as an inseparable mixture of olefin isomers (4:1) in favor of the desired *E* configuration. (Blakemore et al., 1998). Subsequent saponification of the methyl ester gave 3-amino-2-methyl-5*E*-octenoic acid **4a** in 97% yield. Further incorporation of **4a** in the construction of the macrocycle reveled the undesired geometrical isomer could not be completely removed in the later stage of the synthesis. Bearing in mind the problems encountered with the formation of an inseparable mixture of olefin isomers, we required an alternative approach which could efficiently deliver 3-amino-2-methyl-5*E*-octenoic acid **4** in fairly high purity. Thus, the homoallylic alcohol **10** was protected as its *tert*-butyldimethylsilyl ether and the terminal alkene underwent a substrate-controlled dihydroxylation in the presence of osmium tetroxide to provide **18** as a diastereomeric mixture (3.7:1) of diols in 88% combined yields (Figure 5). This mixture was simply exposed to 2,2-dimethoxypropane under acidic conditions to furnish the corresponding acetonide, which was then subjected to hydrogenolysis of the benzyl ether to give rise to **19** in 86% yield for two steps. Dess-Martin oxidation of **19** provided crude aldehyde, which was treated with the anion derived from sulfone **15** and KHMDS. When the olefination was performed in 1,2-dimethoxyethane (DME) at −68°C, we obtained *E/Z* mixtures (13/1) of olefins **20** in 83% yield. Removal of the TBS group in **20** using TBAF and the resultant secondary hydroxy group engaged in Mitsunobu reactions using diphenylphosphoryl azide (DPPA) (Lal et al., 1977) as the nucleophile to give rise to azide **21** in 78% yield. Acid-catalyzed deprotection of the acetonide functionality of **21**, followed by oxidative cleavage of the resulting diol with sodium periodate, followed by Pinnick oxidation (Bal et al., 1981) afforded the carboxylic acid **22a** in 79% yield over three steps. As the absolute configuration of two stereogenic centers in the subunit **4** was not assigned in the original isolation work, our synthetic strategy grew from the desire to have a versatile approach to make all four possible diastereomers of **4**, along with the defined stereochemistry of the disubstituted olefin component. With a practical route to **22a** in hand, we synthesized the other three possible diastereomers (**22b**, **22c**, and **22d**) through the condensation of aldehyde **9** with Brown's (*E*)-(−)-crotyldiisopinocampheylborane, (*Z*)-(+)-crotyldiisopinocampheylborane and (*Z*)-(−)-crotyldiisopinocampheylborane, respectively. Azido acid **22a** was then subjected to hydrogenation in the presence of the palladium on charcoal and the resulting amine was protected as its *tert*-butoxycarbonyl carbamate **4a**.

**Figure 6:**
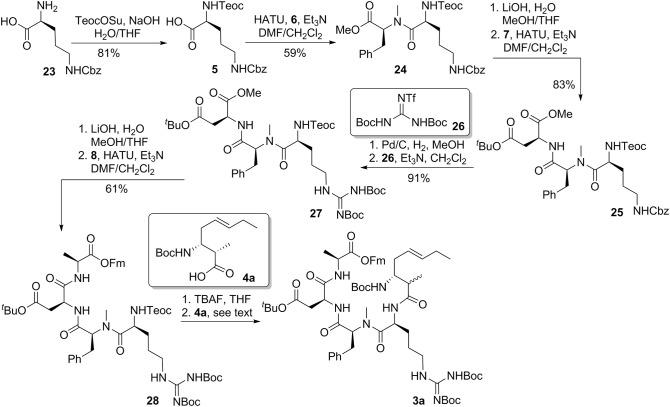
**Synthesis of the linear precursor 3**.

**Figure 7:**
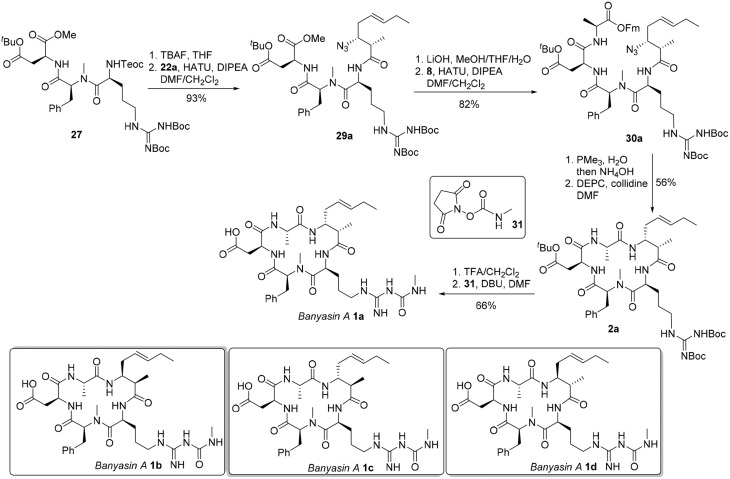
**Completion of the total synthesis of banyasin A (1)**.

With the critical amoa fragment in hand, we then turned our attention to the assembly of the remaining amino acids to a tetrapeptide and further elaboration to the linear precursor **3**. Thus, the 2-(trimethylsilyl)-ethoxycarbonyl (Teoc) protecting group was installed on the the alpha amino group of *N*-δ-Cbz-L-ornithine to afford the orthogonally protected ornithine **5** in 81% yield, which underwent a HATU-mediated coupling reaction with *N*-methyl-*L*-phenylalanine methyl ester (**6**) to provide dipeptide **24** in 59% yield (Figure 6) (Carpino, 1993). Saponification of the methyl ester of **24** produced the corresponding acid, which was then condensed with aspartate **7** in the presence of HATU and triethylamine to provide tripeptide **25** in 83% yield. Tripeptide **25** was submitted to catalytic hydrogenation to remove the benzyl protecting group, and the guanidine function was introduced by reaction with 1,3-di-Boc-2-(trifluoromethanesulfonyl)guanidine (**26**) (Feichtinger et al., 1998) to give **27** in 91% yield. Tripeptide **27** was further elongated to tetrapeptide **28** in 61% yield by a two step sequence, involving (1) hydrolysis of the methyl ester to its free acid, and (2) HATU-mediated condensation of the acid with L-alanine 9-fluorenylmethyl ester (**8**). This set the stage for the introduction of the 3-amino-2-methyl-5*E*-octenoic acid **4** to complete the synthesis of linear precursor **3**. In the event, the Teoc protecting group of **28** was removed in one step using TBAF in THF to provide the corresponding free amine. Unfortunately, condensation of this amine with acid **4a** using various coupling agents, including HATU, PyAOP, DCC and EDCI, turned out to be relatively slow and led to the formation of **3a** with significant epimerization at the center adjacent to the newly formed amide carbonyl. In general, formation of 20–35% of undesired epimer could be observed during the coupling reaction depending on the reagent employed. We speculated that the conformation of the tetrapeptide **28** may significantly affect the reactivity of the amine and resulted in a slow condensation reaction. Despite this significant setback to our synthetic plan toward banyasin A, we sought that to install the 3-amino-2-methyl-5*E*-octenoic acid unit (**4** or its masked form **22**) to the peptide at earlier stage might avoid epimerization of the C2-methyl group. Gratifyingly, HATU-mediated coupling reaction of azido acid **22a** with the amine derived from tripeptide **27** afforded **29a** in 93% yield. That no epimerization had occurred during this coupling reaction was confirmed by synthesizing the corresponding epimeric material through the condensation of the same amine with azide acid **22c**. Saponification of the methyl ester of **29a** and subsequent condensation of the resultant acid with L-alanine 9-fluorenylmethyl ester (**8**) afforded **30a** in 82% yield. Of the various reduction protocols that were examined for converting azido acid **30a** into the corresponding amine, the Staudinger reduction with PMe_3_ in THF:H_2_O (7:1) proved the most successful. In the event, the Staudinger reduction of the azide and the cleavage of 9-fluorenylmethyl ester with aqueous NH_4_OH were carried out in a one-pot procedure to afford the resultant amino acid, which was immediately activated by diethyl cyanophosphonate (DEPC) (Yamada et al., 1973) in the presence of collidine to afford cyclopeptide **2a** in 56% yield over two steps. Simultaneous removal of the *tert*-butyl ester and Boc-protecting group was achieved by treatment of **2a** with trifluoroacetic acid in dichloromethane at room temperature. The guanidino group of the resultant cyclopeptide was mono-acylated with *N*-succinimidyl *N*-methylcarbamate (**31**) in the presence of DBU to afford banyasin A **1a** in 66% yield. (Dixon et al., 2005) Having one diastereomer (**1a**), and a practical route to bayansin A in hand, we set out to pursue total syntheses of the other three possible diastereomers. This was readily achieved by following the same synthetic procedure as for **1a**, by employing azido acids **22b**, **22c**, and **22d** as key building blocks. These syntheses proceeded smoothly under the previous conditions to give rise to three diastereomers of banyasin A, **1b**, **1c**, and **1d** in 28, 27, and 30% overall yield from tripeptide **27** (Figure 7).

## Conclusions

**Figure 8:**
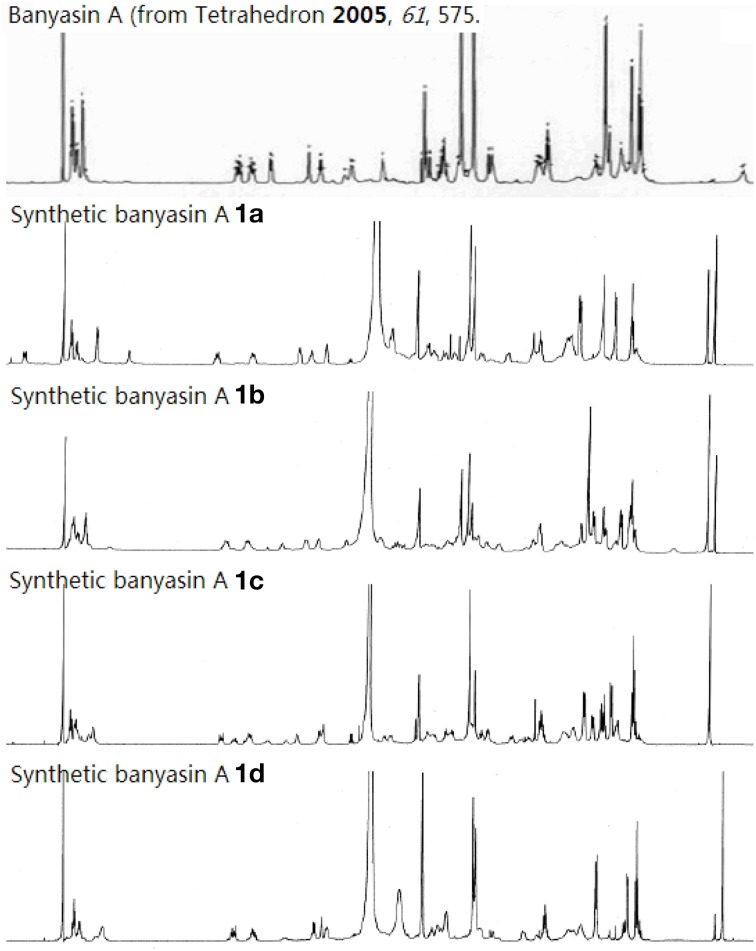
**Comparison of ^1^H NMR of natural and synthetic banyasin A (1a–d)**.

We have developed a convergent route for the synthesis of the proposed structure of banyasin A. The optical rotation measured for synthetic banyasin A **1a–d** ([α]^20^_*D*_ = −45.6 (*c* = 0.70, MeOH); [α]^20^_*D*_ = −59.4 (*c* = 0.70, MeOH); [α]^20^_*D*_ = −37.3 (*c* = 0.70, MeOH); and [α]^20^_*D*_ = −53.1 (*c* = 0.70, MeOH); were identical or nearly identical to that reported for the natural product [α]^20^_*D*_ = −45.3 (*c* = 0.7, MeOH). To our disappointment, both the ^1^H and ^13^C spectroscopic properties of each diastereomer **1a**, **1b**, **1c**, and **1d** were different from those of banyasin A (Figure 8 and Supplementary Material). It would appear that the error in the original assignment of the configuration of banyasin A lies somewhere in the remaining α-aminoacids. The extension of this chemistry toward the synthesis of additional diastereomers aiming to resolve the true structure of natural banyasin A and synthesis of novel analogs of banyasin for biological evaluation are underway in our laboratory.

### Conflict of interest statement

The authors declare that the research was conducted in the absence of any commercial or financial relationships that could be construed as a potential conflict of interest.
