# Supplementary material for: Total synthesis of the putative structure of the proposed Banyasin A
Source: Front Chem. 2015 Mar 17;3:19. doi: 10.3389/fchem.2015.00019 (PMC4362330; doi:10.3389/fchem.2015.00019)
Supplement: Supplementary file 1 [file DataSheet1.PDF]

Supplementary Material

Total Synthesis of the Proposed Banyasin A

Xuguang Gao,[1] Qi Ren,[1] Sun Choi,[2] Zhengshuang Xu,[1]* Tao Ye[1,3]*

[1] Laboratory of Chemical Genomics, School of Chemical Biology and Biotechnology, Peking University Shenzhen Graduate School, Xili, Nanshan District, Shenzhen, 518055, China, E-mail: xuzs@pkusz.edu.cn
[2] National Leading Research Laboratory (NLRL) of Molecular Modeling & Drug Design, College of Pharmacy, Graduate School of Pharmaceutical Sciences, and Global Top 5 Program, Ewha Womans University, Seoul 120-750, Republic of Korea
[3] Department of Applied Biology & Chemical Technology, The Hong Kong Polytechnic University, Kowloon, Hong Kong, E-mail: tao.ye@polyu.edu.hk, tao_ye35@hotmail.com

*** Correspondence:** Tao Ye, Department of Applied Biology & Chemical Technology, The Hong Kong Polytechnic University, Kowloon, Hong Kong, E-mail: tao_ye35@hotmail.com; Zhengshuang Xu, Laboratory of Chemical Genomics, School of Chemical Biology and Biotechnology, Peking University Shenzhen Graduate School, Xili, Nanshan District, Shenzhen, 518055, China, E-mail: xuzs@pkusz.edu.cn

Supplementary Data

Molecular Modeling Studies

The 3D structures of banyasin A were produced using Concord and energy minimized with MMFF94s force field and MMFF94 charge in SYBYL-X 2.0 (Tripos Int., St. Louis, MO, USA). The X-ray crystal structure of *Af*ChiB1 in complex with argifin (PDB ID: 1W9V) [Rao, F. V.; Houston, D. R.; Boot, R. G., Aerts, J.M.F.G., Hodkinson, M.; Adams, D. J.; Shiomi, K.; Ōmura, S.; van Aalten, D. M.F. (2005) Specificity and Affinity of Natural Product Cyclopentapeptide Inhibitors against A. fumigatus, Human, and Bacterial Chitinases *Chem Biology*, *12*, 65–76. DOI 10.1016/j.chembiol.2004.10.013.] was selected and prepared with the Biopolymer Structure Preparation tool in SYBYL. The docking study was performed using Standard Precision (SP) mode of Glide (version 6.1, Schrödinger, LLC, New York, NY, USA). All the computation calculations were carried out on an Intel® Xeon™ Quad-core 2.5 GHz workstation with Linux Cent OS release 5.5.

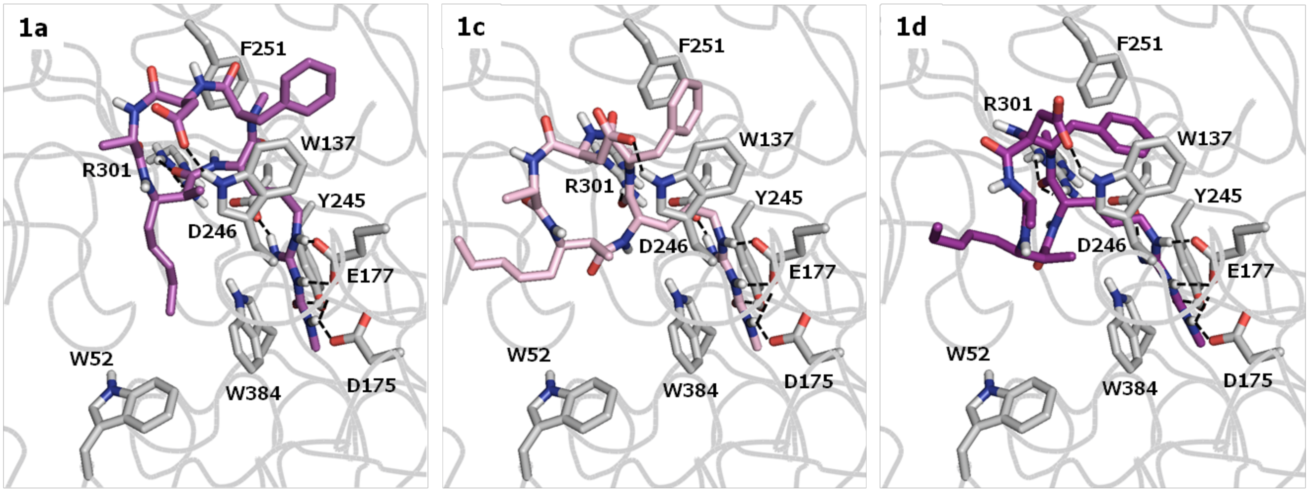

Figure S1. Binding modes of banyasin A **1a**, **1c**, and **1d** in AfChiB1 active site. Banyasin A diastereomers and the key interacting residues (gray carbon atoms) are shown in capped-sticks. Hydrogen bonds are displayed as black dashed lines, and non-polar hydrogens are removed for clarity.

We have employed all the diastereomers **1a-1d** in the docking studies. Compounds **1b**, **1c**, and **1d** showed very similar binding modes to argifin, maintaining the key interactions of the L-N_8-(*N*-methylcarboxyaminde)arginine, benzyl, and carboxyl groups. It appeared that the 3-amino-2-methyl-5*E*-octenoic acid unit neither contributed to nor interfered with their binding to *Af*ChiB1 active site. In the case of **1a**, the 3-amino-2-methyl-5*E*-octenoic acid formed hydrophobic interactions with binding site residues. That might have pushed the benzyl group upward, resulting in its hydrophobic interaction with Trp137 instead of Phe251.

General Experimental

Non-aqueous reactions were carried out under inert atmosphere (nitrogen or argon) with rigid exclusion of moisture from reagents in oven-dried reaction vessels. Solvents were distilled prior to use: THF (tetrahydrofuran) from Na/benzophenone, DCM (dichloromethane), DMF (dimethylformamide), 2,6-lutidine, DEA (diethylamine), TEA (triethylamine) and DIPEA (diisoproylethylamine) from CaH_2. MeOH (methanol) was distilled from Mg/I_2. Flash column chromatography was performed using the indicated solvents on E. Qingdao silica gel 60 (230 – 400 mesh ASTM). TLC (Thin layer chromatography) was carried out using pre-coated sheets (Qingdao silica gel 60-F250, 0.2 mm) which, after development, were visualized at 254 nm, and/or staining in *p*-anisole, ninhydrin or phosphomolybdic acid solution followed by heating. NMR spectra were recorded on Bruker DPX 300 MHz or AV 500 MHz spectrometers. Chemical shifts were reported in parts per million (ppm), relative to either a tetramethylsilane internal standard or the signals due to the solvent. Data were reported as follows: chemical shift, multiplicity (s = singlet, d = doublet, t = triplet, q = quartet, br = broad), coupling constants (Hz) and integration. High resolution mass spectra were measured on ABI Q-star Elite. Optical rotations were recorded on a Perkin-Elmer 351 polarimeter at 589 nm, 100 mm cell at 20 ^{o}C. Data were reported as follow: optical rotation (*c* (g/100 mL), solvent).

Molecular Modeling Studies

The 3D structures of banyasin A were produced using Concord and energy minimized with MMFF94s force field and MMFF94 charge in SYBYL-X 2.0 (Tripos Int., St. Louis, MO, USA). The X-ray crystal structure of AfChiB1 in complex with argifin (PDB ID: 1W9V) [Rao et al 2005] was selected and prepared with the Biopolymer Structure Preparation tool in SYBYL. The docking study was performed using Standard Precision (SP) mode of Glide (version 6.1, Schrödinger, LLC, New York, NY, USA). All the computation calculations were carried out on an Intel® XeonTM Quad-core 2.5 GHz workstation with Linux Cent OS release 5.5.

Experimental Procedures

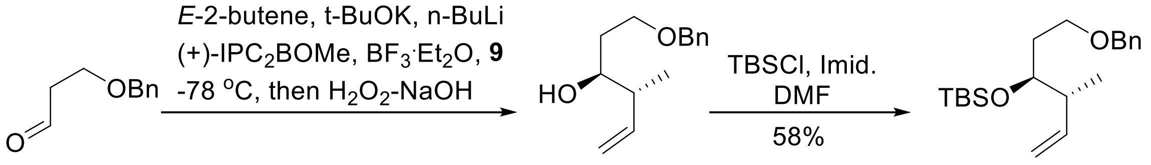

9 **10** **17**

To a stirred mixture of ^{t}BuOK (4.50 g, 40 mmol, dried under high vacuum at 80 ℃ for 10 h) and *trans*-2-butene (8.30 mL, 89 mmol) in THF (30 mL) was added *n*BuLi (20 mL, 40 mmol, 2.0 M in hexane) at –78 ℃. The mixture was stirred at –45 ℃ for 10 min and cooled to –78 ℃. Methoxy-diisopinocampheylborane (16.0 g, 50 mmol) in THF (20 mL) was dropwise added. 30 min later, $BF_3 \cdot Et_2O$ (6.5 mL, 54 mmol) was added dropwise, followed by addition of a solution of aldehyde **9** (8.21 g, 50 mmol) in THF (5 mL). The reaction mixture was stirred for 3 h at –78 ℃ and then quenched by careful addition of methanol (3 mL) and warmed to 0 ℃. NaOH (29 mL, 3 N aqueous solution) and H_2O_2 (15 mL, 30% in water) were subsequently added, then the reaction mixture were refluxed for 2 h. After the reaction mixture was cooled to room temperature, the volatiles were removed in *vacuo*. The residue was extracted with diethyl ether (50 mL x 3). The combined organic layers were washed with water (50 mL) and brine (15 mL), dried over Na_2SO_4 and concentrated in *vacuo* to provide crude product **10**. The residue was dissolved in DMF (100 mL) at 0 ℃. To this solution, TBSCl (39.3 g, 260 mmol) and imidazole (26.6 g, 390 mmol) were added successively. After being stirred at 0 ℃ for 10 min the solution was slowly warmed to 40 ℃ and stirred for 12 h. Ethyl ether (300 mL) and water (200 mL) were added to quench the reaction. Layers were separated, and the aqueous phase was extracted with ethyl ether (150 mL x 2). The combined organic layers were washed with citric acid (100 mL, 10% aqueous solution), water (100 mL), brine (100 mL) and dried over Na_2SO_4, and concentrated in *vacuo*. The residue was purified by flash chromatography on silica gel (EtOAc/Hexane: 1/50) to afford compound **17** (7.75 g, 58%, two steps from compound **9**) as a colorless oil.

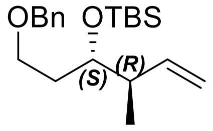

17a

Compound **17a** was prepared from *E*-2-butene and (+)-Ipc_2BOMe. Analytical data for **17a**: $[\alpha]_D^{20} = -11.3$ (c = 0.67, MeOH); ^1H NMR (500 MHz, $CDCl_3$) δ = 7.30-7.36 (m, 3H), 7.24-7.27 (m, 2H), 5.77 (ddd, J = 16.9, 10.8, 7.5 Hz, 1H), 4.95-5.00 (m, 2H), 4.48 (dt, J = 12.0 Hz, 2H), 3.78 (dt, J = 7.6, 4.2 Hz, 1H), 3.51 (dt, J = 4.2, 1.2 Hz, 2H), 2.3 (m, 1H), 1.69 (m, 1H), 1.10 (d, J = 6.9 Hz, 3H), 0.89 (s, 9H), 0.05 (s, 3H), 0.04 (s, 3H); ^{13}C NMR (75 MHz, $CDCl_3$) δ = 140.7, 138.6, 128.3, 127.6, 127.5, 114.6, 72.9, 72.5, 67.4, 43.4, 33.2, 25.9, 18.1, 14.5, –4.4, –4.6 ppm; HRMS

(ESI) m/z calcd for $C_{20}H_{34}O_2SiNa$ $(M+Na)^+$ 357.2226, found 357.2236.

Compound **17b** was prepared from *E*-2-butene and (-)-Ipc_2BOMe. **17b** is the enantiomer of compound **17a**.

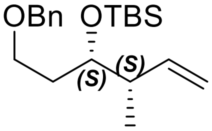

17c

Compound **17c** was prepared from *Z*-2-butene and (+)-Ipc_2BOMe. Analytical data for **17c**: $[\alpha]_D^{20} = -14.4$ (*c* = 5.7, MeOH); ^1H NMR (300 MHz, $CDCl_3$) δ = 7.269 - 7.353 (m, 5H), 5.89 (ddd, *J* = 17.1, 10.8, 6.6 Hz, 1H), 5.04 (s, 1H), 4.99 (d, *J* = 9.0 Hz, 1H), 4.49 (d, *J* = 5.7 Hz, 2H), 3.74 (dt, *J* = 7.8, 4.5 Hz, 1H), 3.54 (t, *J* = 6.3 Hz), 2.27-2.37 (m, 1H), 1.63-1.78 (m, 2H), 0.97 (d, *J* = 6.9 Hz), 0.89 (s, 9H), 0.05 (d, *J* = 3.6 Hz, 6H); ^{13}C NMR (125 MHz, $CDCl_3$) δ = 140.9, 138.7, 128.3, 127.6, 127.5, 114.2, 73.1, 72.9, 43.1, 33.6, 25.9, 18.2, 14.9, –4.3 and –4.5 ppm.

Compound **17d** was prepared from *Z*-2-butene and (-)-Ipc_2BOMe. **17d** is the enantiomer of compound **17c**.

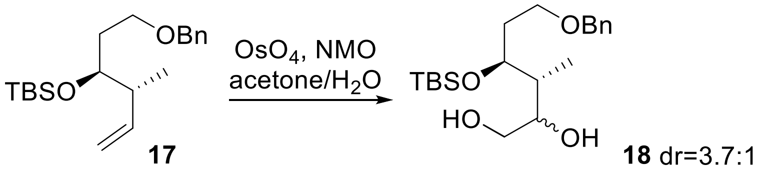

To a solution of alkene **17** (1.0 g, 2.99 mmol) in acetone/H_2O (24 mL, v:v = 5:1) at 0 °C, NMO (0.52 g, 4.48 mmol) and OsO_4 (2 mL, 2.5% in ^{t}BuOH) were added. The reaction mixture was stirred at room temperature for 3 h. Aqueous solution of $Na_2S_2O_3$ (50 mL) was added to quench the reaction. Volatiles were removed in *vacuo*, the residue was extracted with EtOAc (50 mL x 3). The combined organic layers were washed with water (100 mL) and brine (100 mL), dried over Na_2SO_4 and concentrated in *vacuo*. The residue was purified by flash chromatography on silica gel (EtOAc/Hexane: 1/2) to give compound **18** (0.97 g, 88%, 2 diastereomers with 3.7:1 ratio) as a colorless oil.

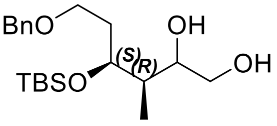

18a

Analytical data for **18a** and its enantiomer **18b**: ^1H NMR (500 MHz, $CDCl_3$) δ = 7.29-7.37 (m, 5H), 4.51 (s, 2H), 4.03 (m, 1H), 3.71 (dd, *J* = 11.0, 3.0 Hz, 1H), 3.56-3.60 (m, overlp, 2H), 3.54-3.56 (m, overlp, 1H), 3.47 (dd, *J* = 10.5, 7.0 Hz, 1H), 1.86-1.93 (m, 1H), 1.82-1.75 (m, overlp, 1H), 1.71-1.76 (m, overlp, 1H), 0.89 (s, 9H), 0.85 (d, *J* = 6.5 Hz, 3H), 0.08 (s, 3H), 0.06 (s, 3H); ^{13}C NMR (125 MHz, $CDCl_3$) δ = 138.3, 128.41 (major) and 128.37, 127.8, 127.68 (major) and 127.64, 74.1, 73.2 (major) and 73.0, 72.2, 67.3 (major) and 66.7, 65.4 and 64.9 (major), 41.0, 34.9 and 34.7 (major), 25.8, 18.0, 11.8 and 11.6 (major), –4.6 ppm; HRMS (ESI) m/z calcd for $C_{20}H_{36}O_4SiNa$ $(M+Na)^+$ 391.2281, found 391.2299.

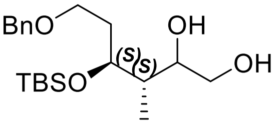

18c

Analytical data for **18c** and its enantiomer **18d**: ^1H NMR (500 MHz, $CDCl_3$) δ = 7.24-7.36 (m, 5H), 4.49 (AB, *J* = 27.5, 11.9Hz, 2H), 4.01 (m, 1H), 3.68 (d, *J* = 9.0 Hz), 3.55 (m, 2H), 3.47 (dd, *J* = 12.2, 5.3 Hz, 1H), 1.95(m, 1H), 1.84 (m, 2H), 0.89 (s, 9H), 0.78 (d, *J* = 7.0 Hz, 3H), 0.12 (s, 3H), 0.07 (s, 3H); ^{13}C NMR (125 MHz, $CDCl_3$) δ = 138.4, 128.4, 127.7, 127.6, 74.2, 74.1, 72.9,

66.9, 64.9, 39.1, 31.7, 25.8, 17.9, 13.1, –4.4, –5.0 ppm.

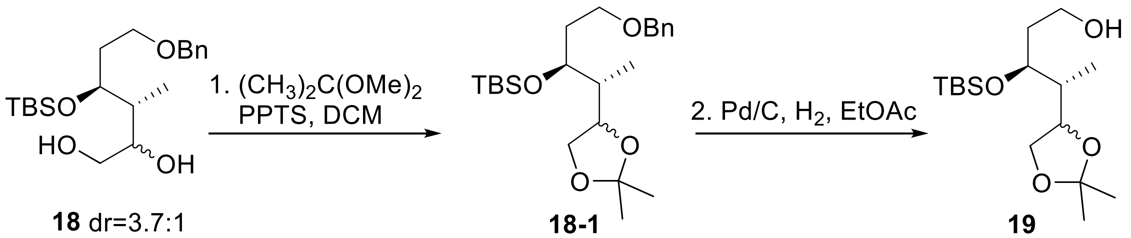

18 dr=3.7:1 **18-1** **19**

To a solution of diol **18** (0.97 g, 2.63 mmol) in DMP/CH_2Cl_2 (44 mL, v:v = 1:10), PPTS (70 mg, 0.027 mmol) was added at room temperature. The reaction mixture was stirred for 16 h and then diluted with CH_2Cl_2 (40 mL) and washed with saturated Na_2CO_3 (30mL) and brine (20 mL). The organic phase was dried with Na_2SO_4 and concentrated in *vacuo*. The residue was purified by flash chromatography on silica gel (EtOAc/Hexane: 1/15) to give compound **18-1** as a colorless oil (1.05 g, 98%).

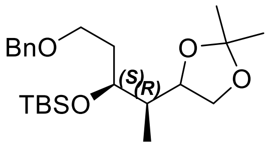

18-1a

Analytical data for **18-1a** and its enantiomer **18-1b**: ^{1}H NMR (300 MHz, $CDCl_3$) δ = 7.18-7.41(m, 5H), 4.52 (s, 2H), 3.93-4.07 (m, 3H), 3.55-3.64 (m, 3H), 1.84-1.91 (m,1H), 1.73-1.83 (m, 2H), 1.40 (s, 3H), 1.35 (s, 3H), 0.89 (s, 9H), 0.99 (d, J = 6.6 Hz) and 0.85 (d, J = 7.2 Hz, major, 3H), 0.05 (d, J = 3.3 Hz, 6H); ^{13}C NMR (125 MHz, $CDCl_3$) δ = 138.6, 127.6, 127.5, 127.4, 108.4, 76.9, 73.0 and 72.9 (major), 70.2, 68.2 and 68.1 (major), 67.9, 42.9 and 42.5 (major), 32.2, 26.7, 25.9, 25.7, 18.0, 9.9, –4.47 and –4.53 (major), –4.7 ppm; HRMS (ESI) m/z calcd for $C_{23}H_{40}O_4SiNa$ $(M+Na)^+$ 431.2594, found 431.2610.

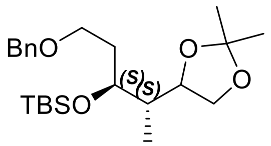

18-1c

Analytical data for **18-1c** and its enantiomer **18-1d**: ^{1}H NMR (300 MHz, $CDCl_3$) δ = 7.29-7.36 (m, 5H), 4.50 (d, J = 5.7 Hz, 2H), 4.17(m, 1H), 3.93-4.04 (m, 2H), 3.46-3.58 (m, 3H), 1.80 (dt, J = 10.2, 7.2 Hz, 2H), 1.60 (m, 1H), 1.39 (s, 3H), 1.35 (s, 3H), 0.89 (s, 9H), 0.77 (d, J = 6.9 Hz, 3H), 0.075 (d, J = 4.5 Hz, 6H); ^{13}C NMR (75 MHz, $CDCl_3$) δ = 138.3, 128.3, 127.5, 127.3, 108.3, 76.7, 72.8, 68.5, 67.2, 41.4, 35.0, 26.8, 25.8, 25.6, 18.0, 8.3, –4.4, –4.8 ppm.

To a solution of compound **18-1** (0.41 g, 1.0 mmol) in ethyl acetate (10 mL), Pd/C (0.11 g, 10% on charcoal) was added. The reaction vessel was sealed and exposed to H_2 (balloon). The reaction mixture was then vigorously stirred for 20 h and filtered through a short pad of silica gel. The filtrate was concentrated and purified by flash chromatography on silica gel (EtOAc/Hexane: 1/9) to afford alcohol **19** (0.28 g, 88%) as a colorless oil.

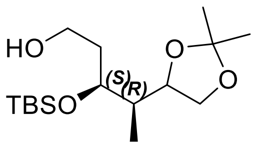

19a

Analytical data for **19a** and its enantiomer **19b**: ^{1}H NMR (300 MHz, $CDCl_3$) δ = 4.10-4.18 (m, 1H), 4.02 (d, J = 7.8 Hz, 1H), 3.81-3.89 (m, 1H), 3.68-3,81 (m, 2H), 3.60 (d, J = 7.8 Hz, 1H), 1.80-1.92 (m, 1H), 1.72 (dt, J = 6.0, 9.0 Hz , 2H), 1.39 (s, 3H), 1.34 (s, 3H), 0.90 (s, 9H), 0.83 (d, J = 6.9 Hz, 3H), 0.097 (d, J = 6.6 Hz, 6H); ^{13}C NMR (75 MHz, $CDCl_3$) δ = 108.68, 76.45, 72.69 and 71.58 (major), 68.46 and 67.7 (major), 60.92 and 59.8 (major), 42.9, 33.3, 26.6 and 26.3 (major), 25.7 (major) and 25.6, 25.2, 17.8, 9.6 (major) and 9.4, –4.6, –4.7 and –4.8 (major) ppm; HRMS (ESI) m/z calcd for $C_{16}H_{34}O_4SiNa$ $(M+Na)^+$

341.2124, found 341.2137.

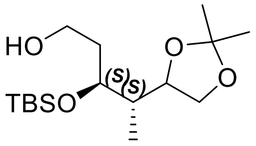

19c

Analytical data for **19c** and its enantiomer **19d**: ^{1}H NMR (300 MHz, $CDCl_3$) δ = 4.12 (dt, J = 6.6, 2.4 Hz, 1H), 3.92-4.04 (m, 2H), 3.68 (t, J = 6.3 Hz, 2H), 3.53 (t, J = 7.5 Hz, 1H), 1.90 (br, 1H), 1.75 (dt, J = 9.6, 3.3 Hz, 2H), 1.64 (m, 1H), 1.38 (s, 3H), 1.34 (s, 3H), 0.89 (s, 9H), 0.79 (d, J = 6.9 Hz, 3H), 0.09 (s, 6H); ^{13}C NMR (75 MHz, $CDCl_3$) δ = 108.4, 69.1, 68.5, 59.8, 41.6, 37.7, 26.7, 25.8, 25.7, 18.0, 8.8, –4.4, –4.8 ppm.

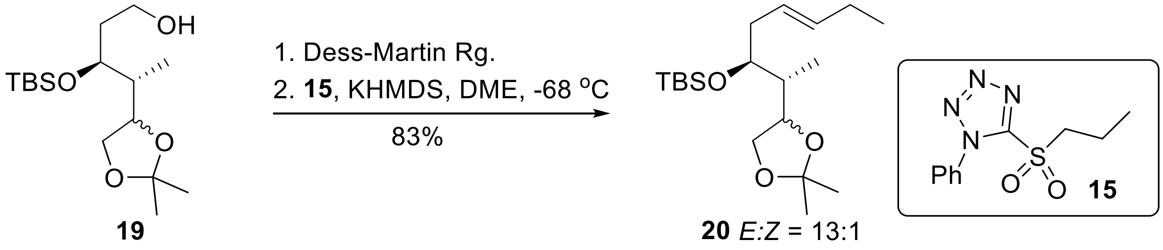

To a cold solution of compound **19** (0.50 g, 1.57 mmol) in CH_2Cl_2 (20mL) Dess-Martin periodinane (1.31 g, 3.10 mmol) was added in one portion. The reaction mixture was stirred for 30 min, and filtered through a short pad of silica gel and eluted with diethyl ether (100 mL). The filtrate was washed with aqueous solution of $Na_2S_2O_3$ (50 mL, 10% in water) and brine (50 mL). The organic phase was dried over Na_2SO_4 and concentrated in *vacuo* to produce the corresponding aldehyde as a colorless oil, which was used directly in the next step.

To a solution of **15** (0.59 g, 2.36 mmol) in DME (30mL), KHMDS (3.15 mL, 0.5 M in toluene) was added at –68 ℃. 30 min later, a solution of the aldehyde in DME (10 mL) was added slowly. The reaction mixture was allowed to warm to room temperature within 2 h and quenched by addition of a saturated aqueous solution of NH_4Cl (30 ml). After volatiles were removed under reduced pressure, the residue was partitioned between ethyl ether (100 mL) and water (50 mL). Layers were separated, the aqueous phase was extracted with ethyl ether (50 mL x 2). The combined organic layers were washed with brine (50 mL), dried over Na_2SO_4 and concentrated in *vacuo*. The residue was purified by flash chromatography on silica gel (EtOAc/Hexane: 1/99) to offer the product **20** (0.45 g, 83% yield) as a colorless oil.

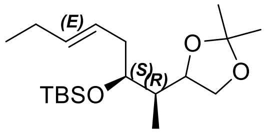

20a

Analytical data for **20a** and its enantiomer **20b**: ^{1}H NMR (500 MHz, $CDCl_3$) δ = 5.50 (dt, J = 15.2, 6.0 Hz, 1H), 5.40 (dt, J = 15.1, 6.9 Hz, 1H), 4.18-4.15 (m) and 4.08-4.04 (m, major, 1H), 4.01-3.95 (m, 1H), 3.81-3.78 (m, 1H), 3.63-3.56 (m, 1H), 2.22-2.16 (m, 1H), 2.13-2.08 (m, 1H), 2.04-2.01 (m, 2H), 1.92-1.96 (m, 1H), 1.39 (s, 3H), 1.34 (s, major) and 1.32 (s, minor, 3H), 0.97 (t, J = 7.2 Hz, 3H), 0.89 (s, 9H), 0.84 (d, J = 6.9 Hz, 3H), 0.04(s, 6H); ^{13}C NMR (125 MHz, $CDCl_3$) δ = 134.6 and 134.2 (major), 125.9 (major) and 124.9, 108.3 and 108.0 (major), 76.5 (major) and 76.4, 68.2 and 67.5 (major), 41.3 (major) and 41.1, 37.1 and 36.5 (major), 29.7 (major) and 29.3, 26.6 (major) and 26.5, 25.9, 25.7 (major) and 25.6, 18.11 and 18.06 (major), 13.7 and 13.6 (major), 10.41 (major) and 10.37, –4.4, –4.6 ppm; HRMS (ESI) m/z calcd for $C_{19}H_{38}O_3SiNa$

$(M+Na)^+$ 365.2488, found 365.2502.

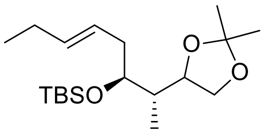

20c

Analytical data for **20c** and its enantiomer **20d**: ^{1}H NMR (300 MHz, $CDCl_3$) δ = 5.49 (dt, J = 15.3, 6.3 Hz, 1H), 5.30 (dt, J = 15.3, 6.3 Hz, 1H), 3.91-4.03 (m, 3H), 3.54 (t, J = 6.9 Hz, 1H), 2.14 (m, 2H), 2.00 (m, 2H), 1.64 (m, 1H), 1.38 (s, 3H), 1.34 (s, 3H), 0.97 (t, J = 7.5Hz, 3H), 0.89 (s, 9H), 0.75 (d, J = 6.6Hz, 3H), 0.06 (d, J = 0.9Hz, 6H); ^{13}C NMR (75 MHz, $CDCl_3$) δ = 134.5, 125.4, 108.6, 77.4, 71.3, 68.8, 40.6, 38.7, 27.1, 26.1, 26.0, 25.8, 18.3, 13.9, 8.1, –3.9, –4.6 ppm.

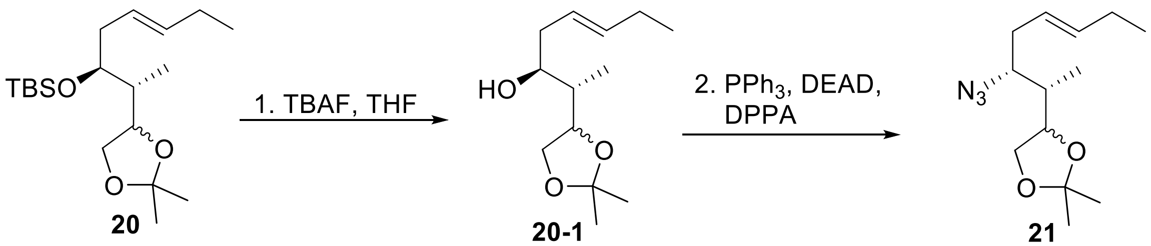

20 **20-1** **21**

To a solution of **20** (1.08 g, 3.15 mmol) in THF (25 mL), TBAF (6.5 mL, 1.0M in THF) was slowly added at room temperature. The reaction mixture was stirred for 16 h and quenched by addition of a saturated aqueous solution of NH_4Cl (30 mL). Volatiles were removed under reduced pressure. The residue was partitioned between EtOAc (100 mL) and water (50 mL). Layers were separated, the aqueous phase was back extracted with EtOAc (50 mL). The combined organic layers were washed with brine (50 mL) and dried over Na_2SO_4. Solvents were removed in *vacuo*, the residue was purified by flash chromatography on silica gel (EtOAc/Hexane: 1/9) to produce compound **20-1** (0.65 g, 91%) as a colorless oil.

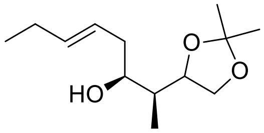

20-1a

Analytical data for **20-1a** and its enantiomer **20-1b**: ^{1}H NMR (300 MHz, $CDCl_3$) δ = 5.45-5.64 (m, 2H), 4.04-4.11 (m, 1H), 3.99-4.04 (m, overlp, 1H), 3.74-3.68 (m, overlp, 1H), 3.61-3.68(m, overlp, 1H), 3.35 (s, 1H), 2.31-2.35 (m, 1H), 2.10-2.14 (m, overlp, 1H), 2.00-2.14 (m, 2H), 1.74-1.79 (m, 1H), 1.41 (s, 3H), 1.37 (s, major) and 1.35 (s, 3H), 0.99 (t, J = 7.2 Hz, 3H), 0.80 (d, J = 6.9 Hz, 3H); ^{13}C NMR (125 MHz, $CDCl_3$) δ = 136.3 and 135.3 (major), 125.0 (major) and 124.7, 109.3 (major) and 108.4, 79.6, 74.4 (major) and 73.0, 68.6 (major) and 67.7, 41.6 (major) and 40.3, 38.2 and 37.2 (major), 26.6 (major) and 26.3, 25.7, 25.6 (major) and 25.3, 13.8, 12.0 (major) and 11.5 ppm; HRMS (ESI) m/z calcd. for $C_{13}H_{24}O_3Na$ $(M+Na)^+$ 251.1623, found 251.1617.

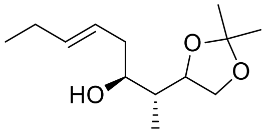

20-1c

Analytical data for **20-1c** and its enantiomer **20-1d**: ^{1}H NMR (500 MHz, $CDCl_3$) δ = 5.58 (dt, J = 15.3, 6.3 Hz, 1H), 5.43 (dt, J = 15.3, 6.3 Hz, 1H), 4.03-4.11 (m, 2H), 3.83 (br, 1H), 3.63 (t, J = 7.6 Hz, 1H), 2.21 (br, 1H), 2.15-2.20 (m, 2H), 2.00-2.06 (m, 2H), 1.75 (m, 1H), 1.41 (s, 3H), 1.36 (s, 3H), 0.98 (t, J = 7.5 Hz, 3H), 0.86 (d, J = 7.1 Hz, 3H); ^{13}C NMR (75 MHz, $CDCl_3$) δ = 135.5, 125.6, 109.0, 78.5, 71.5, 68.5, 40.8, 37.7, 26.9, 25.8, 25.7, 13.9, 10.1 ppm.

To a solution of **20-1** (0.59 g, 2.58 mmol) and PPh_3 (2.62 g, 10.0 mmol) in THF (50 mL), DEAD (1.65 mL, 10.0 mmol) and DPPA (2.15 mL, 10.0 mmol) were successively added at 0 ℃, The resulting solution was allowed to warm to room temperature and stirred overnight. The reaction mixture was concentrated in *vacuo*. The residue was purified by flash chromatography on silica gel (EtOAc/Hexane: 1/50) to provide compound **21** (0.54 g, 86%) as a colorless oil.

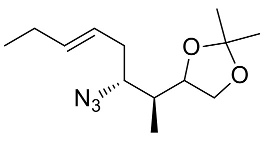

21a

Analytical data for **21a** and its enantiomer **21b**: ^{1}H NMR (300 MHz, $CDCl_3$) δ = 5.63 (dt, J = 15.3, 6.0Hz, 1H), 5.40 (dt, J = 15.3, 6.0 Hz, 1H), 4.00-4.07 (m, overlp, 1H), 3.95-4.00 (m, overlp, 1H), 3.78-3.84 (m, 1H), 3.59 (dd, J = 7.2, 7.2 Hz, 1H), 2.33-2.43 (m, 1H), 2.11-2.22 (m, 1H), 2.00-2.09 (m, 2H), 1.71-1.76 (m, 1H), 1.41 (s) and 1.39 (s, major, 3H), 1.36 (s, 3H), 0.99 (t, J = 7.2 Hz, 3H), 0.78 (d, J = 6.9 Hz, 3H); ^{13}C NMR (75 MHz, $CDCl_3$) δ = 136.1, 124.4, 109.0, 77.6, 68.3, 63.0, 40.7 (major) and 39.7, 35.7, 27.0 (major) and 26.7, 25.80, 25.75 (major) and 25.6, 13.7, 9.3 ppm; HRMS (ESI) m/z calcd for $C_{13}H_{23}N_3O_2Na$ $(M+Na)^+$ 276.1688, found 276.1680.

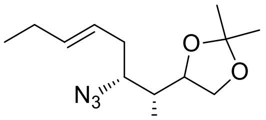

21c

Analytical data for **21c** and its enantiomer **21d**: ^{1}H NMR (300 MHz, $CDCl_3$) δ = 5.54 (dt, J = 15.3, 6.3 Hz, 1H), 5.37 (dt, J = 15.3, 6.3Hz, 1H), 3.89-3.97 (m, 1H), 3.52 (brs, 1H), 3.42-3.48 (m, 1H), 2.25-2.33 (m, 1H), 2.10-2.21 (m, 1H), 1.90-2.02 (m, 2H), 1.81-1.87 (m, 1H), 1.28 (s, 3H), 1.23 (s, 3H), 0.90 (t, J = 7.5 Hz, 3H), 0.78 (d, J = 7.2 Hz, 3H); ^{13}C NMR (75 MHz, CD_3OD) δ = 136.7, 126.3, 110.1, 77.9, 68.9, 66.5, 41.8, 35.1, 27.0, 26.7, 25.9, 14.0, 11.9 ppm.

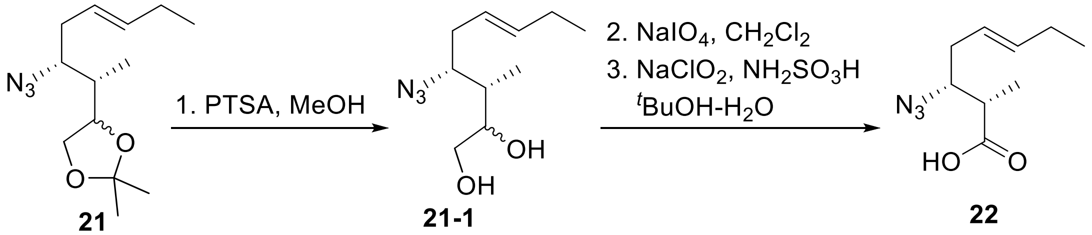

21 **21-1** **22**

To a solution of 2**1** (0.55 g, 2.16 mmol) in MeOH (10 mL), PTSA (74 mg, 0.39 mmol) was added in one portion at room temperature. The reaction solution was heated to reflux for 6 h, it was then quenched by addition of a saturated aqueous solution of $NaHCO_3$ (10 mL) at 0 ℃. Volatiles were removed in *vacuo*. The aqueous residue was extracted with CH_2Cl_2 (60 mL). The combined organic layers were washed with water (10 mL) and brine (10 mL), dried with Na_2SO_4 and concentrated in *vacuo*. The residue was purified by flash chromatography on silica gel (EtOAc/Hexane: 1/2) to give **21-1** (0.35 g, 75%, 98% brsm) as a colorless oil.

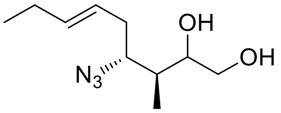

21-1a

Analytical data for **21-1a** and its enantiomer **21-1b**: ^{1}H NMR (500 MHz, $CDCl_3$) δ = 5.56 (dt, J = 15.2, 6.0 Hz, 1H), 5.33 (dt, J = 15.2, 6.0 Hz, 1H), 3.80-3.84 (m, 1H), 3.72 (dd, J = 11.0, 2.7 Hz, 1H), 3.53-3.57 (m, 1H), 3.45 (dd, J = 11.0, 6.9 Hz, 1H), 2.55 (brs, 1H), 2.31-2.36 (m, 1H), 2.11-2.16 (m, 1H), 1.95-1.98 (m, 2H), 1.89 (brs, 1H), 1.66-1.69 (m, 1H), 0.91 (t, J = 7.4 Hz, 3H), 0.77 (d, J = 7.0 Hz, 3H); ^{13}C NMR (75 MHz, $CDCl_3$) δ = 136.1, 124.3, 73.5, 65.0, 62.5, 38.8, 35.5, 25.6, 13.6, 9.8 ppm; HRMS (ESI) m/z calcd for

$C_{10}H_{19}N_3O_2Na$ $(M+Na)^+$ 236.1375, found 236.1376.

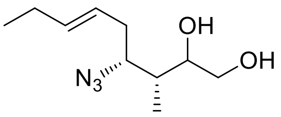

21-1c

Analytical data for **21-1c** and its enantiomer **21-1d**: ^{1}H NMR (300 MHz, $CDCl_3$) δ = 5.64 (dt, *J* = 15.3, 6.3 Hz, 1H), 5.44 (dt, *J* = 15.3, 6.3 Hz, 1H), 3.74 (dd, *J* = 10.8, 2.7 Hz, 1H), 3.57-3.67 (m, 2H), 3.52 (dd, *J* = 10.8, 7.5 Hz, 1H), 3.23 (br, 1H), 2.86 (br, 1H), 2.32-2.44 (m, 1H), 2.19-2.26 (m, 1H), 2.04 (m, 2H), 1.88 (m, 1H), 0.99 (t, *J* = 7.5 Hz, 3H), 0.89 (d, *J* = 6.9 Hz, 3H); ^{13}C NMR (125 MHz, $CDCl_3$) δ = 136.2, 124.5, 73.7, 65.0, 64.8, 39.8, 34.4, 25.8, 13.6, 12.2 ppm.

To a suspension of silica gel supported $NaIO_4$ (2.80 g, 2.80 mmol) in CH_2Cl_2 (7 mL), a solution of diol **21-1** (0.30 g, 1.4 mmol) in CH_2Cl_2 (7mL) was added at room temperature. 20 min later, the reaction mixture was filtered off through a short pad of silica gel and eluted with CH_2Cl_2 (50 mL). The filtrate was concentrated to give rise to the corresponding aldehyde as a colorless oil, which was used directly in the next step.

To a cold solution of aldehyde in ^{t}BuOH (10 mL), a solution of $NaClO_2$ (0.43 g, 4.70 mmol) and sulfamic acid (0.46 g, 4.70 mmol) in an aqueous solution of KH_2PO_4 (5 mL, 1 M) was added slowly. The resulting yellow solution was stirred at 0 ℃ for 0.5 h and room temperature for additional 3 h. It was then diluted with brine (5 mL) and ^{t}BuOH was removed in *vacuo*. The residue was partitioned between EtOAc (50 mL) and water (30 mL). Layers were separated, and the aqueous layer was extracted with EtOAc (30 mL x 2). The combined organic layers were washed with water (20 mL), brine (20 mL), dried over Na_2SO_4 and concentrated in *vacuo*. The residue was purified by flash chromatography on silica gel (EtOAc/Hexane: 1/6) to afford acid **22** (0.21 g, 79%) as a light yellow oil.

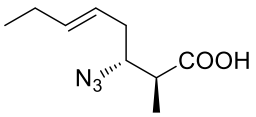

22a

Analytical data for **22a**: $[\alpha]_D^{20}$ = –33.4 (*c* = 0.9, MeOH); ^{1}H NMR (500 MHz, $CDCl_3$) δ = 5.66 (dt, *J* = 15.3, 6.0 Hz, 1H), 5.41 (dt, *J* = 15.3, 6.0 Hz, 1H), 3.78-3.82 (m, 1H), 2.59-2.64 (m, 1H), 2.37 (ddd, *J* = 7.1 Hz, 1H), 2.30 (m, 1H), 2.02-2.08 (m, 2H), 1.24 (d, *J* = 7.1 Hz, 3H), 1.00 (t, *J* = 7.5 Hz, 3H); ^{13}C NMR (125 MHz, $CDCl_3$) δ = 178.1, 136.9, 123.3, 63.4, 42.9, 35.8, 25.6, 13.4, 11.7 ppm; HRMS (ESI) m/z calcd for $C_9H_{14}N_3O_2Na_2$ $(M-H+Na\times2)^+$ 242.0882, found 242.0868.

22b is the enantiomer of compound **22a**.

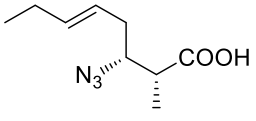

22c

Analytical data for **22c**: $[\alpha]_D^{20}$ = –38.1 (*c* = 0.47, MeOH); ^{1}H NMR (500 MHz, $CDCl_3$) δ = 5.67 (dt, *J* = 15.2, 6.3 Hz, 1H), 5.43 (dt, *J* = 15.2, 6.3 Hz, 1H), 3.63 (dt, *J* = 11.1, 4.2 Hz, 1H), 2.64 (m, 1H), 2.40-2.47 (m, 1H), 2.28 (m, 1H), 2.06 (m, 2H), 1.23 (d, *J* = 10.7 Hz, 3H), 1.00 (t, *J* = 7.5 Hz, 3H); ^{13}C NMR (75 MHz, $CDCl_3$) δ = 180.2, 137.0, 123.1, 64.0, 43.5, 34.6, 25.7, 13.6, 13.5 ppm.

22d is the enantiomer of compound **22c**.

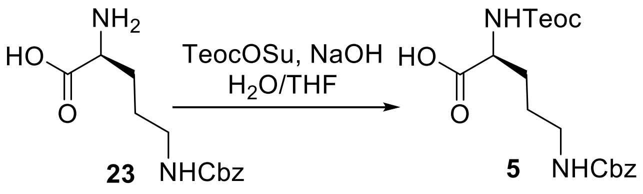

To a solution of *L*-Orn·HCl (50.4 g, 300 mmol) in a solution of NaOH (24.0 g, 600 mmol) in water (250 mL), $CuSO_4$ (23.9 g, 150 mmol, in 100 mL of water) was slowly added. The resulting suspension was stirred for 5 hours and then cooled to 0 ℃. To this solution, NaOH (14.4 g, 360 mmol), $NaHCO_3$ (25.2 g, 300 mmol) and CbzCl (51 mL, 360 mmol) were added successively. The reaction mixture was then allowed to warm to room temperature and stirred for additional 6 h. The precipitation was collected by filtration. The solid was washed with water (150 mL x 3), ethanol (150 mL x 2) and diethyl ether (100 mL x 2) to afford the desired complex (87.3 g, 98% crude yield), which was used in the next step of the reaction.

The copper complex (6.35 g, 10.7 mmol) was suspended in methanol (1 mL) and water (60 mL). To this solution, $NaBH_4$ (0.81 g, 21.4 mmol) was added in small portions, and the reaction mixture was kept for additional 2 h. The black precipitation was filtered off and washed with an aqueous solution of NaOH (5 ml, 3 N in water). The combined filtrates were cooled to 0 ℃, TeocOSu (7.80 g, 30.1 mmol) in THF (80 mL) was slowly added at 0 ℃. 10 h later, the volatiles were removed in *vacuo* and the aqueous solution was washed with diethyl ether (30 mL). The organic phase was discarded, the aqueous solution was cooled to 0 ℃ and acidified to pH 2 with dilute HCl (1 N) and extracted with ethyl acetate (80 mL x 2). The combined extracts were washed with brine (30 mL), dried over Na_2SO_4 and concentrated in *vacuo* to afford acid **5** (7.1 g, 81% yield) as a colorless oil. $[\alpha]_D^{20} = -0.86$ (c = 0.81, MeOH); ^{1}H NMR (300 MHz, $CDCl_3$) δ = 7.26-7.39 (m, 5H), 5.54 (d, J = 8.4 Hz, 1H), 5.20 (s, 1H), 5.08 (s, 2H), 4.30-4.40 (m, 1H), 4.16 (t, J = 4.8 Hz, 2H), 3.12-3.26 (m, 2H), 1.81-1.95 (m, 1H), 1.63-1.77 (m, 1H), 1.51-1.63 (m, 2H), 0.98 (t, J = 4.9 Hz, 2H), 0.03 (s, 9H); ^{13}C NMR (125 MHz, $CDCl_3$) δ = 175.5, 157.0, 156.8, 136.6, 128.6, 128.2, 128.1, 67.0, 63.8, 53.5, 40.6, 29.7, 25.9, 17.9, –1.4 ppm; HRMS (ESI) m/z calcd for $C_{19}H_{30}N_2O_6SiNa$ $(M+Na)^+$ 433.1771, found 433.1763.

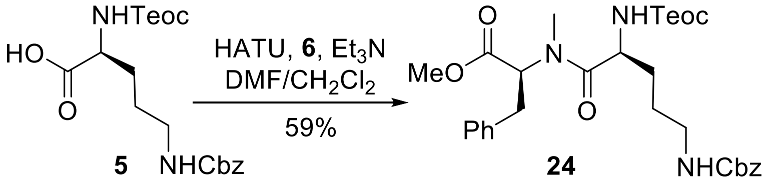

To a solution of acid **5** (2.70 g, 6.59 mmol) and amine **6** (1.60 g, 8.33 mmol) in CH_2Cl_2 (70 mL) and DMF (7 mL), HATU (3.80 g, 10.0 mmol) and Et_3N (2.4 mL, 17.2 mmol) were successively added at 0 ℃. The reaction mixture was allowed to stir at room temperature for 16 h and concentrated in *vacuo*. The residue was partitioned between ethyl ether (100 mL) and saturated aqueous solution of NH_4Cl (30 mL). Layers were separated, the organic phase was washed with water (20 mL) and brine (15 mL), dried over Na_2SO_4 and concentrated in *vacuo*. The residue was purified by flash chromatography on silica gel ((EtOAc/Hexane: 1/2) to offer dipeptide **24** (2.27 g, 59%) as a white foam like solid. $[\alpha]_D^{20} = -42.2$ (c = 0.36, MeOH); ^{1}H NMR (500 MHz, $CDCl_3$) δ = 7.28-7.37 (m, 5H), 7.16-7.25 (m, 5H), 5.30 (d, J = 7.8 Hz, 1H), 5.24 (s, 1H), 5.09 (s, 2H), 4.95 (s, 1H), 4.51-4.59 (m, 1H), 4.11 (t, J = 8.3 Hz, 2H), 3.74 (s) and 3.71 (s, 3H, RI), 3.38 (dd, J = 14.8, 5.3 Hz, 1H), 3.20 (d, J = 5.3 Hz, 1H), 3.02 (dd, J = 14.7, 10.7 Hz, 1H), 2.96 (dd, J = 6.5 Hz, 1.5Hz, 1H), 2.92(s) and 2.87(s,

3H), 1.77(m, 1H), 1.69 (m, 1H), 1.51 (m, 2H), 0.96 (t, *J* = 9.5 Hz, 2H), 0.04 (s) and 0.03 (s, 9H); ^{13}C NMR (125 MHz, $CDCl_3$) δ = 172.7, 171.2, 156.7, 156.6, 137.0, 137.0, 129.5, 129.3, 129.1, 128.9 and 128.8 (RI), 128.4, 127.2, 66.9, 65.0, 63.6, 61.9 and 59.1 (RI), 52.6, 50.7, 41.1 and 39.8 (major, RI), 34.8 (major) and 33.2 (RI), 30.5 (major) and 30.2 (RI), 25.7, 18.1, –1.1 ppm; HRMS (ESI) m/z calcd for $C_{30}H_{44}N_3O_7Si$ $(M+H)^+$ 586.2948, found 586.2920.

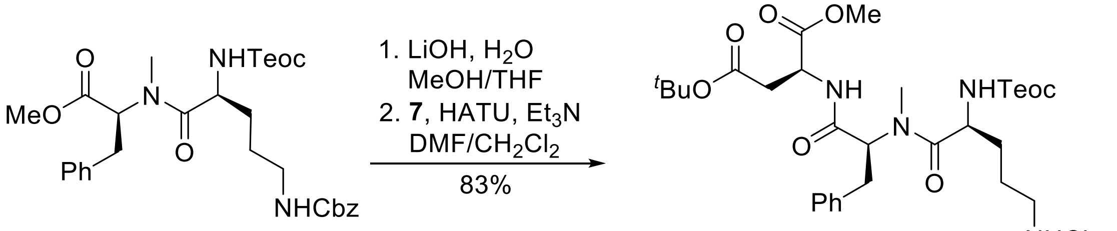

24 **25**

To a solution of dipeptide **24** (6.0 g, 10.3 mmol) in MeOH/THF (80 mL, v:v = 1:1), aqueous solution of LiOH (20 mL, 0.65 M in water) was added at 0 ℃. The reaction mixture was stirred at room temperature for 4 h. The volatiles were removed in *vacuo*. The aqueous layer was washed with diethyl ether (50 mL), and the organic phase was discarded. The pH value of the aqueous phase was adjusted to 3 by addition of an aqueous solution of citric acid (10% in water) at 0 ℃ and then extracted with EtOAc (60 mL x 3). The combined organic layers were washed with brine (50 mL), dried over Na_2SO_4 and concentrated in *vacuo* to give the corresponding acid as a colorless paste, which was used directly in the next step.

To a solution of the above acid and amine **7** (3.6 g, 12.0 mmol) in CH_2Cl_2 (100 mL) and DMF (10 mL), HATU (4.30 g, 11.3 mmol) and DIPEA (3.80 mL, 21.8 mmol) were successively added at 0 ℃. The resulting yellow solution was then stirred at room temperature for 16 h. Volatiles were removed in *vacuo*, the residue was dissolved in diethyl ether (120 mL) and washed with a saturated aqueous solution of NH_4Cl (40 mL), water (30 mL) and brine (30 mL). The organic phase was dried over Na_2SO_4 and concentrated. The residue was purified by flash chromatography on silica gel (EtOAc/Hexane: 1/2) to afford tripeptide **25** (6.50 g, 83%) as a foamy solid. $[\alpha]_D^{20}$ = –55.0 (*c* = 1.03, MeOH); ^{1}H NMR (300 MHz, $CDCl_3$) δ = 7.30-7.48 (m, 5H), 7.12-7.23 (m, 5H), 5.42 (dd, *J* = 11.7, 6.6Hz) and 4.94-4.99 (m, 1H, RI), 5.22-5.27 (m, 1H, RI), 5.10 (s) and 5.07 (s, 2H, RI), 4.81-4.88 (m, 1H) and 4.51-4.56 (m, 1H, RI), 4.63-4.72 (m, 1H), 4.17-4.24 (m) and 3.39 (dd, *J* = 14.7, 6.6 Hz, RI), 4.07-4.14 (m, 2H), 3.76 (s) and 3.66 (s, 3H, RI), 3.34-3.42 (m) and 3.02-3.11 (m, overlp, 2H), 3.15-3.21 (m, 2H), 2.93 (s) and 2.89 (s, 3H, RI), 2.76-2.84 (m, 2H), 1.55-1.61 (m, 2H), 1.40 (s, 9H), 1.22-1.30 (m) and 0.83-0.91 (m, 2H, RI), 0.93-0.99 (m, 2H), 0.04 (s) and 0.03 (s, 9H); ^{13}C NMR (75 MHz, $CDCl_3$) δ = 173.1 and 173.0 (RI), 171.2 and 171.2 (RI), 170.1 and 169.5 (RI), 169.2 and 169.0 (RI), 157.6, 156.4 and 156.3 (RI), 138.1, 136.8 and 136.6 (RI), 129.6, 129.1 and 129.0 (RI), 128.7 and 128.6 (RI), 128.3 and 128.2 (RI), 127.1, 126.9, 82.1 and 81.6 (RI), 66.8 and 66.7 (RI), 64.3 and 63.5 (RI), 62.2, 52.9 and 52.6 (RI), 50.4 and 49.6 (RI), 49.1, 40.9 and 40.5 (RI), 37.4, 34.4 and 33.8 (RI), 31.7 and 30.3 (RI), 29.3, 28.1, 26.6 and 25.5 (RI), 17.8 and 17.7 (RI), –1.3 and –1.4 (RI) ppm; HRMS (ESI) m/z calcd for $C_{38}H_{57}N_4O_{10}Si$ $(M+H)^+$ 757.3844, found 757.3834.

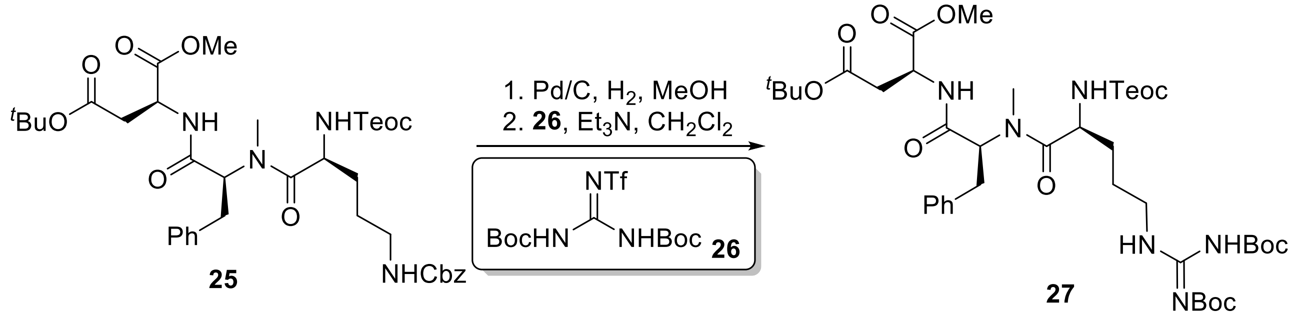

To a solution of tripeptide **25** (5.5 g, 7.28 mmol) in MeOH (25 mL), Pd/C (0.72 g, 10% on charcoal) was added. The reaction vessel was sealed and exposed to H_2 for 2 h. Pd/C was removed by filtration and eluted with MeOH (25 mL). The filtrate was concentrated to give the desired amine as a colorless paste, which was used directly in the next step.

To a solution of the above amine and Et_3N (1.23 mL, 8.82 mmol) in CH_2Cl_2 (60 mL), compound **26** (3.17 g, 8.00 mmol) was added at room temperature and the reaction mixture was stirred for 5 h. The mixture was then diluted with CH_2Cl_2 (100 mL) and washed with an aqueous solution of $NaHSO_4$ (60 mL, 2 N), brine (30 mL). The organic layer was dried over Na_2SO_4 and concentrated in *vacuo*. The residue was purified by flash chromatography on silica gel (EtOAc/Hexane: 1/2) to give tripeptide **27** (5.73 g, 91% yield) as a foamy solid. $[\alpha]_D^{20} = -54.0$ (c = 1.34, MeOH); ^{1}H NMR (500 MHz, $CDCl_3$) δ = 8.19 (s) and 8.30 (s, 1H, RI), 8.06 (d, J = 8.5Hz, 1H), 7.34 (m, 2H), 7.25 (m, 1H), 7.11 (d, J = 7.7 Hz, 1H), 7.07 (d, J = 7.6 Hz, 1H), 5.26 (d, J = 8.5 Hz, 1H), 5.17 (d, J = 8.3 Hz) and 4.99 (dd, J = 11.2, 3.1 Hz, 1H, RI), 4.84 (m, 1H), 4.51 (m) and 4.72 (m, 1H, RI), 4.22-4.24(m) and 3.97-4.03 (m, 1H, RI), 4.01-4.07 (m, 2H), 3.71 (s) and 3.75 (s, 3H, RI), 3.34-3.42 (m, 1H), 3.18 (m) and 3.22-3.30 (m, 1H, RI), 2.98-3.09 (m, 2H), 2.89 (s) and 2.96 (s, 3H, RI), 2.72-2.82 (m, 2H, RI), 1.68-1.80 (m, 1H), 1.54-1.65 (m, 1H), 1.48- 1.52 (m, 2H), 1.50 (s) and 1.51 (s, 9H, RI), 1.49 (s, 9H), 1.40 (s) and 1.42 (s, 9H, RI), 0.95-0.99 (m, 2H), 0.02 (s) and 0.03 (s, 9H, RI); ^{13}C NMR (125 MHz, $CDCl_3$) δ = 172.9, 171.0 and 170.8, 169.7 and 169.4, 169.1 and 168.9, 163.6, n 157.5, 156.1 and 156.0 (RI), 153.3 and 153.2 (RI), 137.6, 129.4 and 129.1 (RI), 128.8 and 128.5 (RI), 126.7, 83.1 and 83.0 (RI), 81.7 and 81.4 (RI), 79.2 and 79.1 (RI), 64.1, 63.3 and 62.2 (RI), 52.6 and 52.4 (RI), 50.6 and 49.9 (RI), 49.5 and 49.0 (RI), 40.3 and 40.1 (RI), 37.3, 34.3 and 33.8 (RI), 29.9 and 29.0 (RI), 28.3, 28.2, 28.1, 28.0, 26.1 and 25.1 (RI), 17.7 and 17.6 (RI), –1.5 and –1.6 (RI) ppm; HRMS (ESI) m/z calcd for $C_{41}H_{69}N_6O_{12}Si$ $(M+H)^+$ 865.4742, found 865.4731.

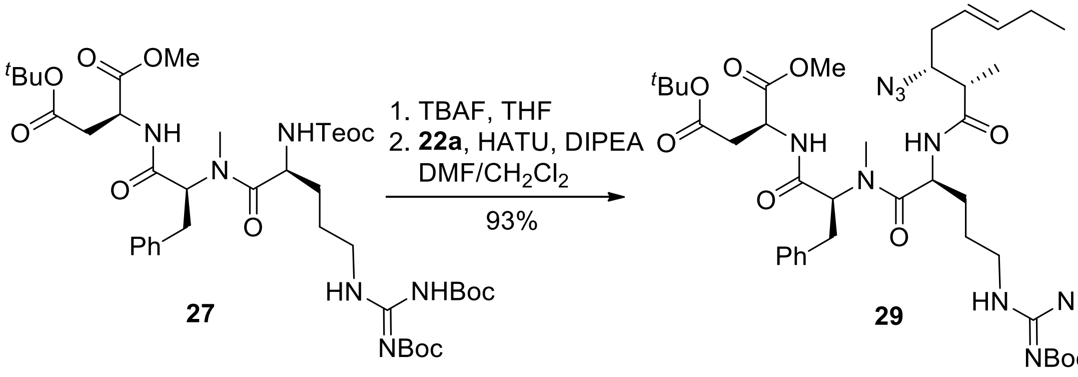

To a solution of tripeptide **27** (156 mg, 0.18 mmol) in THF (5 mL), TBAF (0.75 mL, 1.0 M in THF) was added at room temperature. After being stirred for 16 h, the reaction mixture was concentrated

and purified with a short flash chromatography on silica gel ($MeOH/CH_2Cl_2$: 1/20) to produce the desired amine, which was used directly in the next step.

To a solution of the above amine and acid **22** (20 mg, 0.1 mmol) in CH_2Cl_2 (2.0 mL) and DMF (0.2 mL) HATU (63 mg, 0.17 mmol) and collidine (40 μL, 0.3 mmol) were successively added at 0 ℃. The reaction mixture was stirred at room temperature for 16 h and then poured into diethyl ether (50 mL). the organic layer was washed with saturated aqueous solution of NH_4Cl (15 mL), water (10 mL) and brine (15 mL), dried over Na_2SO_4 and concentrated in *vacuo*. The residue was purified by flash chromatography on silica gel (EtOAc/Hexane: 1/2) to afford tetrapeptide **29** as a foamy solid (83 mg, 93%).

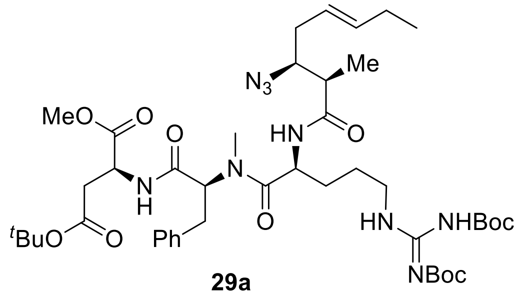

29a

Analytical data for **29a**: $[\alpha]_D^{20}$ = –42.2 (*c* = 0.60, MeOH); ^{1}H NMR (500 MHz, $CDCl_3$) δ = 11.47 (s, 1H), 8.31 (d, *J* = 7.5 Hz, 1H), 8.21-8.23 (m) and 6.91 (d, *J* = 7.5 Hz, 1H, RI), 6.47 (dd, *J* = 21.0, 7.0 Hz, 1H), 5.58-5.68 (m, 1H), 5.30-5.40 (m, 1H), 5.21 (t, *J* = 4.8 Hz) and 4.20 (t, *J* = 7.5 Hz, 1H, RI), 4.93 (dd, *J* =11.0, 2.5 Hz) and 4.71 (m, 1H, RI), 4.81 (m, 1H), 3.75 (s) and 3.72 (s, 3H, RI), 3.57-3.61 (m) and 3.35-3.36 (m, overlp, 1H, RI), 3.36-3.40 (m, 1H), 3.17-3.24 (m, 1H), 3.02-3.07 (m, 2H), 2.82-2.83 (m, 1H, RI), 2.93 (s) and 2.87 (s, 3H, RI), 2.69-2.76 (m, 1H), 2.37-2.40 (m) and 2.28-2.31 (m, 1H, RI), 2.14-2.24 (m, 2H), 2.00-2.05 (m, 2H), 1.83-1.88 (m) and 1.72-1.77 (m, 1H, RI), 1.57-1.62 (m, overlp, 1H), 1.53-1.60 (m, overlp, 2H), 1.50 (s, 9H), 1.48 (s, 9H), 1.42 (s) and 1.41 (s, 9H, RI), 1.19 (d, *J* = 7.1 Hz) and 1.17 (d, *J* = 7.1 Hz, 3H, RI), 0.99 (t, *J* = 7.5 Hz) and 0.97 (t, *J* = 7.5 Hz, 3H, RI); ^{13}C NMR (125 MHz, $CDCl_3$) δ = 174.9, 173.1 and 172.3 (RI), 171.0 and 170.8 (RI), 170.0, 169.2 and 168.9 (RI), 163.6 and 163.5 (RI), 156.2, 153.3 and 153.2 (RI), 137.8, 136.6 and 136.5 (RI), 129.4 and 129.1 (RI), 128.8 and 128.5 (RI), 127.2 and 126.7 (RI), 123.8 and 123.7 (RI), 83.2 and 83.0 (RI), 81.7 and 81.2 (RI), 79.2 and 79.1 (RI), 64.4 and 63.9 (RI), 62.5, 52.6 and 52.4 (RI), 49.7, 49.0 and 48.8 (RI), 44.8 and 43.7 (RI), 40.3 and 40.0 (RI), 37.3 and 37.2 (RI), 35.9 and 35.7 (RI), 34.2 and 33.8 (RI), 29.6 and 29.5 (RI), 29.1 (RI), 28.3, 28.1, 28.0, 26.1, 25.6 and 25.0 (RI), 13.8 and 13.0 (RI), 13.5 ppm; HRMS (ESI) m/z calcd for $C_{44}H_{70}N_9O_{11}$ $(M+H)^+$ 900.5195, found 900.5166.

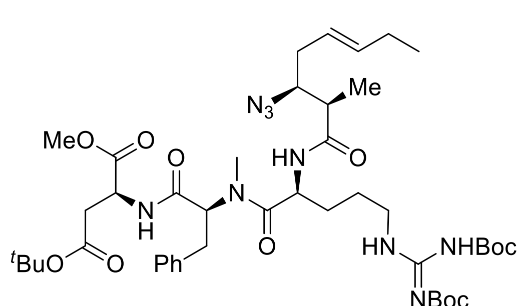

29b

Analytical data for **29b**: $[\alpha]_D^{20}$ = –47.4 (*c* = 0.57, MeOH); ^{1}H NMR (500 MHz, $CDCl_3$) δ = 11.50 (s) and 11.49 (s, 1H, RI), 8.30-8.32 (m) and 8.24-8.26 (m, overlp, 1H, RI), 8.23 (d, *J* = 8.0 Hz) and 6.93 (d, *J* = 8.0 Hz, 1H, RI), 7.31-7.36 (m, 2H), 7.25-7.27 (m, 1H), 7.15-7.21 (m, 2H), 6.77 (d, *J* = 7.0 Hz) and 6.41 (d, *J* = 8.0 Hz, 1H, RI), 5.56-5.64 (m, 1H), 5.36-5.43 (m, 1H), 5.17-5.20 (m) and 4.22 (dt, *J* = 14.0, 3.0 Hz, 1H, RI), 4.96 (dd, *J* = 11.5, 3.5 Hz) and 4.85 (dd, *J* = 13.0, 7.0 Hz, 1H, RI), 4.80-4.82 (m) and 4.73 (ddd, *J* = 9.0, 4.5, 4.5 Hz, 1H, RI), 3.77 (s) and 3.73 (s, 3H, RI), 3.54-3.61 (m, 1H), 3.40-3.44 (m, 1H), 3.36-3.39 (m) and 3.20 (dd, *J* = 14.0, 3.0 Hz, 1H, RI), 3.26-3.33 (m) and 3.01-3.08 (m, overlp, 1H), 3.02-3.09 (m, 1H), 2.93 (s) and 2.88 (s, 3H, RI), 2.82-2.85 (m, 1H), 2.73-2.78 (m, 1H), 2.41-2.46 (m) and 2.27-2.33 9 (m, overlp, 1H, RI), 2.27-2.33 (m, overlp, 1H), 2.15-2.23 (m, 1H), 2.00-2.07 (m, 2H), 1.75-1.79 (m) and 1.60-1.64 (m, overlp, 1H), 1.56-1.60 (m, 2H), 1.51 (s) and 1.50 (s, 9H, RI), 1.49 (s, 9H), 1.43 (s) and 1.42 (s, 9H, RI), 1.16 (d, *J* = 7.0 Hz)

and 1.15 (d, J = 7.0 Hz, 3H, RI), 1.07-1.12 (m) and 0.03-0.08 (m, 1H, RI), 0.99 (t, J = 7.5 Hz) and 0.97 (t, J = 7.5 Hz, 3H, RI); ^{13}C NMR (75 MHz, $CDCl_3$) δ = 174.9, 173.2 and 172.4 (RI), 171.3 and 171.0 (RI), 170.2, 169.4 and 169.1 (RI), 163.7 and 163.6 (RI), 156.5 and 156.3 (RI), 153.4 and 153.3 (RI), 137.9, 136.9 and 136.7 (RI), 129.5 and 129.2 (RI), 129.0 and 128.7 (RI), 127.3 and 126.9 (RI), 123.7 and 123.6 (RI), 83.4 and 83.2 (RI), 81.9 and 81.5 (RI), 79.5 and 79.4 (RI), 64.7 and 64.6 (RI), 62.5, 52.8 and 52.7 (RI), 49.6 and 49.3 (RI), 49.0, 44.9 and 44.2 (RI), 40.4 and 40.0 (RI), 37.4 and 37.3 (RI), 36.0 and 35.8 (RI), 34.3 and 33.9 (RI), 30.1 and 29.9 (RI), 29.2, 28.4, 28.2, 28.1, 27.5 and 25.5 (RI), 26.1, 14.1 and 13.7 (RI), 13.6 ppm.

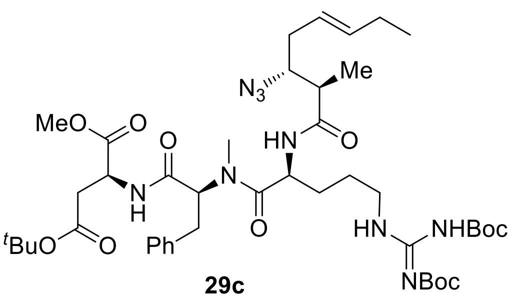

29c

Analytical data for **29c**: $[\alpha]_D^{20}$ = –56.6 (c = 0.64, MeOH); ^{1}H NMR (500 MHz, $CDCl_3$) δ = 11.49 (s) and 11.46 (s, 1H), 8.30 (d, J = 7.1 Hz, 1H), 8.23-8.25 (m) and 6.41 (d, J = 8.5 Hz, 1H, RI), 7.29-7.33 (m, 2H), 7.22-7.26 (m, 1H), 7.14-7.18 (m, 2H), 6.91 (dd, J = 19.5, 7.8 Hz, 1H), 5.59-5.65 (m, 1H), 5.37-5.42 (m, 1H), 5.17 (t, J = 7.0 Hz) and 4.25 (t, J = 7.0 Hz, 1H, RI), 4.94-4.97 (m) and 4.70-4.73 (m, 1H, RI), 4.81-4.85 (m, 1H), 3.75 (s) and 3.71 (s, 3H, RI), 3.51-3.59 (m, 1H), 3.34-3.45 (m, 2H), 3.19 (dd, J = 14.0, 2.0 Hz) and 2.75 (dd, J = 14.0, 4.5 Hz, 1H, RI), 3.00-3.09 (m, 2H), 2.65-2.72 (m, 1H), 2.92 (s) and 2.86 (s, 3H, RI), 2.37-2.41 (m, 1H), 2.31-2.35 (m) and 2.21-2.25 (m, 1H, RI), 2.13-2.19 (m, 1H), 1.99-2.07 (m, 2H), 1.72-1.76 (m) and 1.58-1.62 (m, overlp, 1H, RI), 1.58-1.64 (m, 1H), 1.49 (s, 9H), 1.47 (s, 9H), 1.42 (s) and 1.41 (s, 9H, RI), 1.12-1.17 (m) and 0.00-0.06 (m, 2H, RI), 1.08 (d, J = 6.0 Hz, 3H), 0.97 (t, J = 7.5 Hz, 3H); ^{13}C NMR (125 MHz, $CDCl_3$) δ = 175.1, 173.3 and 172.4 (RI), 171.1 and 170.8 (RI), 169.9, 169.1 and 169.0 (RI), 163.52 and 163.46 (RI), 156.3 and156.2 (RI), 153.2 and 153.1 (RI), 137.7, 136.9 and 136.5 (RI), 129.4 and 129.0 (RI), 128.8 and 128.5 (RI), 127.1 and 126.7 (RI), 123.1, 83.1 and 82.9 (RI), 81.7 and 81.2 (RI), 79.1 and 79.0, 64.7, 62.3, 52.5 and 52.4 (RI), 49.5 and 49.3 (RI), 48.9 and 48.8 (RI), 45.0 and 44.2 (RI), 40.3 and 39.8 (RI), 37.3 and 37.2 (RI), 34.7, 34.2 and 33.7 (RI), 29.6 and 29.4 (RI), 29.0, 28.29 and 28.27 (RI), 28.02, 27.99, 26.9 and 26.8 (RI), 25.6 and 25.1 (RI), 14.6 and 14.3 (RI), 13.5 ppm.

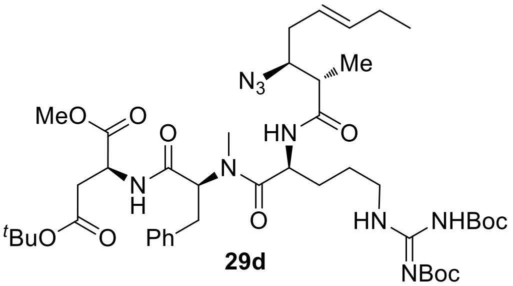

29d

Analytical data for **29d**: $[\alpha]_D^{20}$ = –43.9 (c = 0.36, MeOH); ^{1}H NMR (500 MHz, $CDCl_3$) δ = 11.46 (s, 1H), 8.30 (brs) and 8.21 (brs, 1H, RI), 8.15 (d, J = 7.5 Hz) and 6.90 (d, J = 8.0 Hz, 1H, RI), 7.31-7.34 (m, 2H), 7.25-7.27 (m, 1H), 7.14-7.19 (m, 2H), 6.44 (d, J = 6.3 Hz) and 6.40 (d, J = 6.3 Hz, 1H, RI), 5.59-5.65 (m, 1H), 5.36-5.44 (m, 1H), 5.12-5.15 (m) and 4.19-4.22 (m, 1H, RI), 4.93 (dd, J = 11.0, 3.0 Hz) and 4.71-4.75 (m, 1H), 4.81-4.84 (m, 1H), 3.75 (s) and 3.71 (s, 3H, RI), 3.56-3.60 (m, 1H), 3.36-3.42 (m, 1H), 3.19-3.24 (m, 1H), 3.01-3.07 (m, 2H), 2.92 (s) and 2.87 (s, 3H, RI), 2.81-2.84 (m, 1H), 2.70-2.76 (m, 1H), 2.37-2.41 (m, 1H), 2.27-2.34 (m, 1H), 2.13-2.21 (m, 1H), 1.99-2.07 (m, 2H), 1.83-1.88 (m) and 1.76-1.79 (m, 1H, RI), 1.58 (brs, 2H), 1.50 (s, 9H), 1.48 (s, 9H), 1.42 (s, 9H), 1.13 (d, J = 7.0 Hz) and 1.12 (d, J = 7.0 Hz, 3H, RI), 0.96-1.00 (m, 3H), 1.06-1.08 (m) and 0.10-0.15 (m, 1H, RI); ^{13}C NMR (125 MHz, $CDCl_3$) δ = 175.2, 173.5, 172.5 and 172.4 (RI), 171.5 and 171.1 (RI), 169.6 and 169.2 (RI), 163.91 and 163.88 (RI), 156.5, 153.64 and 153.56 (RI), 138.1 and 137.3 (RI), 136.9, 129.7 and 129.4 (RI), 129.3 and 128.9 (RI), 127.5 and 127.1 (RI), 123.8 and 123.6 (RI), 83.5 and 83.3 (RI), 82.1 and 81.6, 79.5 and 79.4 (RI), 65.0 and 64.9 (RI), 62.8, 52.9 and 52.7 (RI), 50.0, 49.3 and 49.2 (RI), 45.2 and 44.6 (RI), 40.7 and 40.4 (RI), 37.7 and 37.6 (RI), 35.2, 34.6 and 34.2

(RI), 29.9, 29.6, 28.6, 28.4, 28.3, 26.5 and 25.9 (RI), 25.4, 15.2 and 14.7 (RI), 13.8 ppm.

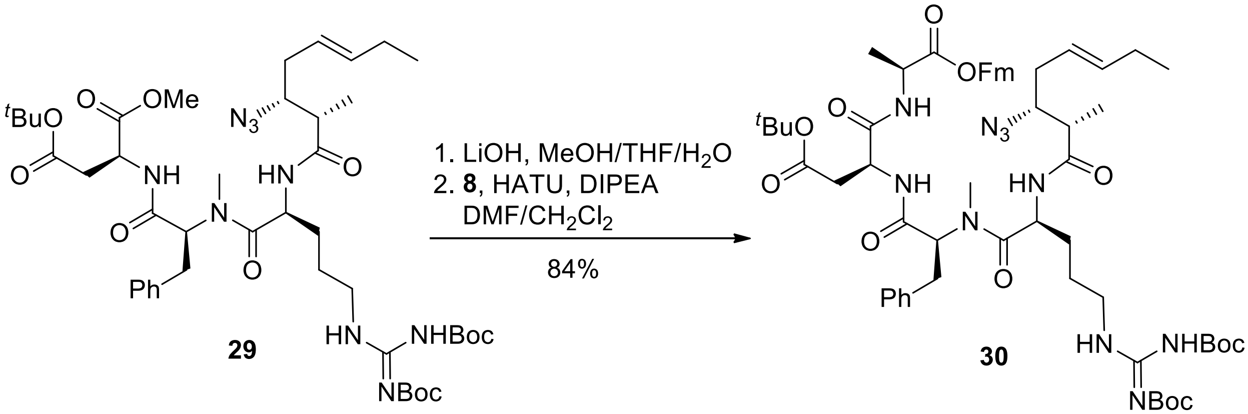

To a solution of tetrapeptide **29** (110 mg, 0.12 mmol) in MeOH/THF (1.2 mL, v:v = 1:1), LiOH (10 mg, 0.24 mmol, in 0.3 mL of water) was slowly added at 0 ℃. After being stirred for 2.5 h, the pH of the solution was adjusted to 3 by the addition of a aqueous solution of citric acid (10% in water). Volatiles were removed under reduced pressure, the residue was partitioned between EtOAc (30 mL) and water (10 mL). Layers were separated, the aqueous layer was extracted with EtOAc (30 mL). The combined organic layers were washed with brine (10 mL), dried over Na_2SO_4 and concentrated to give the corresponding acid as a colorless paste, which was used directly in the next step.

To a solution of the above acid and amine **8** in CH_2Cl_2 (2 mL) and DMF (0.2 mL), HATU (60 mg, 0.16 mmol) and DIPEA (130 μL, 0.75 mmol) were added at 0 ℃. The reaction mixture was stirred at room temperature for 8 h and then poured into diethyl ether (100 mL) and washed with saturated solution of NH_4Cl (20 mL), water (15 mL) and brine (15 mL). The organic phase was dried over Na_2SO_4 and concentrated in *vacuo*. The residue was purified by flash chromatography on silica gel (EtOAc/Hexane: 1/2) to give pentapeptide **30** (110 mg, 82%) as a foamy solid.

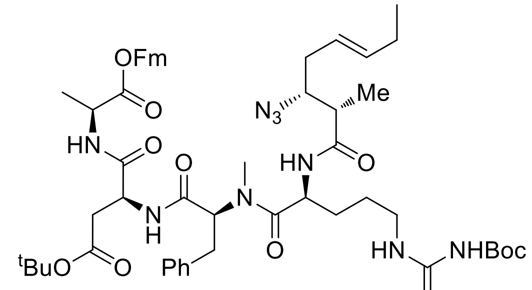

30a

Analytical data for **30a**: $[\alpha]_D^{20}$ = –35.7 (*c* = 0.30, MeOH); ^{1}H NMR (500 MHz, $CDCl_3$) δ = 11.49 (s) and 11.47 (s, RI), 8.31-8.33 (m) and 8.22-8.24 (m, 1H, RI), 8.17 (d, *J* = 7.0 Hz) and 7.4 (m, overlp, 1H, RI), 7.76-7.79 (m, 2H), 7.61-7.64 (m, 1H), 7.57 (d, *J* = 7.5 Hz, 1H), 7.39-7.42 (m, 2H), 7.30-7.36 (m, 5H), 7.15-7.22 (m, 2H), 7.06-7.07 (m, 1H), 6.79 (d, *J* = 8.0 Hz) and 6.63 (d, *J* = 7.0 Hz, 1H, RI), 5.63-5.69 (m) and 5.53-5.61 (m, 1H, RI), 5.34-5.39 (m, 1H), 4.85-4.93 (m, 1H), 4.79-4.82 (m) and 4.15-4.18 (m, 1H, RI), 4.62-4.68 (m, 1H), 4.69-4.72 (m) and 4.63-4.66 (m, overlp, 1H, RI), 4.39-4.51 (m, 2H), 4.23 (t, *J* = 7.0 Hz, 1H), 3.65-3.69 (m) and 3.58-3.61 (m, 1H, RI), 3.36-3.40 (m, 1H), 3.25-3.34 (m, 1H), 3.12-3.20 (m, 1H), 3.00-3.05 (m, 1H), 2.96 (dd, *J* = 17.5, 7.0 Hz) and 2.57 (dd, *J* = 17.0, 6.0 Hz, 1H, RI), 2.89 (s) and 2.87 (s, 3H, RI), 2.69-2.80 (m, 1H), 2.36-2.45 (m, 1H), 2.31-2.36 (m) and 2.22-2.25 (m, 1H, RI), 2.15-2.20 (m, 1H), 2.00-2.04 (m, 2H), 1.84-1.89 (m) and 1.70-1.77 (m, 1H, RI), 1.56-1.61 (m, 1H), 1.59-1.62 (m) and 1.50-1.53 (m, 1H, RI), 1.06-1.11 (m) and -0.08-0.03 (m, 1H, RI), 1.50 (s, 9H), 1.48 (s, 9H), 1.45 (s) and 1.41 (s, 9H), 1.37 (t, *J* = 7.5 Hz, 3H), 1.17 (d, *J* = 7.0Hz, 3H), 0.96 (t, *J* = 7.5 Hz, 3H); ^{13}C NMR (125 MHz, $CDCl_3$) δ = 175.6, 173.8, 172.8 and 172.7 (RI), 170.5 and 170.4 (RI), 170.1, 169.7 and 169.6 (RI), 163.9 and 163.8 (RI), 156.5, 153.7, 153.6, 144.0 and

143.7 (RI), 141.7, 138.1, 137.2 and 136.8 (RI), 129.7 and 129.5 (RI), 129.3 and 129.0 (RI), 128.2, 127.5, 127.3, 125.4 and 125.3 (RI), 124.2 and 123.9 (RI), 120.4, 83.6 and 83.4 (RI), 82.2 and 81.8 (RI), 79.6 and 79.5 (RI), 67.5, 64.8 and 64.3 (RI), 63.2, 50.8, 49.9, 49.4 and 49.1 (RI), 48.8 and 48.6 (RI), 47.2 and 47.1 (RI), 45.0 and 44.2 (RI), 40.7 and 40.3 (RI), 37.5 and 37.0 (RI), 36.3 and 36.0 (RI), 34.5 and 34.2 (RI), 30.0 and 29.7 (RI), 28.7, 28.42, 28.39, 26.5 and 25.5 (RI), 26.0, 18.4 and 18.3 (RI), 14.2 and 13.2 (RI), 13.8 ppm; HRMS (ESI) m/z calcd for $C_{60}H_{83}N_{10}O_{12}$ $(M+H)^+$ 1135.6192, found 1135.6172.

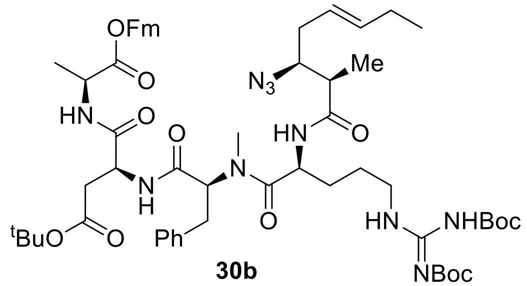

30b

Analytical data for **30b**: $[\alpha]_D^{20}$ = –45.2 (*c* = 0.60, MeOH); ^{1}H NMR (500 MHz, $CDCl_3$) δ = 11.50 (s, 1H), 8.32-8.34 (m) and 8.25-8.27 (m, 1H, RI), 8.14 (d, *J* = 8.1 Hz) and 7.40-7.42 (overlp, 1H, RI), 7.76-7.79 (m, 2H), 7.57-7.63 (m, 2H), 7.39-7.43 (m, 2H), 7.28-7.36 (m, 5H), 7.22 (d, *J* = 7.3 Hz) and 6.97 (d, *J* = 7.3 Hz, 1H, RI), 7.17 (d, *J* = 7.3 Hz, 1H), 7.08 (d, *J* = 7.5 Hz, 1H), 5.54-5.63 (m, 1H), 5.32-5.40 (m, 1H), 4.92-4.96 (m) and 4.84-4.89 (m, 1H), 4.76-4.80 (m) and 4.67-4.70 (m, 1H, RI), 4.62-4.67 (m, 1H), 4.55-4.60 (m) and 4.16-4.20 (m, overlp, 1H, RI), 4.40-4.48 (m, 2H), 4.22-4.25 (m, 1H), 3.54-3.60 (m, 1H), 3.40-3.42 (m, 1H), 3.26-3.33 (m, 1H), 3.18-3.26 (m, 1H), 2.94-3.03 (m, 2H), 2.88 (s) and 2.86 (s, 3H, RI), 2.70-2.82 (m, 1H), 2.58 (dd, *J* = 17.0, 4.5 Hz) and 2.16-2.22 (m, overlp, 1H, RI), 2.23-2.29 (m, 1H), 2.14-2.18 (m, overlp, 1H), 1.97-2.04 (m, 2H), 1.82-1.88 (m) and 1.55-1.60 (m, overlp, 1H, RI), 1.55-1.60 (m, 2H), 1.50 (s, 9H), 1.48 (s, 9H), 1.44 (s) and 1.41 (s, 9H), 1.38 (t, *J* = 7.6Hz, 3H), 1.16 (d, *J* = 7.3 Hz) and 1.14 (d, *J* = 7.3 Hz, 3H), 1.04-1.08 (m) and -0.4--0.8 (m, 1H, RI), 0.95 (t, *J* = 7.5 Hz) and 0.94 (t, *J* = 7.5 Hz, 3H, RI), ; ^{13}C NMR (125 MHz, $CDCl_3$) δ = 175.2, 173.6 and 173.2 (RI), 172.5 and 171.5 (RI), 170.3, 170.1 and 169.9 (RI), 169.4, 163.7 and 163.6 (RI), 156.5, 156.3, 153.5 and 153.4 (RI), 143.8 and 143.5 (RI), 141.5 and 141.4 (RI), 137.8 and 137.0 (RI), 136.8 and 136.5 (RI), 129.5 and 129.2 (RI), 129.1 and 128.8 (RI), 128.0, 127.4 and 127.3 (RI), 127.1, 125.2 and 125.1 (RI), 123.8 and 123.6 (RI), 120.2, 83.4 and 83.2 (RI), 81.9 and 81.5 (RI), 79.5 and 79.3, 67.3, 64.8 and 64.6 (RI), 62.9, 50.5, 49.8 and 49.4, 49.0 and 48.6 (RI), 48.4, 47.0 and 46.9 (RI), 44.9 and 44.3 (RI), 40.4 and 40.0 (RI), 37.2 and 36.7 (RI), 36.0 and 35.8 (RI), 34.3 and 34.0 (RI), 29.7 an 29.4 (RI), 28.5 and 28.4 (RI), 28.20, 28.17, 27.2 and 27.0 (RI), 25.7 and 25.5 (RI), 18.1, 14.3 and 13.6 (RI), 13.5 and 13.4 (RI) ppm.

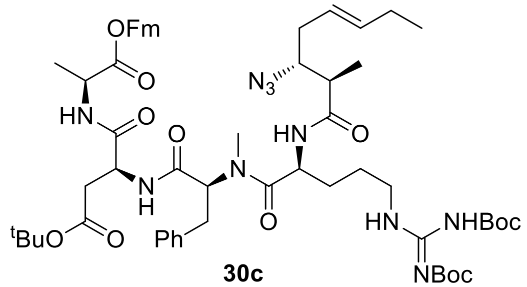

30c

Analytical data for **30c**: $[\alpha]_D^{20}$ = –45.7 (*c* = 1.94, MeOH); ^{1}H NMR (500 MHz, $CDCl_3$) δ = 11.51 (s) and 11.50 (s, 1H, RI), 8.30-8.32 (m) and 8.23-8.25 (m, overlp, 1H, RI), 8.21-8.24 (m, 1H), 7.75-7.79 (m, 2H), 7.57-7.61 (m, 2H), 7.38-7.45 (m, 2H), 7.32-7.36 (m, 3H), 7.27-7.30 (m, 2H), 7.165-7.22 (m, 2H), 7.05-7.06 (m, 1H), 6.99 (d, *J* = 7.5 Hz) and 6.77 (d, *J* = 8.5 Hz, 1H, RI), 5.59-5.65 (dt, *J* = 15.0, 6.5 Hz) and 5.53-5.59 (m, 1H, RI), 5.32-5.41 (m, 1H), 4.96 (dd, *J* = 11.5, 3.0 Hz) and 4.88 (dd, *J* = 14.0, 7.0 Hz, 1H, RI), 4.80-4.83 (m) and 4.20-4.23 (m, 1H, RI), 4.60-4.70 (m, 1H), 4.53-4.55 (m, 1H), 4.39-4.52 (m, 2H), 4.22-4.26 (m, 1H), 3.72 (brs) and 3.41 (dd, *J* = 11.0, 5.5 Hz, 1H, RI), 3.52-3.56 (m, 1H), 3.33-3.37 (m) and 2.94-2.95 (m, 1H, RI), 3.32 (dd, *J* = 13.5, 6.0 Hz) and 2.60 (dd, *J* = 17.0, 6.0 Hz, 1H, RI), 3.18-3.24 (m, 1H), 2.98-3.04 (m, 1H), 2.88 (s) and 2.86 (s, 3H, RI), 2.36-2.45 (m, 1H), 2.31-2.36 (m) and 2.22-2.25 (m, 1H, RI), 2.15-2.20 (m, 1H), 2.00-2.04 (m, 2H),; ^{13}C NMR (75 MHz, $CDCl_3$) δ = 175.5, 174.0 and 173.2 (RI), 172.6 and 172.5 (RI),

171.6, 170.4 and 170.0, 169.9 and 169.6, 163.7 and 163.5 (RI), 156.5 and 156.3 (RI), 153.4, 153.3, 143.9 and 143.7 (RI), 143.4, 141.5 and 141.4 (RI), 137.8, 137.0 and 136.6 (RI), 129.4 and 129.2 (RI), 129.1 and 128.8 (RI), 128.0, 127.4 and 127.3 (RI), 127.1, 125.2 and 125.1 (RI), 123.2 and 123.0 (RI), 120.2, 83.4 and 83.2 (RI), 82.0, 81.5, 79.4 and 79.3 (RI), 67.3, 64.8 and 64.7, 62.9, 50.4, 49.7 and 49.5 (RI), 48.6 and 48.3 (RI), 46.9 and 46.8 (RI), 45.1 and 44.3 (RI), 40.4 and 39.8 (RI), 37.1 and 36.5 (RI), 34.9, 34.2 and 33.9 (RI), 29.6 and 29.4 (RI), 28.5 and 28.4 (RI), 28.2, 28.1, 27.1 and 26.8 (RI), 25.7 and 25.3 (RI), 18.1, 14.9 and 14.6 (RI), 13.6 ppm.

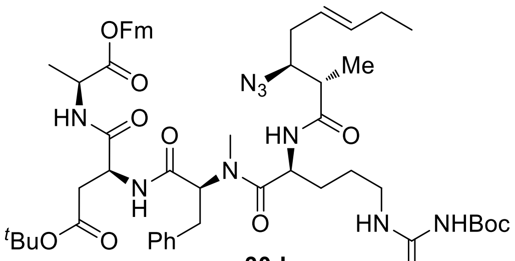

30d

Analytical data for **30d**: $[\alpha]_D^{20} = -26.1$ (c = 0.95, MeOH); ^{1}H NMR (500 MHz, $CDCl_3$) δ = 11.49 (s) and 11.47 (s, RI), 8.33-8.35 (m) and 8.01-8.04 (m, 1H, RI), 8.17-8.22 (m, 1H), 7.76-7.78 (m, 2H), 7.61-7.64 (m, 1H), 7.57-7.61 (m, 1H), 7.40-7.43 (m, 2H), 7.29-7.37 (m, 5H), 7.18-7.23 (m, 2H), 7.19 (d, J = 4.5Hz) and 7.06 (d, J = 4.5Hz, 1H), 5.57-5.65 (m,1H), 5.36-5.42 (m, 1H), 4.87-4.98 (m, 1H), 4.81-4.85 (m) and 4.16-4.19 (m, overlp, 1H, RI), 4.68-4.79 (brm, 1H), 4.61-4.64 (m, 1H), 4.40-4.52 (m, 2H), 4.23-4.26 (m, 1H), 3.52-3.61 (m, 1H), 3.38-3.44 (m, 1H), 3.27-3.37 (m, 1H), 3.16-3.27 (m, 1H), 3.01-3.06 (m, 1H), 2.95-2.99 (m) and 2.59 (dd, J = 17.0, 6.0 Hz, 1H, RI), 2.89 (s) and 2.87 (s, 3H, RI), 2.69-2.80 (m, 1H), 2.28-2.43 (brm,2H), 2.12-2.20 (m, 1H), 1.97-2.06 (m, 2H), 1.70-1.77 (m, 2H), 1.56-1.64 (m, 2H), 1.51 (s, 9H), 1.49 (s, 9H), 1.45 (s) and 1.42 (s, 9H), 1.37 (t, J = 7.5 Hz, 3H), 1.15 (d, J = 7.0 Hz) and 1.10 (d, J = 7.0 Hz, 3H), 0.97 (t, J = 7.5 Hz, 3H); ^{13}C NMR (75 MHz, $CDCl_3$) δ = 175.3, 173.6 and 173.1 (RI), 172.4 and 172.1 (RI), 170.3 and 170.1 (RI), 169.9 and 169.8 (RI), 169.5 and 169.2 (RI), 163.6, 156.4, 156.2, 153.3 and 153.2 (RI), 143.8 and 143.3 (RI), 141.4 and 141.3 (RI), 137.7 and 137.5 (RI), 136.9 and 136.6 (RI), 129.4 and 129.1 (RI), 129.0, 128.7, 127.9, 127.2, 125.1 and 125.0 (RI), 123.2 and 123.1 (RI), 120.1, 83.3 and 83.1 (RI), 81.9 and 81.4 (RI), 79.3 and 79.2 (RI), 67.1, 64.7 and 64.6 (RI), 62.9 and 62.6 (RI), 52.7, 50.7 and 50.3 (RI), 49.8 and 49.6 (RI), 48.9 and 48.5 (RI), 46.8 and 46.7 (RI), 44.8 and 44.5 (RI), 40.4 and 40.0 (RI), 37.2 and 36.8 (RI), 34.8, 34.2 and 33.8 (RI), 29.7 and 29.6 (RI), 28.3, 28.1, 28.0, 26.2 and 25.2 (RI), 25.6, 17.8, 15.0 and 14.2 (RI), 13.5 ppm.

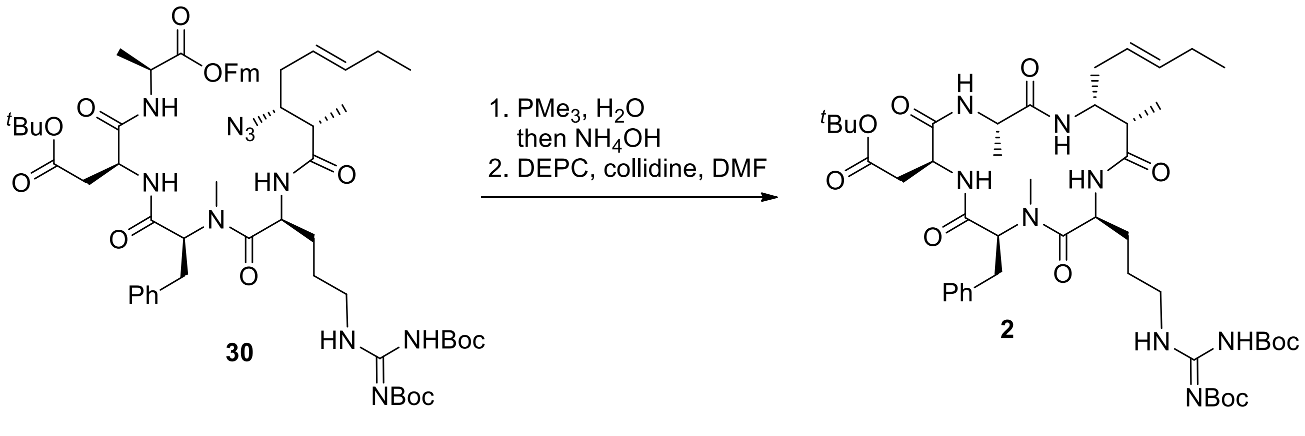

To a solution of **30** (70 mg, 0.063 mmol) in water (0.3 mL) and THF (2 mL), PMe_3 (250 µL, 1.0 M in THF) was added at 0℃. The reaction mixture was stirred at room temperature for 3 h, and then quenched by the adiition of an aqueous solution of NH_4OH (0.5 mL, 30% in water). The cloudy solution was stirred at room temperature overnight. Volatiles were removed in *vacuo*, the residue was azeotropically dried with benzene (3 mL x 3) prior to be dissolved in DMF (50mL). To this solution,

DEPC (140 µL, 0.9 mmol) and DIPEA (160 µL, 0.9 mmol) were successively added at room temperature. The reaction mixture was stirred at room temperature for 36 h and concentrated in *vacuo*. The residue was filtered through a short pad of silica gel, eluting with MeOH/CH_2Cl_2 (3/97). The filtrate was concentrated, the residue was then purified by HPLC to give **2** (32 mg, 56%) as a viscous oil.

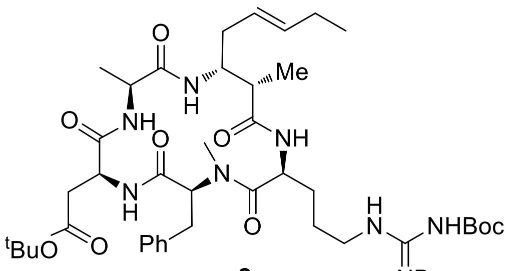

2a

Analytical data for **2a**: $[\alpha]_D^{20}$ = –65.7 (*c* = 1.1, MeOH); ^{1}H NMR (500 MHz, $CDCl_3$) *δ* = 11.47 (s, 1H), 8.35 (m, 1H), 7.91 (d, *J* = 7.5 Hz, 1H), 7.60 (d, *J* = 7.5 Hz, 1H), 7.35 (m, 2H), 7.29 (d, *J* = 7.4 Hz, 1H), 7.17 (d, *J* = 7.2 Hz, 2H), 6.34 (d, *J* = 6.5 Hz, 1H), 5.92 (d, *J* = 9.0 Hz, 1H), 5.62 (dt, *J* = 15.2, 6.3 Hz, 1H), 5.22 (dt, *J* = 15.2, 6.3 Hz, 1H), 4.80 (m, 1H), 4.64 (m, 1H), 4.50 (m, 1H), 3.74 (dd, *J* = 11.1, 4.7 Hz, 1H), 3.66 (m, 1H), 3.48 (t, *J* = 12.4 Hz, 2H), 3.42 (m, 1H), 3.23 (dd, *J* = 12.7, 4.6 Hz, 1H), 3.15 (dd, *J* = 11.7, 4.9 Hz, 1H), 2.88-2.91 (m, overlp, 1H), 2.88 (s, 3H), 2.60 (dd, *J* = 16.6, 4.8 Hz, 1H), 2.33-2.39 (m, 1H), 2.01-2.07 (m, 1H), 1.95-1.99 (m, 2H), 1.83-1.95 (brm, 1H), 1.58-1.68 (brm, 3H), 1.55 (d, *J* = 7.4 Hz, 3H), 1.51 (s, 9H), 1.49 (s, 9H), 1.46 (s, 9H), 1.13 (d, *J* = 6.9 Hz, 3H), 0.93 (t, *J* = 7.5 Hz, 3H); ^{13}C NMR (75 MHz, $CDCl_3$) *δ* = 173.9, 173.6, 173.0, 171.8, 171.1, 169.1, 163.8, 156.6, 153.7, 136.6, 135.9, 129.4, 129.3, 127.8, 125.2, 83.6, 82.8, 79.7, 68.3, 54.3, 51.4, 49.2, 48.2, 42.4, 40.8, 40.6, 35.6, 34.2, 32.3, 30.7, 29.6, 28.6, 28.4, 25.9, 23.0, 18.4, 14.4, 13.7 ppm; HRMS (ESI) m/z calcd for $C_{46}H_{73}N_8O_{11}$ $(M+H)^+$ 913.5399, found 913.5374.

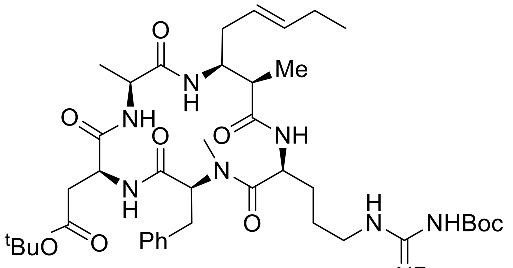

2b

Analytical data for **2b**: $[\alpha]_D^{20}$ = –77.2 (*c* = 1.0, MeOH); ^{1}H NMR (500 MHz, $CDCl_3$) δ = 11.5 (s, 1H), 8.35 (m, 1H), 7.83 (d, *J* = 9.0 Hz, 1H), 7.66 (d, *J* = 8.0 Hz, 1H), 7.33-7.36 (m, 3H), 7.18 (d, *J* = 9.0 Hz, 2H), 6.33 (d, *J* = 7.0 Hz, 1H), 6.01 (d, *J* = 8.0 Hz, 1H), 5.55 (dt, *J* = 15.2, 6.9 Hz, 1H), 5.30 (dt, *J* = 15.2, 6.0 Hz, 1H), 4.80-4.86 (m) and 4.69-4.72 (m, 1H, RI), 4.60-462 (m) and 4.54-4.57 (m, 1H, RI), 3.94-4.02 (m, 1H), 3.74-3.77 (dd, *J* = 11.5, 5.0 Hz, 1H), 3.54-3.60 (m, 1H), 3.45-3.49(m, 1H), 3.40-3.43 (m, 1H), 3.18-3.25 (m, 1H), 3.02-3.06 (m, 1H), 2.89 (s) and 2.88 (s, major, 3H, RI), 2.83-2.85 (m, 1H), 2.78-2.89 (m, 1H), 2.57-2.64 (m, 2H), 2.29-2.34 (m) and 2.05-2.10 (m, 1H, major, RI), 1.96-2.02 (m, 2H), 1.60-1.68 (m, 2H), 1.51 (s, 9H), 1.49 (s, 9H), 1.46 (s, 9H), 1.30-1.34 (m, 1H, overlp), 1.26 (d, *J* = 7.2 Hz, 3H), 1.11 (d, *J* = 7.2 Hz, 3H), 0.94 (t, *J* = 7.5 Hz, 3H), 0.88-0.93 (m, major) and 0.18-0.22 (m, 1H, RI); ^{13}C NMR (125 MHz, $CDCl_3$) *δ* = 175.7, 173.7, 173.4, 172.0, 171.5, 169.8, 163.9, 153.8, 150.2, 138.1 and 136.8 (major, RI), 135.7, 129.6, 129.3, 127.8, 125.6 (major) and 124.8 (RI), 83.7, 82.6, 79.7, 68.6, 64.3, 56.1, 51.2, 50.1, 49.0, 46.6, 41.0, 40.5, 37.4 and 35.9 (major), 34.3, 33.7 (major) and 32.3 (RI), 30.0 (major) and 29.5 (RI), 28.7, 28.5, 28.4, 25.9, 18.8 (major) and 17.4 (RI), 14.0, 11.8 ppm.

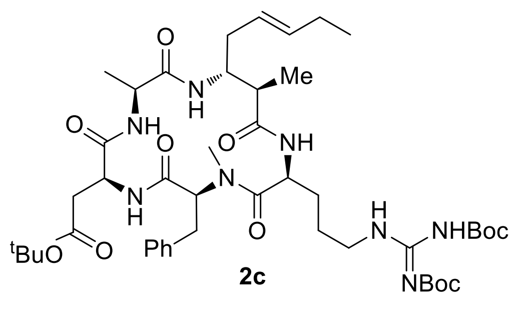

Analytical data for **2c**: $[\alpha]_D^{20} = -74.2$ (c = 0.91, MeOH); ^{1}H NMR (500 MHz, $CDCl_3$) δ = 11.53 (s, 1H), 8.34 (brs, 1H), 7.83 (d, J = 9.1 Hz, 1H), 7.60 (d, J = 7.6 Hz, 1H), 7.34-7.37 (m, 2H), 7.28 (d, J = 10.5 Hz, 1H), 7.18 (d, J = 7.4 Hz, 2H), 5.98 (d, J = 8.7 Hz, 1H), 5.85 (d, J = 9.0 Hz, 1H), 5.59 (dt, J = 15.1, 6.3 Hz, 1H), 5.28 (dt, J = 15.1, 6.3 Hz, 1H), 4.82 (brs, 1H), 4.60-4.63 (m, 1H), 4.54-4.57 (m, 1H), 3.98-4.03 (m, 1H), 3.72-3.75 (m, 1H), 3.46-3.51 (m, 2H), 3.40-3.43 (m, 1H), 3.24 (dd, J = 13.0, 1.9 Hz, 1H), 3.18 (dd, J = 17.1, 4.9 Hz, 1H), 2.82 (s, 3H), 2.61-2.64 (m, 1H), 2.58-2.61 (m, 1H), 2.30-2.33 (m, 1H), 2.12-2.16 (m, 1H), 1.95-1.98 (m, 2H), 1.86 (brs, 1H), 1.60-1.63 (m, 3H), 1.57 (d, J = 12.0 Hz, 3H), 1.51 (s, 9H), 1.50 (s, 9H), 1.46 (s, 9H), 1.21 (d, J = 7.5 Hz, 3H), 0.93 (t, J = 7.5 Hz, 3H); ^{13}C NMR (125 MHz, $CDCl_3$) δ = 173.8, 173.8, 173.6, 171.6, 170.2, 169.3, 163.5, 156.5, 153.5, 136.5, 136.1, 129.4, 129.2, 127.5, 124.4, 83.5, 82.5, 79.5, 68.3, 52.3, 51.0, 50.0, 48.2, 46.5, 40.5, 40.3, 36.6, 35.7, 34.2, 29.9, 29.6, 28.5, 28.3, 28.2, 25.6, 19.1, 16.5, 13.7 ppm.

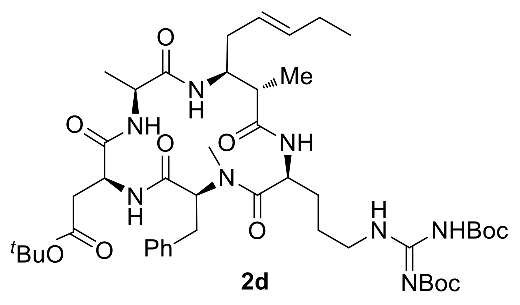

Analytical data for **2d**: $[\alpha]_D^{20} = -74.3$ (c = 0.98, MeOH); ^{1}H NMR (500 MHz, $CDCl_3$) δ = 11.50 (s, 1H), 8.34-8.35 (m, 1H), 8.17 (d, J = 10.5 Hz, 1H), 7.61 (d, J = 8.1 Hz, 1H), 7.34 (t, J = 7.5 Hz, 2H), 7.25-7.27 (overlp, 1H), 7.17 (d, J = 7.5 Hz, 2H), 6.62 (d, J = 6.5 Hz, 1H), 6.24 (d, J = 7.7 Hz, 1H), 5.48 (dt, J = 15.2, 6.3 Hz, 1H), 5.24 (dt, J = 15.2, 6.3 Hz, 1H), 4.55-4.62 (m, overlp, 3H), 3.74 (dd, J = 11.2, 4.8 Hz, 1H), 3.38-3.49 (brm, overlp, 4H), 3.21 (dd, J = 13.1, 4.9 Hz, 2H), 3.03-3.08 (m, 1H), 2.95 (s, 3H), 2.73-2.79 (m, 1H), 2.56 (dd, J = 16.8, 4.7 Hz, 1H), 2.20-2.24 (m, 1H), 1.97-2.01 (m, 2H), 1.84-1.88 (m, 2H), 1.60-1.70 (m, 2H), 1.52 (s, 9H), 1.50 (s, 9H), 1.46 (s, overlp, 9H), 1.45 (d, overlp, 3H), 1.08 (d, J = 6.8 Hz, 3H), 0.96 (t, J = 7.5 Hz, 3H); ^{13}C NMR (125 MHz, $CDCl_3$) δ =173.6, 173.3, 172.8, 171.8, 171.5, 170.8, 163.8, 156.6, 153.7, 136.9, 135.1, 129.4, 129.3, 127.6, 125.6, 83.4, 82.5, 79.5, 68.0, 56.1, 52.9, 48.9, 48.0, 42.1, 40.6, 40.3, 35.3, 33.9, 30.4, 29.3, 28.3, 28.1, 28.1, 25.7, 25.5, 17.7, 15.8, 14.0 ppm.

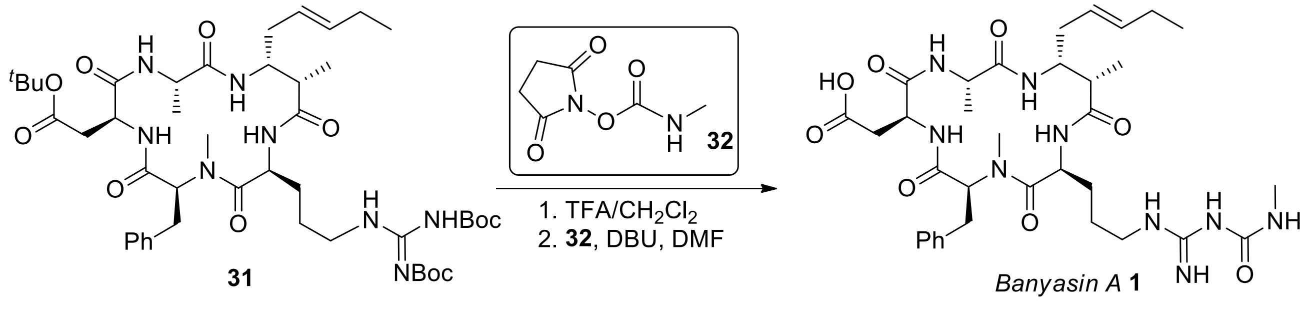

To a solution of **31** (9 mg, 0.01 mmol) in CH_2Cl_2 (1.0 mL), TFA (0.5 mL) was added at 0 ℃. The resulting solution was stirred at room temperature for 4 h. Volatiles were removed in *vacuo*. The residue was dried under high vacuum for 3 h and then dissolved in DMF (0.5mL) at 0℃. To this solution, **32** (2.2 mg, 0.013 mmol) and DBU (10 μL, 0.066 mmol) were successively added. The reaction mixture was stirred at 40 ℃ for 2 h and then cooled to 0 ℃, followed by addition of TFA (0.2 mL) and concentrated under high vacuum. The residue was purified by HPLC to give banyasin

A **1** (4.6 mg, 66%).

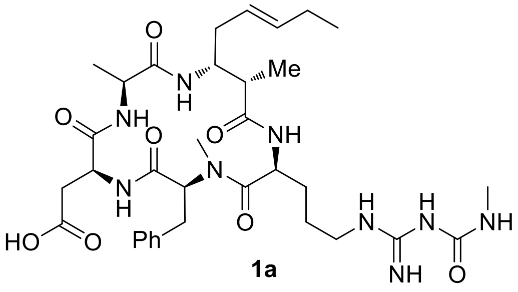

1a

Analytical data for banyasin A **1a**: $[\alpha]_D^{20}$ = –45.6 (*c* = 0.70, MeOH); ^{1}H NMR (300 MHz, $CDCl_3/CD_3OD$ 4:1) δ = 7.27 (t, *J* = 7.5 Hz, 2H), 7.21 (d, *J* = 7.0 Hz, 1H), 6.96 (d, *J* = 7.0 Hz, 2H), 5.61 (dt, *J* = 15.0, 6.0 Hz, 1H), 5.20 (dt, *J* = 15.0, 7.0 Hz, 1H), 4.65-4.68 (m, 1H), 4.53-4.55 (m, 1H), 4.37 (brs, 1H), 3.60-3.65 (m, 3H), 3.20-3.22 (m, 1H), 3.14-3.19 (m, 1H), 3.02-3.05 (m, 1H), 2.88-2.90 (m, 1H), 2.76 (s, 3H), 2.71 (s, 3H), 2.49-2.51 (m, 1H), 2.40-2.44 (m, 1H), 2.30-2.32 (m, 1H), 2.01-2.03 (m, 1H), 1.93-1.96 (m, 2H), 1.65-1.68 (m, 2H), 1.63-1.65 (m, 2H), 1.51 (d, *J* = 7.0 Hz, 3H), 1.10 (d, *J* = 7.0 Hz, 3H), 0.91 (t, *J* = 7.5 Hz, 3H); ^{13}C NMR (75 MHz, $CDCl_3/CD_3OD$ 4:1) δ = 178.2, 174.1, 173.6, 173.4, 171.3, 171.2, 155.8, 155.2, 136.8, 136.0, 129.3, 129.1, 127.6, 124.9, 66.4, 54.1, 52.1, 42.5, 40.5, 37.0, 33.8, 31.8, 30.8, 28.6, 26.3, 25.8, 24.8, 22.9, 17.9, 13.7, 13.5 ppm; HRMS (ESI) m/z calcd for $C_{34}H_{52}N_9O_8$ $(M+H)^+$ 714.3939, found 714.3913.

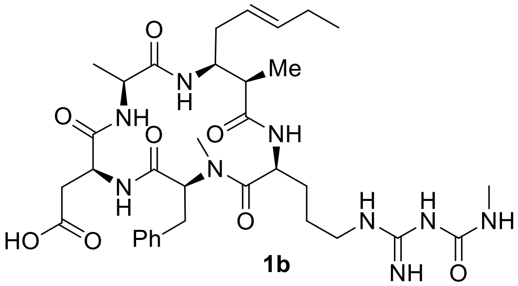

1b

Analytical for banyasin A **1b**: $[\alpha]_D^{20}$ = –59.4 (*c* = 0.70, MeOH); ^{1}H NMR (500 MHz, $CDCl_3/CD_3OD$ 4:1) δ = 7.22-7.26 (m, 2H), 7.18 (d, *J* = 7.6 Hz, 1H), 7.09 (d, *J* = 6.4 Hz, 2H), 5.50 (dt, *J* = 15.1, 6.0 Hz, 1H), 5.26 (dt, *J* = 15.1, 6.9 Hz, 1H), 4.84-4.89 (m, 1H), 4.55-4.62 (m, 1H), 4.42-4.44 (m, 1H), 4.15-4.18 (m, 1H), 3.71-3.74 (m, 1H), 3.49-3.48 (m, 1H), 3.13-3.23 (m, 1H), 2.98-3.02 (m, 2H), 2.86-2.90 (m, 1H, overlp), 2.88 (s, 3H), 2.73 (s) and 2.68 (s, 3H), 2.64-2.66 (m, 2H), 2.57-2.59 (m, 1H), 2.38-2.45 (m, 1H), 2.00-2.03 (m, 1H), 1.92-2.00 (m, 2H), 1.65-1.75 (m, 3H), 1.48 (d, *J* = 6.8 Hz) and 1.33 (d, *J* = 7.5 Hz, 3H, RI), 1.22 (d, *J* = 6.9 Hz) and 1.03 (d, *J* = 6.9 Hz, 3H, RI), 1.08-1.12 (m) and 0.44 (brs, 1H), 0.91 (t, *J* = 7.5 Hz, 3H); ^{13}C NMR (125 MHz, $CDCl_3/CD_3OD$ 4:1) δ = 177.7, 173.8 and 173.6 (RI), 172.7, 172.6, 171.3, 170.1, 155.9, 155.2, 138.6, 135.8, 129.2, 128.6, 127.3, 124.4, 68.6, 54.9, 52.5, 51.9, 45.8, 40.8 and 40.6 (RI), 40.2 and 39.9 (RI), 38.5, 34.7 and 33.9 (RI), 32.9 and 32.2 (RI), 30.4 and 30.0, 29.1 and 28.5 (RI), 27.0 and 26.4 (RI), 25.8, 24.5 and 24.3 (RI), 18.1 and 17.0 (RI), 13.8, 13.8 ppm.

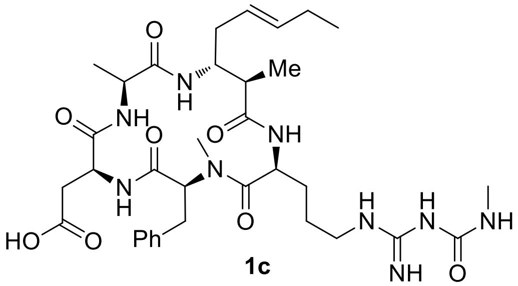

1c

Analytical data for banyasin A **1c**: $[\alpha]_D^{20}$ = –37.3 (*c* = 0.70, MeOH); ^{1}H NMR (500 MHz, $CDCl_3/CD_3OD$ 4:1) δ = 7.27 (t, *J* = 7.0 Hz, 2H), 7.22 (d, *J* = 7.0 Hz, 1H), 7.02-7.05 (m, 2H), 5.56 (dt, *J*=15.1, 6.0 Hz) and 5.42 (dt, *J* = 15.1, 6.0 Hz, 1H, RI), 5.26 (dt, *J* = 15.2, 6.9 Hz, 1H), 5.02-5.04 (m) and 4.70-4.72 (m, 1H, RI), 4.82-4.84 (m) and 4.44-4.46 (m, 1H, RI), 4.40-4.44 (m, 1H), 3.71-3.73 (m, 1H), 3.62-3.66 (m, 1H), 3.18-3.24 (m, 1H), 3.14-3.18 (m, 1H), 2.94-3.00 (m, 1H), 2.75-2.80 (m, 2H, overlp), 2.76 (s, 3H), 2.70 (s, 3H), 2.61-2.64 (m, 1H), 2.54-2.58 (m, 1H), 2.26-2.30 (m, 1H), 2.15-2.19 (m) and 1.09-2.13 (m, 1H, RI), 1.92-1.98 (m, 2H), 1.68-1.72 (m, 2H), 1.55-1.61 (m, 2H), 1.47 (d, *J* = 7.0 Hz) and 1.37 (d, *J* = 7.5 Hz, 3H), 1.23 (d, *J* = 7.0 Hz) and 1.16 (d, *J* = 7.0 Hz, 3H, RI), 0.91 (t, *J* = 7.5 Hz, 3H); ^{13}C NMR (125 MHz, $CDCl_3/CD_3OD$ 4:1) δ = 174.7, 174.3, 173.4, 172.2, 171.8, 170.9, 155.5, 155.4, 137.2, 136.6, 130.0, 129.4 and 129.3 (RI), 127.7, 124.2, 68.8, 52.2, 50.2, 48.1, 46.5, 40.6, 40.5, 37.7, 36.7, 34.2, 30.0,

29.1, 26.4, 25.8, 24.9, 18.8, 16.7, 13.8 ppm.

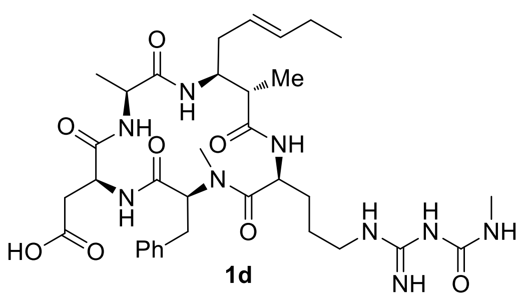

1d

Analytical data for banyasin A **1d**: $[\alpha]_D^{20} = -53.1$ (c = 0.70, MeOH); ^{1}H NMR (500 MHz, $CDCl_3/CD_3OD$ 4:1) δ = 7.24 (t, J = 7.4 Hz, 2H), 7.17 (d, J = 7.2 Hz, 1H), 6.92 (brs, 2H), 5.44 (dt, J = 15.1, 6.0 Hz, 1H), 5.22 (dt, J = 15.1, 6.9 Hz, 1H), 4.54 (dd, J = 14.0, 6.8 Hz, 1H), 4.44-4.46 (m, 1H), 4.39-4.41 (m, 1H), 3.55-3.59 (m, 2H), 3.21-3.25 (m, 1H), 3.12-3.17 (m, 1H), 3.05-3.09 (m, 2H), 2.76 (s, 3H), 2.74 (s, 3H), 2.67-2.69 (m, 2H), 2.55 (dd, J = 16.5, 8.4 Hz, 1H), 2.16-2.22 (m, 1H), 2.02-2.04 (m, 1H), 1.93-1.98 (m, 2H), 1.65-1.69 (m, 2H), 1.52-1.56 (m, 2H), 1.38 (d, J = 7.0 Hz, 3H), 1.03 (d, J = 6.0 Hz, 3H), 0.92 (t, J = 7.5 Hz, 3H); ^{13}C NMR (125 MHz, $CDCl_3/CD_3OD$ 4:1) δ = 176.9, 173.8, 173.4, 173.4, 171.7, 171.1, 156.0, 155.4, 137.1, 135.2, 129.3, 128.9, 127.4, 125.3, 68.1, 57.0, 53.6, 51.6, 42.8, 40.7, 40.5, 37.3, 33.8, 32.2, 28.0, 26.1, 25.7, 25.0, 24.0, 17.2, 15.1, 13.8 ppm.

NMR spectra:

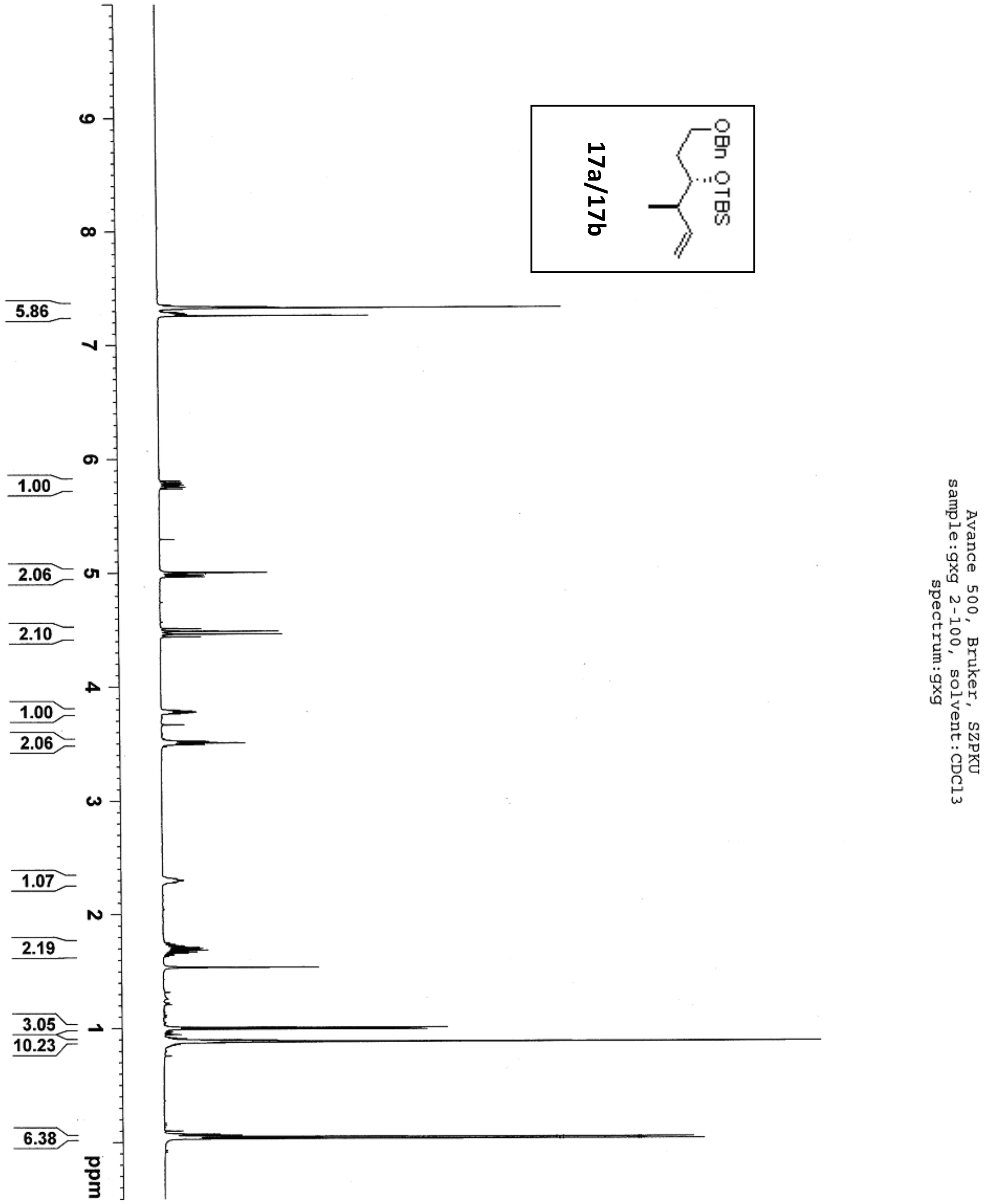
OBn OTBS
17a/17b
Avance 500, Bruker, SZPKU
sample:gxg 2-100, solvent:CDCl3
spectrum:gxg
5.86
1.00
2.06
2.10
1.00
2.06
1.07
2.19
3.05
10.23
6.38
ppm

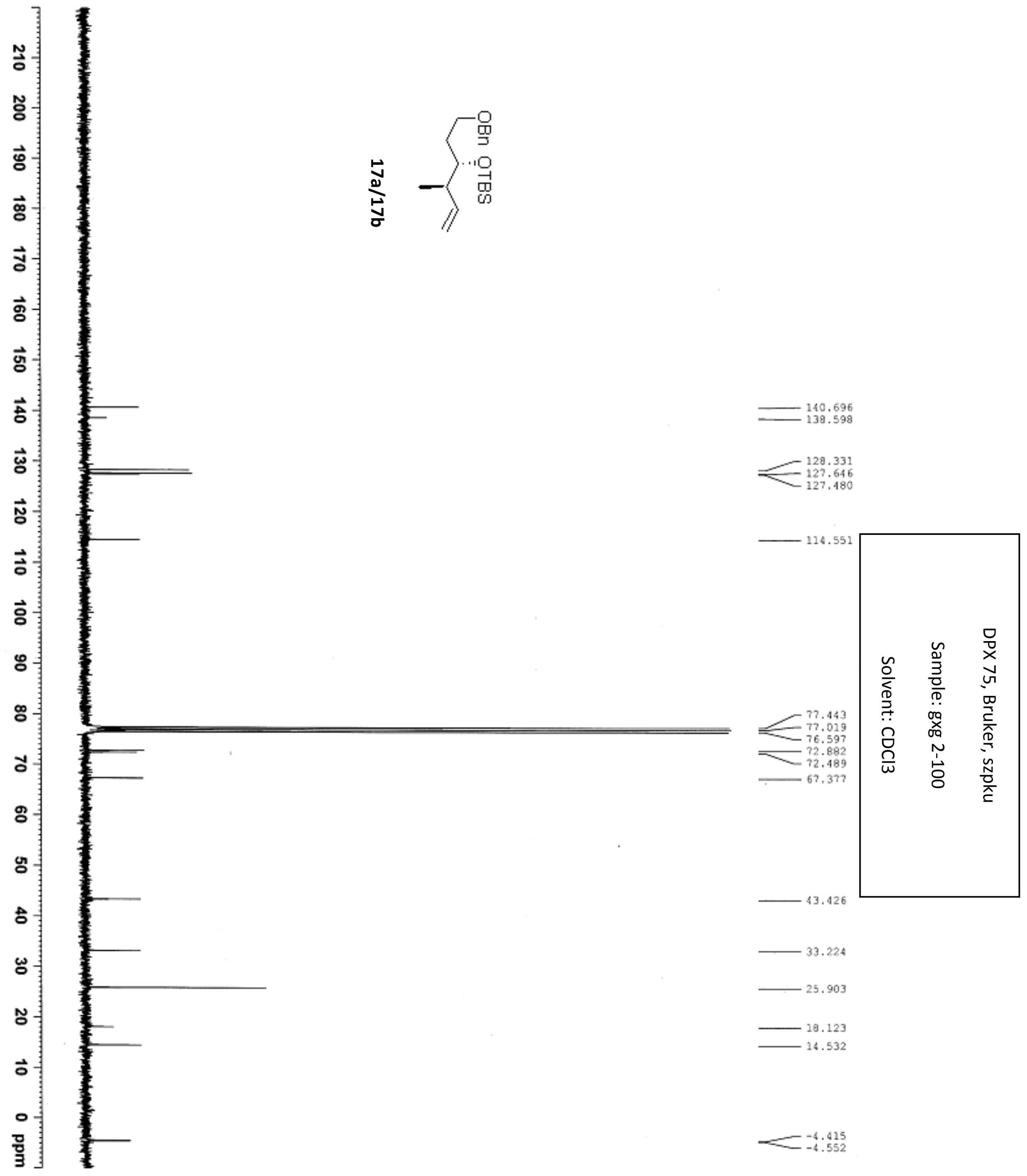
OBn OTBS
17a/17b
140.696
138.598
128.331
127.646
127.480
114.551
77.443
77.019
76.597
72.882
72.489
67.377
43.426
33.224
25.903
18.123
14.532
-4.415
-4.552
DPX 75, Bruker, szpku
Sample: gxg 2-100
Solvent: CDCl3
210 200 190 180 170 160 150 140 130 120 110 100 90 80 70 60 50 40 30 20 10 0 ppm

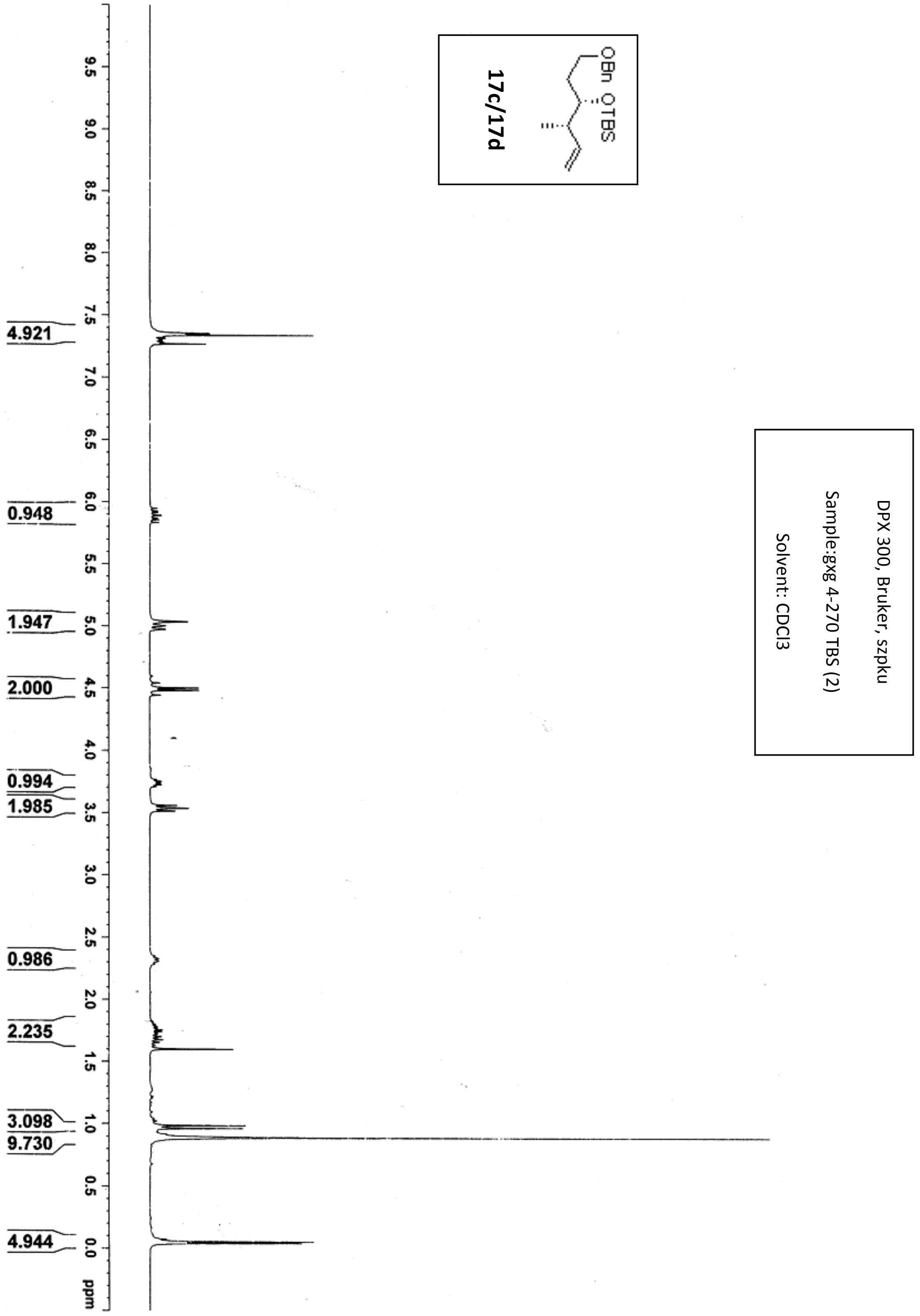

OBn OTBS
17c/17d
DPX 300, Bruker, szpku
Sample:gxg 4-270 TBS (2)
Solvent: CDCl3
4.921
0.948
1.947
2.000
0.994
1.985
0.986
2.235
3.098
9.730
4.944
ppm

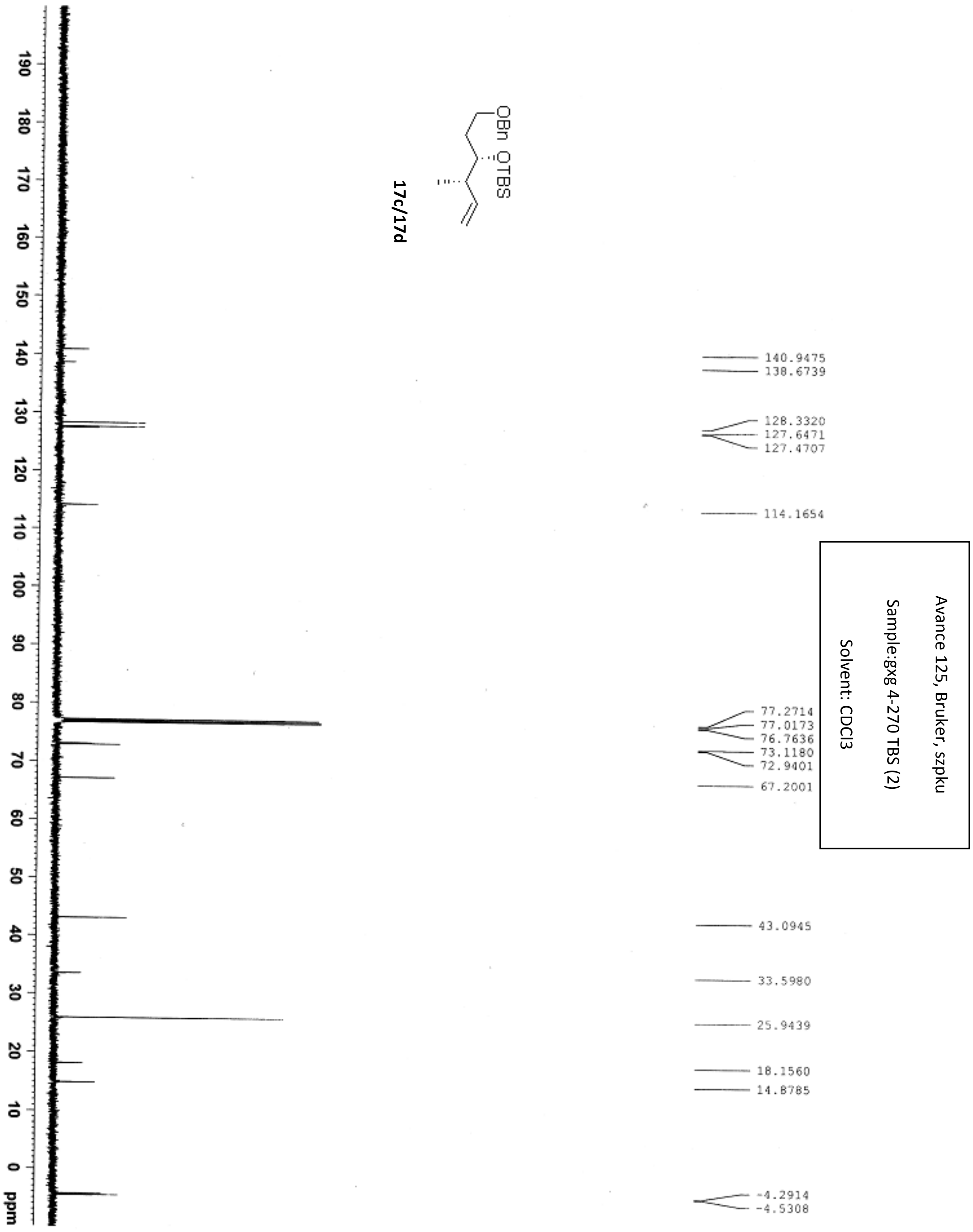
OBn OTBS
17c/17d
140.9475
138.6739
128.3320
127.6471
127.4707
114.1654
77.2714
77.0173
76.7636
73.1180
72.9401
67.2001
43.0945
33.5980
25.9439
18.1560
14.8785
-4.2914
-4.5308
Avance 125, Bruker, szpku
Sample:gxg 4-270 TBS (2)
Solvent: CDCl3
ppm

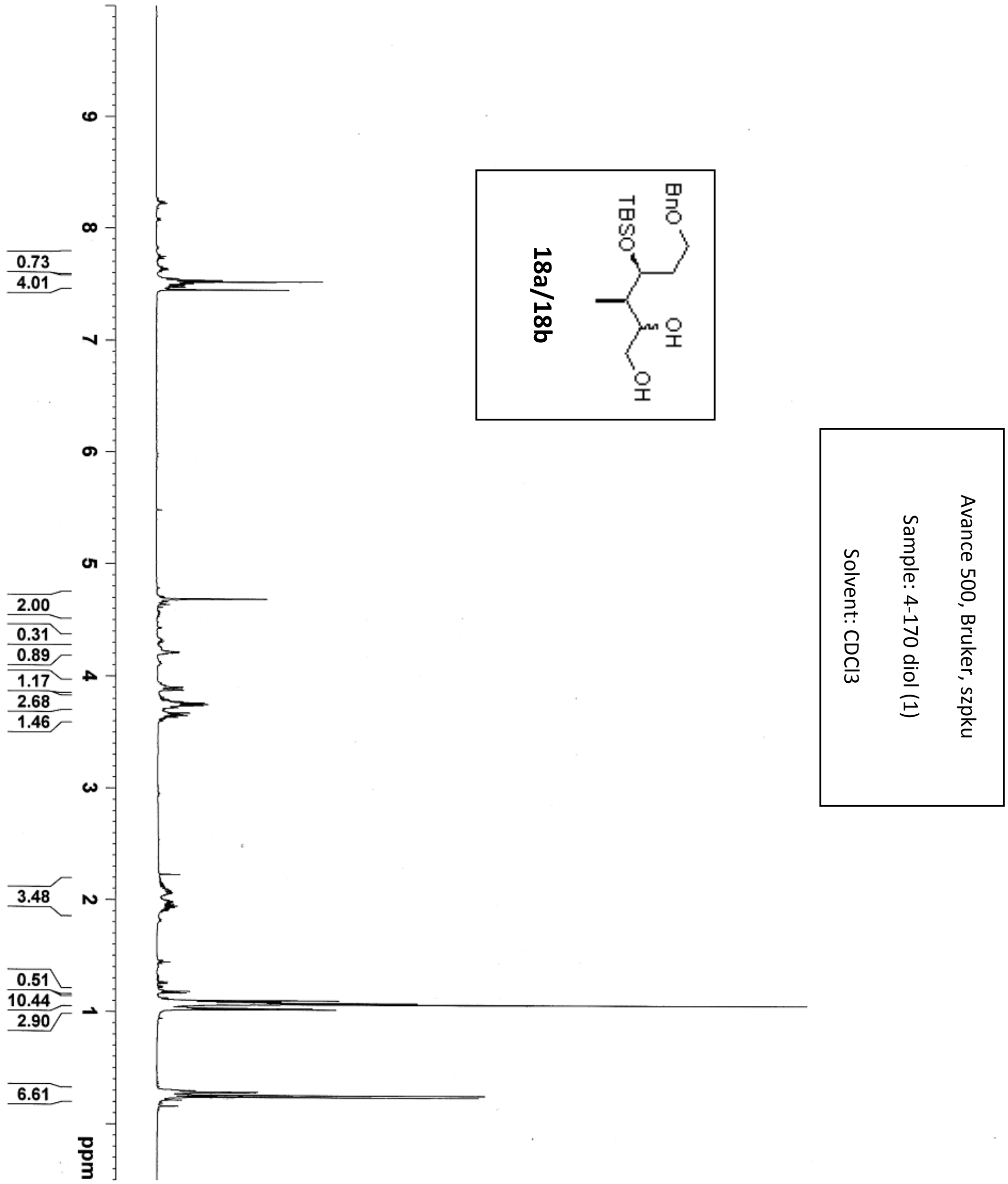
18a/18b
BnO
TBSO
OH
OH
Avance 500, Bruker, szpku
Sample: 4-170 diol (1)
Solvent: CDCl3
ppm

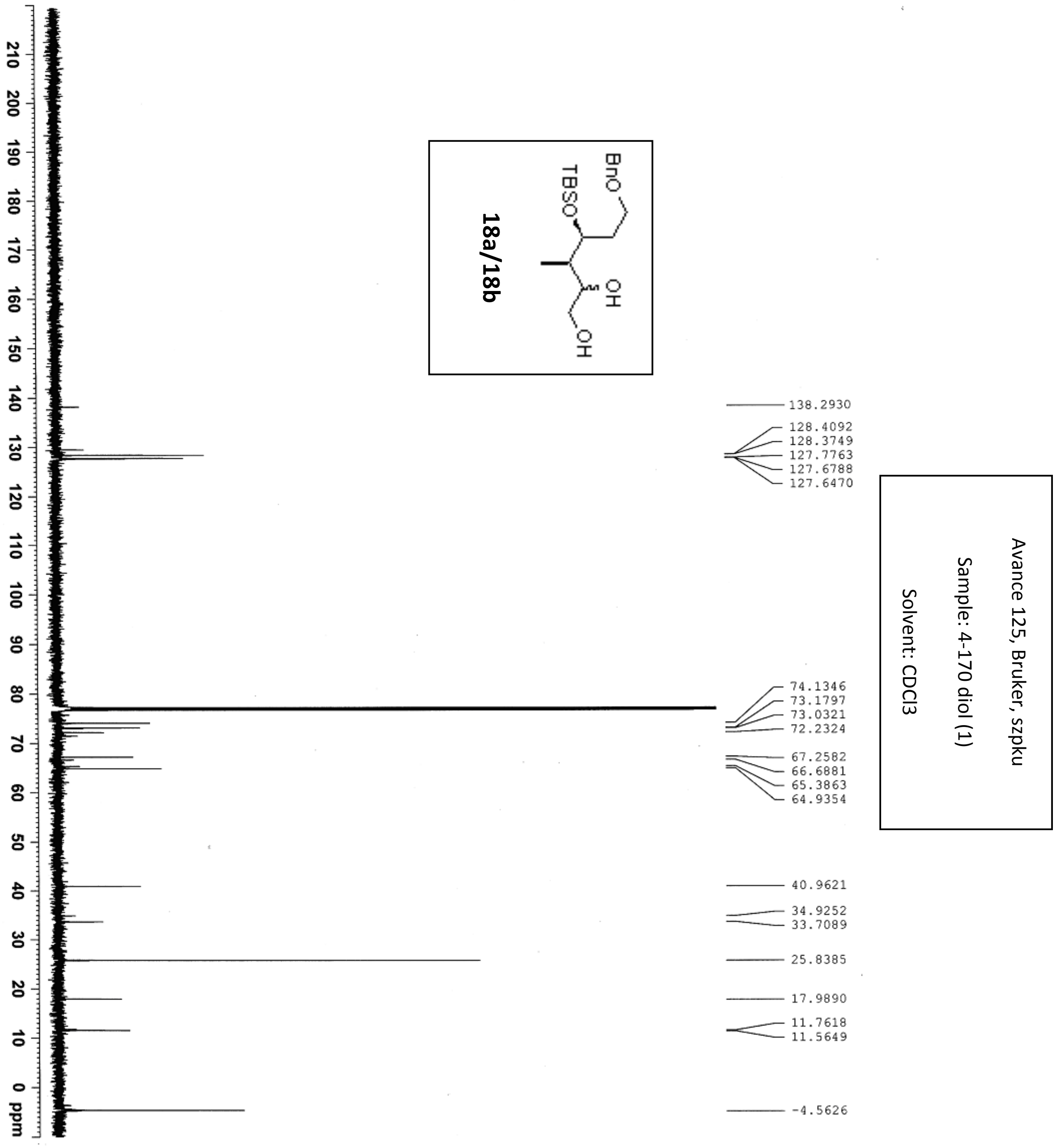
18a/18b
Avance 125, Bruker, szpku
Sample: 4-170 diol (1)
Solvent: CDCl3
138.2930
128.4092
128.3749
127.7763
127.6788
127.6470
74.1346
73.1797
73.0321
72.2324
67.2582
66.6881
65.3863
64.9354
40.9621
34.9252
33.7089
25.8385
17.9890
11.7618
11.5649
-4.5626
ppm

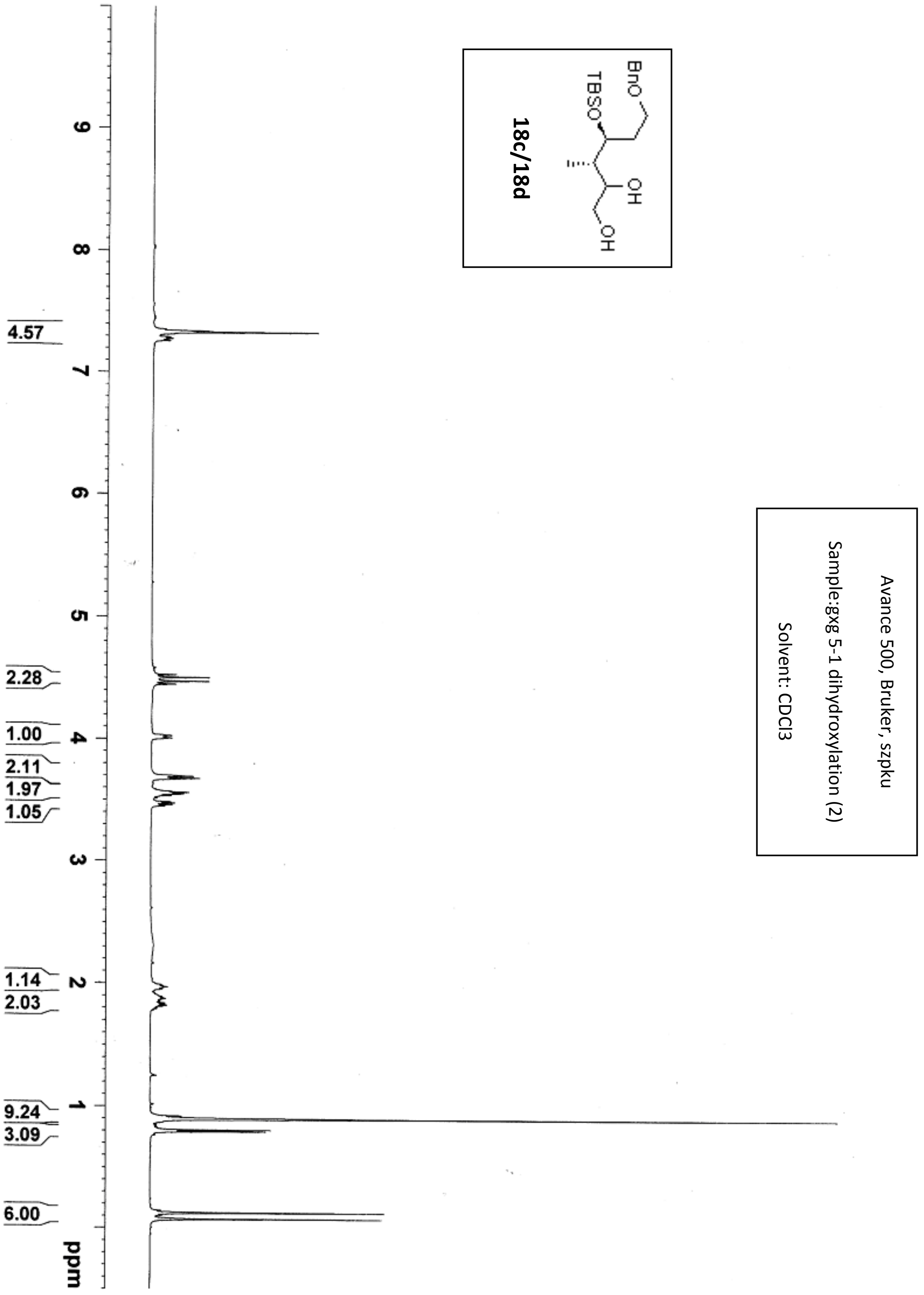
BnO
TBSO
OH
OH
18c/18d
Avance 500, Bruker, szpku
Sample:gxg 5-1 dihydroxylation (2)
Solvent: CDCl3
4.57
2.28
1.00
2.11
1.97
1.05
1.14
2.03
9.24
3.09
6.00
9
8
7
6
5
4
3
2
1
ppm

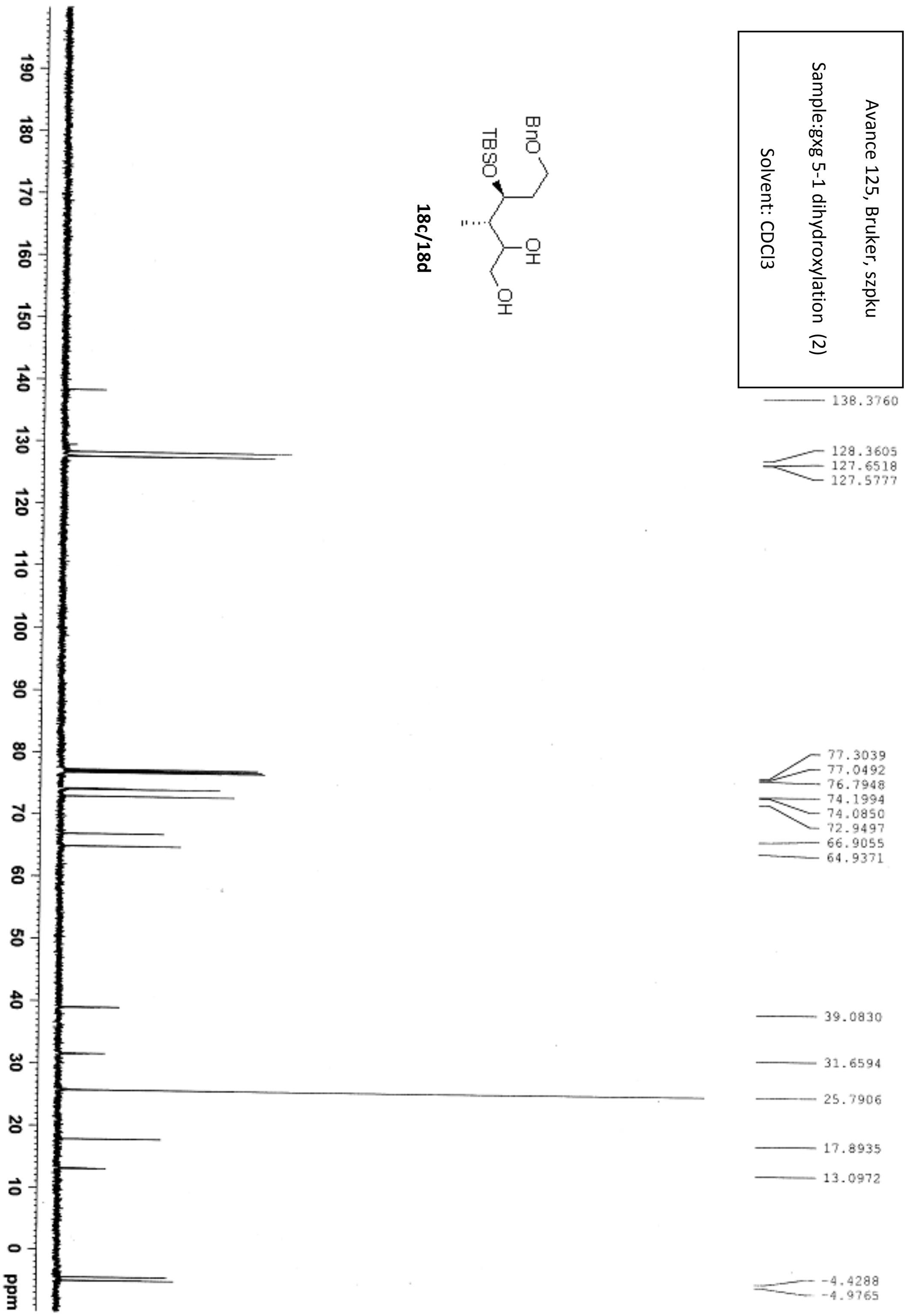
Avance 125, Bruker, szpku
Sample:gxg 5-1 dihydroxylation (2)
Solvent: CDCl3
BnO
TBSO
OH
OH
18c/18d
138.3760
128.3605
127.6518
127.5777
77.3039
77.0492
76.7948
74.1994
74.0850
72.9497
66.9055
64.9371
39.0830
31.6594
25.7906
17.8935
13.0972
-4.4288
-4.9765
190
180
170
160
150
140
130
120
110
100
90
80
70
60
50
40
30
20
10
0
ppm

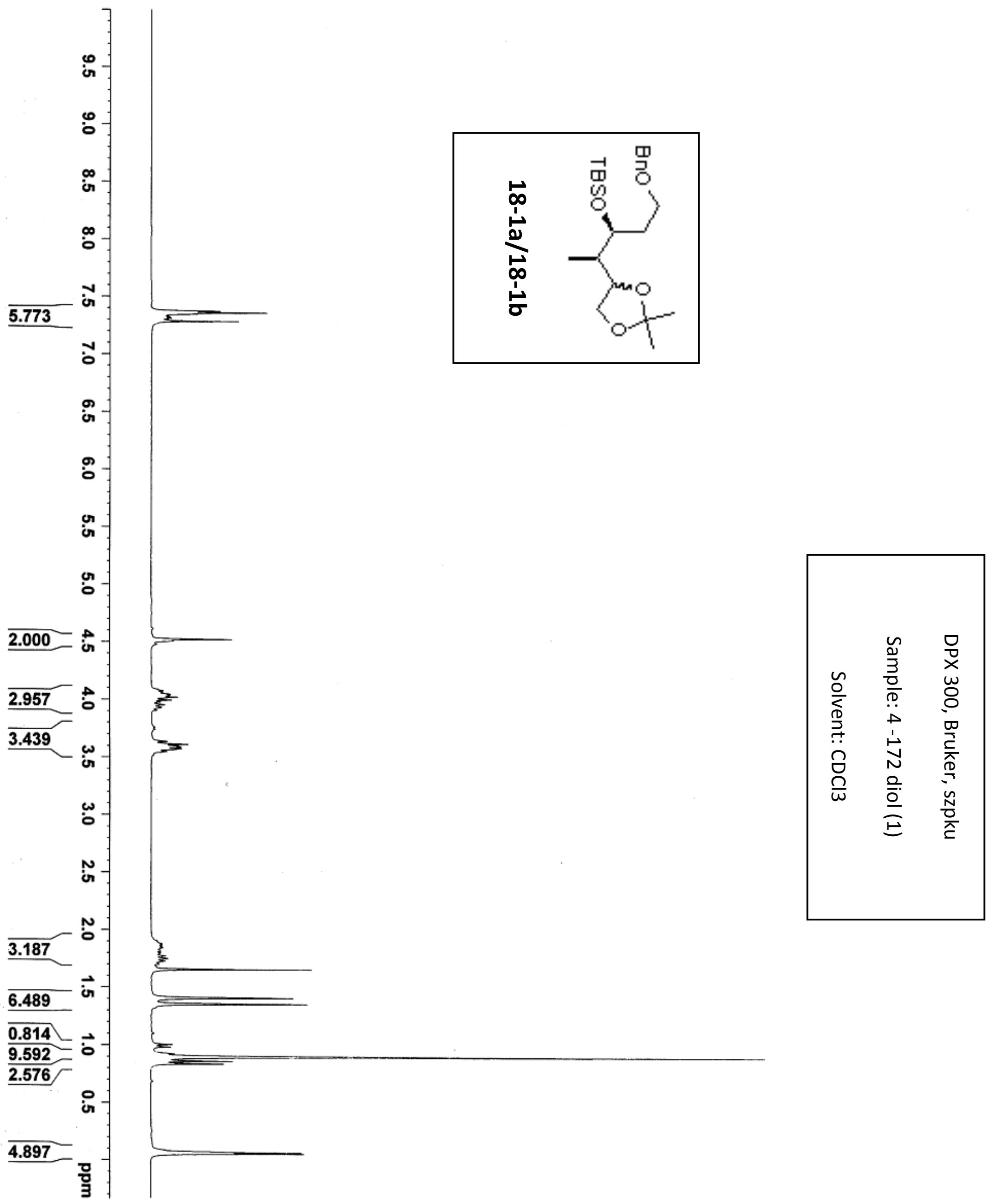
BnO
TBSO
18-1a/18-1b
DPX 300, Bruker, szpku
Sample: 4 -172 diol (1)
Solvent: CDCl3
ppm

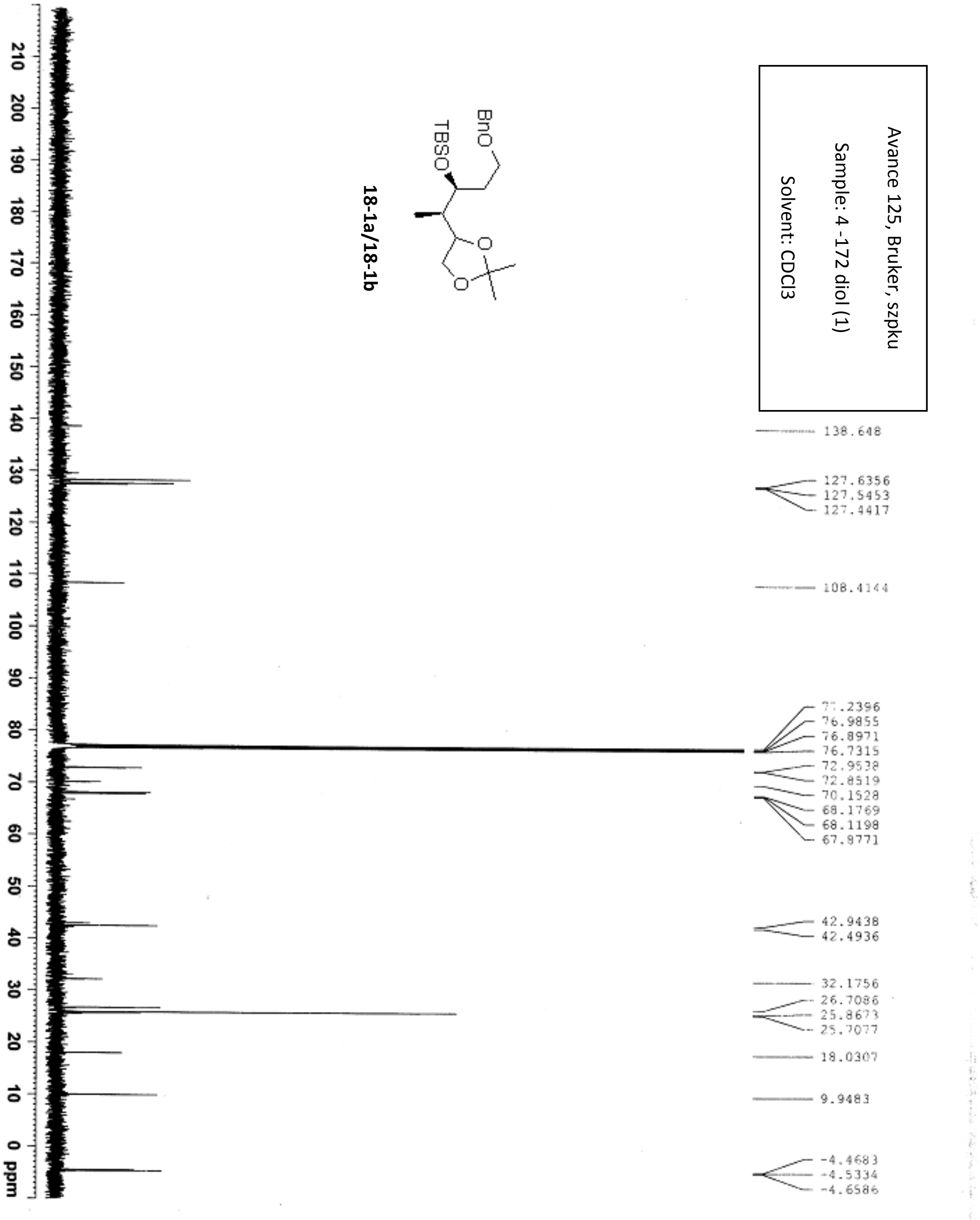
Avance 125, Bruker, szpku
Sample: 4 -172 diol (1)
Solvent: CDCl3
BnO
TBSO
18-1a/18-1b
138.648
127.6356
127.5453
127.4417
108.4144
76.9855
76.8971
76.7315
72.9538
72.8519
70.1528
68.1769
68.1198
67.9771
42.9438
42.4936
32.1756
26.7086
25.8673
25.7077
18.0307
9.9483
-4.4683
-4.5334
-4.6586
210
200
190
180
170
160
150
140
130
120
110
100
90
80
70
60
50
40
30
20
10
0 ppm

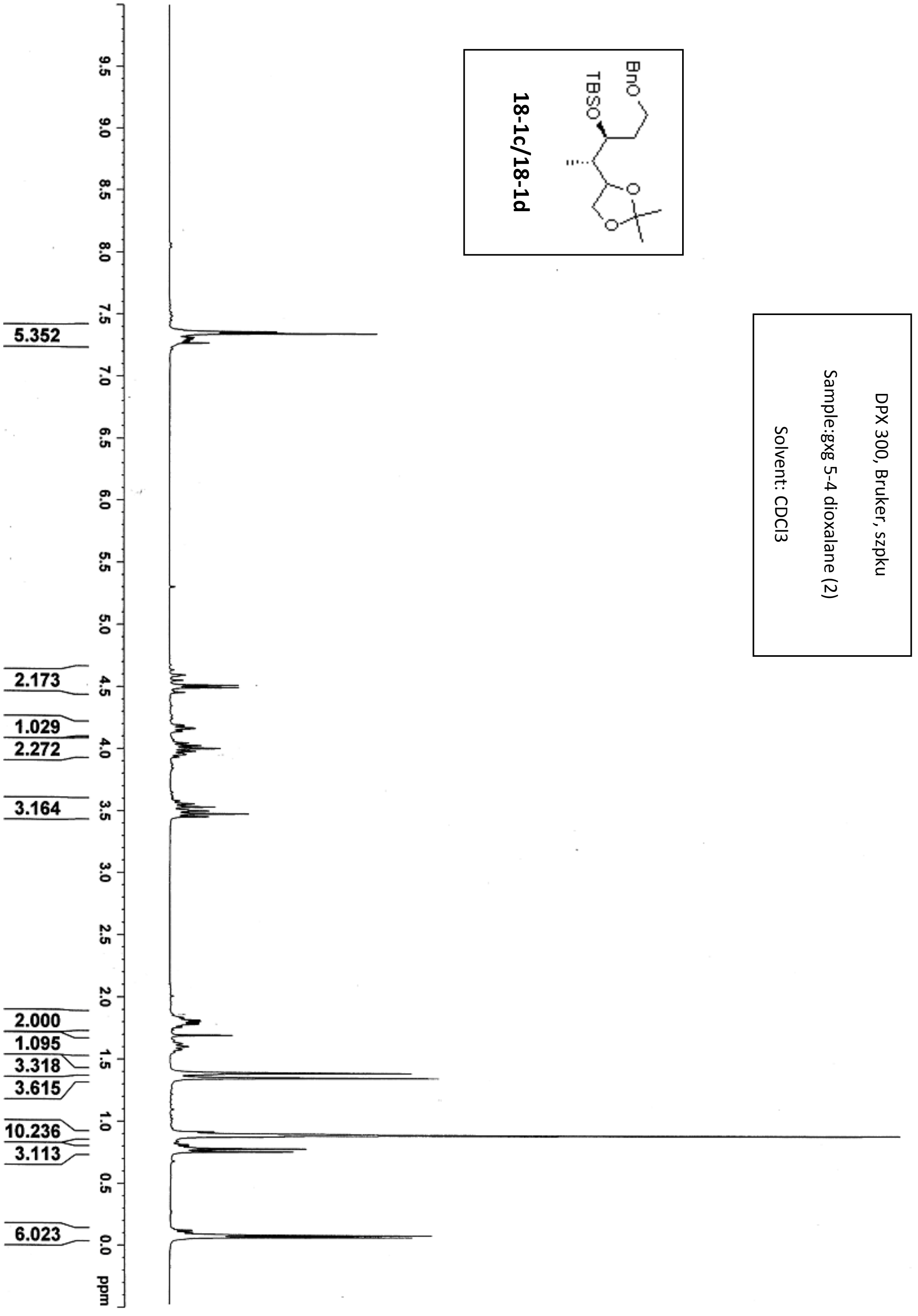
BnO
TBSO
18-1c/18-1d
DPX 300, Bruker, szpku
Sample:gxg 5-4 dioxalane (2)
Solvent: CDCl3
5.352
2.173
1.029
2.272
3.164
2.000
1.095
3.318
3.615
10.236
3.113
6.023
ppm

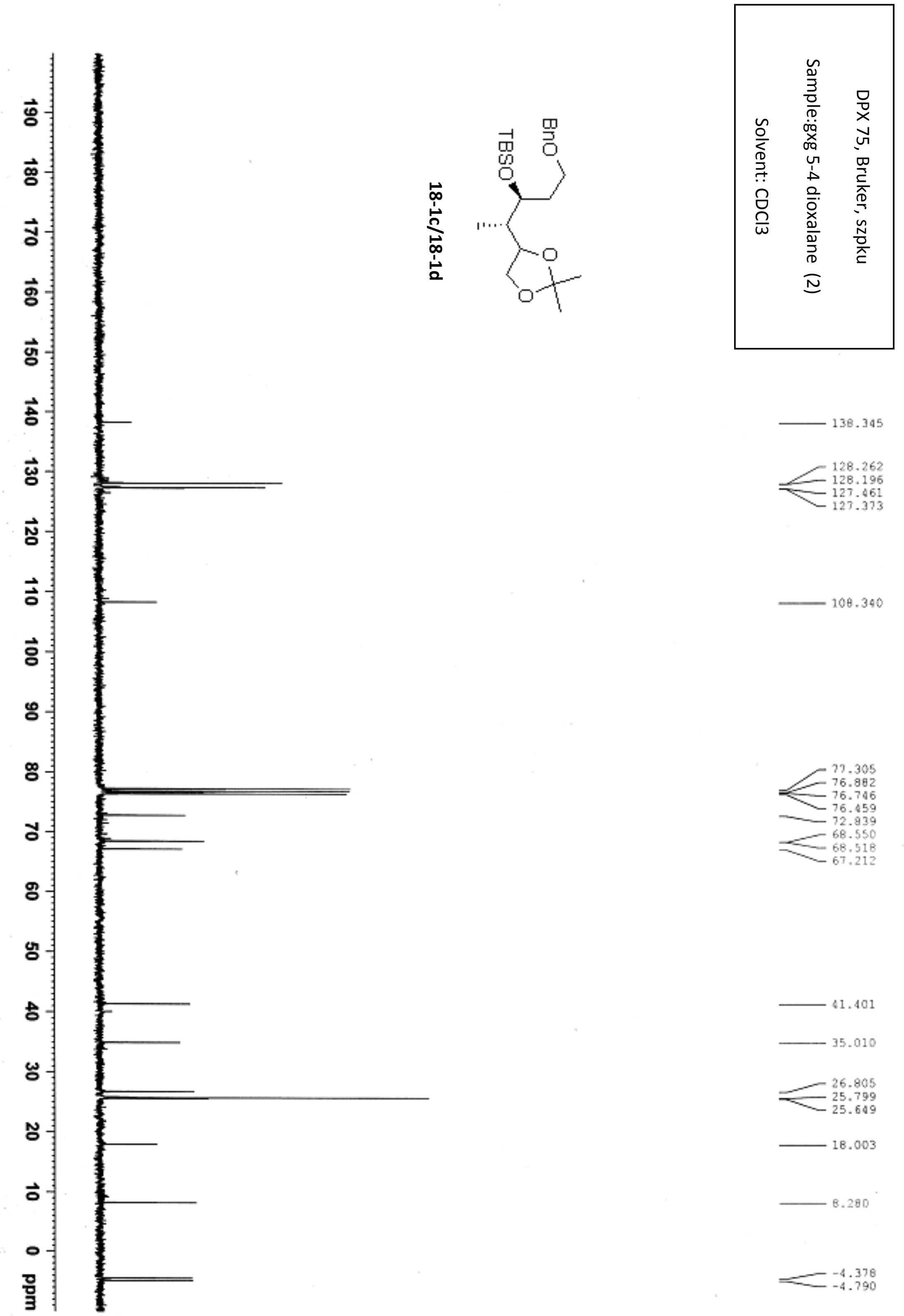
DPX 75, Bruker, szpku
Sample:gxg 5-4 dioxalane (2)
Solvent: CDCl3
BnO
TBSO
O
O
18-1c/18-1d
138.345
128.262
128.196
127.461
127.373
108.340
77.305
76.882
76.746
76.459
72.839
68.550
68.518
67.212
41.401
35.010
26.805
25.799
25.649
18.003
8.280
-4.378
-4.790
190
180
170
160
150
140
130
120
110
100
90
80
70
60
50
40
30
20
10
0
ppm

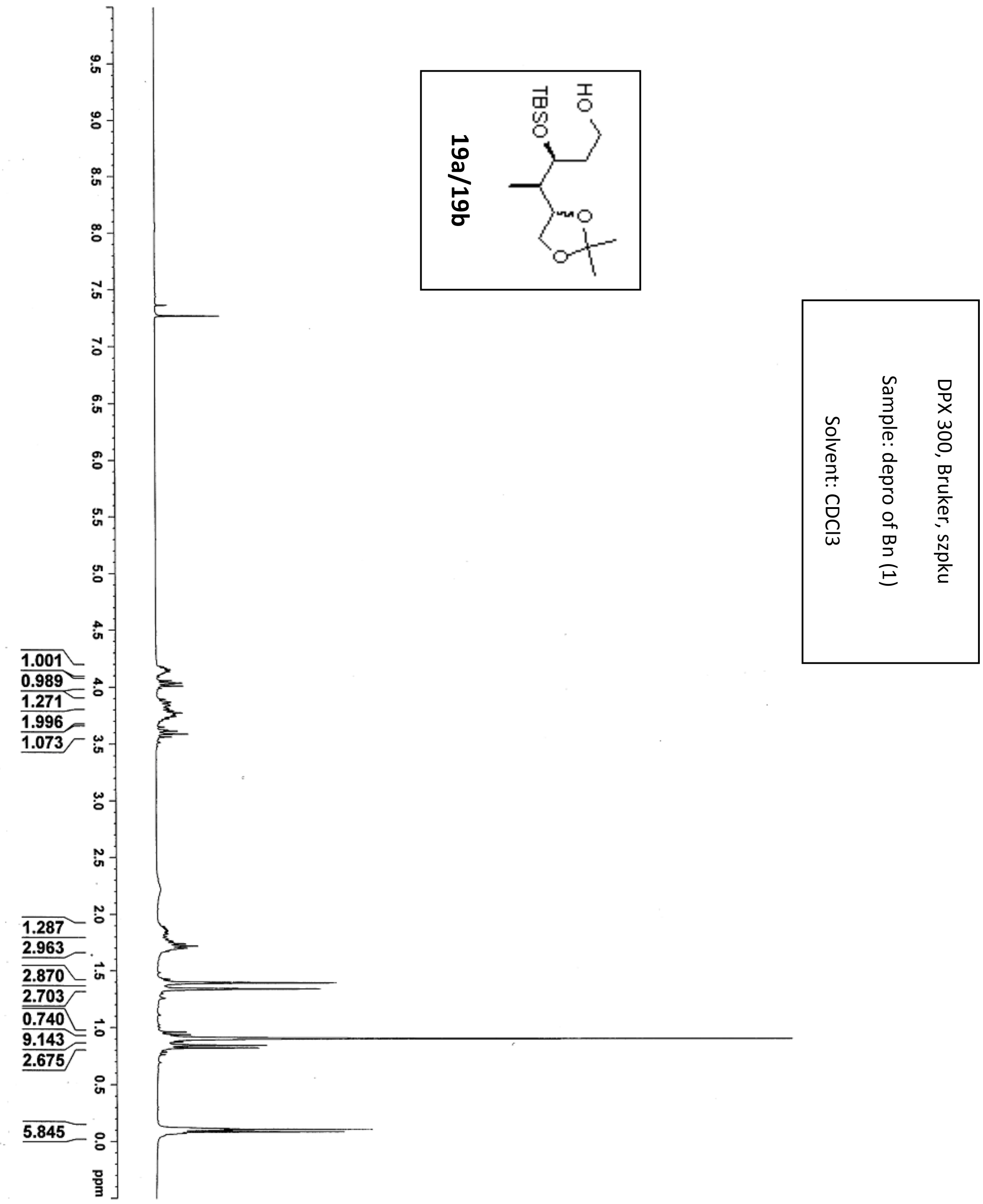
HO
TBSO
19a/19b
DPX 300, Bruker, szpku
Sample: depro of Bn (1)
Solvent: CDCl3
1.001
0.989
1.271
1.996
1.073
1.287
2.963
2.870
2.703
0.740
9.143
2.675
5.845
ppm

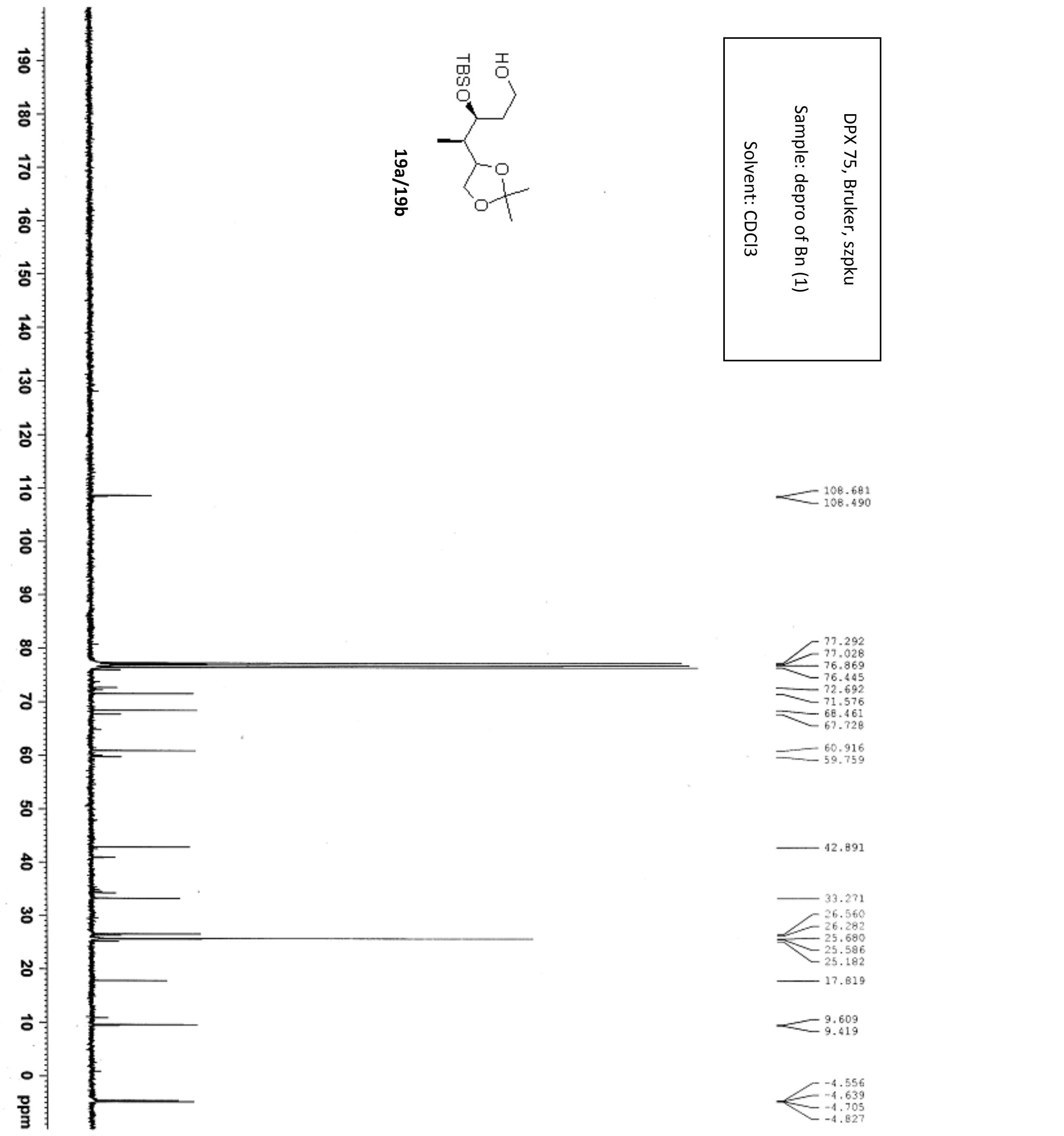
DPX 75, Bruker, szpku
Sample: depro of Bn (1)
Solvent: CDCl3
HO
TBSO
O
O
108.681
108.490
77.292
77.028
76.869
76.445
72.692
71.576
68.461
67.728
60.916
59.759
42.891
33.271
26.560
26.282
25.680
25.586
25.182
17.819
9.609
9.419
-4.556
-4.639
-4.705
-4.827
190 180 170 160 150 140 130 120 110 100 90 80 70 60 50 40 30 20 10 0 ppm

19a/19b

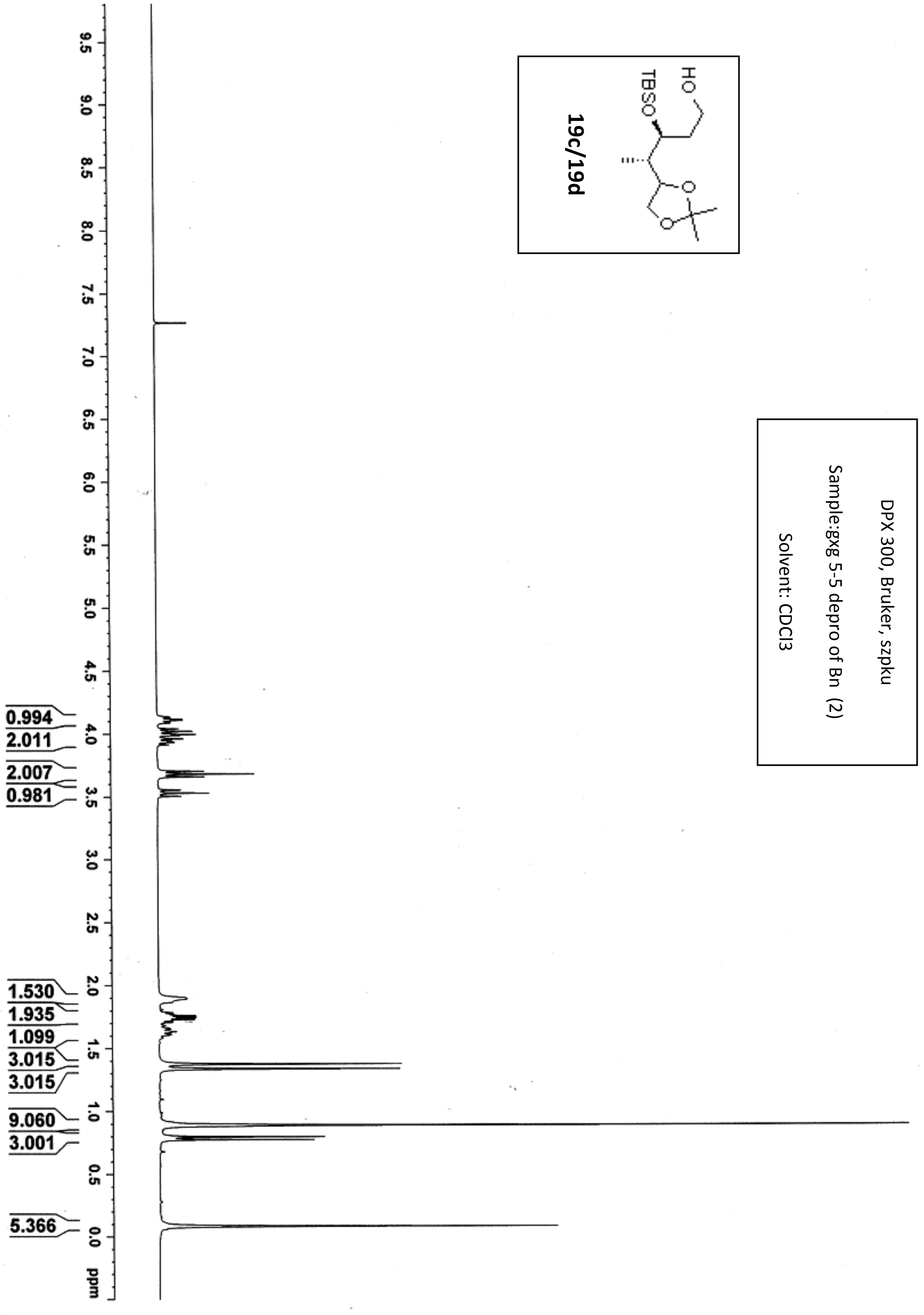
19c/19d
HO
TBSO
DPX 300, Bruker, szpku
Sample:gxg 5-5 depro of Bn (2)
Solvent: CDCl3
0.994
2.011
2.007
0.981
1.530
1.935
1.099
3.015
3.015
9.060
3.001
5.366
ppm

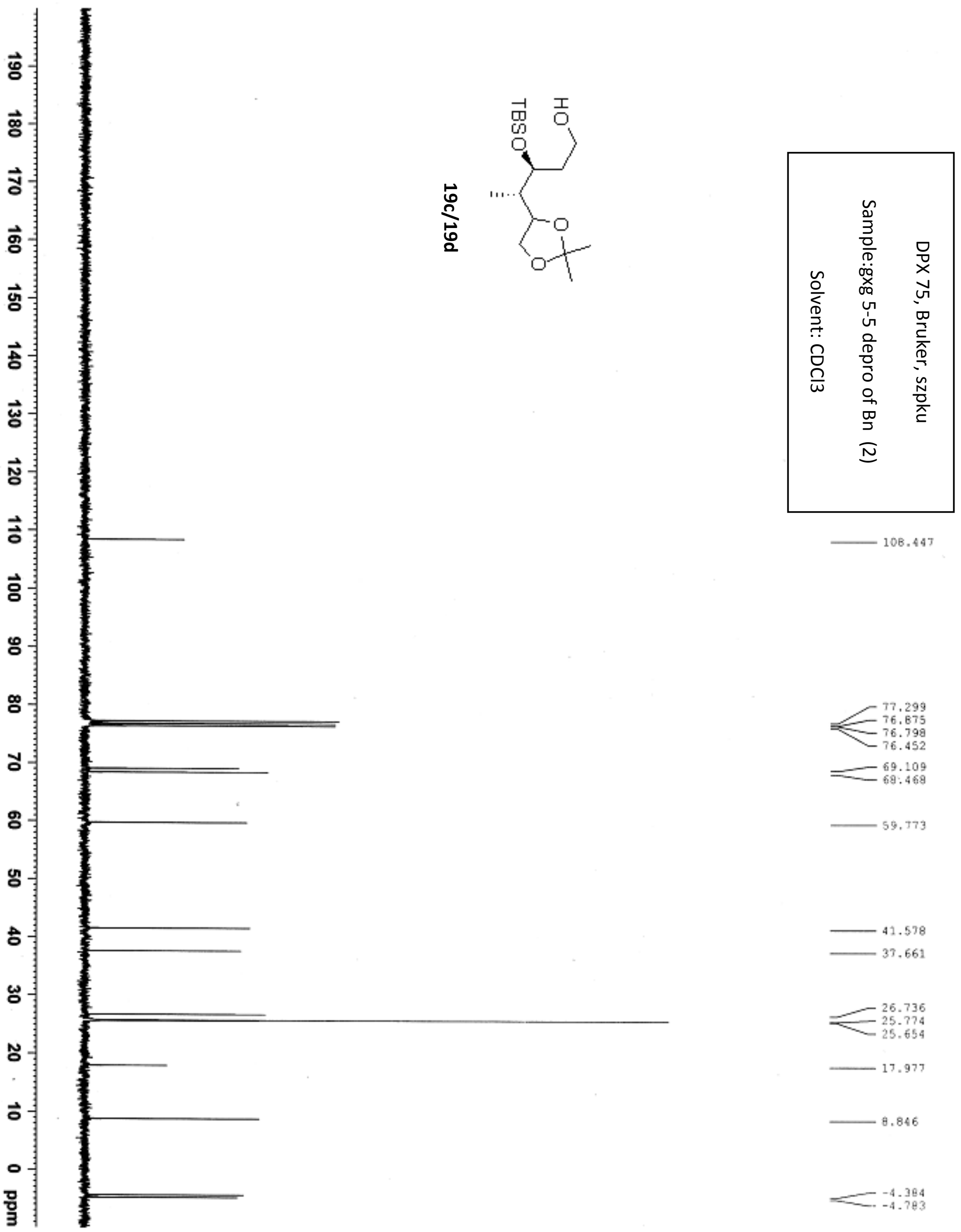
HO
TBSO
19c/19d
DPX 75, Bruker, szpku
Sample:gxg 5-5 depro of Bn (2)
Solvent: CDCl3
108.447
77.299
76.875
76.798
76.452
69.109
68.468
59.773
41.578
37.661
26.736
25.774
25.654
17.977
8.846
-4.384
-4.783
ppm

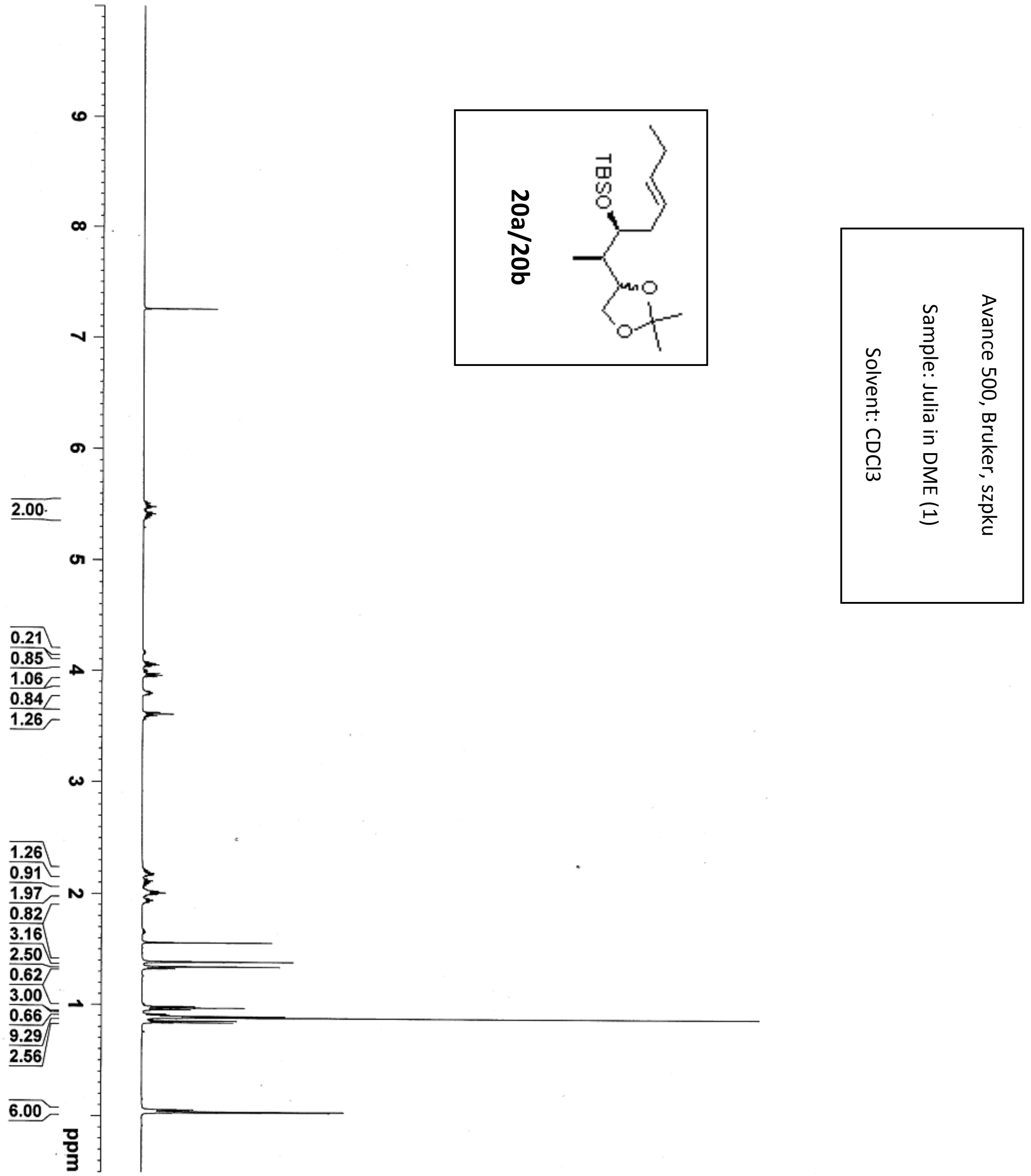
TBSO
20a/20b
Avance 500, Bruker, szpku
Sample: Julia in DME (1)
Solvent: CDCl3
ppm

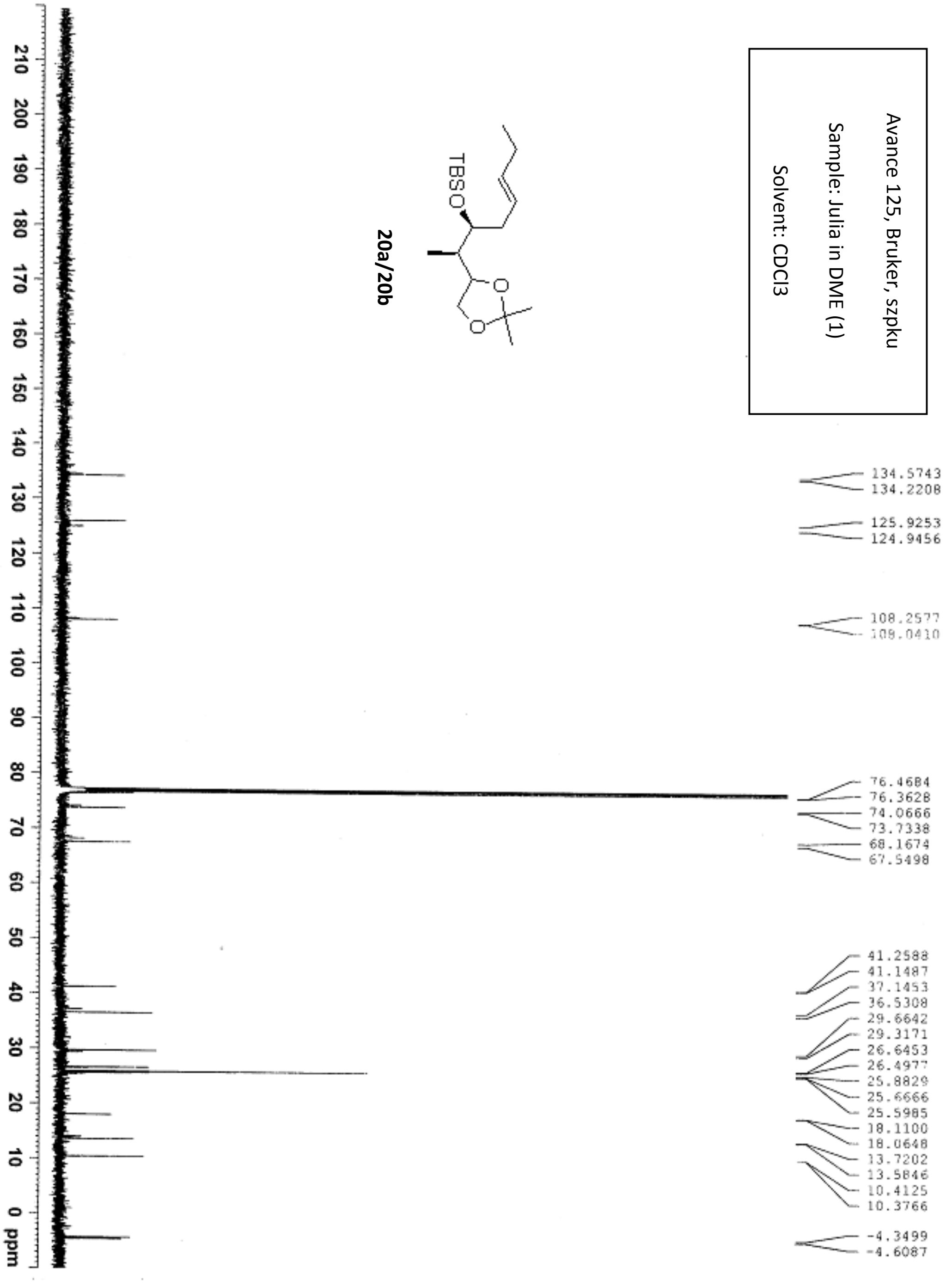
Avance 125, Bruker, szpku
Sample: Julia in DME (1)
Solvent: CDCl3
TBSO
20a/20b
134.5743
134.2208
125.9253
124.9456
108.2577
108.0410
76.4684
76.3628
74.0666
73.7338
68.1674
67.5498
41.2588
41.1487
37.1453
36.5308
29.6642
29.3171
26.6453
26.4977
25.8829
25.6666
25.5985
18.1100
18.0648
13.7202
13.5846
10.4125
10.3766
-4.3499
-4.6087
ppm

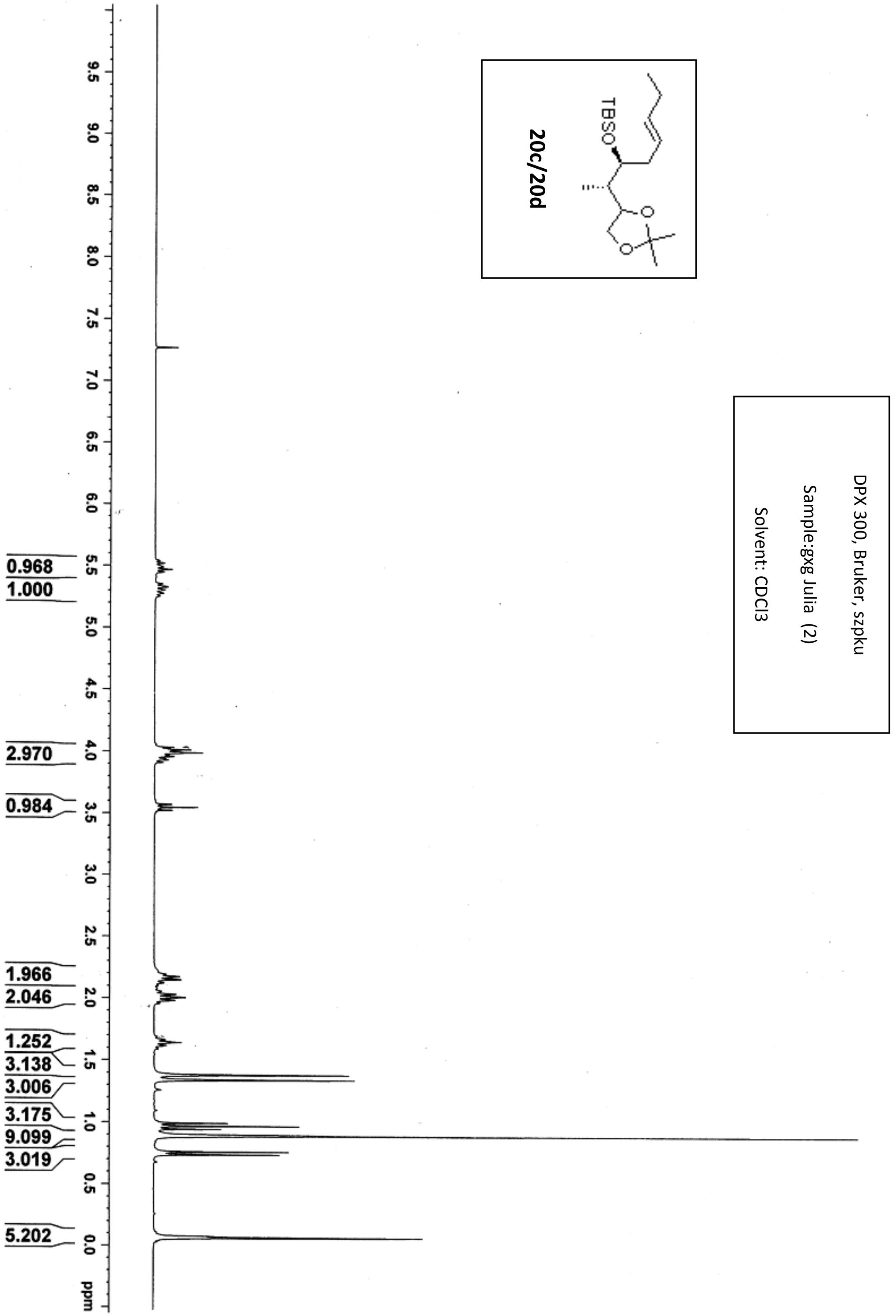
20c/20d
TBSO
DPX 300, Bruker, szpku
Sample:gxg Julia (2)
Solvent: CDCl3
0.968
1.000
2.970
0.984
1.966
2.046
1.252
3.138
3.006
3.175
9.099
3.019
5.202
ppm

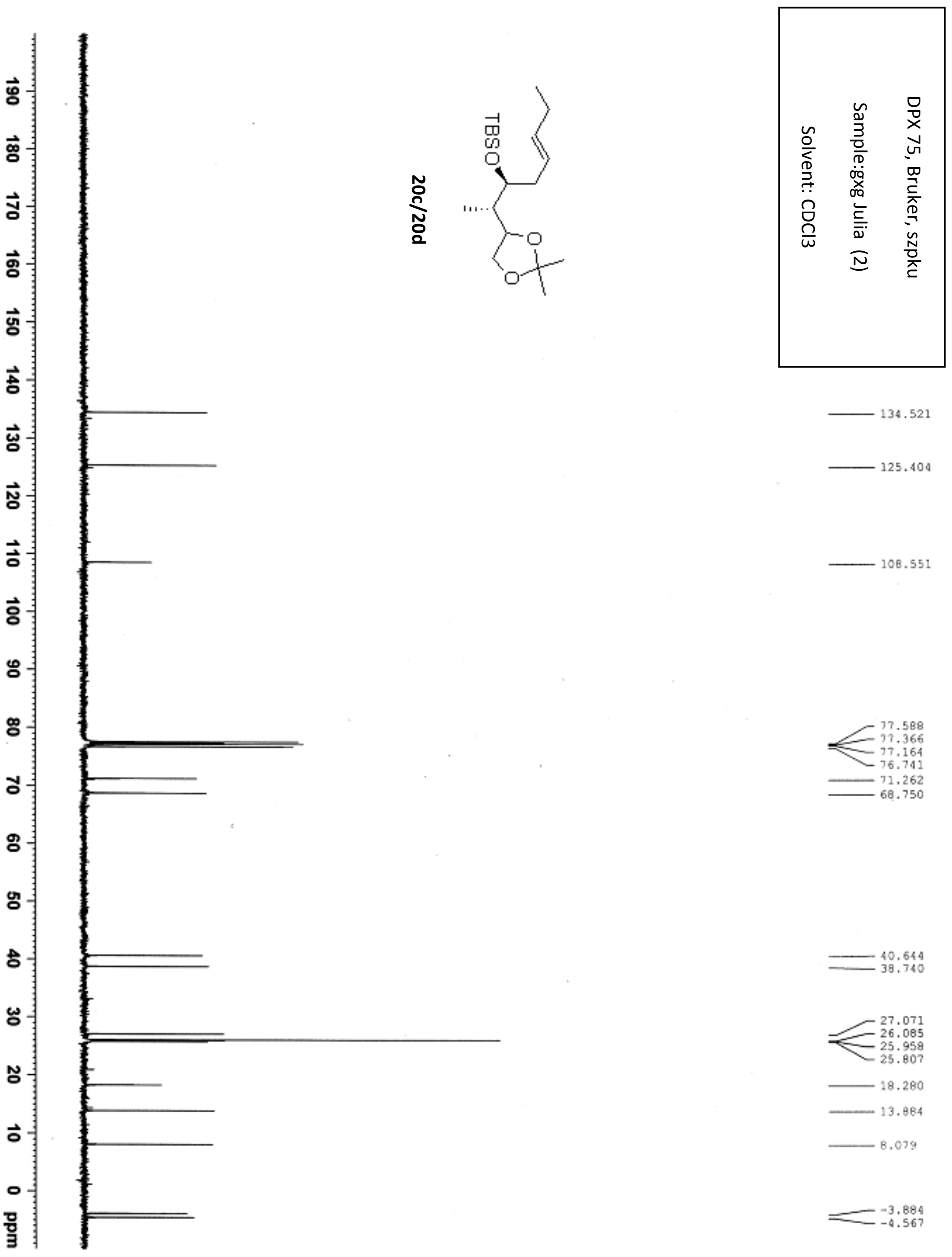
DPX 75, Bruker, szpku
Sample:gxg Julia (2)
Solvent: CDCl3
TBSO
20c/20d
134.521
125.404
108.551
77.588
77.366
77.164
76.741
71.262
68.750
40.644
38.740
27.071
26.085
25.958
25.807
18.280
13.884
8.079
-3.884
-4.567
ppm

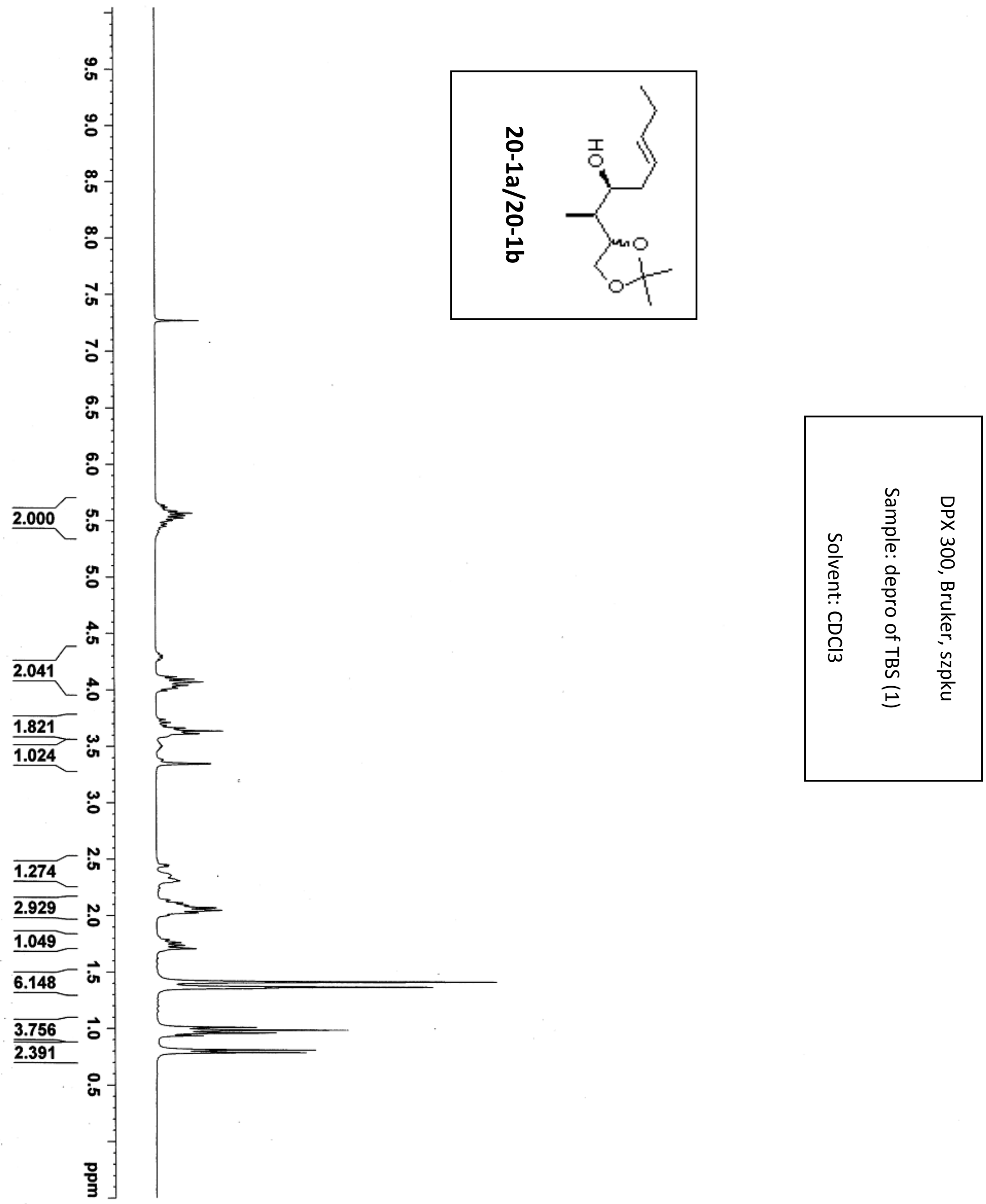
20-1a/20-1b
HO
DPX 300, Bruker, szpku
Sample: depro of TBS (1)
Solvent: CDCl3
2.000
2.041
1.821
1.024
1.274
2.929
1.049
6.148
3.756
2.391
9.5
9.0
8.5
8.0
7.5
7.0
6.5
6.0
5.5
5.0
4.5
4.0
3.5
3.0
2.5
2.0
1.5
1.0
0.5
ppm

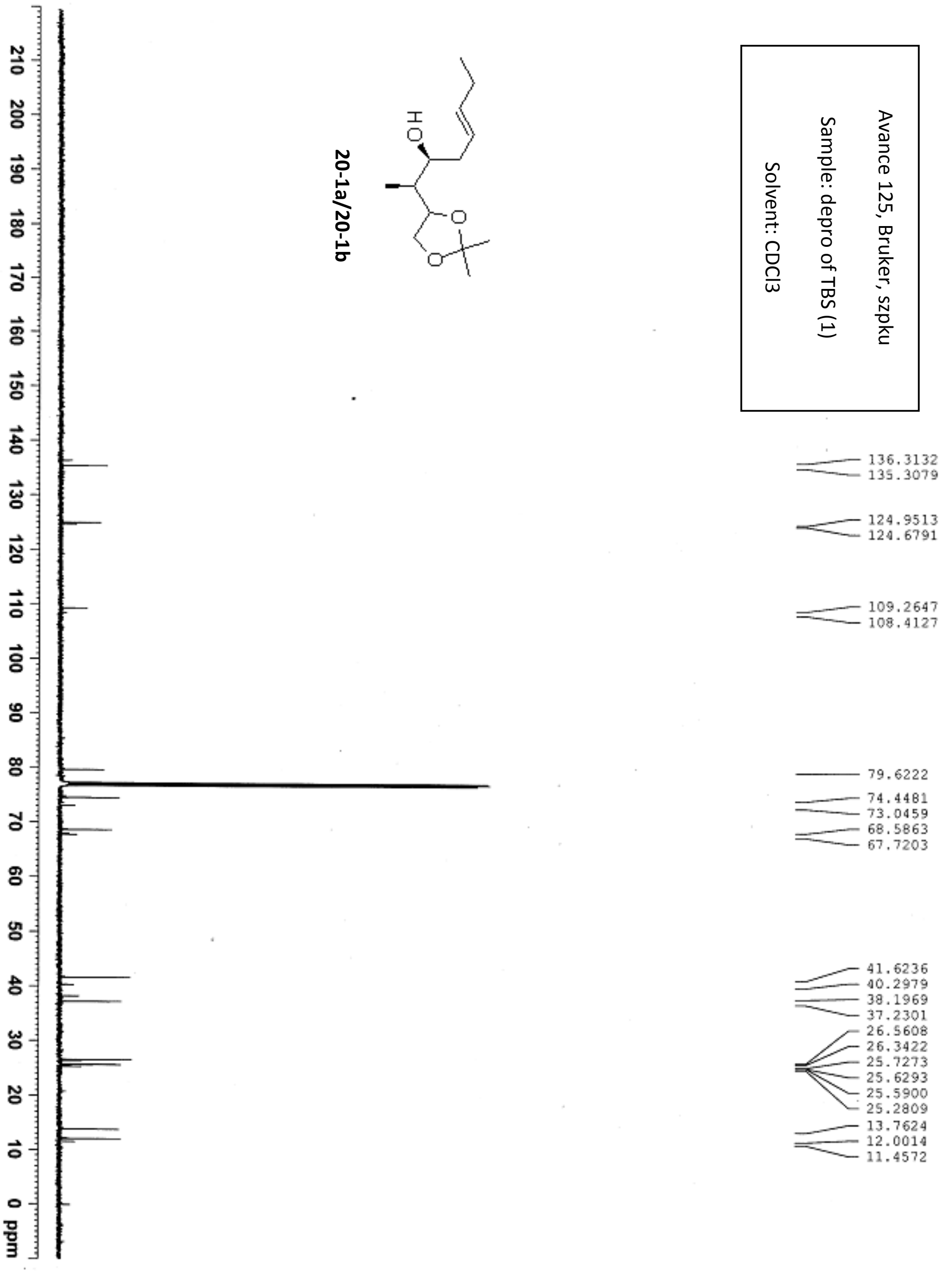
Avance 125, Bruker, szpku
Sample: depro of TBS (1)
Solvent: CDCl3
20-1a/20-1b
HO
136.3132
135.3079
124.9513
124.6791
109.2647
108.4127
79.6222
74.4481
73.0459
68.5863
67.7203
41.6236
40.2979
38.1969
37.2301
26.5608
26.3422
25.7273
25.6293
25.5900
25.2809
13.7624
12.0014
11.4572
210 200 190 180 170 160 150 140 130 120 110 100 90 80 70 60 50 40 30 20 10 0 ppm

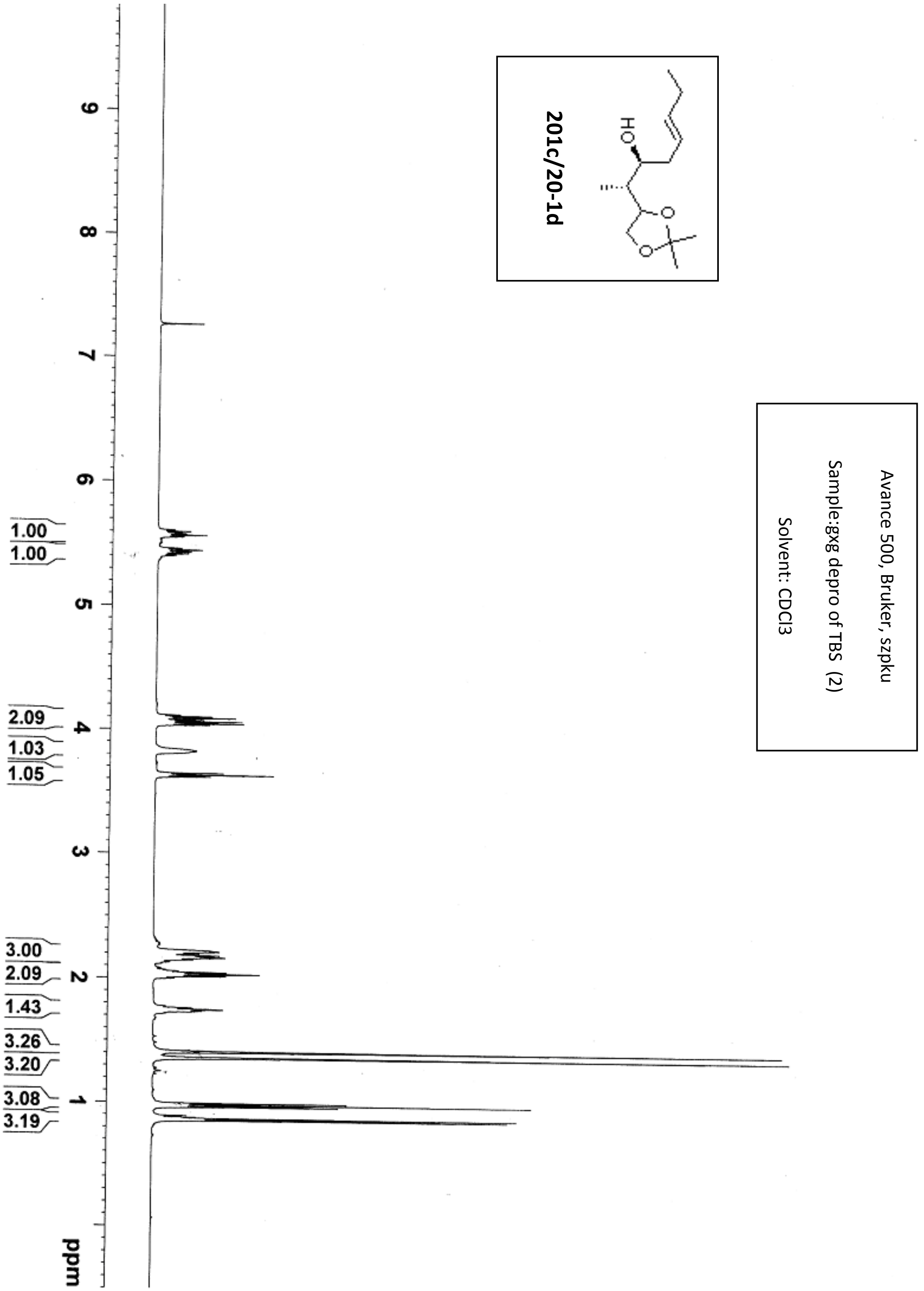
201c/20-1d
HO
Avance 500, Bruker, szpku
Sample:gxg depro of TBS (2)
Solvent: CDCl3
1.00
1.00
2.09
1.03
1.05
3.00
2.09
1.43
3.26
3.20
3.08
3.19
9
8
7
6
5
4
3
2
1
ppm

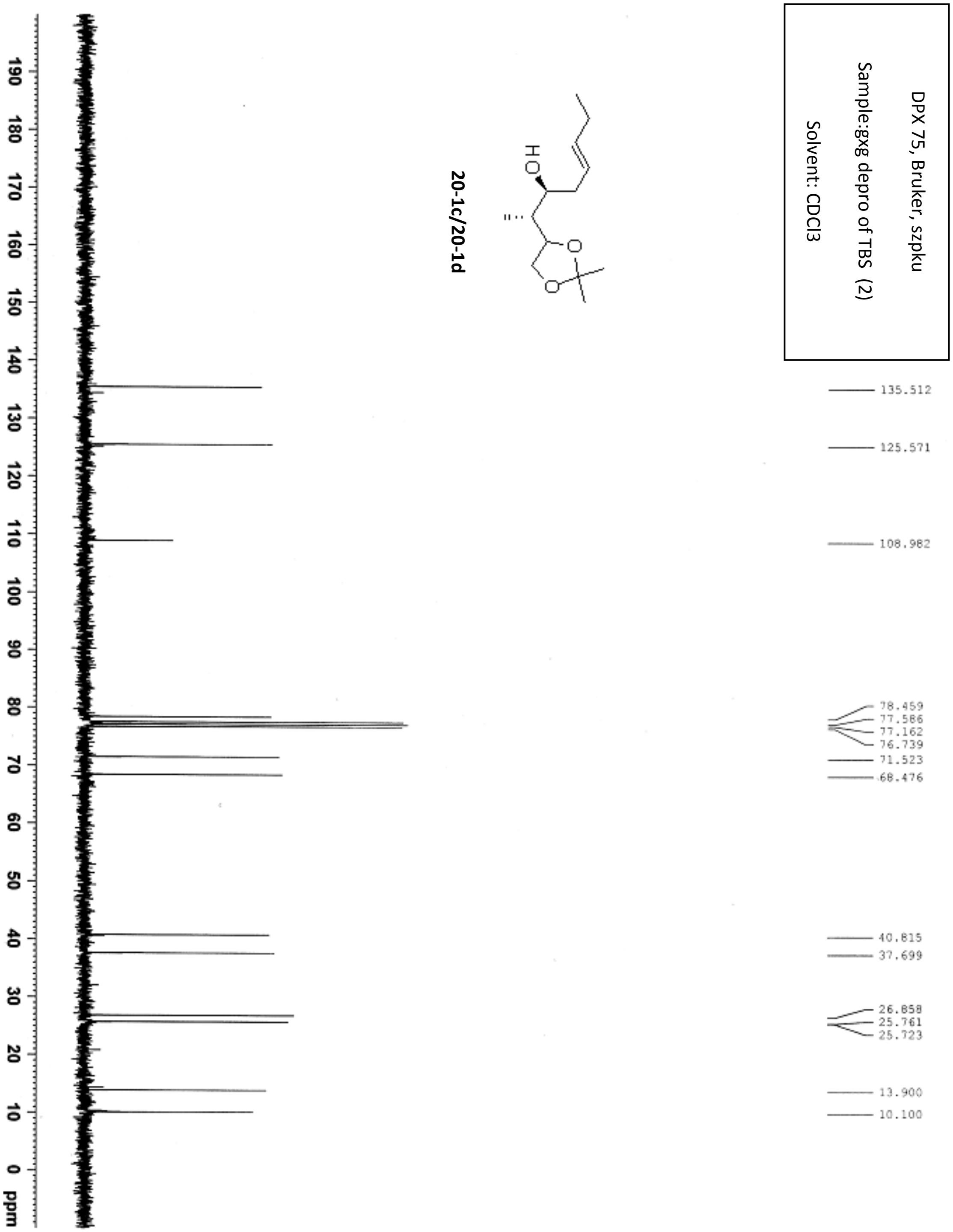
DPX 75, Bruker, szpku
Sample:gxg depro of TBS (2)
Solvent: CDCl3
20-1c/20-1d
HO
O
O
135.512
125.571
108.982
78.459
77.586
77.162
76.739
71.523
68.476
40.815
37.699
26.858
25.761
25.723
13.900
10.100
190 180 170 160 150 140 130 120 110 100 90 80 70 60 50 40 30 20 10 0 ppm

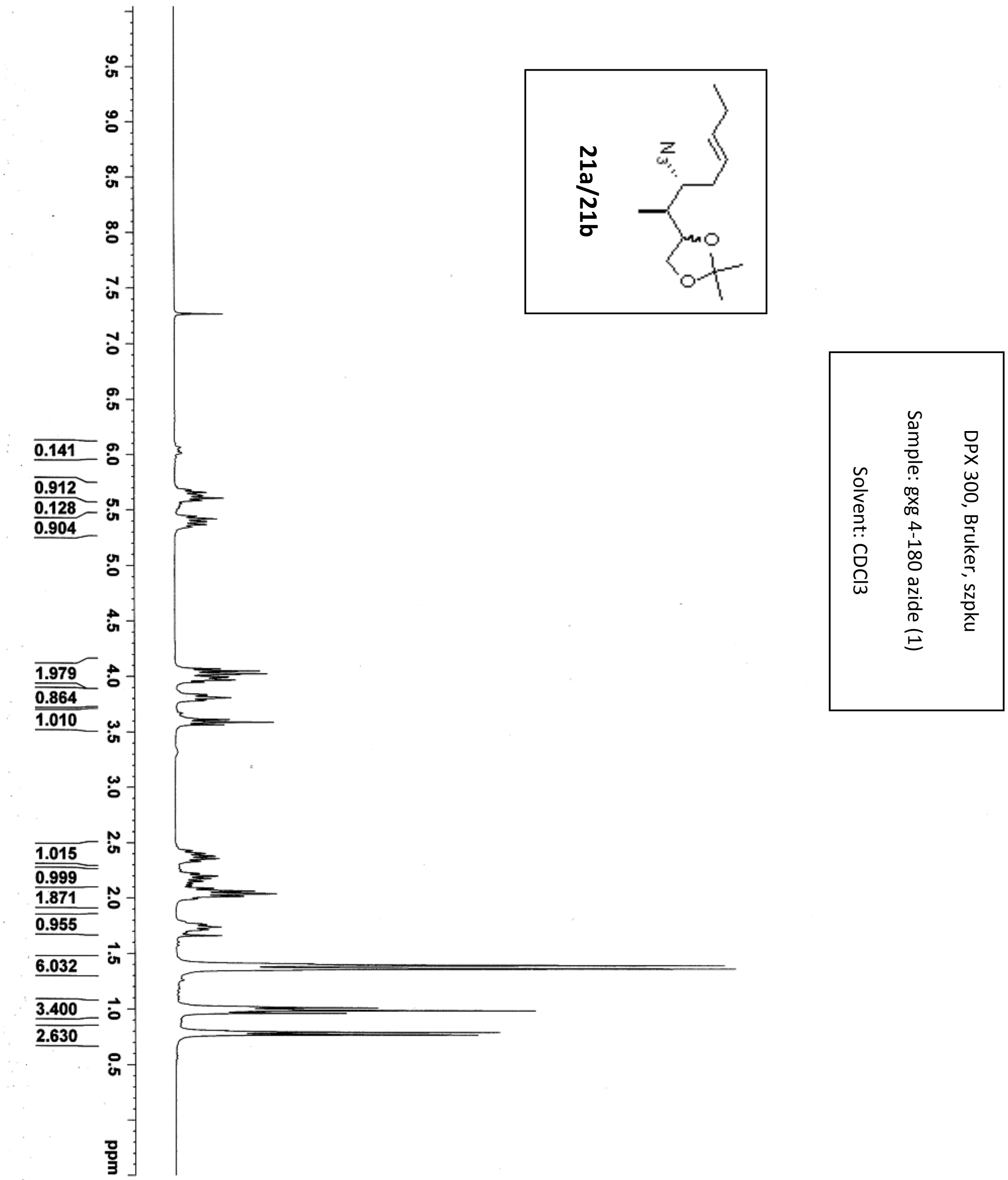
21a/21b
DPX 300, Bruker, szpku
Sample: gxg 4-180 azide (1)
Solvent: CDCl3
9.5 9.0 8.5 8.0 7.5 7.0 6.5 6.0 5.5 5.0 4.5 4.0 3.5 3.0 2.5 2.0 1.5 1.0 0.5 ppm
0.141
0.912
0.128
0.904
1.979
0.864
1.010
1.015
0.999
1.871
0.955
6.032
3.400
2.630

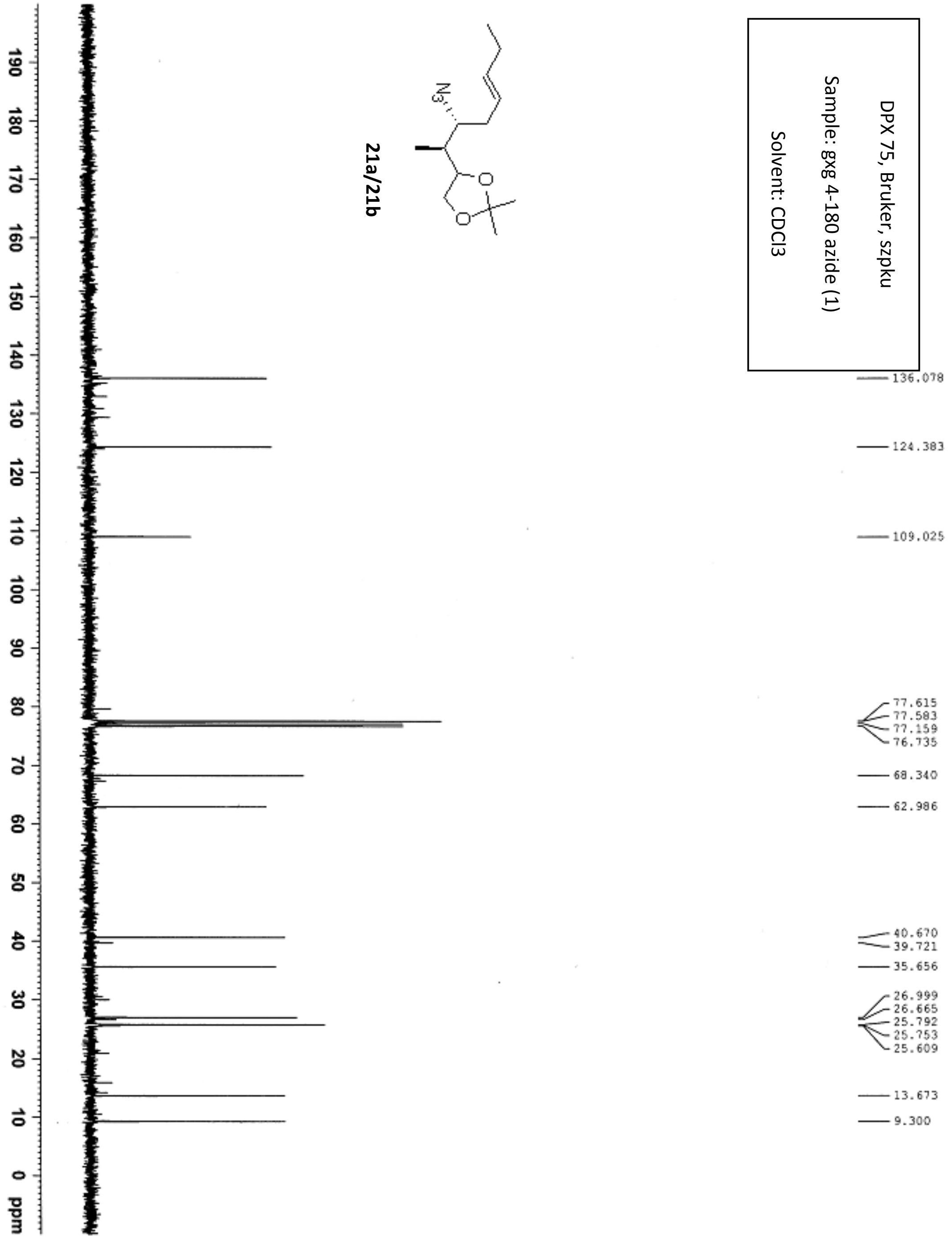
DPX 75, Bruker, szpku
Sample: gxg 4-180 azide (1)
Solvent: CDCl3
21a/21b
136.078
124.383
109.025
77.615
77.583
77.159
76.735
68.340
62.986
40.670
39.721
35.656
26.999
26.665
25.792
25.753
25.609
13.673
9.300
ppm

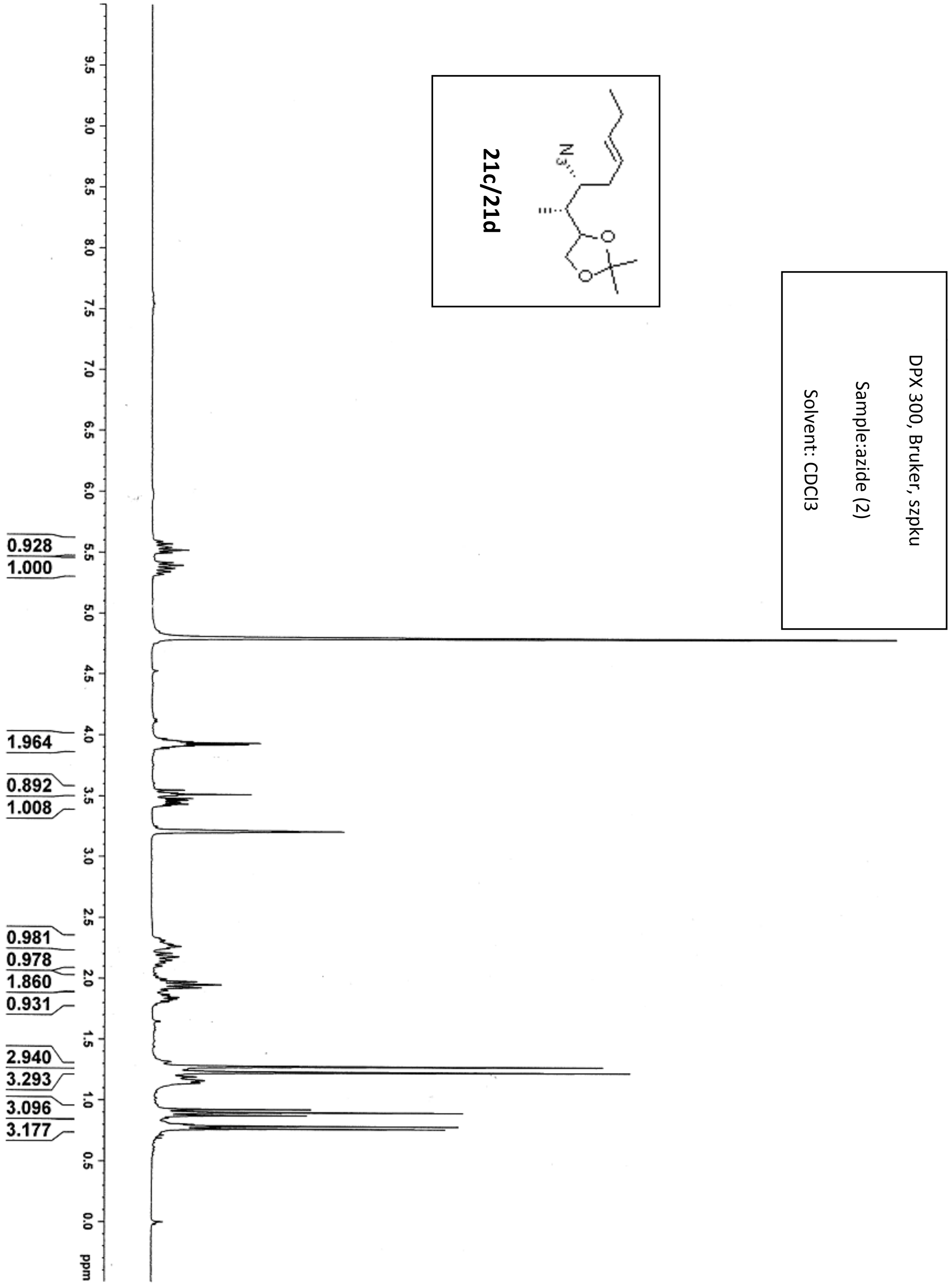
21c/21d
N3
DPX 300, Bruker, szpku
Sample:azide (2)
Solvent: CDCl3
0.928
1.000
1.964
0.892
1.008
0.981
0.978
1.860
0.931
2.940
3.293
3.096
3.177
ppm

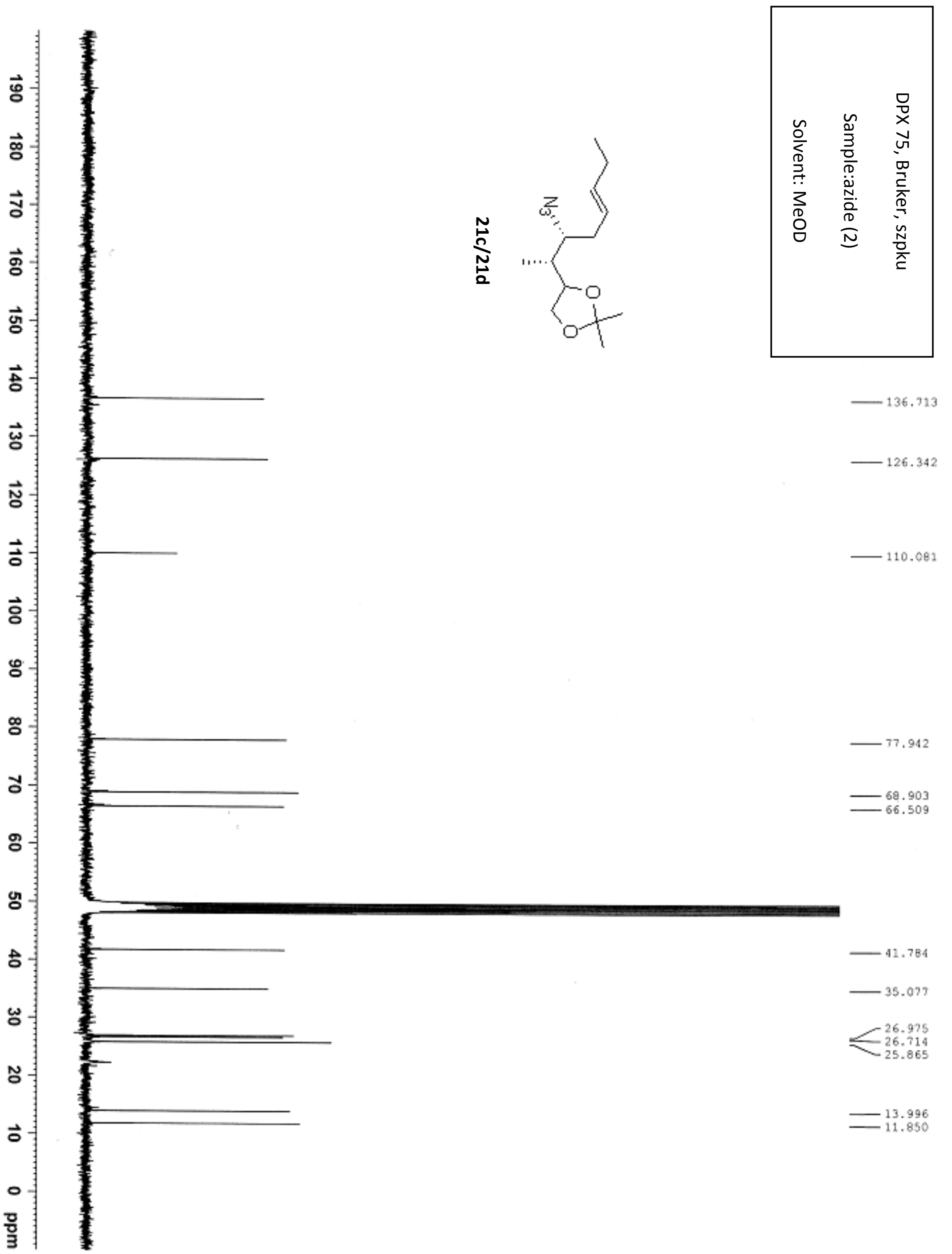
DPX 75, Bruker, szpku
Sample:azide (2)
Solvent: MeOD
N_3
21c/21d
136.713
126.342
110.081
77.942
68.903
66.509
41.784
35.077
26.975
26.714
25.865
13.996
11.850
ppm

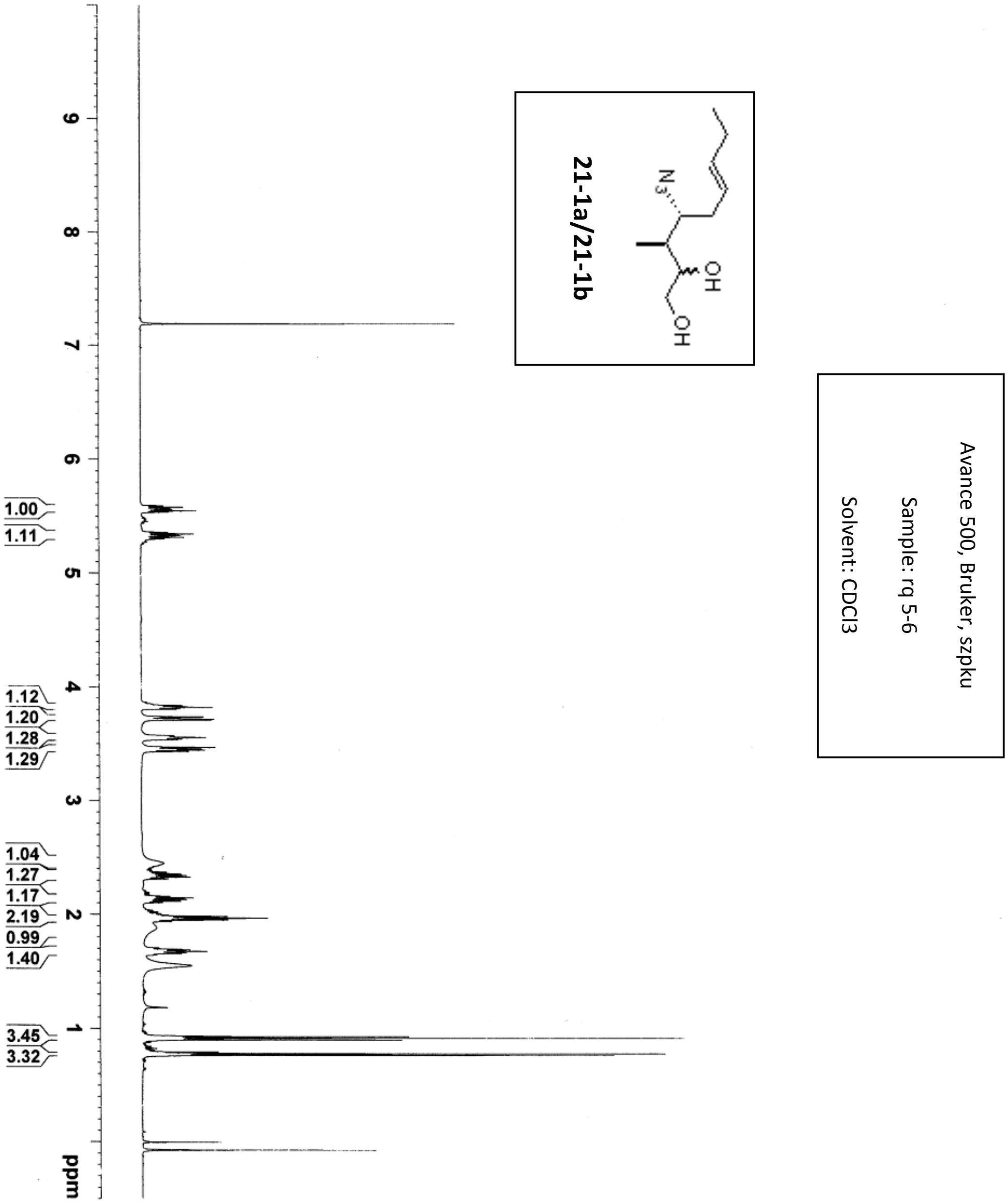
21-1a/21-1b
Avance 500, Bruker, szpku
Sample: rq 5-6
Solvent: CDCl3

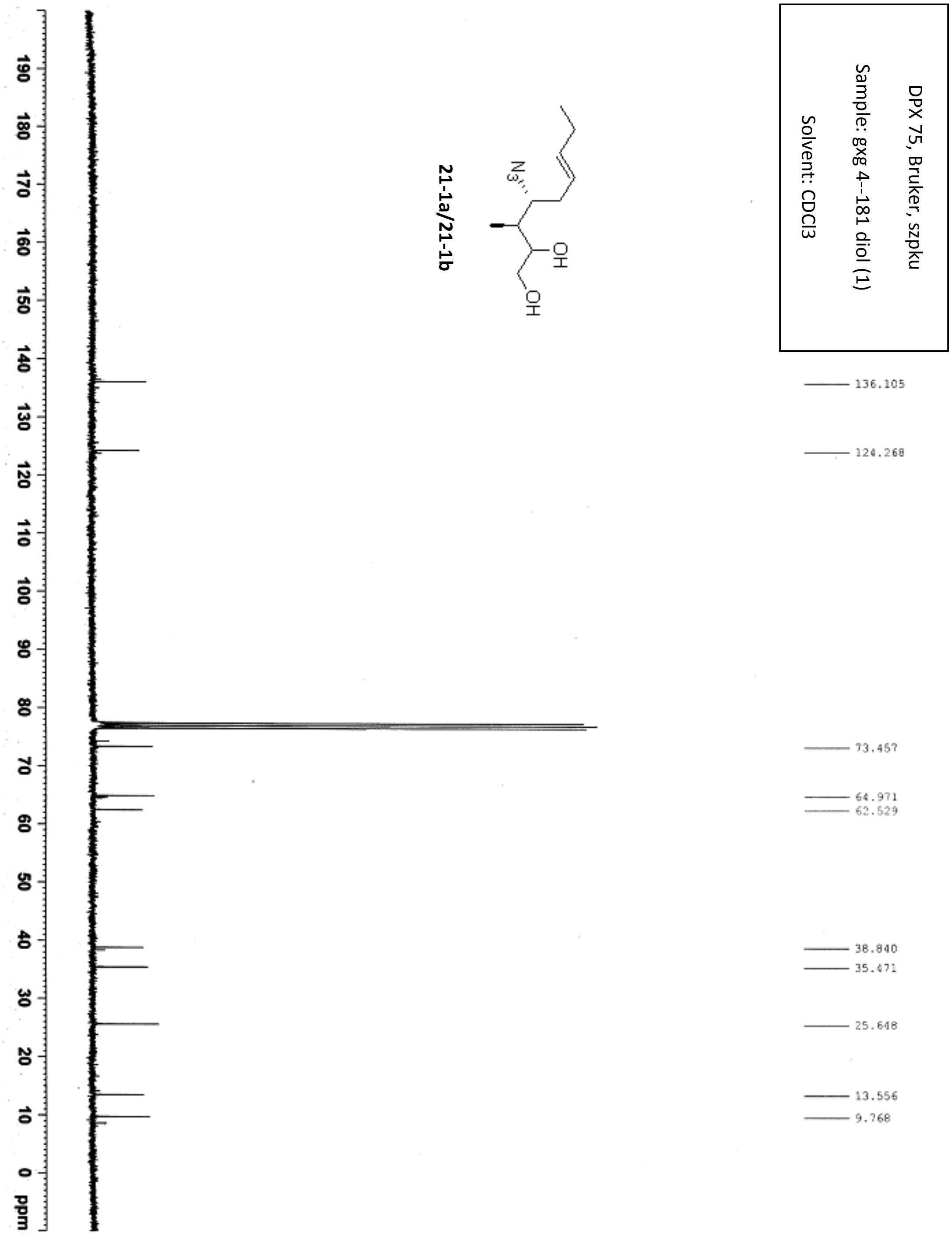
DPX 75, Bruker, szpku
Sample: gxg 4--181 diol (1)
Solvent: CDCl3
21-1a/21-1b
OH
OH
N3
136.105
124.268
73.457
64.971
62.529
38.840
35.471
25.648
13.556
9.768
ppm

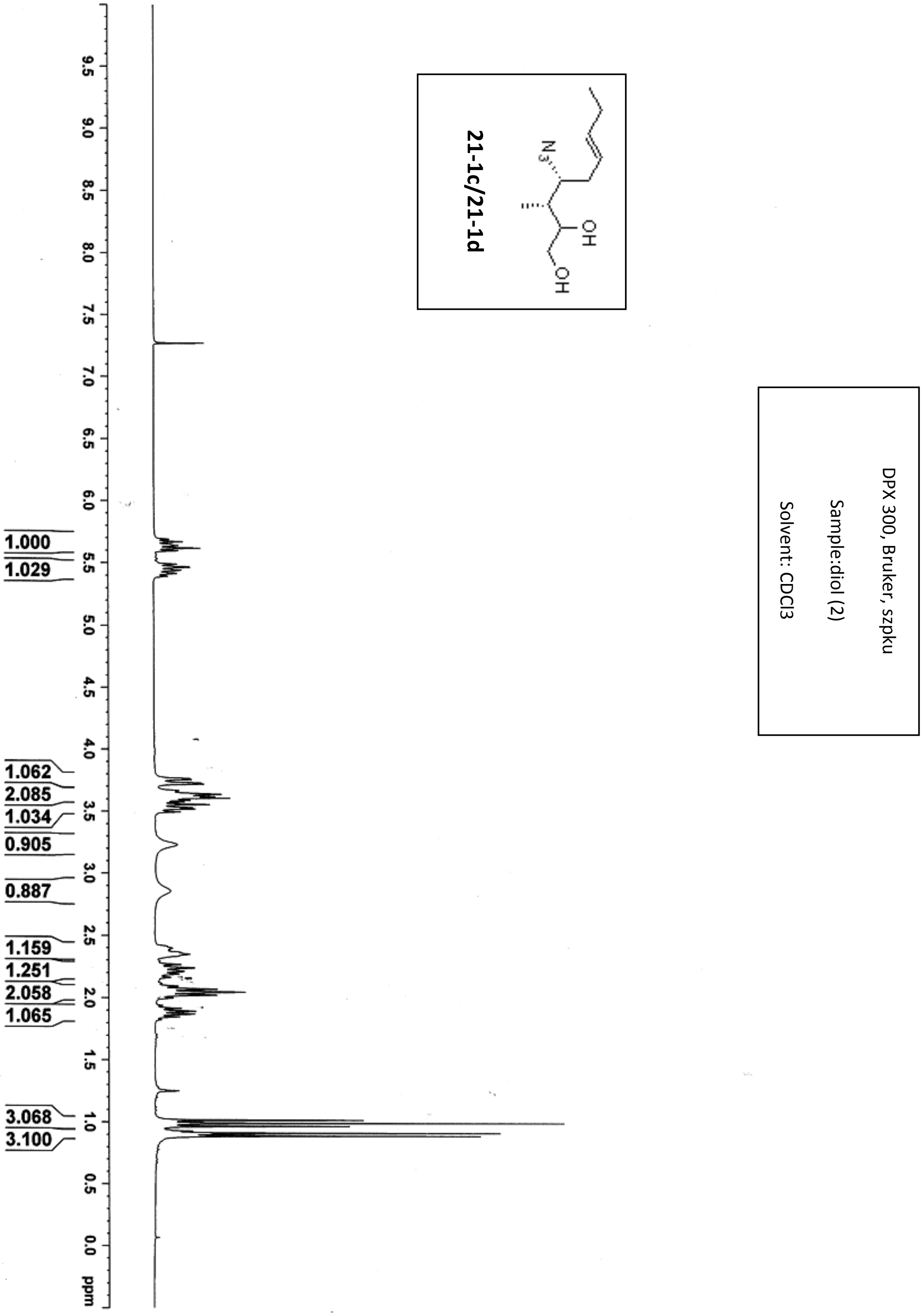
21-1c/21-1d
DPX 300, Bruker, szpku
Sample:diol (2)
Solvent: CDCl3
ppm

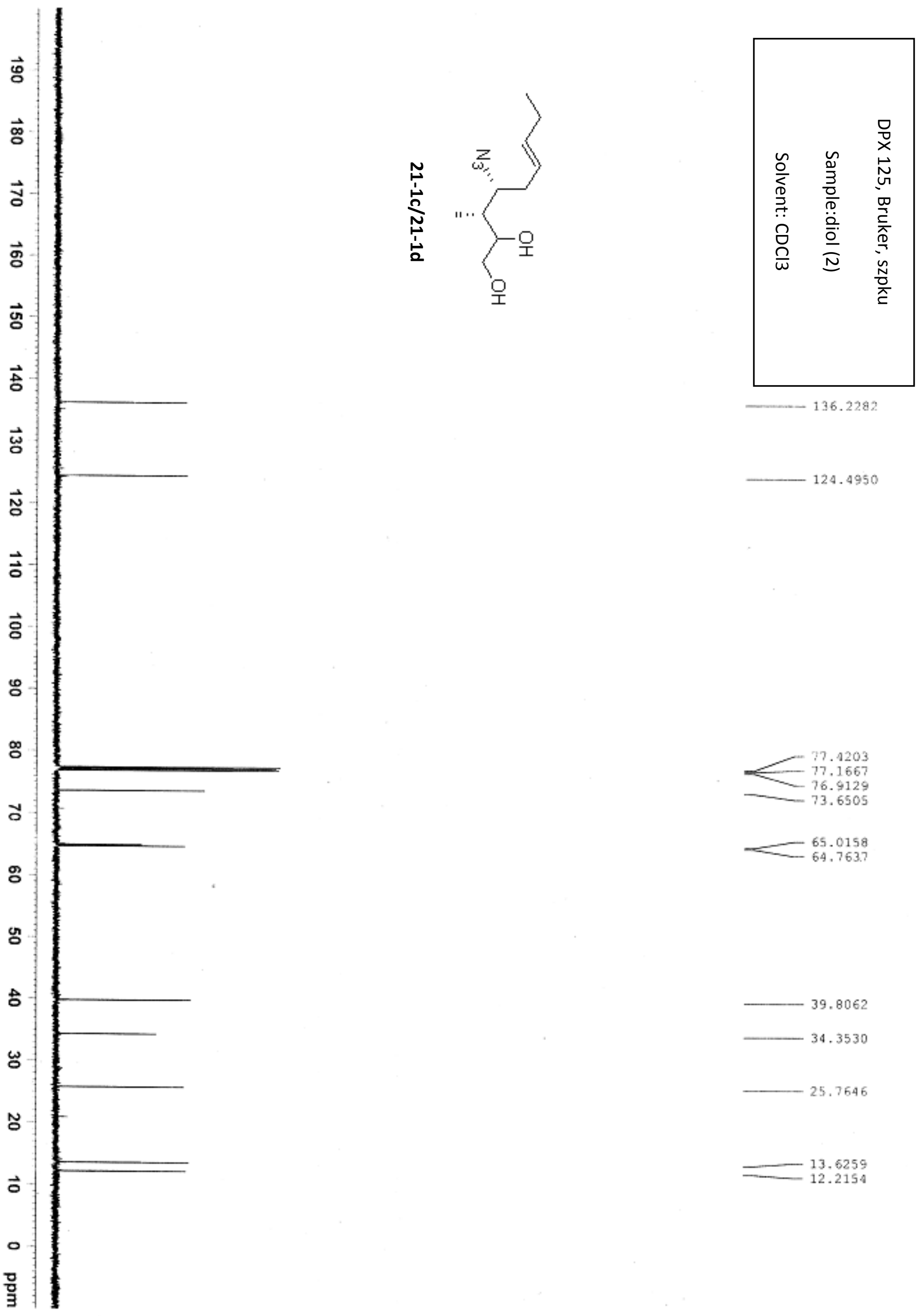
DPX 125, Bruker, szpku
Sample:diol (2)
Solvent: CDCl3
21-1c/21-1d
136.2282
124.4950
77.4203
77.1667
76.9129
73.6505
65.0158
64.7637
39.8062
34.3530
25.7646
13.6259
12.2154
ppm

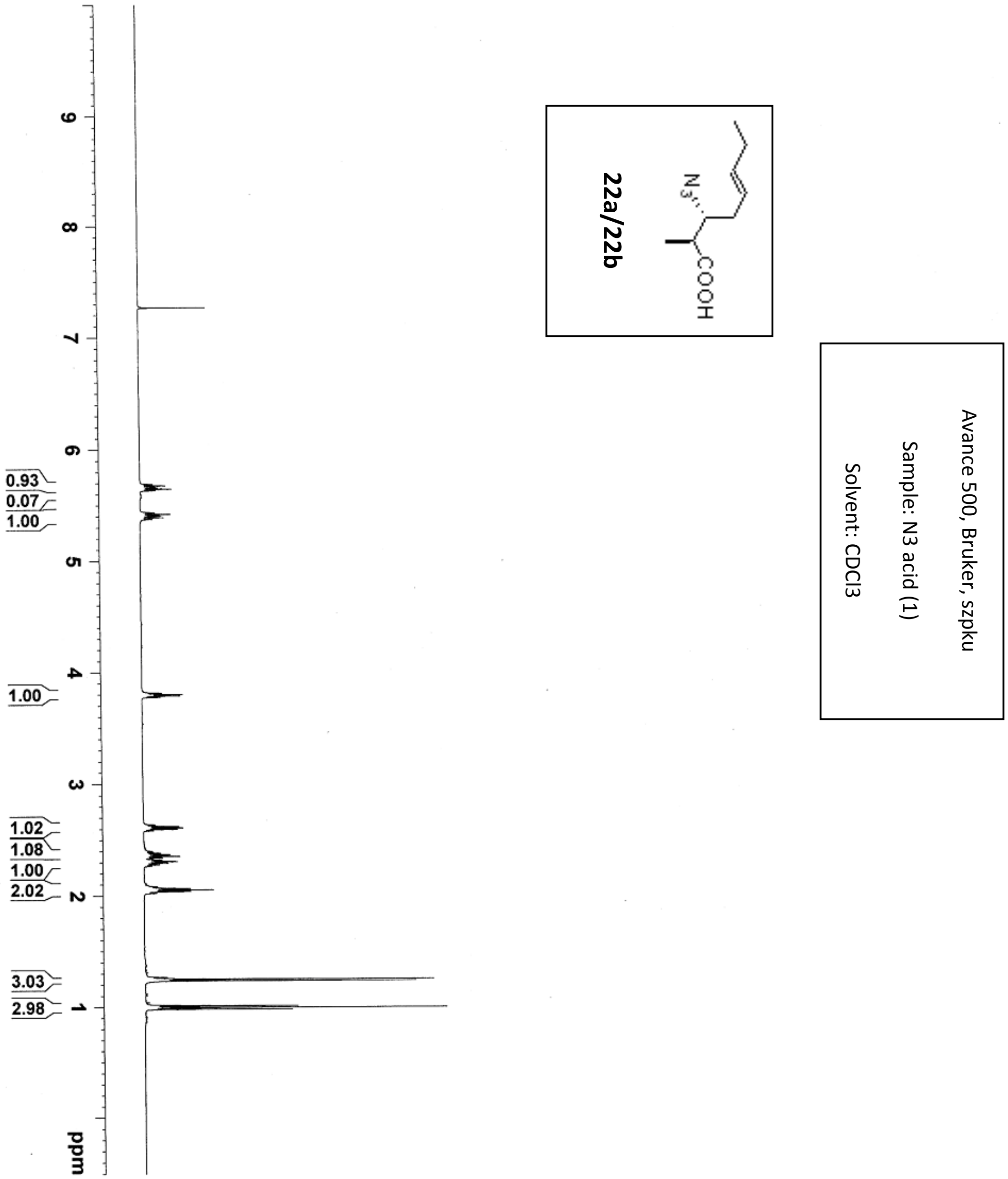
22a/22b
N_3
COOH
Avance 500, Bruker, szpku
Sample: N3 acid (1)
Solvent: CDCl3
9
8
7
6
5
4
3
2
1
ppm
0.93
0.07
1.00
1.00
1.02
1.08
1.00
2.02
3.03
2.98

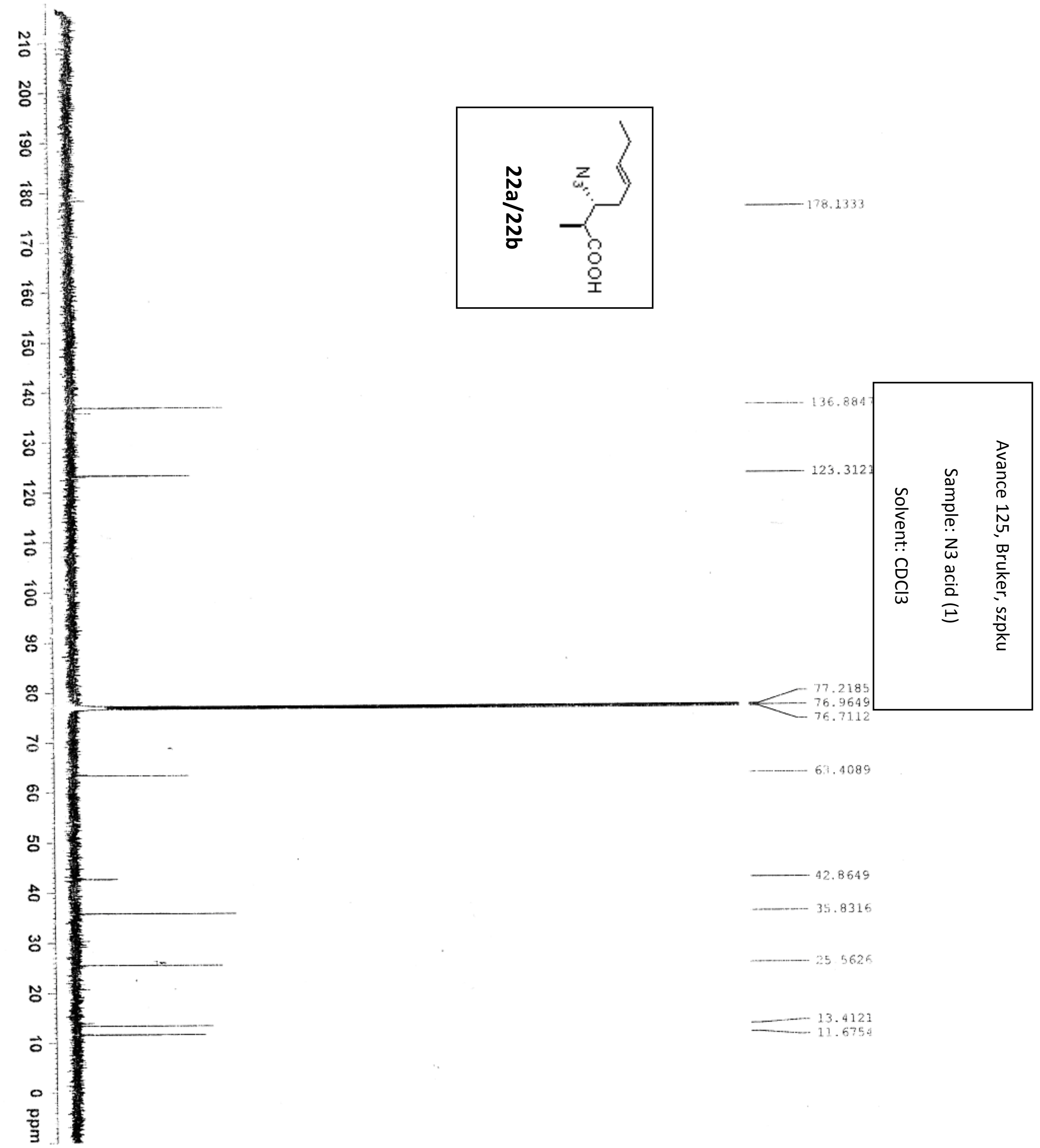
22a/22b
COOH
Avance 125, Bruker, szpku
Sample: N3 acid (1)
Solvent: CDCl3
178.1333
136.8847
123.3121
77.2185
76.9649
76.7112
63.4089
42.8649
35.8316
25.5626
13.4121
11.6754
ppm

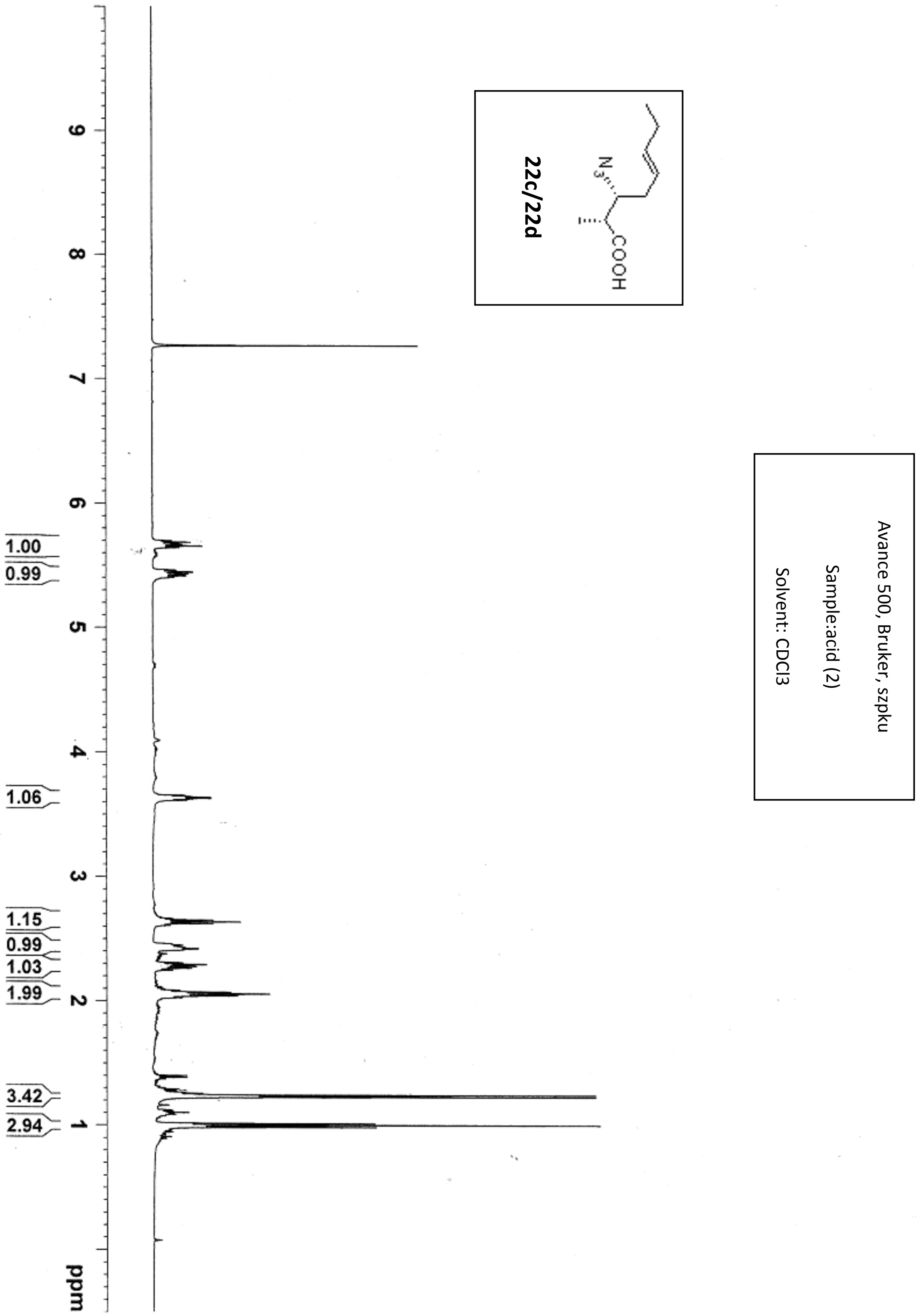
22c/22d
COOH
Avance 500, Bruker, szpku
Sample:acid (2)
Solvent: CDCl3
1.00
0.99
1.06
1.15
0.99
1.03
1.99
3.42
2.94
ppm

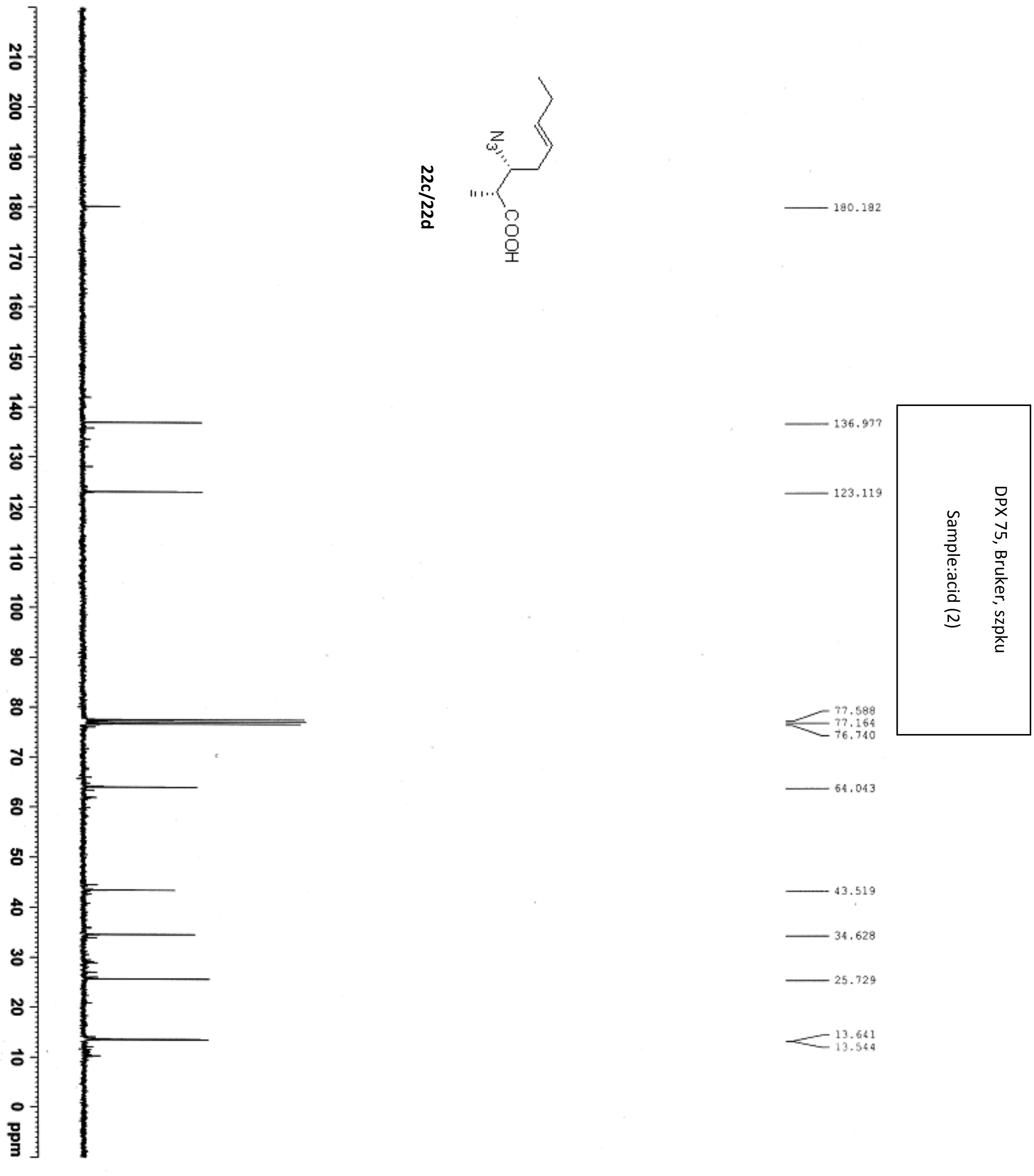
22c/22d
COOH
N3
180.182
136.977
123.119
77.588
77.164
76.740
64.043
43.519
34.628
25.729
13.641
13.544
DPX 75, Bruker, szpku
Sample:acid (2)
210 200 190 180 170 160 150 140 130 120 110 100 90 80 70 60 50 40 30 20 10 0 ppm

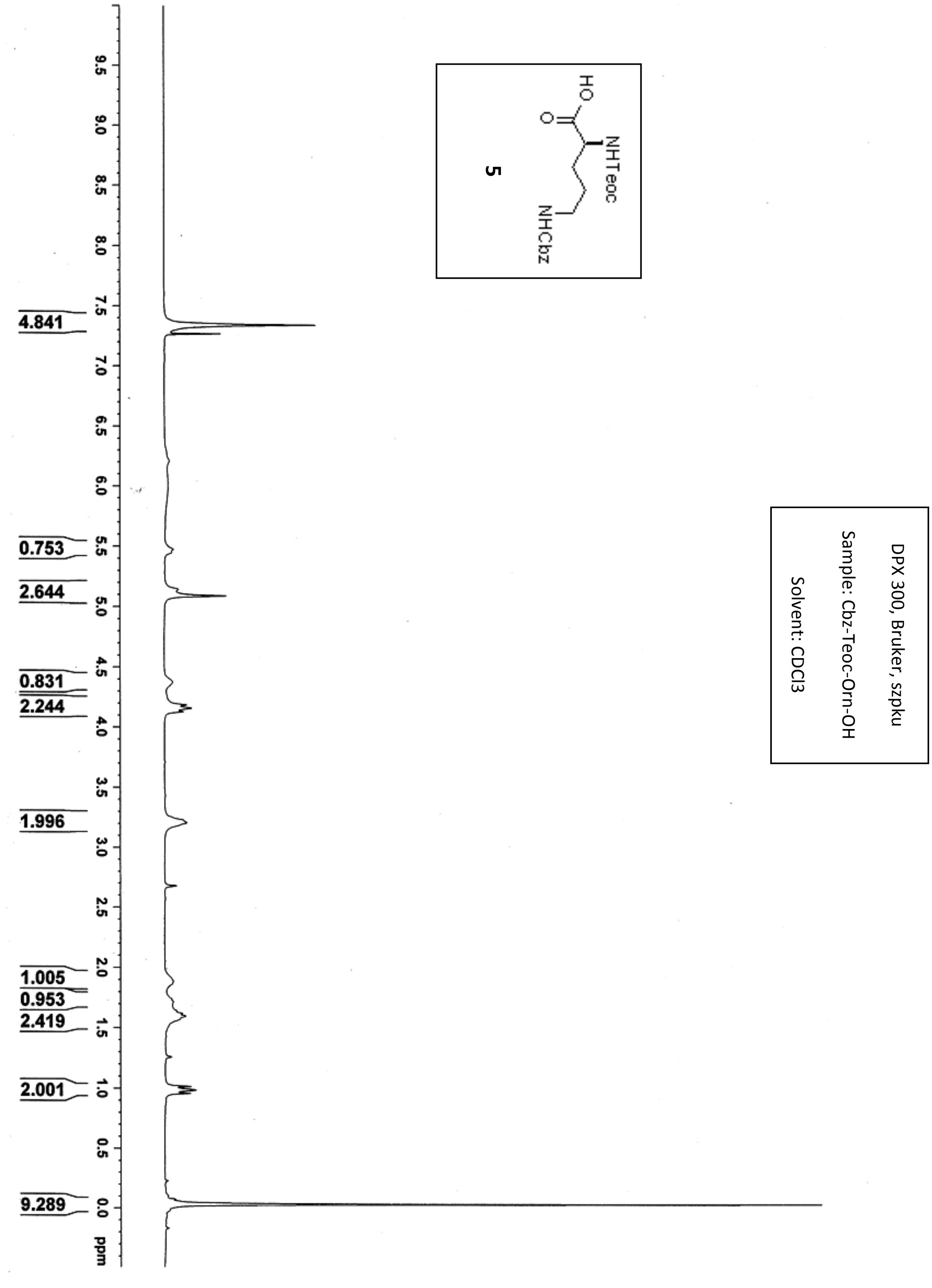

DPX 300, Bruker, szpku
Sample: Cbz-Teoc-Orn-OH
Solvent: CDCl3
5
NHTeoc
NHCbz
HO
O
4.841
0.753
2.644
0.831
2.244
1.996
1.005
0.953
2.419
2.001
9.289
ppm

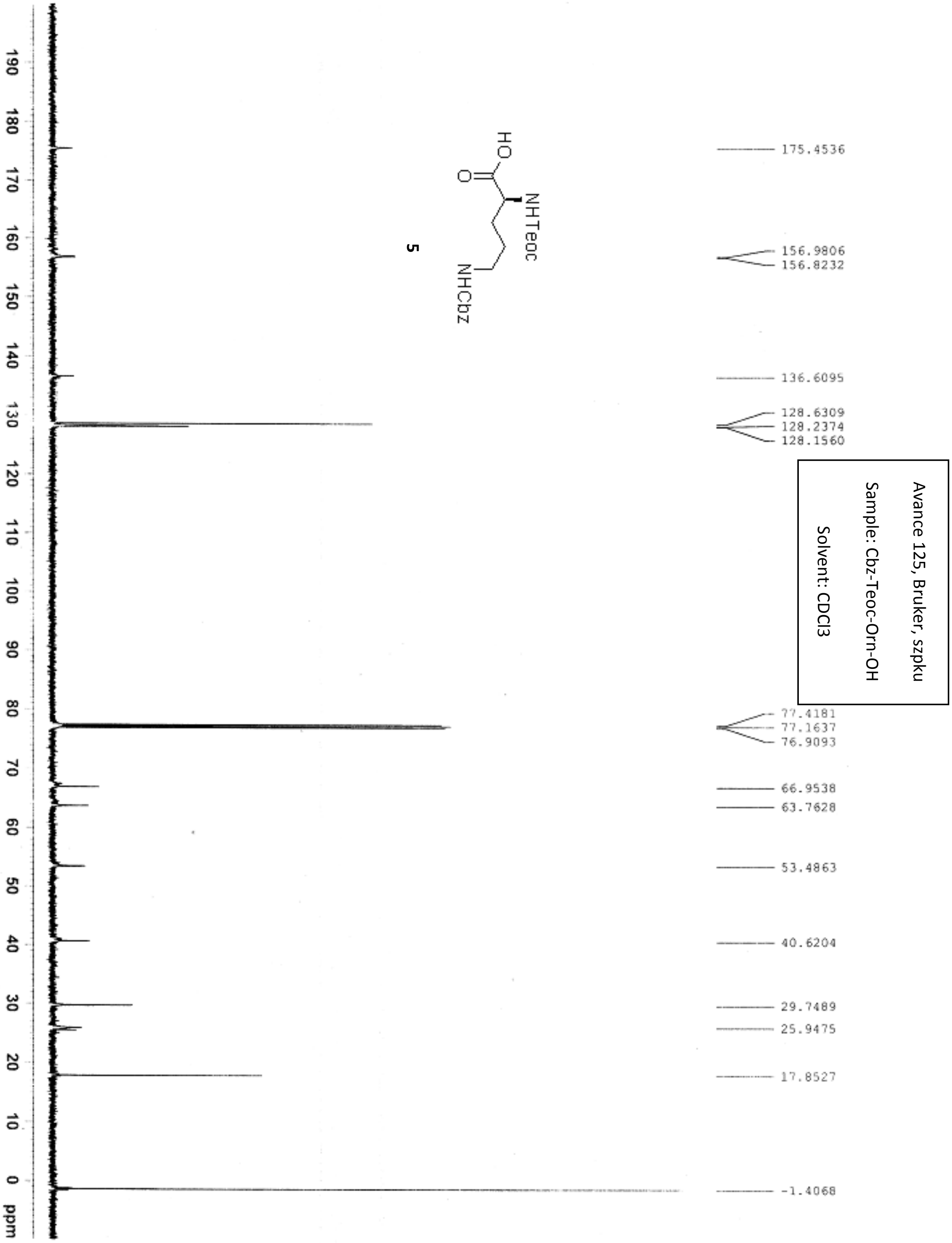
Avance 125, Bruker, szpku
Sample: Cbz-Teoc-Orn-OH
Solvent: CDCl3
NHTeoc
HO
O
NHCbz
5
175.4536
156.9806
156.8232
136.6095
128.6309
128.2374
128.1560
77.4181
77.1637
76.9093
66.9538
63.7628
53.4863
40.6204
29.7489
25.9475
17.8527
-1.4068
ppm

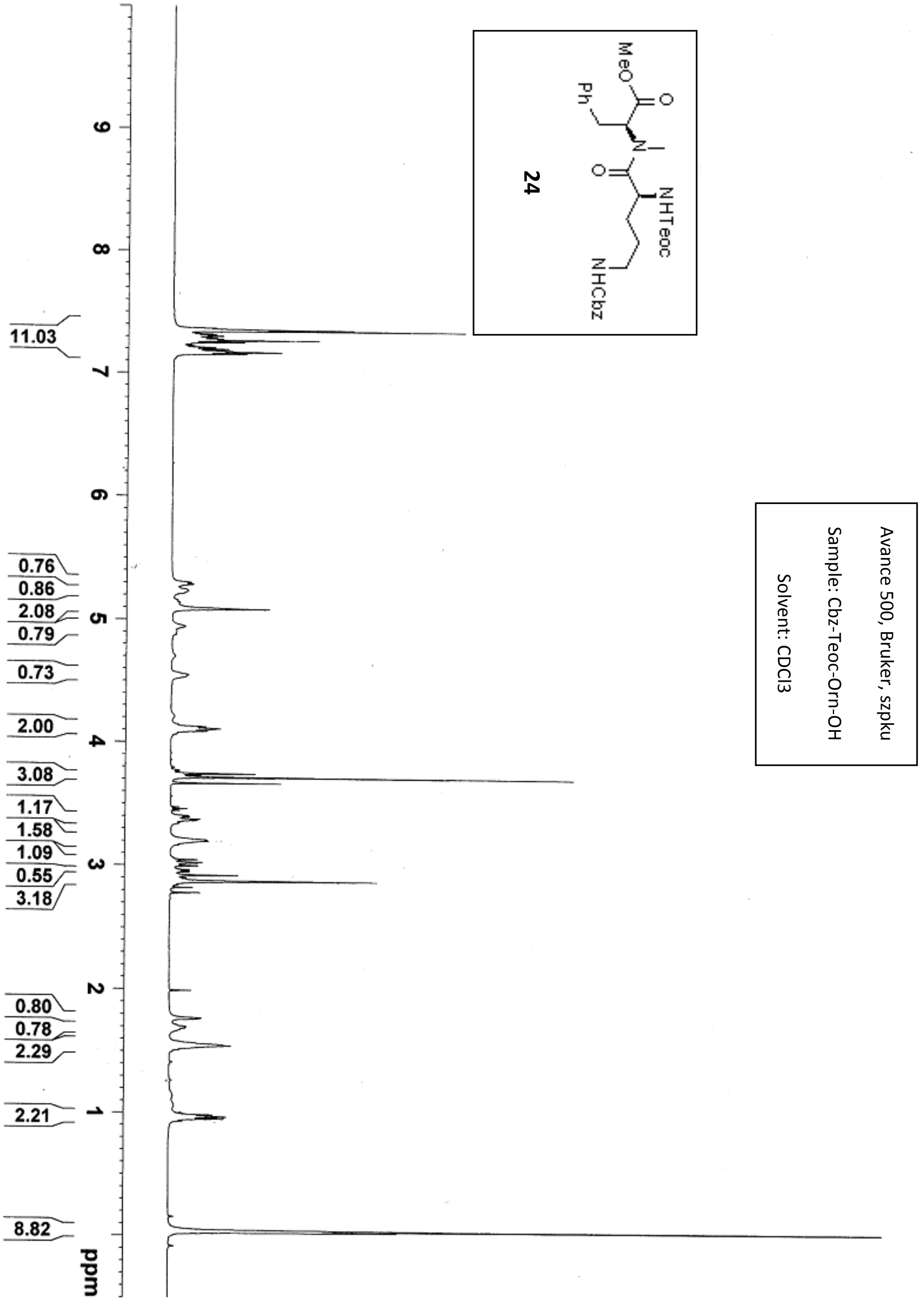

Avance 500, Bruker, szpku
Sample: Cbz-Teoc-Orn-OH
Solvent: CDCl3
24
NHTeoc
NHCbz
MeO
Ph
ppm

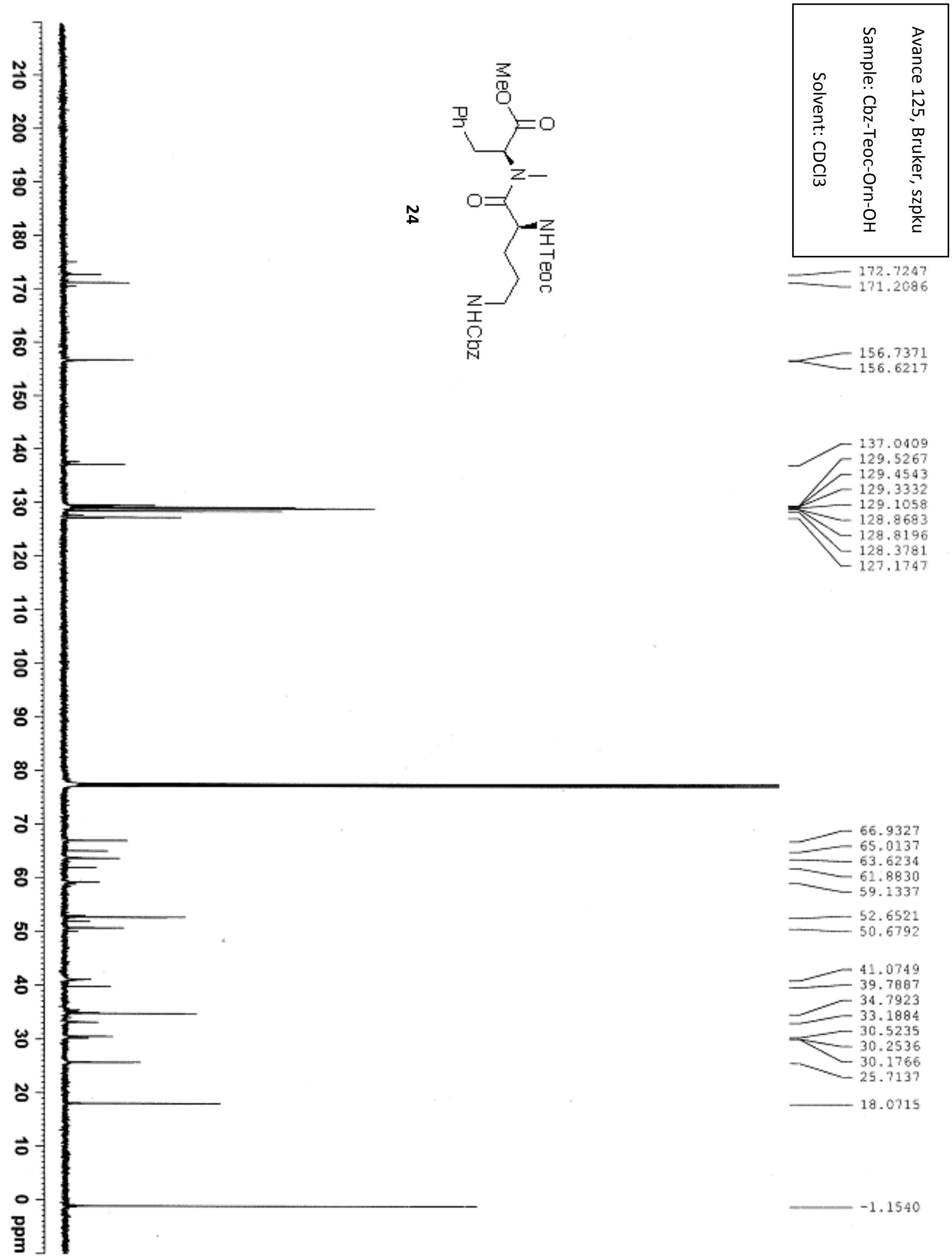
Avance 125, Bruker, szpku
Sample: Cbz-Teoc-Orn-OH
Solvent: CDCl3
MeO
O
Ph
N
O
NHTeoc
NHCbz
24
172.7247
171.2086
156.7371
156.6217
137.0409
129.5267
129.4543
129.3332
129.1058
128.8683
128.8196
128.3781
127.1747
66.9327
65.0137
63.6234
61.8830
59.1337
52.6521
50.6792
41.0749
39.7887
34.7923
33.1884
30.5235
30.2536
30.1766
25.7137
18.0715
-1.1540
210
200
190
180
170
160
150
140
130
120
110
100
90
80
70
60
50
40
30
20
10
0
ppm

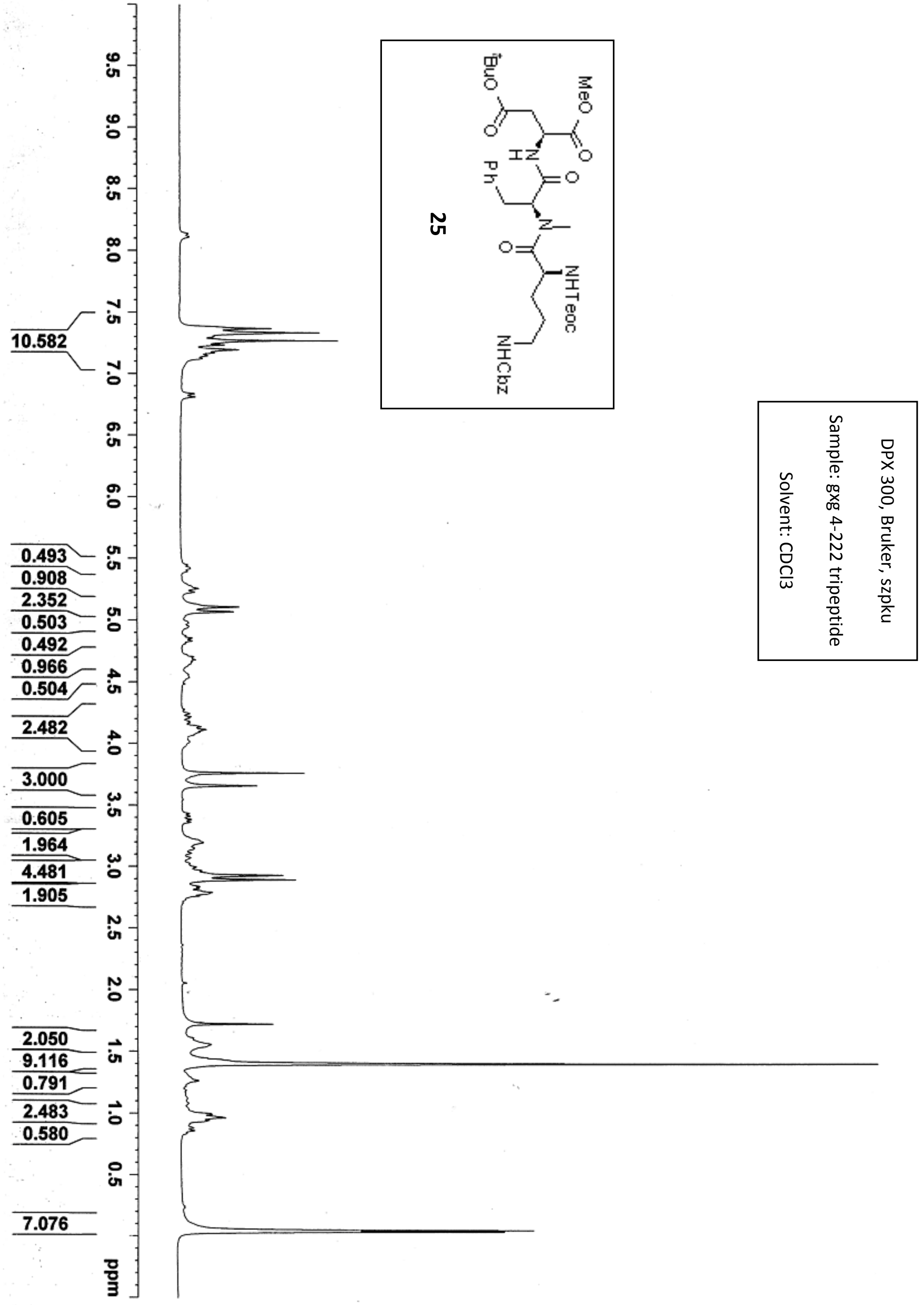
MeO
tBuO
NHTeoc
NHCbz
Ph
25
DPX 300, Bruker, szpku
Sample: gxg 4-222 tripeptide
Solvent: CDCl3
10.582
0.493
0.908
2.352
0.503
0.492
0.966
0.504
2.482
3.000
0.605
1.964
4.481
1.905
2.050
9.116
0.791
2.483
0.580
7.076
ppm

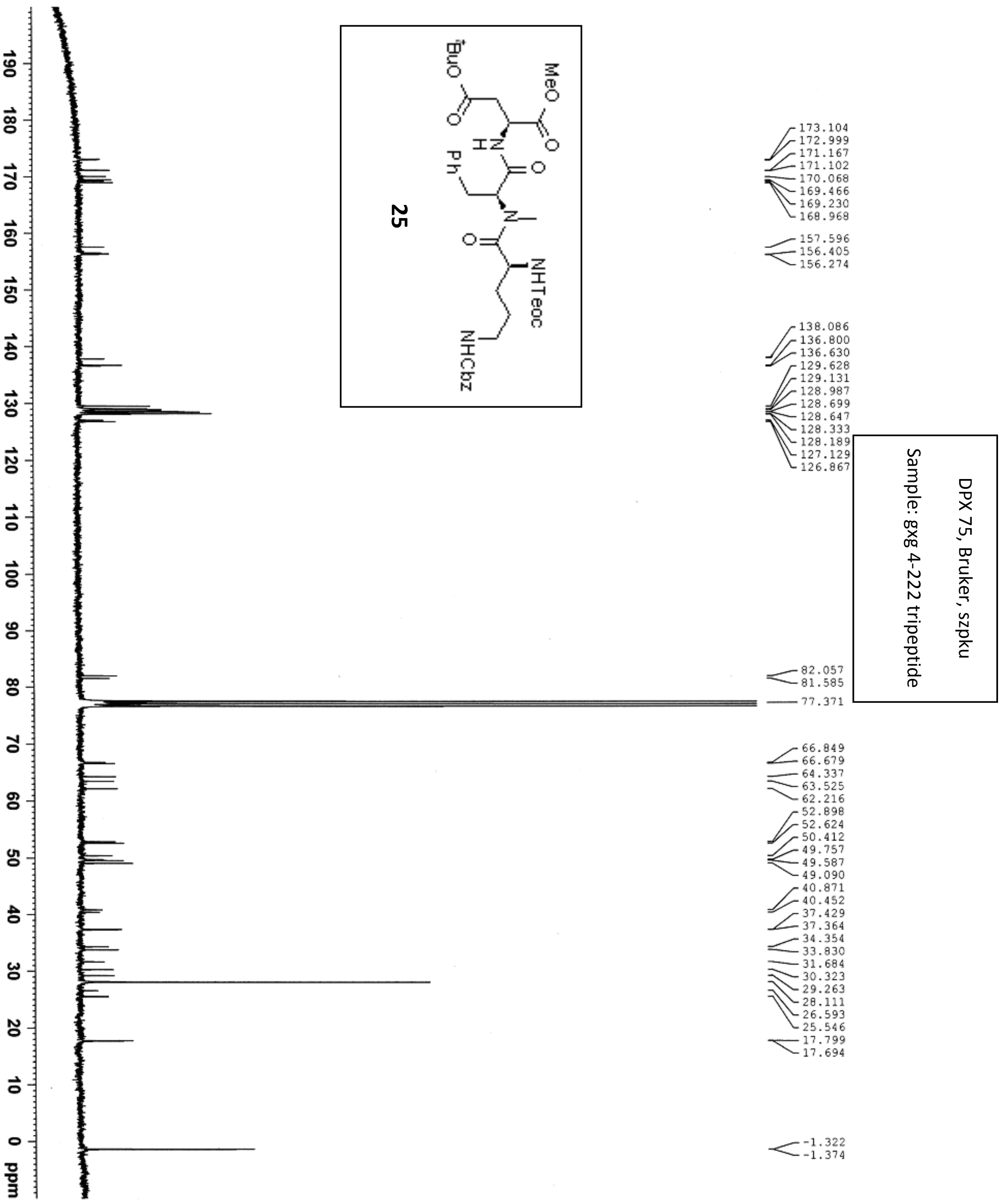
DPX 75, Bruker, szpku
Sample: gxg 4-222 tripeptide
25
MeO
NHTeoc
NHCbz
Ph

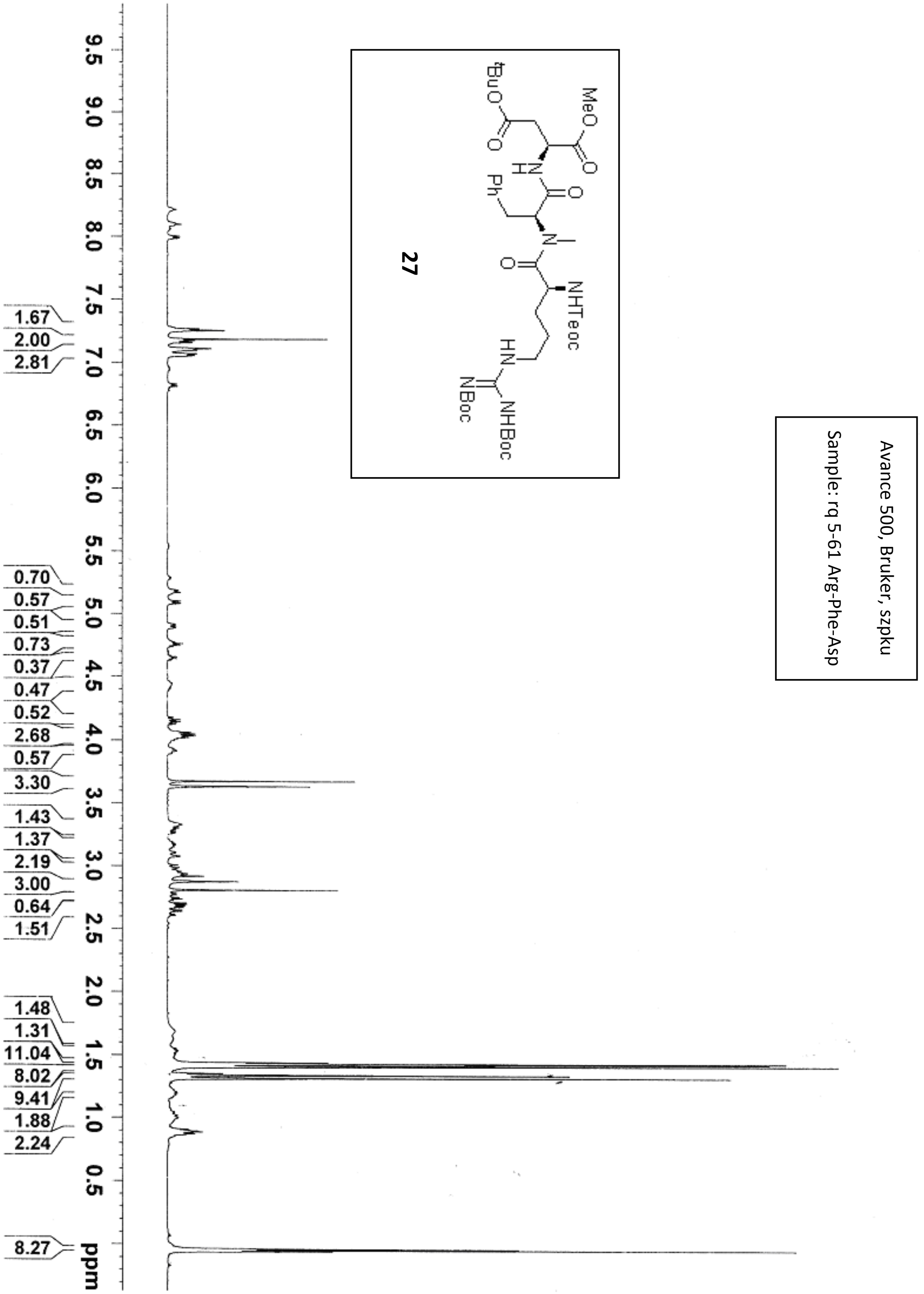
27
Avance 500, Bruker, szpku
Sample: rq 5-61 Arg-Phe-Asp

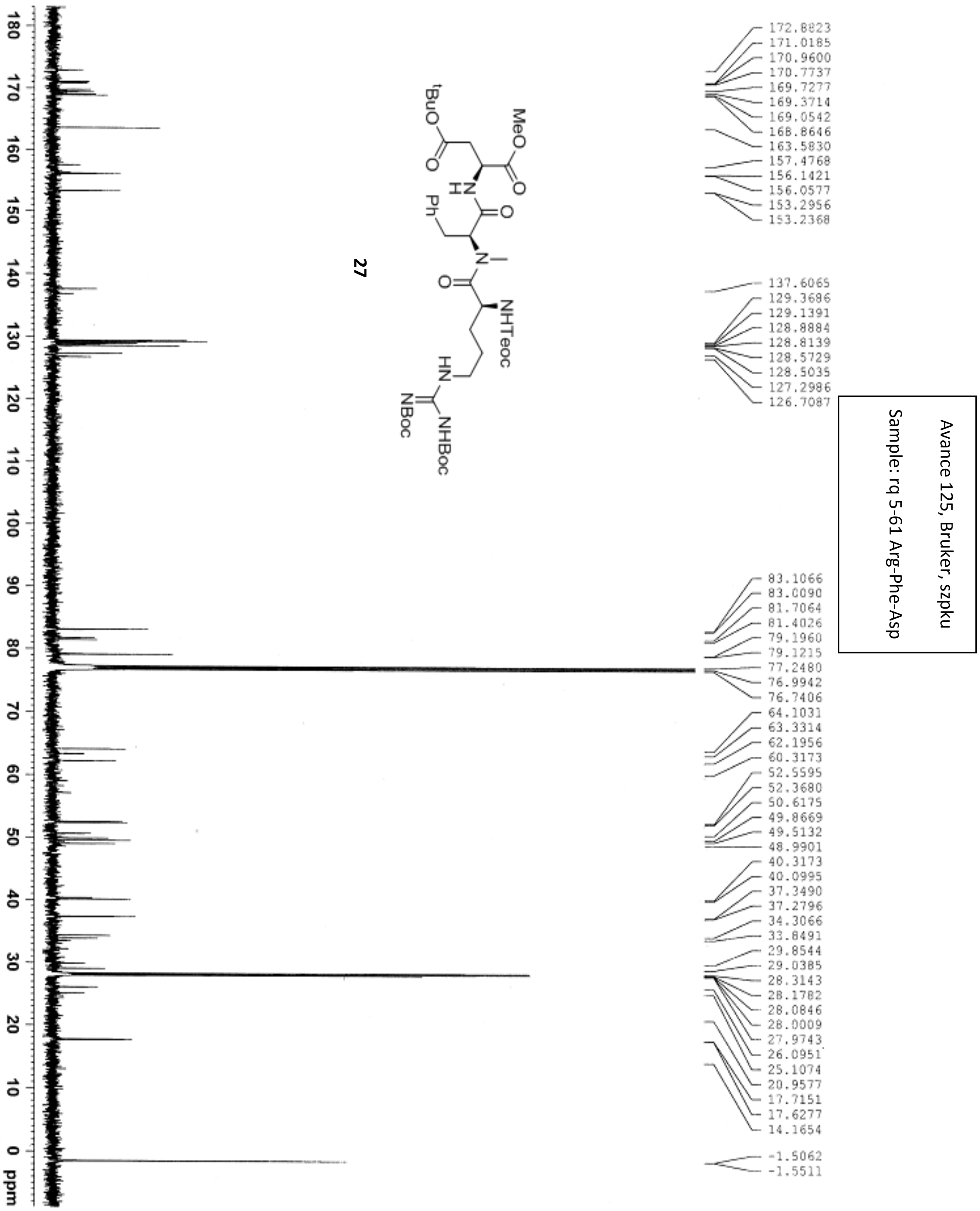
Avance 125, Bruker, szpku
Sample: rq 5-61 Arg-Phe-Asp
27
MeO
tBuO
NHTeoc
NHBoc
NBoc
Ph
172.8823
171.0185
170.9600
170.7737
169.7277
169.3714
169.0542
168.8646
163.5830
157.4768
156.1421
156.0577
153.2956
153.2368
137.6065
129.3686
129.1391
128.8884
128.8139
128.5729
128.5035
127.2986
126.7087
83.1066
83.0090
81.7064
81.4026
79.1960
79.1215
77.2480
76.9942
76.7406
64.1031
63.3314
62.1956
60.3173
52.5595
52.3680
50.6175
49.8669
49.5132
48.9901
40.3173
40.0995
37.3490
37.2796
34.3066
33.8491
29.8544
29.0385
28.3143
28.1782
28.0846
28.0009
27.9743
26.0951
25.1074
20.9577
17.7151
17.6277
14.1654
-1.5062
-1.5511
ppm

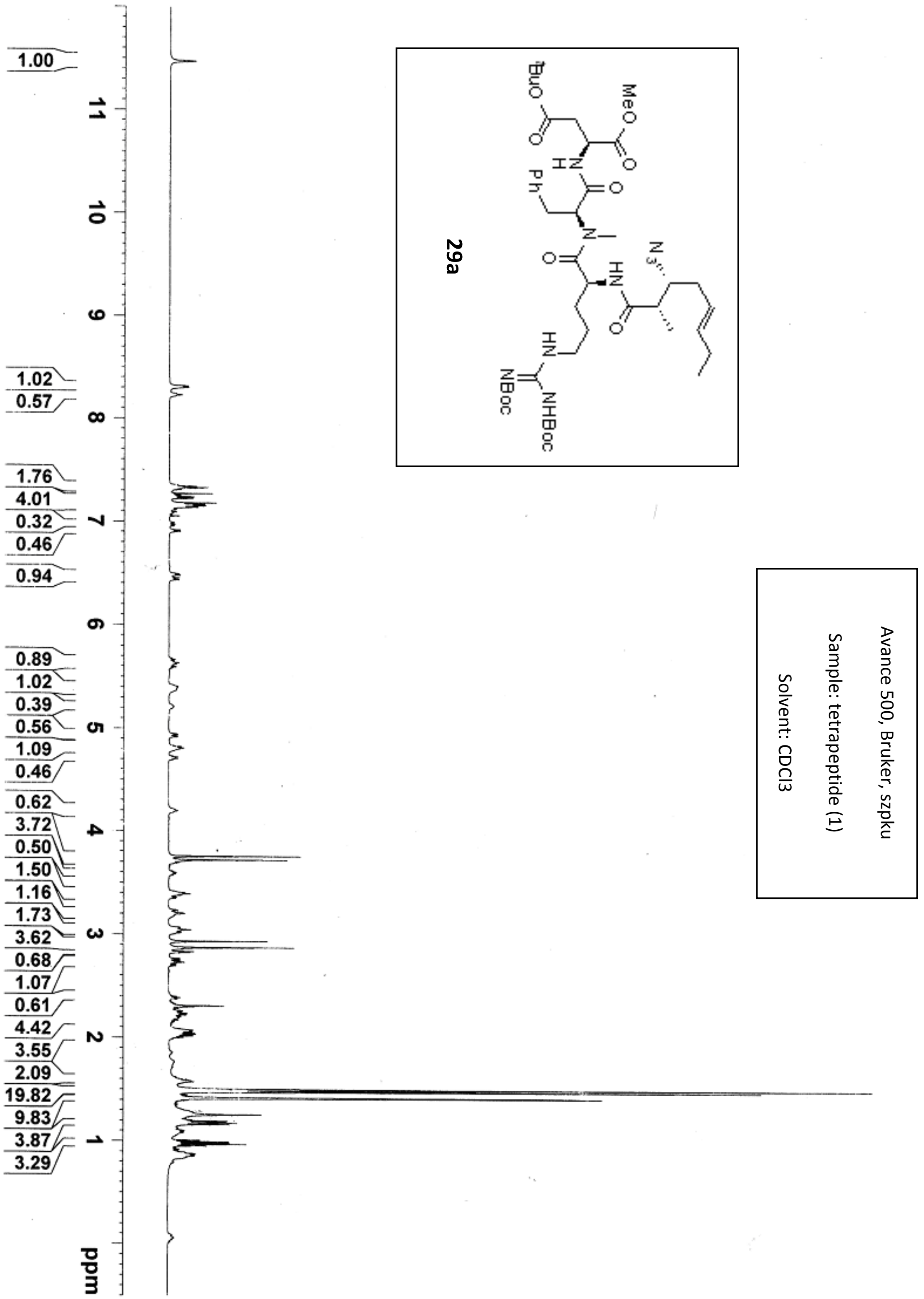
29a
Avance 500, Bruker, szpku
Sample: tetrapeptide (1)
Solvent: CDCl3
ppm

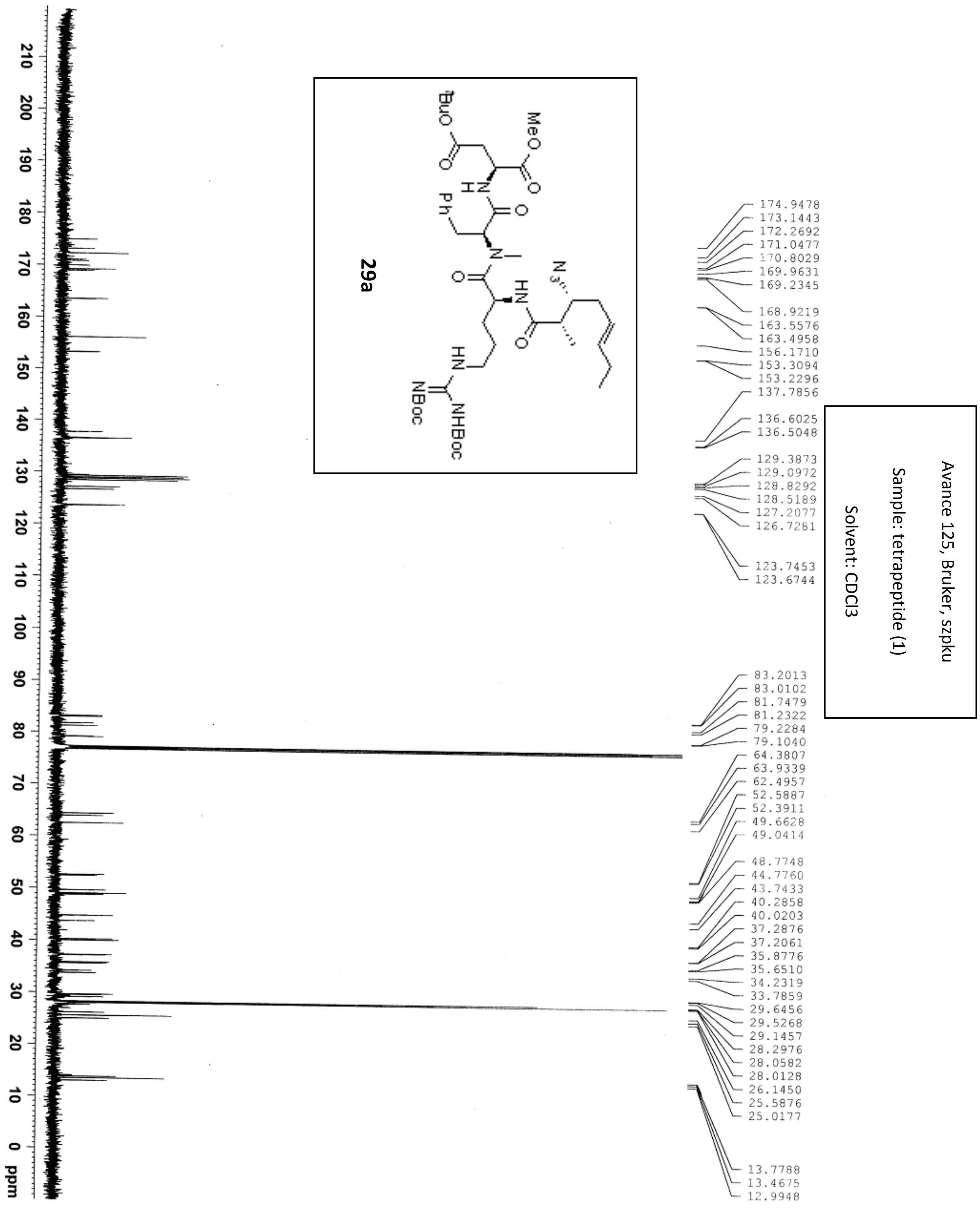
29a
Avance 125, Bruker, szpku
Sample: tetrapeptide (1)
Solvent: CDCl3
174.9478
173.1443
172.2692
171.0477
170.8029
169.9631
169.2345
168.9219
163.5576
163.4958
156.1710
153.3094
153.2296
137.7856
136.6025
136.5048
129.3873
129.0972
128.8292
128.5189
127.2077
126.7261
123.7453
123.6744
83.2013
83.0102
81.7479
81.2322
79.2284
79.1040
64.3807
63.9339
62.4957
52.5887
52.3911
49.6628
49.0414
48.7748
44.7760
43.7433
40.2858
40.0203
37.2876
37.2061
35.8776
35.6510
34.2319
33.7859
29.6456
29.5268
29.1457
28.2976
28.0582
28.0128
26.1450
25.5876
25.0177
13.7788
13.4675
12.9948
ppm

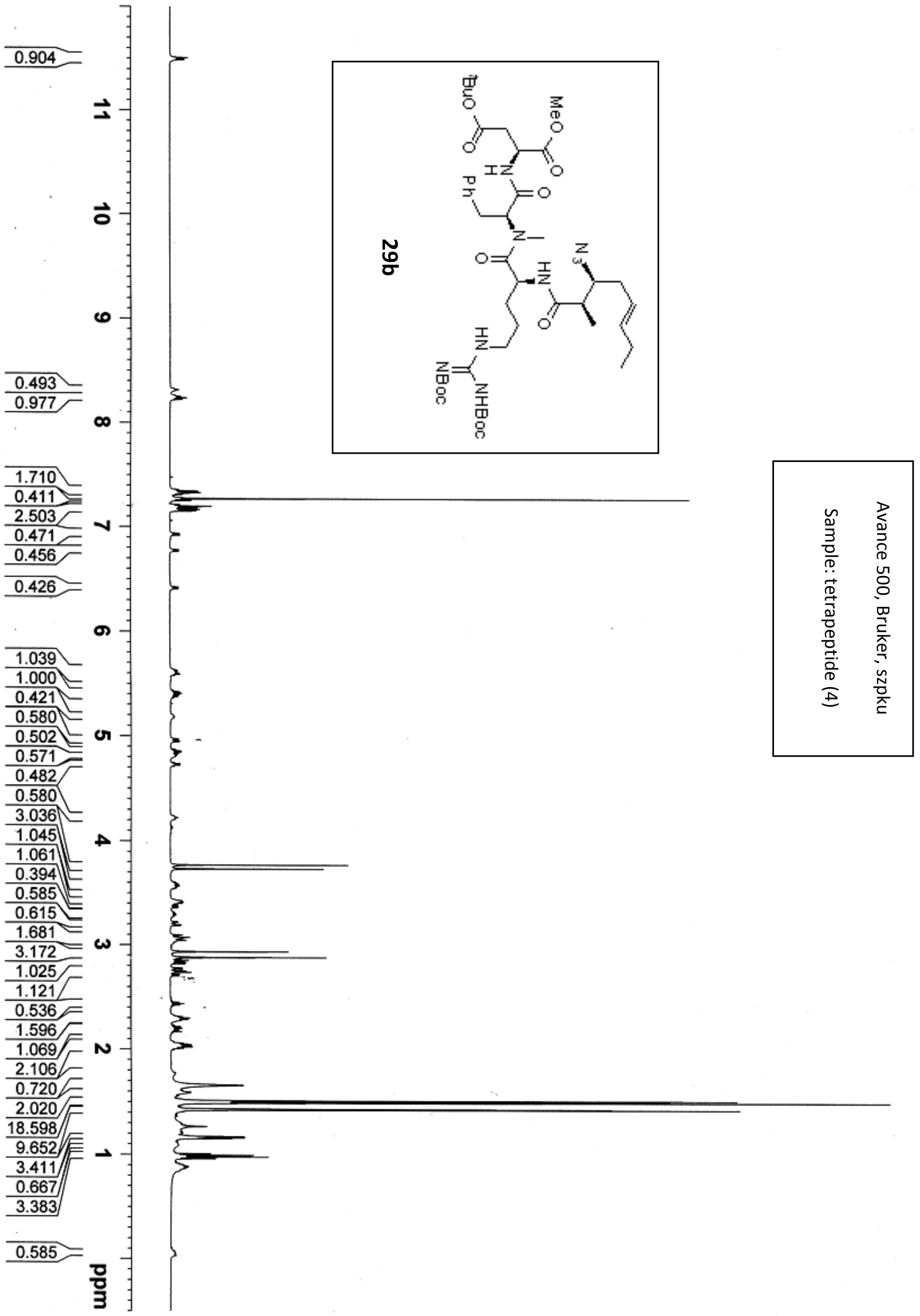
29b
Avance 500, Bruker, szpku
Sample: tetrapeptide (4)
ppm

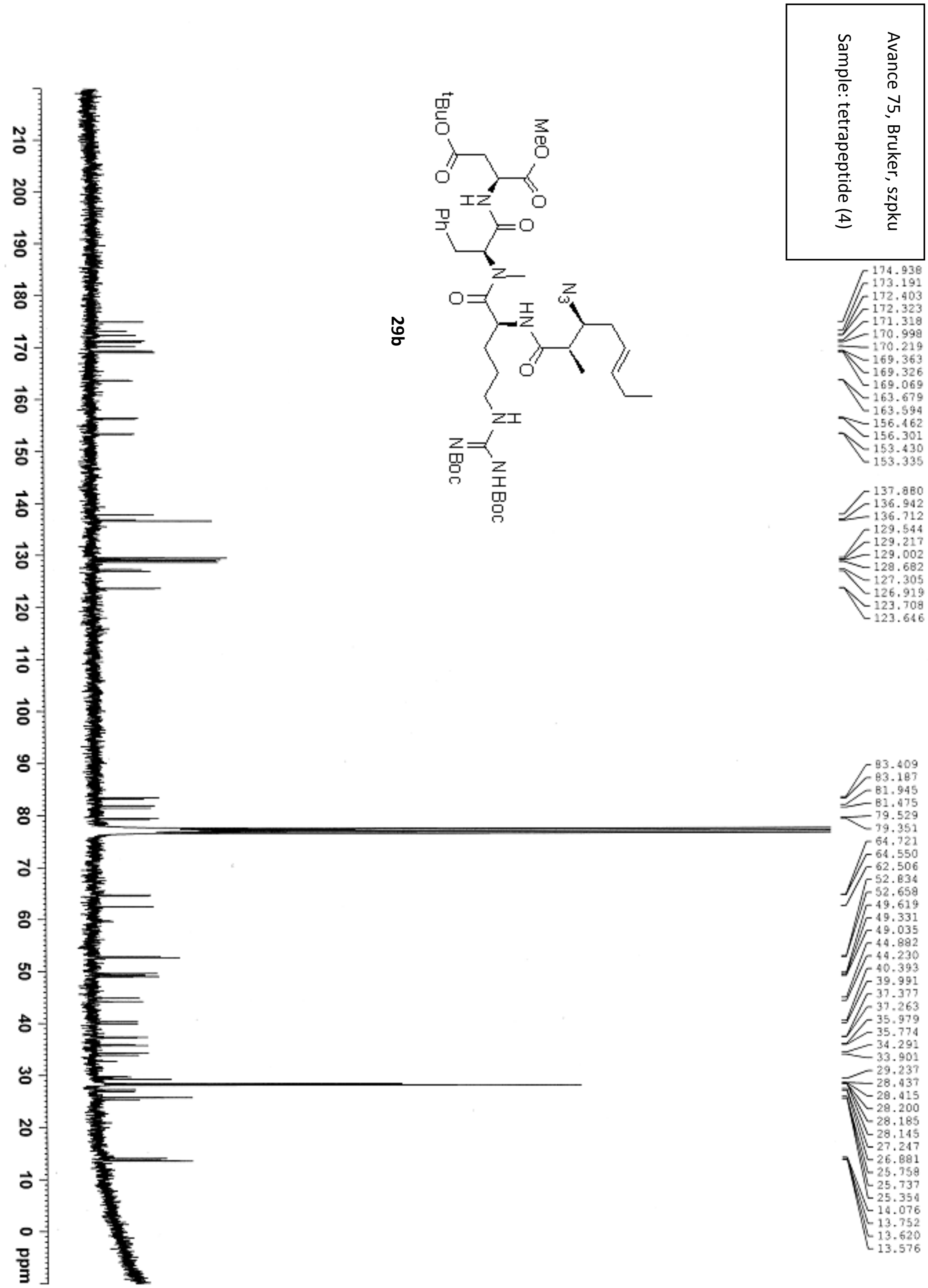
Avance 75, Bruker, szpku
Sample: tetrapeptide (4)
29b
MeO
tBuO
Ph
N3
HN
NHBoc
NBoc
174.938
173.191
172.403
172.323
171.318
170.998
170.219
169.363
169.326
169.069
163.679
163.594
156.462
156.301
153.430
153.335
137.880
136.942
136.712
129.544
129.217
129.002
128.682
127.305
126.919
123.708
123.646
83.409
83.187
81.945
81.475
79.529
79.351
64.721
64.550
62.506
52.834
52.658
49.619
49.331
49.035
44.882
44.230
40.393
39.991
37.377
37.263
35.979
35.774
34.291
33.901
29.237
28.437
28.415
28.200
28.185
28.145
27.247
26.881
25.758
25.737
25.354
14.076
13.752
13.620
13.576
ppm

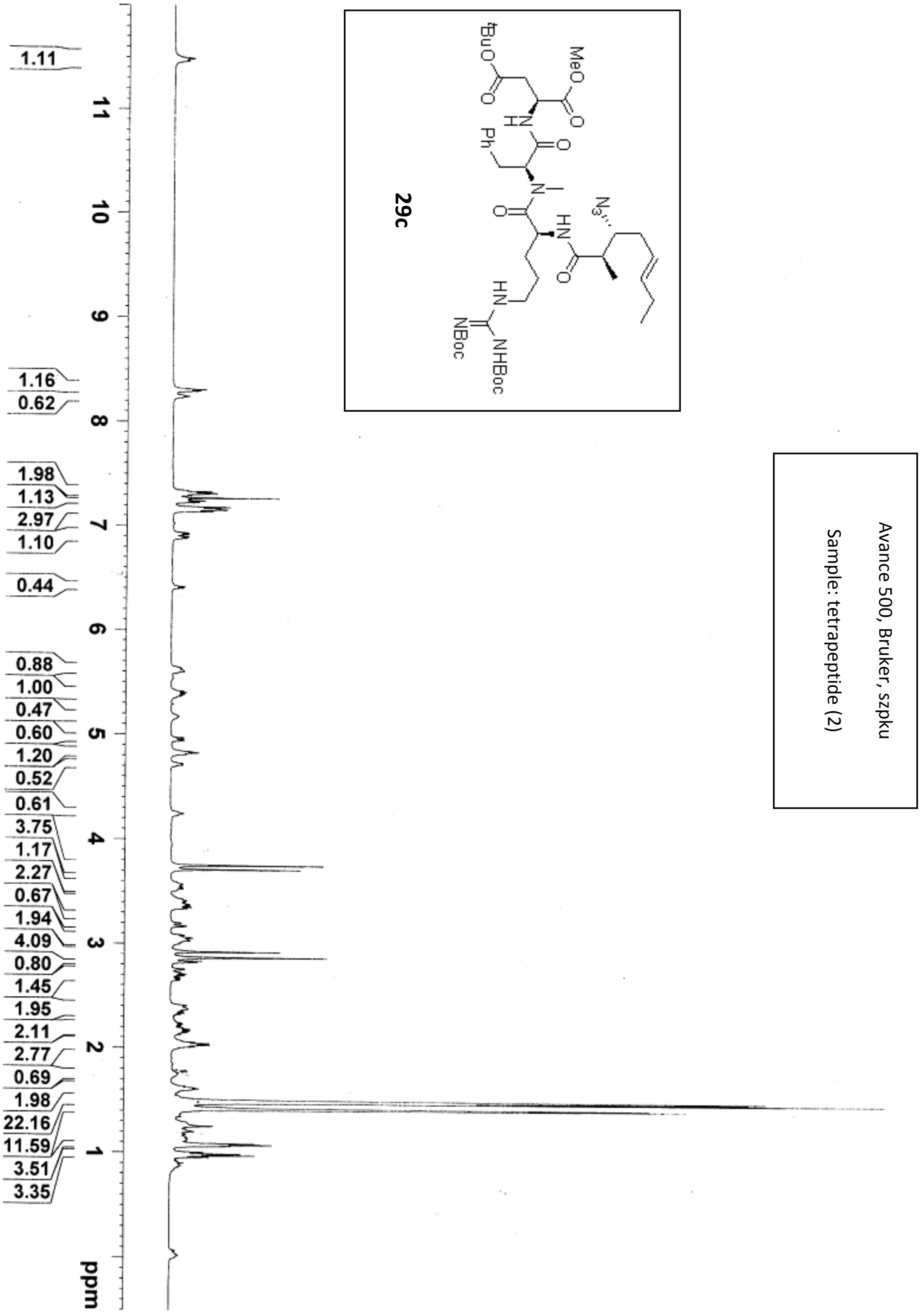
29c
Avance 500, Bruker, szpku
Sample: tetrapeptide (2)
ppm

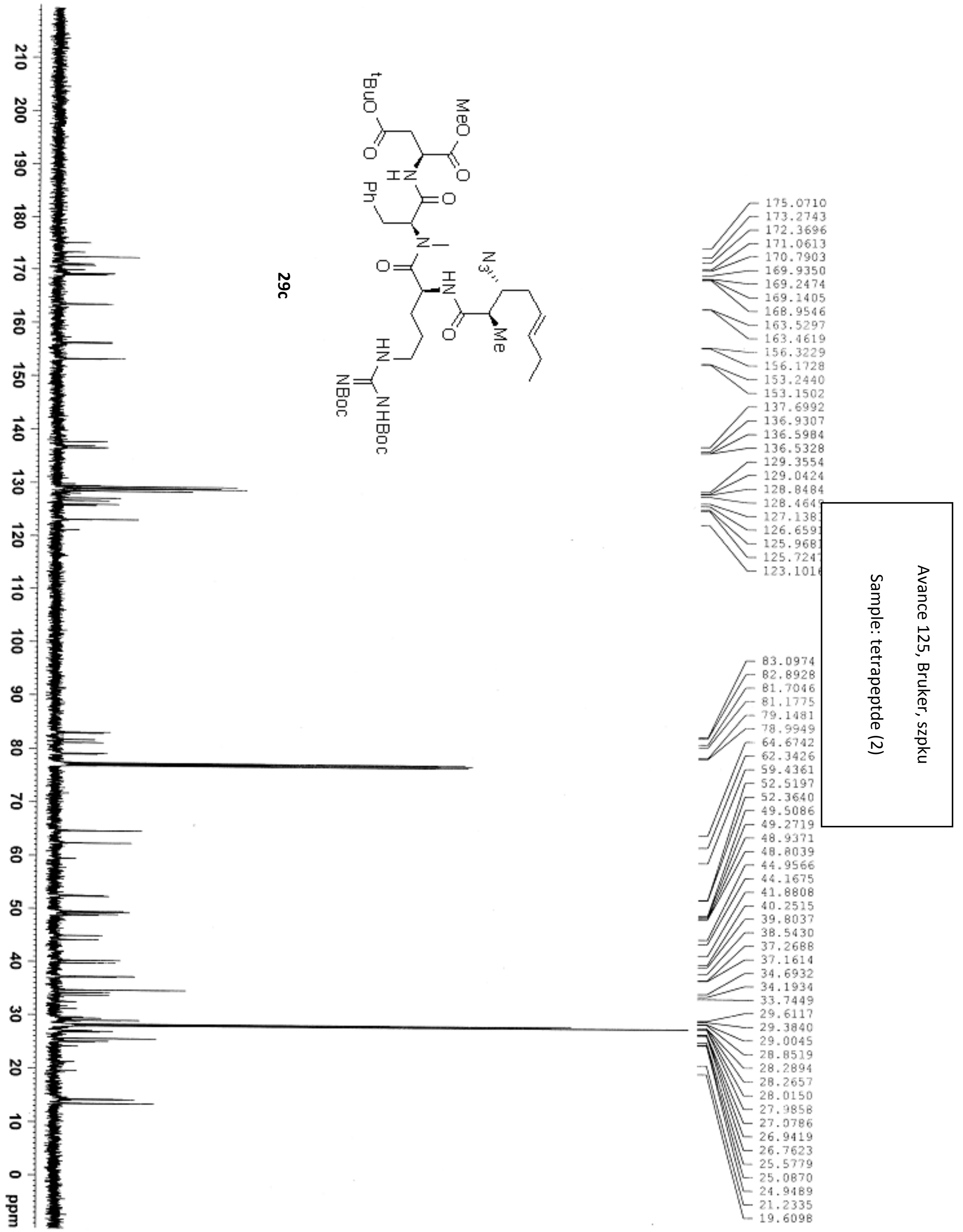
29c
Avance 125, Bruker, szpku
Sample: tetrapeptde (2)
ppm

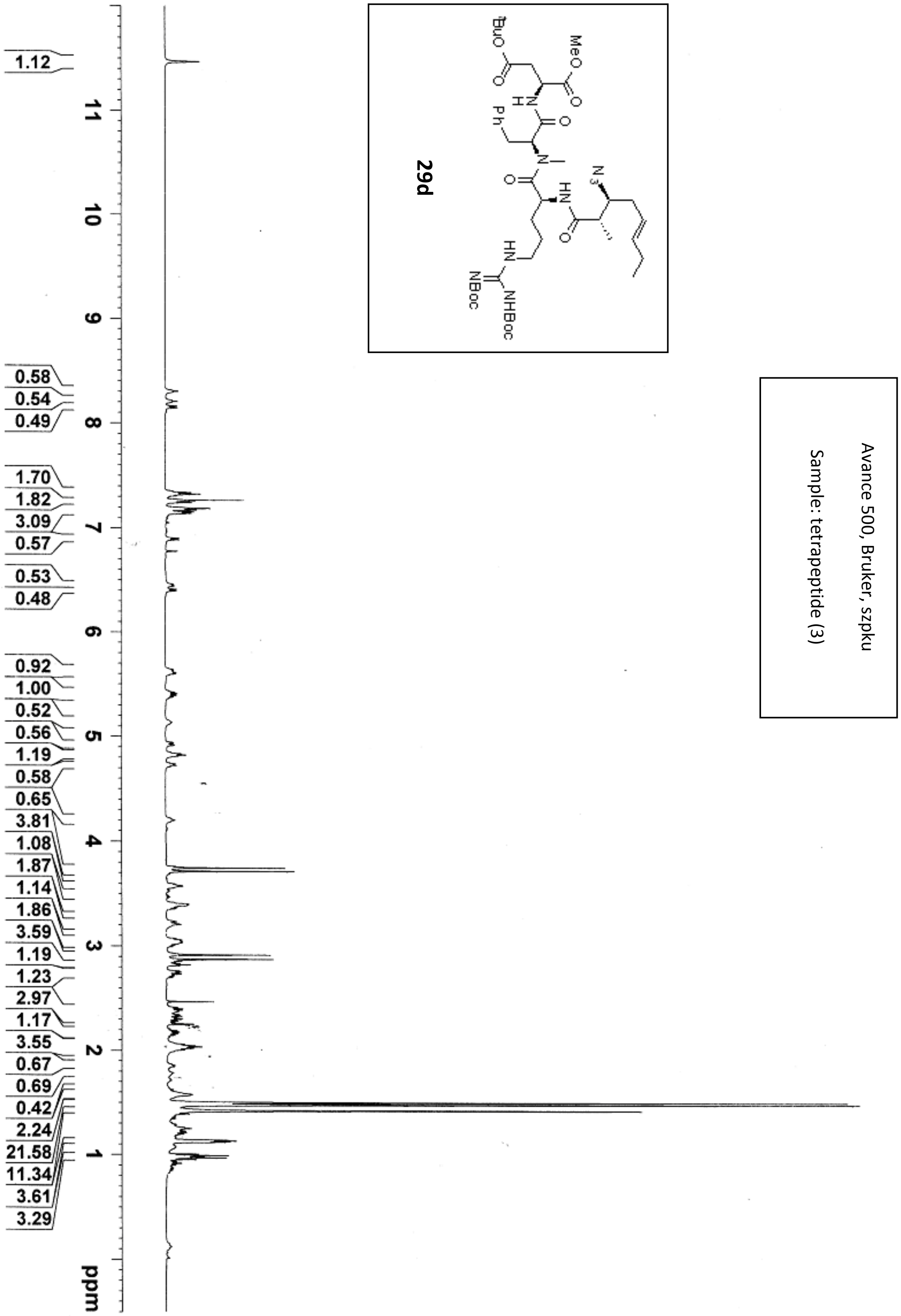
29d
Avance 500, Bruker, szpku
Sample: tetrapeptide (3)
ppm

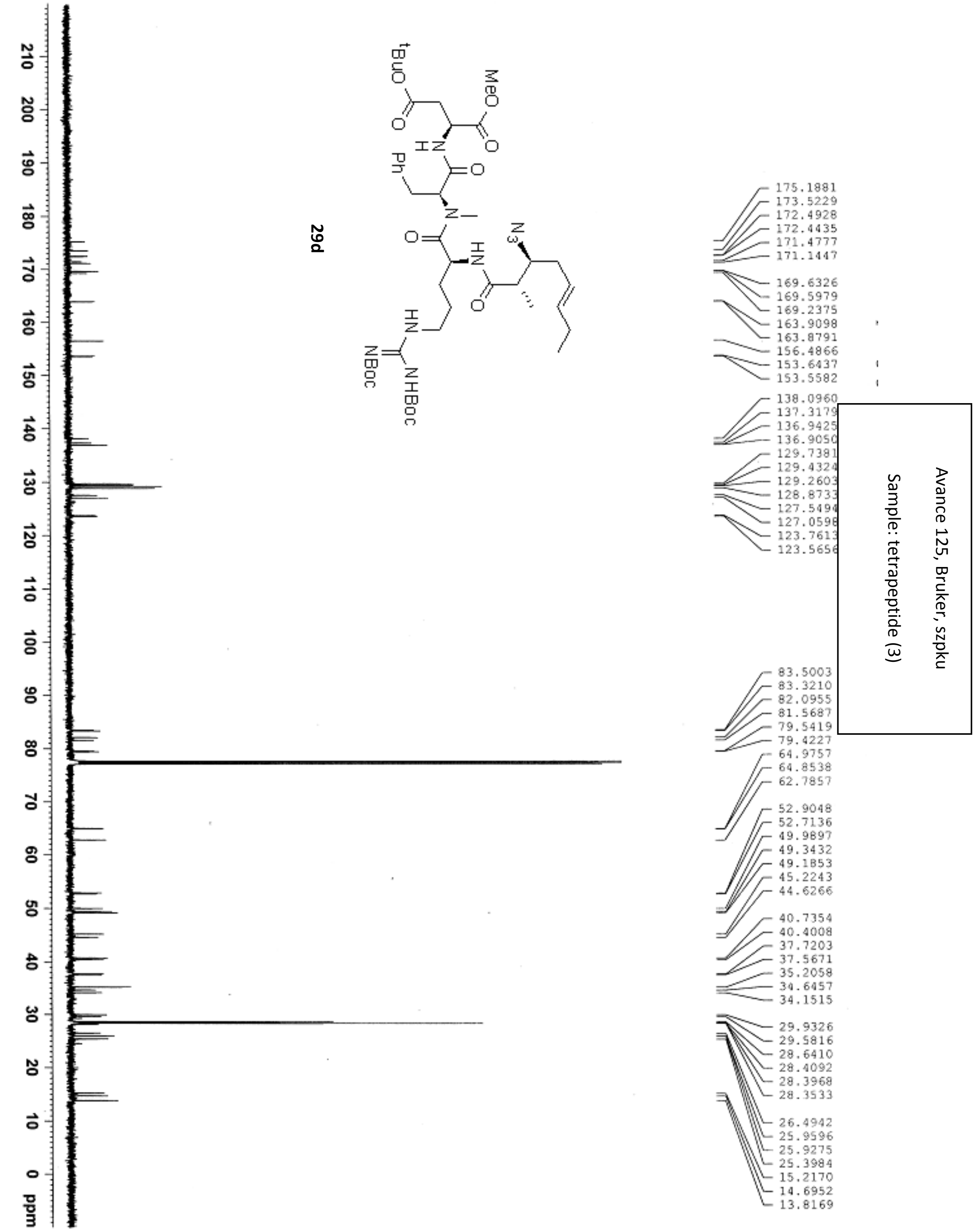
Avance 125, Bruker, szpku
Sample: tetrapeptide (3)
MeO
tBuO
NHBoc
NBoc
Ph
N3
175.1881
173.5229
172.4928
172.4435
171.4777
171.1447
169.6326
169.5979
169.2375
163.9098
163.8791
156.4866
153.6437
153.5582
138.0960
137.3179
136.9425
136.9050
129.7381
129.4324
129.2603
128.8733
127.5494
127.0598
123.7613
123.5656
83.5003
83.3210
82.0955
81.5687
79.5419
79.4227
64.9757
64.8538
62.7857
52.9048
52.7136
49.9897
49.3432
49.1853
45.2243
44.6266
40.7354
40.4008
37.7203
37.5671
35.2058
34.6457
34.1515
29.9326
29.5816
28.6410
28.4092
28.3968
28.3533
26.4942
25.9596
25.9275
25.3984
15.2170
14.6952
13.8169
ppm

29d

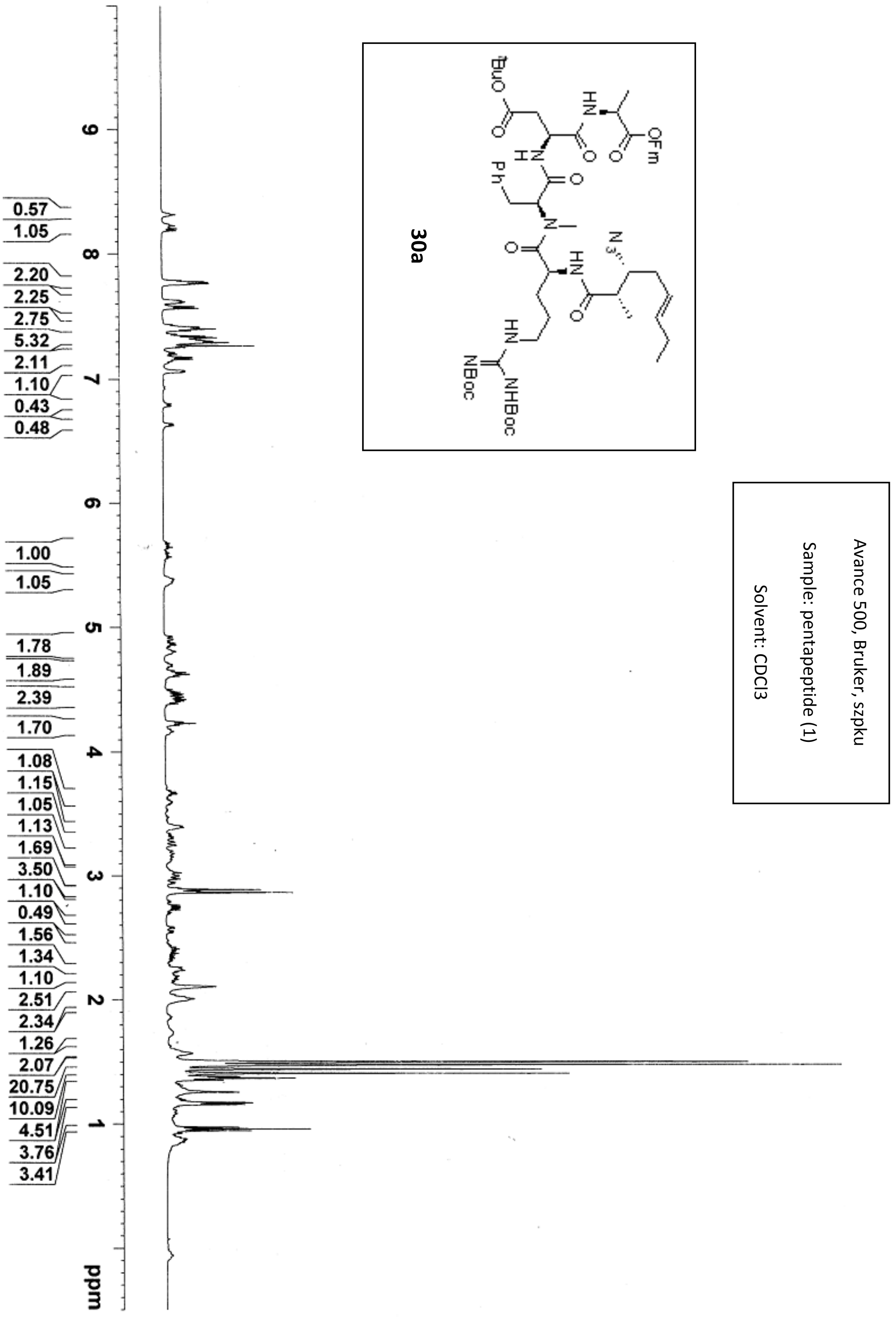
30a
Avance 500, Bruker, szpku
Sample: pentapeptide (1)
Solvent: CDCl3
ppm

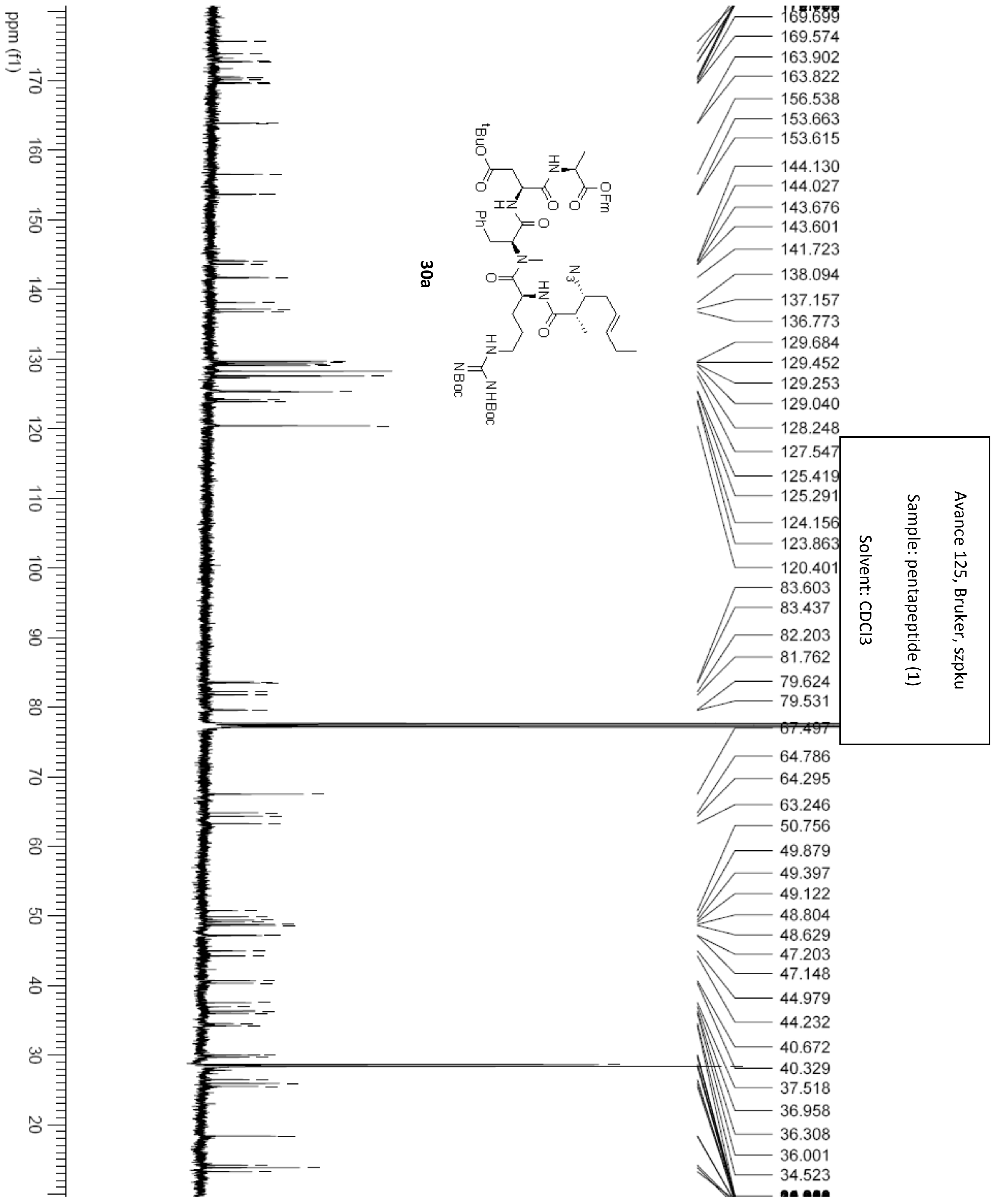
30a
Avance 125, Bruker, szpku
Sample: pentapeptide (1)
Solvent: CDCl3
ppm (f1)
169.699
169.574
163.902
163.822
156.538
153.663
153.615
144.130
144.027
143.676
143.601
141.723
138.094
137.157
136.773
129.684
129.452
129.253
129.040
128.248
127.547
125.419
125.291
124.156
123.863
120.401
83.603
83.437
82.203
81.762
79.624
79.531
67.497
64.786
64.295
63.246
50.756
49.879
49.397
49.122
48.804
48.629
47.203
47.148
44.979
44.232
40.672
40.329
37.518
36.958
36.308
36.001
34.523

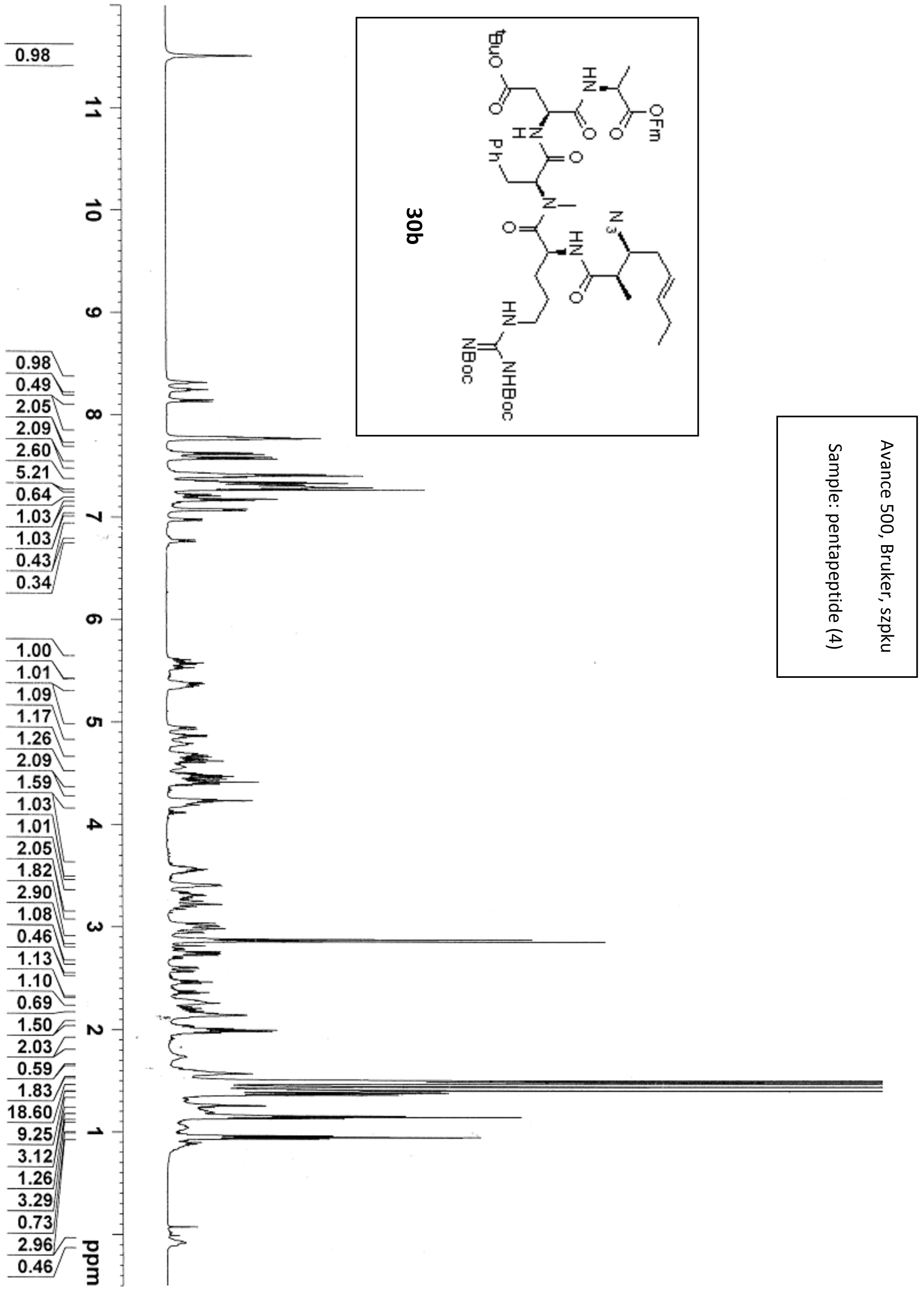
30b
Avance 500, Bruker, szpku
Sample: pentapeptide (4)
ppm

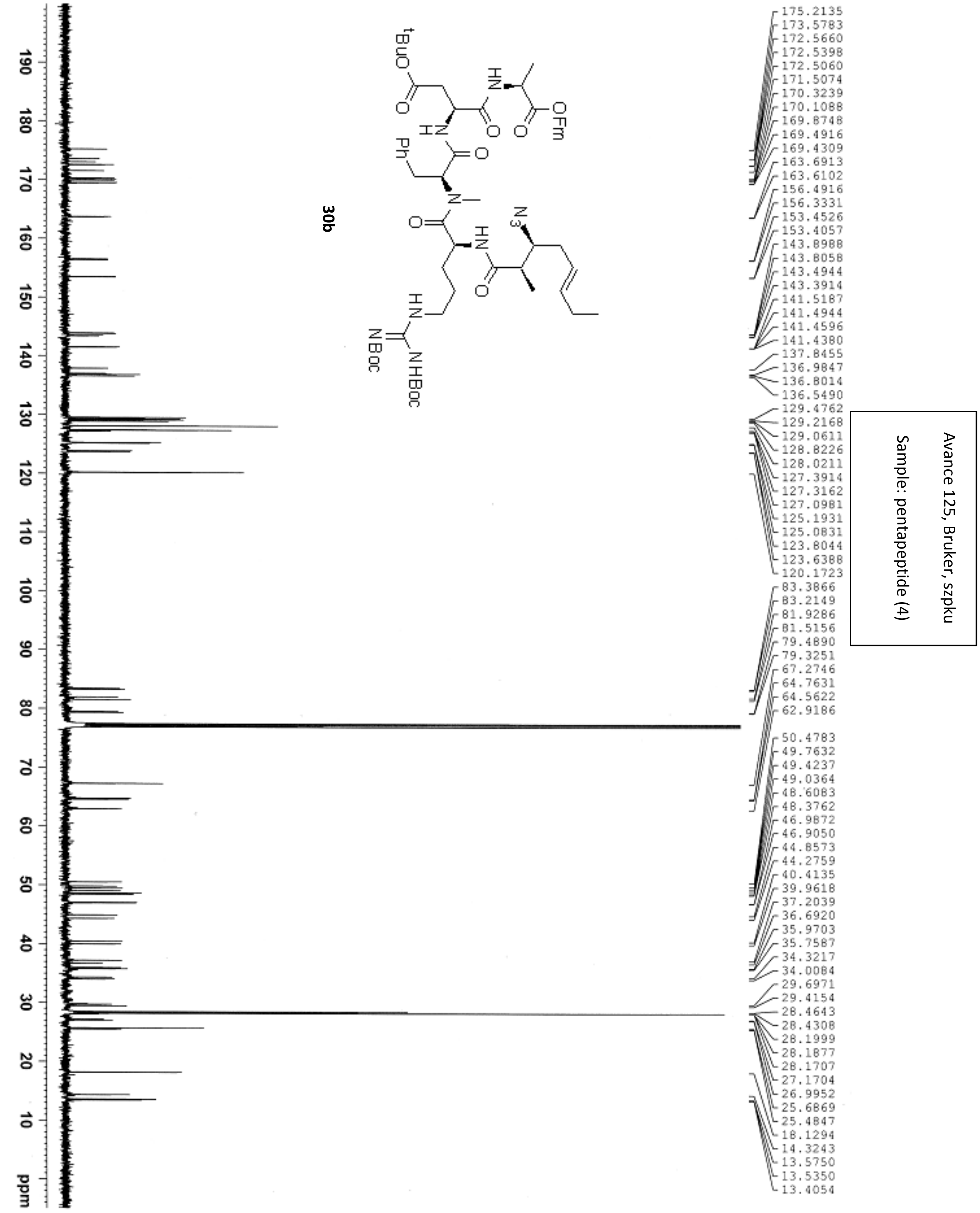
30b
Avance 125, Bruker, szpku
Sample: pentapeptide (4)
ppm
190
180
170
160
150
140
130
120
110
100
90
80
70
60
50
40
30
20
10
175.2135
173.5783
172.5660
172.5398
172.5060
171.5074
170.3239
170.1088
169.8748
169.4916
169.4309
163.6913
163.6102
156.4916
156.3331
153.4526
153.4057
143.8988
143.8058
143.4944
143.3914
141.5187
141.4944
141.4596
141.4380
137.8455
136.9847
136.8014
136.5490
129.4762
129.2168
129.0611
128.8226
128.0211
127.3914
127.3162
127.0981
125.1931
125.0831
123.8044
123.6388
120.1723
83.3866
83.2149
81.9286
81.5156
79.4890
79.3251
67.2746
64.7631
64.5622
62.9186
50.4783
49.7632
49.4237
49.0364
48.6083
48.3762
46.9872
46.9050
44.8573
44.2759
40.4135
39.9618
37.2039
36.6920
35.9703
35.7587
34.3217
34.0084
29.6971
29.4154
28.4643
28.4308
28.1999
28.1877
28.1707
27.1704
26.9952
25.6869
25.4847
18.1294
14.3243
13.5750
13.5350
13.4054

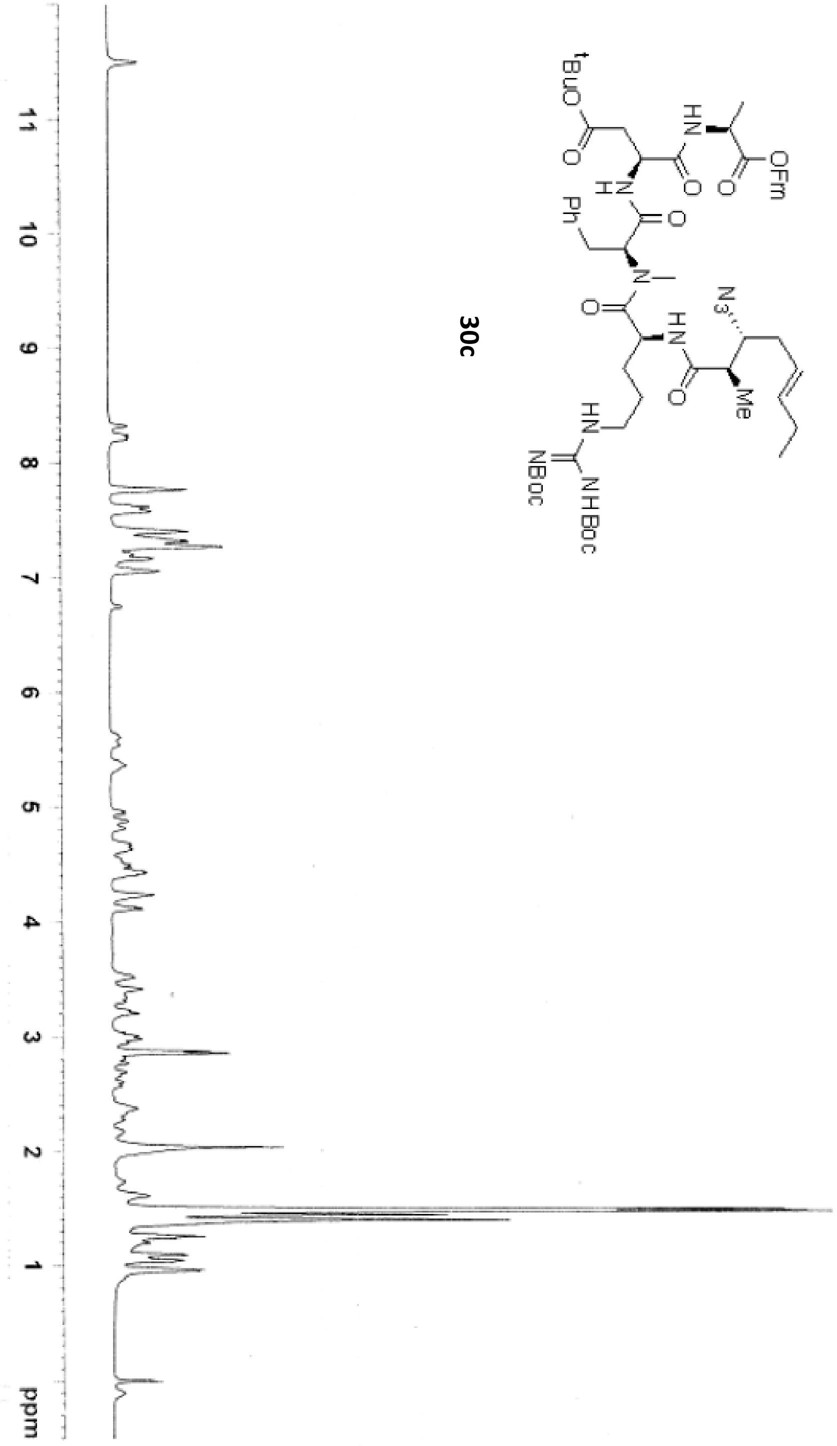

30c

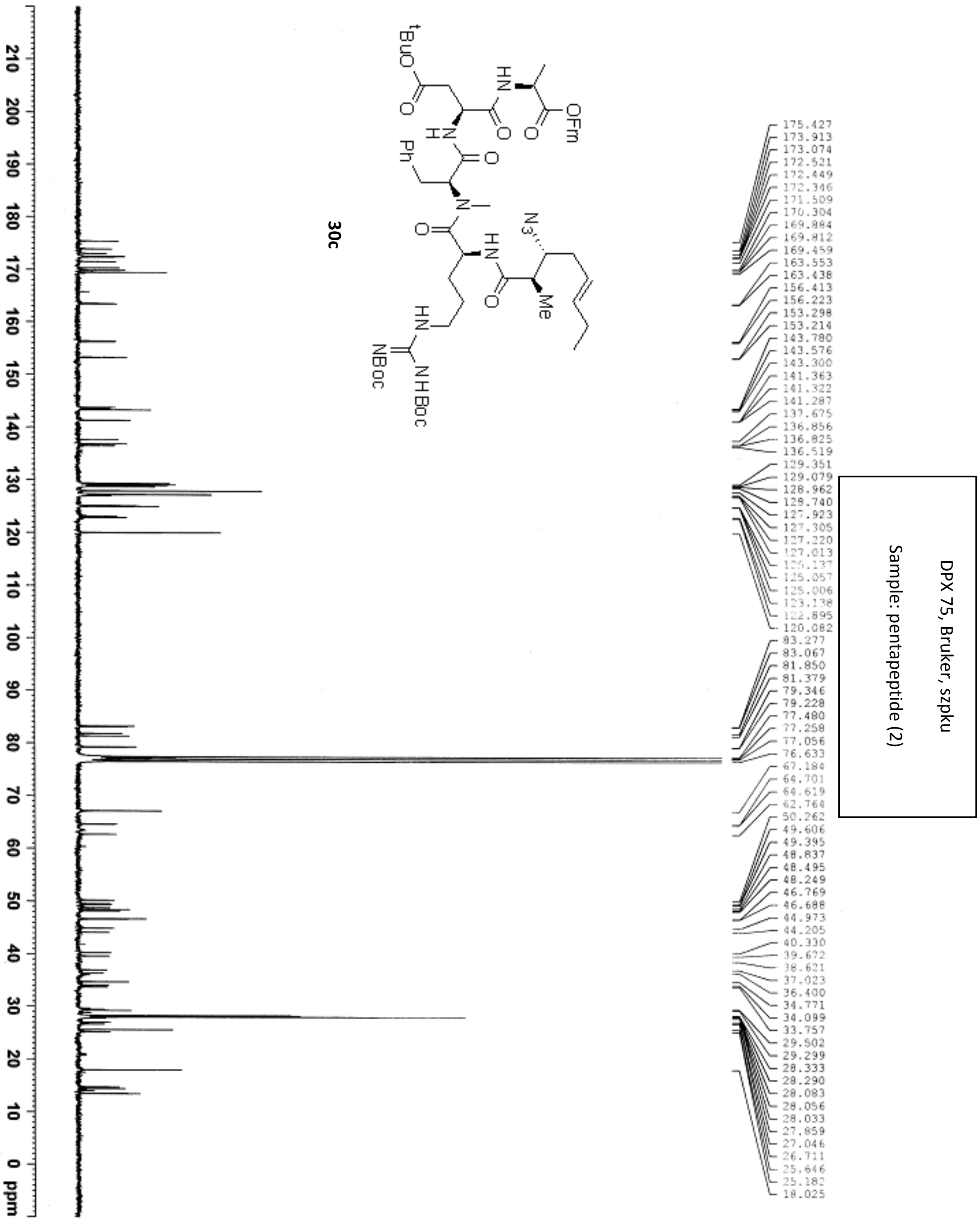
30c
DPX 75, Bruker, szpku
Sample: pentapeptide (2)

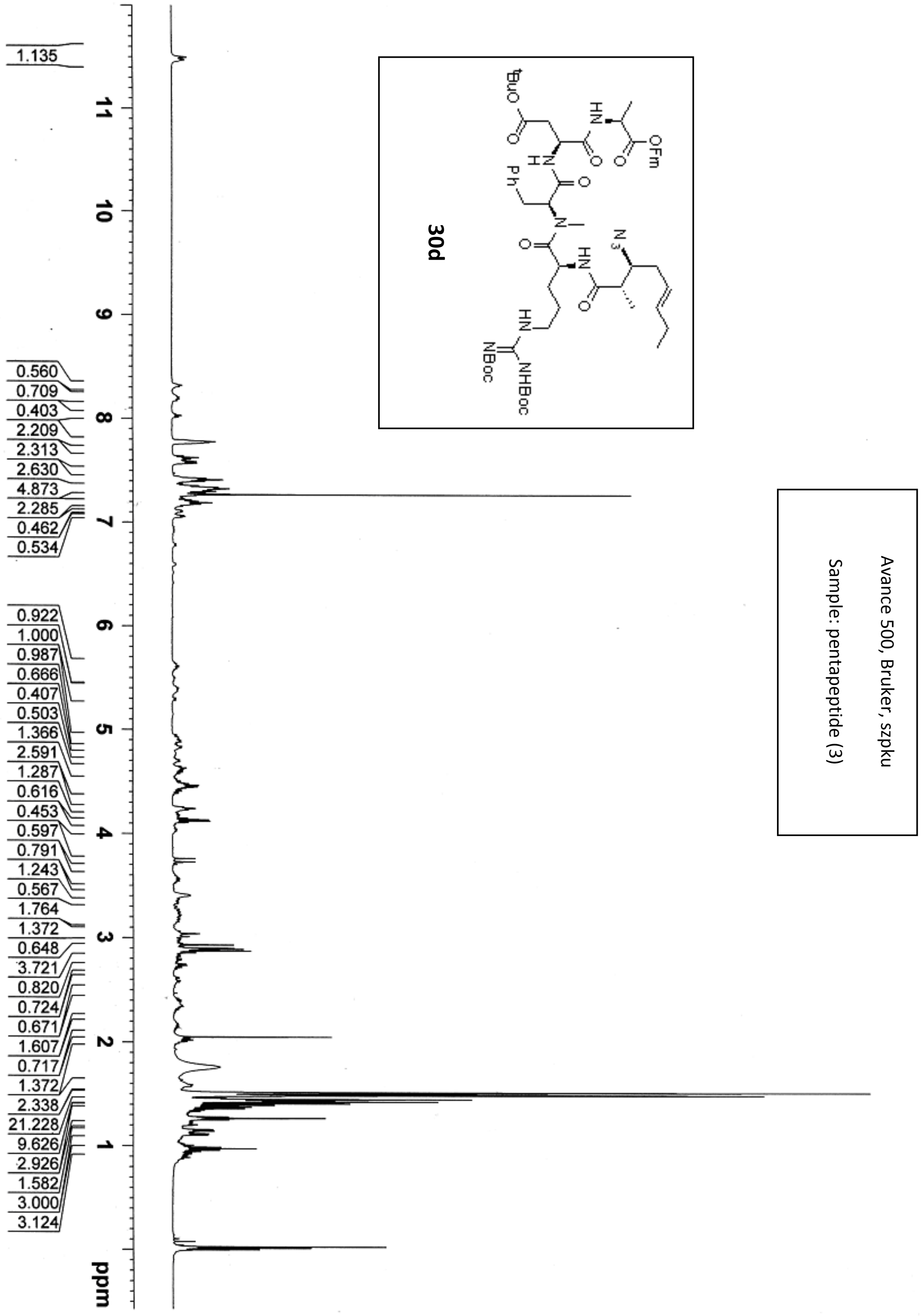
30d
Avance 500, Bruker, szpku
Sample: pentapeptide (3)
ppm

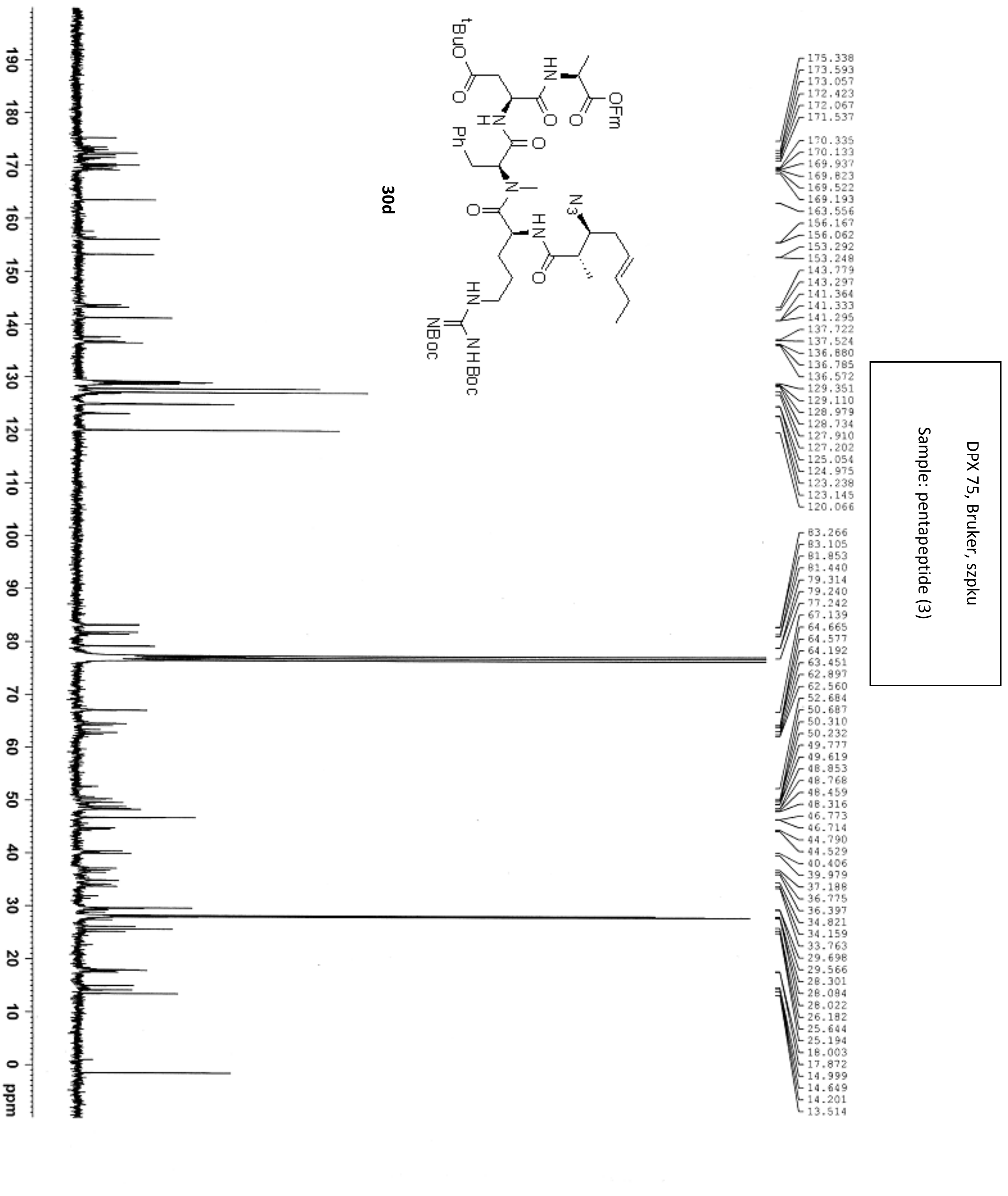
30d
DPX 75, Bruker, szpku
Sample: pentapeptide (3)

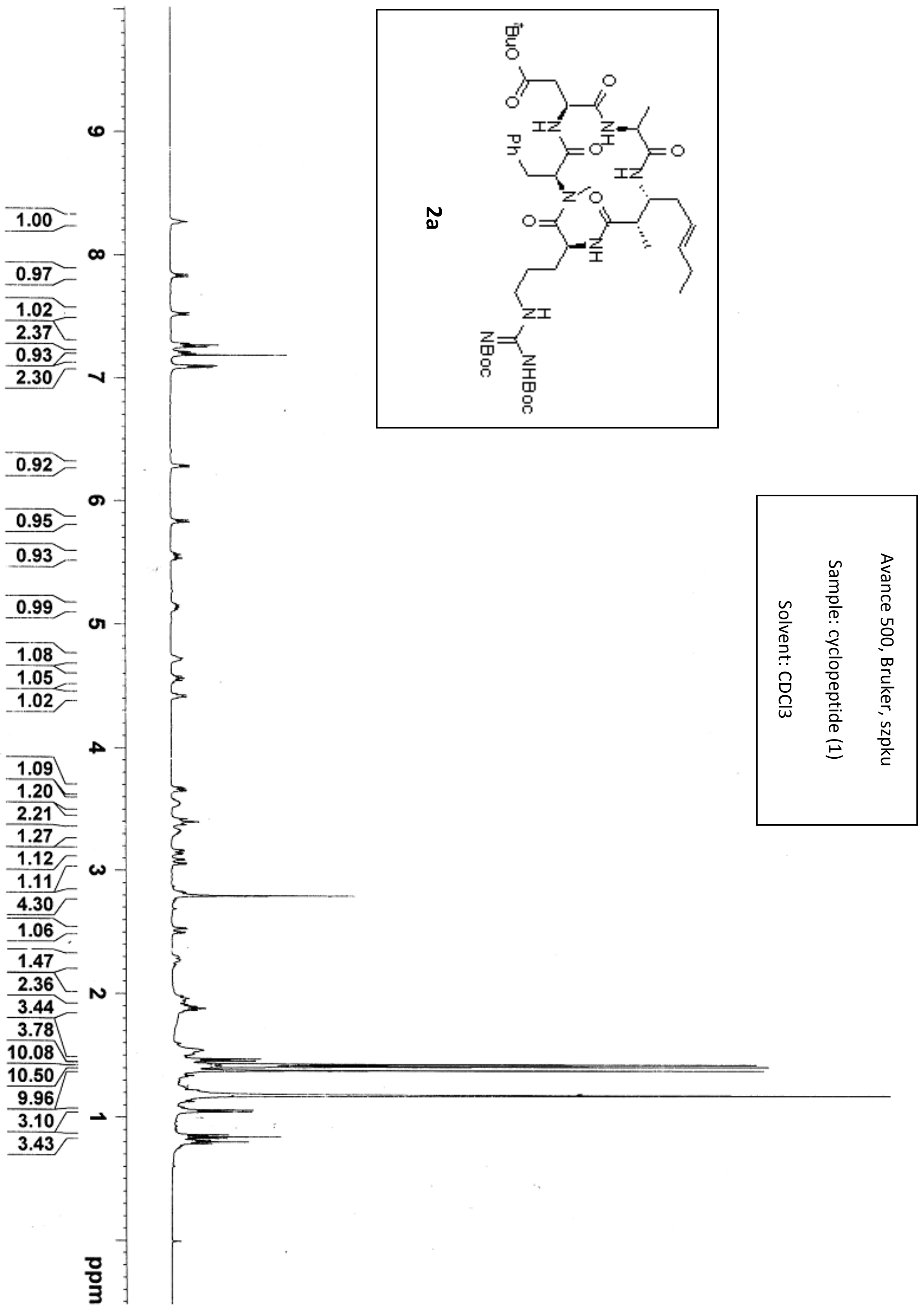
2a
Avance 500, Bruker, szpku
Sample: cyclopeptide (1)
Solvent: CDCl3
ppm

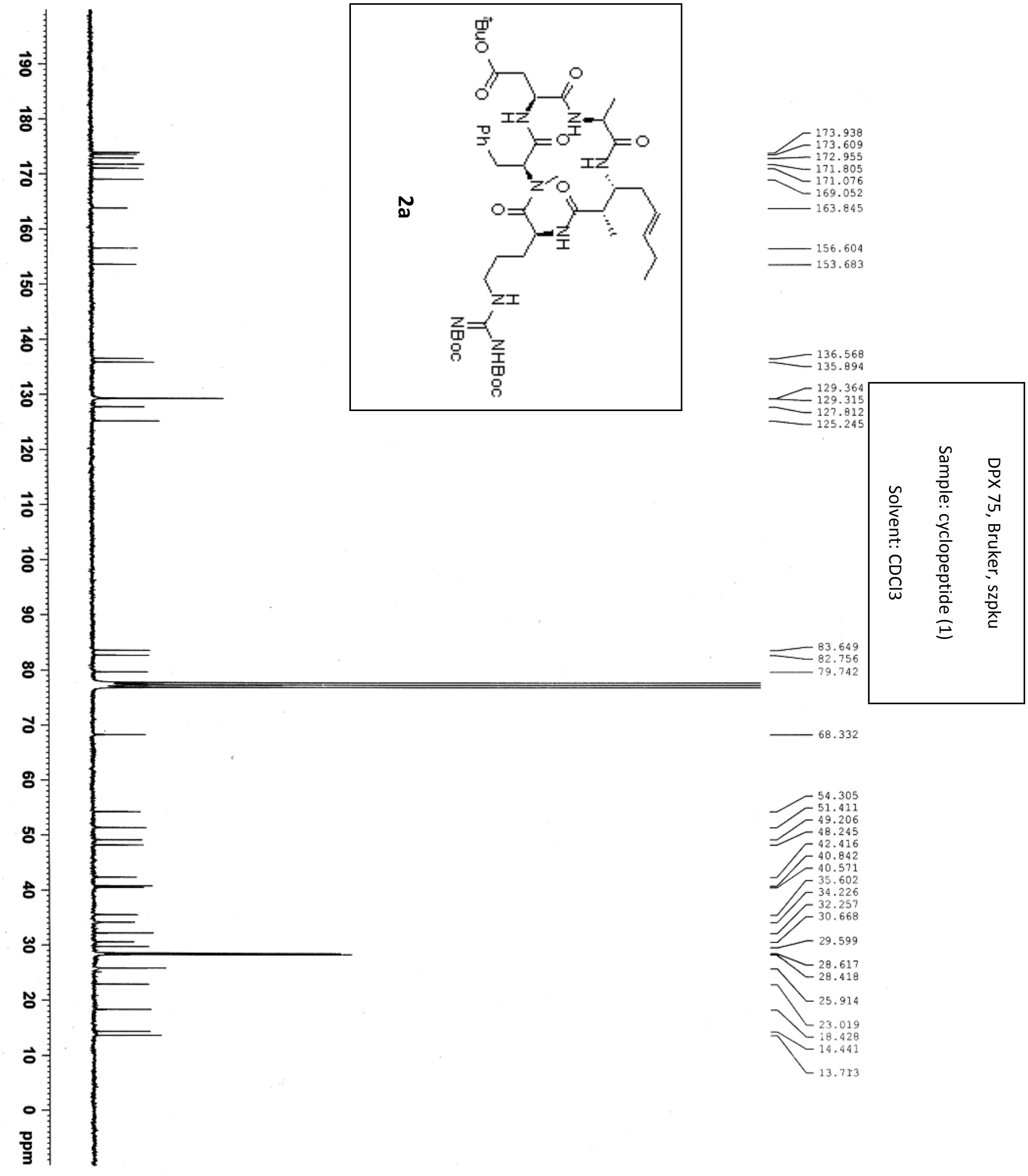
2a
DPX 75, Bruker, szpku
Sample: cyclopeptide (1)
Solvent: CDCl3
173.938
173.609
172.955
171.805
171.076
169.052
163.845
156.604
153.683
136.568
135.894
129.364
129.315
127.812
125.245
83.649
82.756
79.742
68.332
54.305
51.411
49.206
48.245
42.416
40.842
40.571
35.602
34.226
32.257
30.668
29.599
28.617
28.418
25.914
23.019
18.428
14.441
13.713
ppm

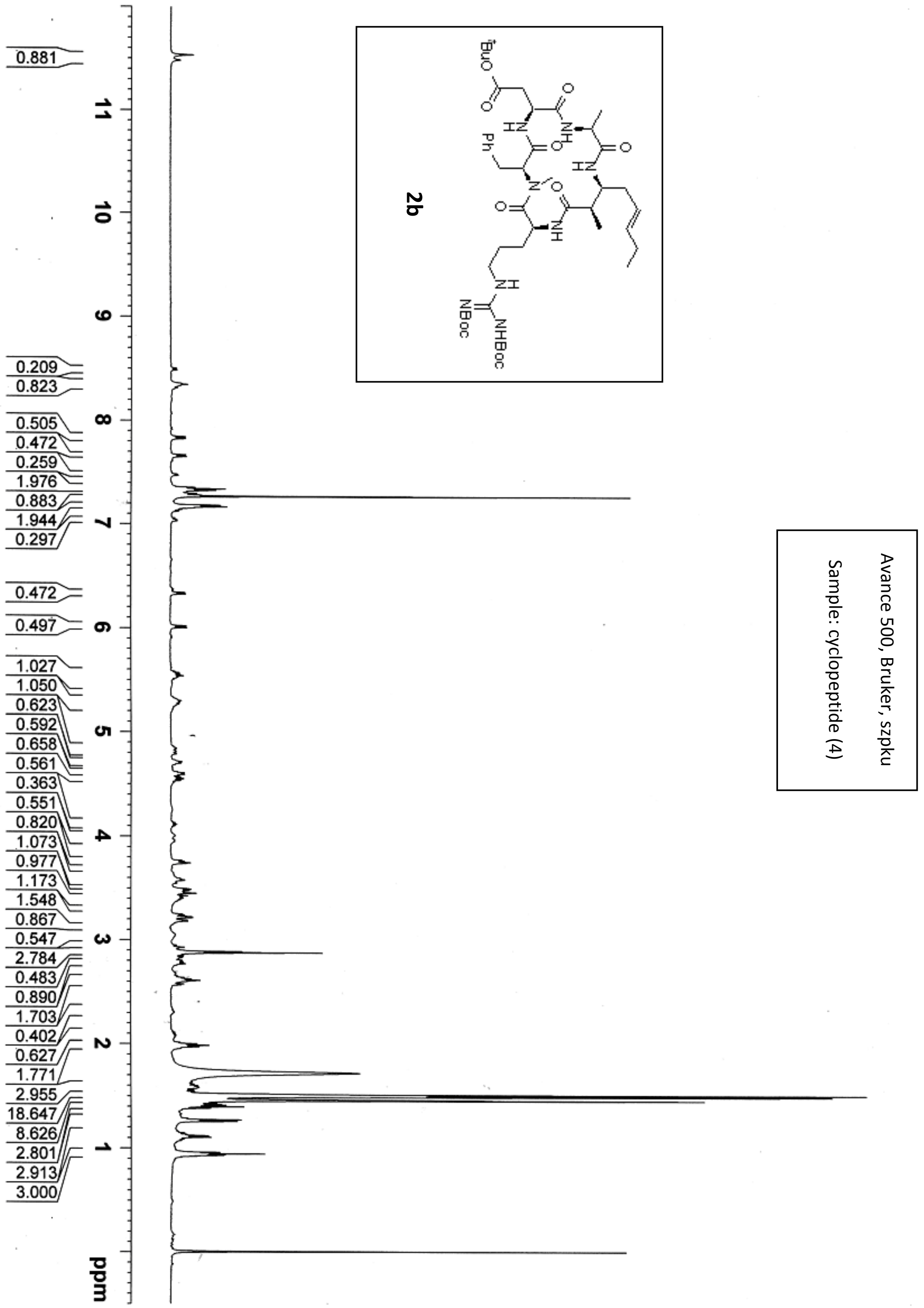
2b
Avance 500, Bruker, szpku
Sample: cyclopeptide (4)
ppm

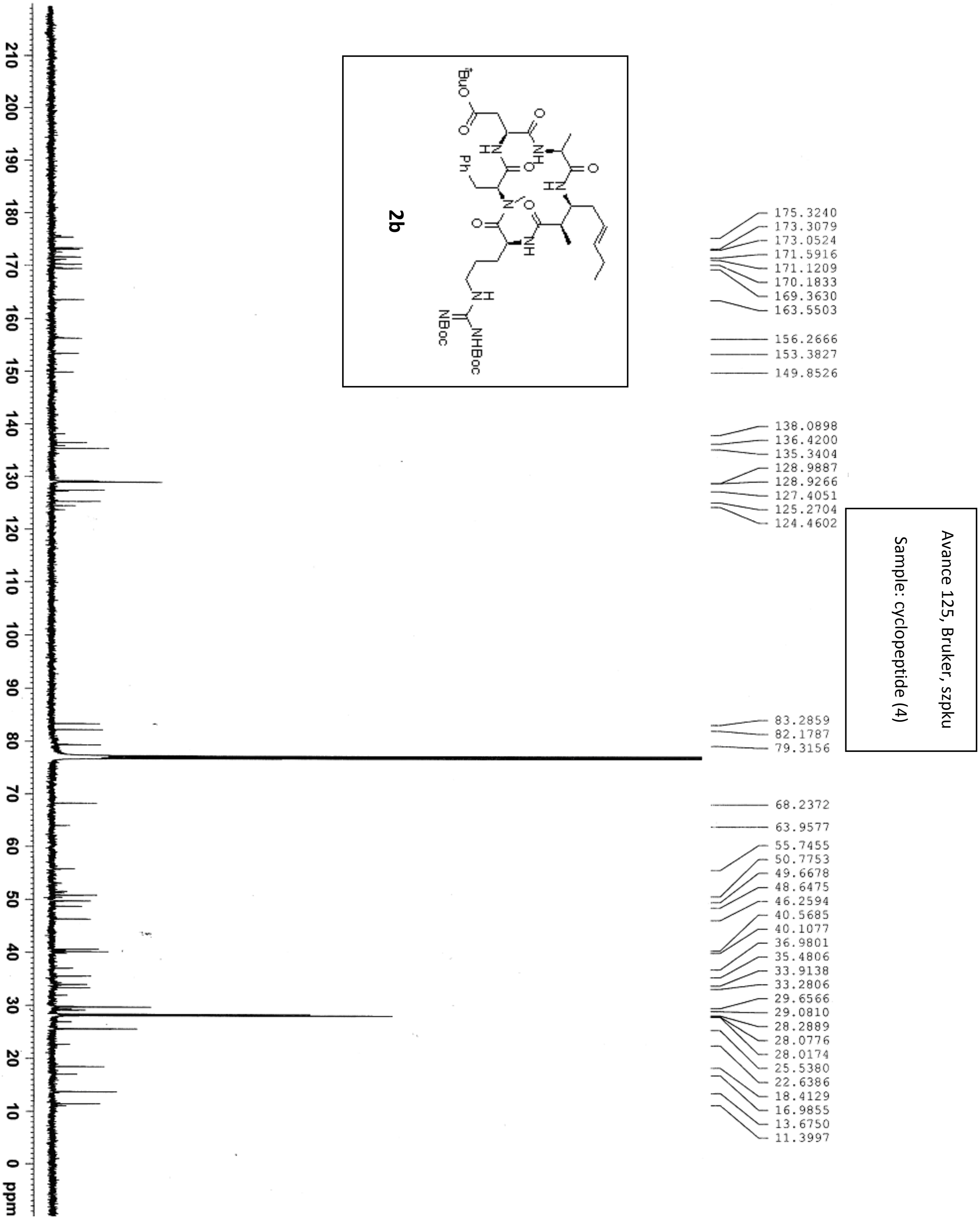
2b
175.3240
173.3079
173.0524
171.5916
171.1209
170.1833
169.3630
163.5503
156.2666
153.3827
149.8526
138.0898
136.4200
135.3404
128.9887
128.9266
127.4051
125.2704
124.4602
83.2859
82.1787
79.3156
68.2372
63.9577
55.7455
50.7753
49.6678
48.6475
46.2594
40.5685
40.1077
36.9801
35.4806
33.9138
33.2806
29.6566
29.0810
28.2889
28.0776
28.0174
25.5380
22.6386
18.4129
16.9855
13.6750
11.3997
Avance 125, Bruker, szpku
Sample: cyclopeptide (4)
ppm

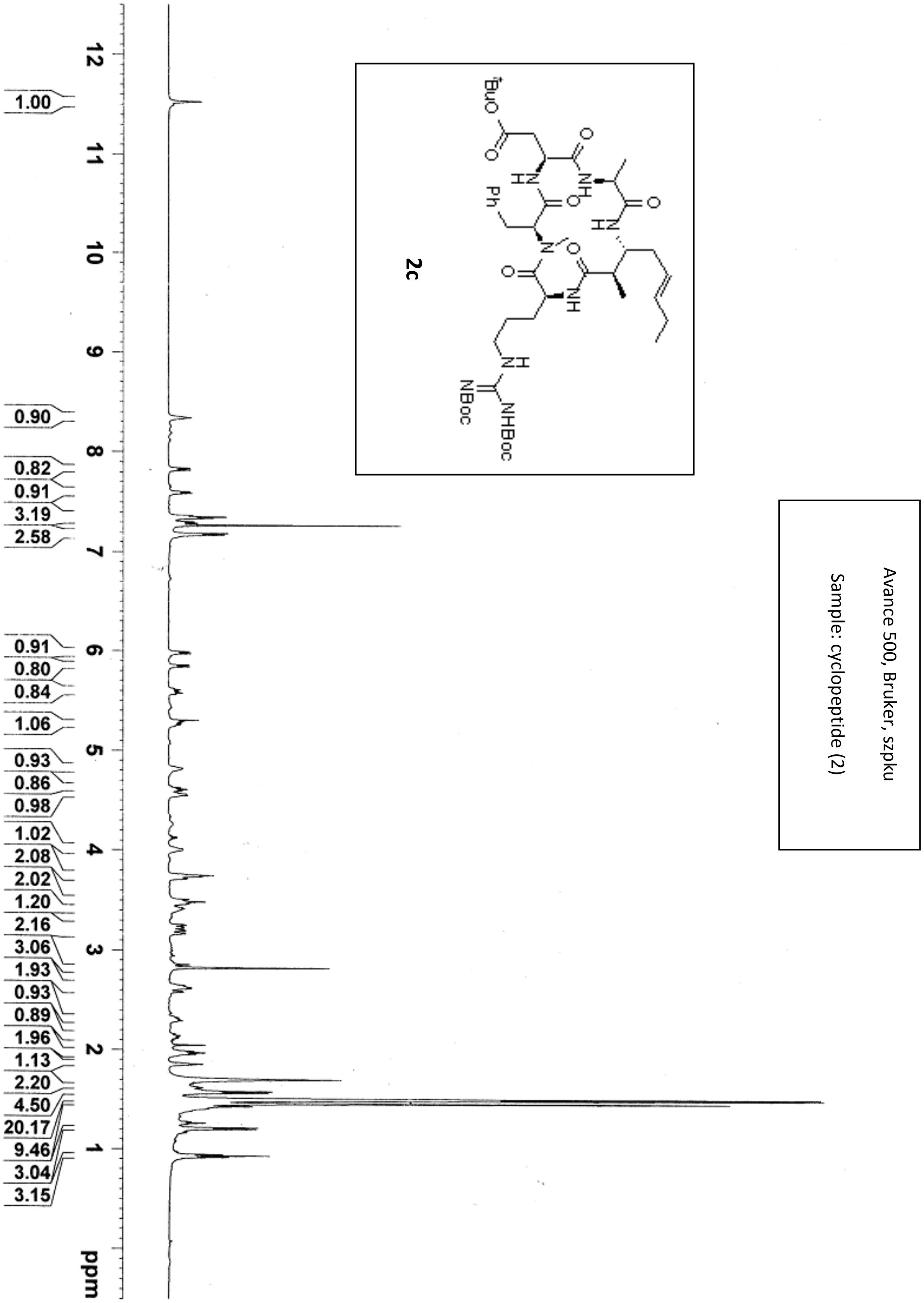
2c
Avance 500, Bruker, szpku
Sample: cyclopeptide (2)
ppm
1.00
0.90
0.82
0.91
3.19
2.58
0.91
0.80
0.84
1.06
0.93
0.86
0.98
1.02
2.08
2.02
1.20
2.16
3.06
1.93
0.93
0.89
1.96
1.13
2.20
4.50
20.17
9.46
3.04
3.15

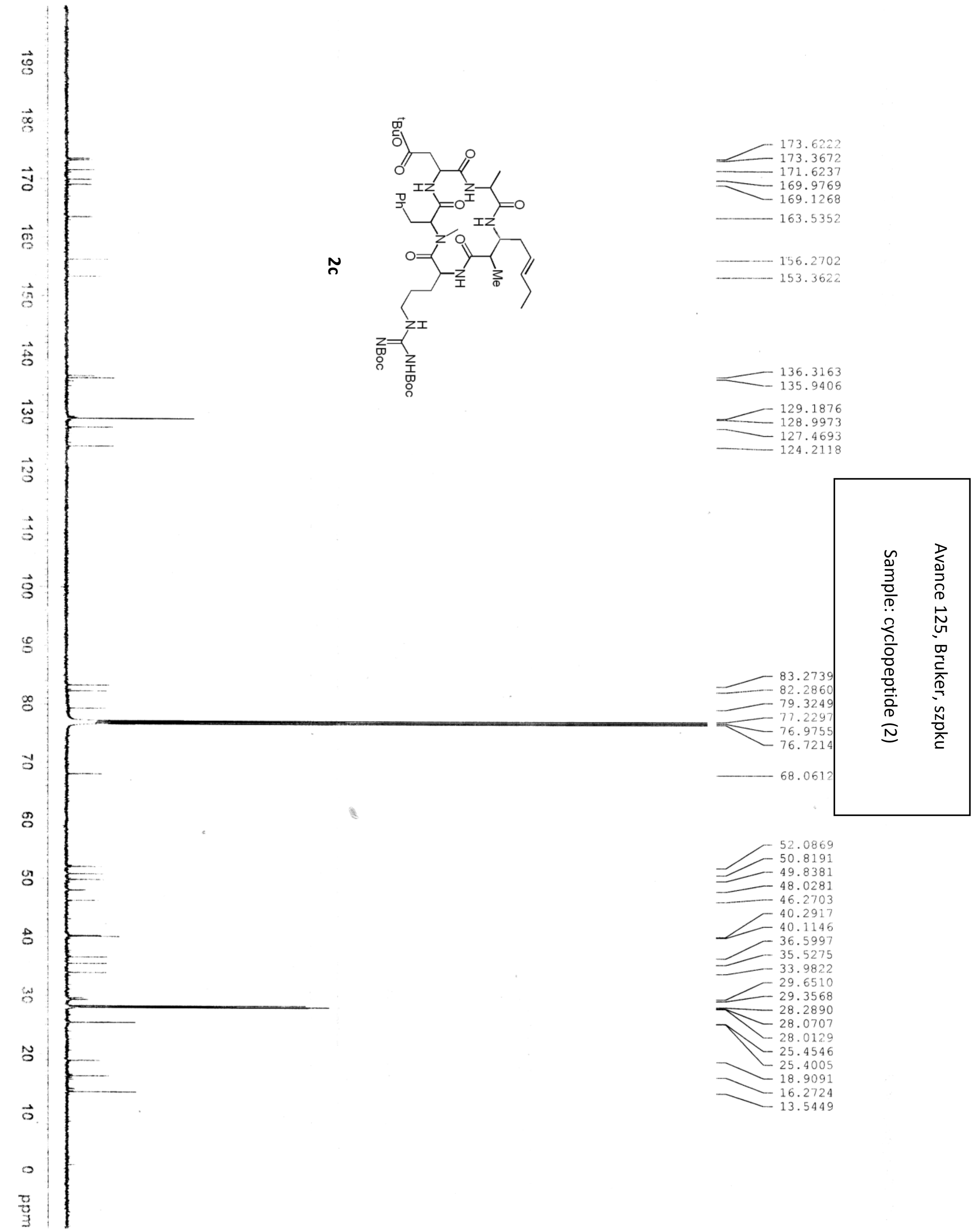
2c
Avance 125, Bruker, szpku
Sample: cyclopeptide (2)
173.6222
173.3672
171.6237
169.9769
169.1268
163.5352
156.2702
153.3622
136.3163
135.9406
129.1876
128.9973
127.4693
124.2118
83.2739
82.2860
79.3249
77.2297
76.9755
76.7214
68.0612
52.0869
50.8191
49.8381
48.0281
46.2703
40.2917
40.1146
36.5997
35.5275
33.9822
29.6510
29.3568
28.2890
28.0707
28.0129
25.4546
25.4005
18.9091
16.2724
13.5449
ppm

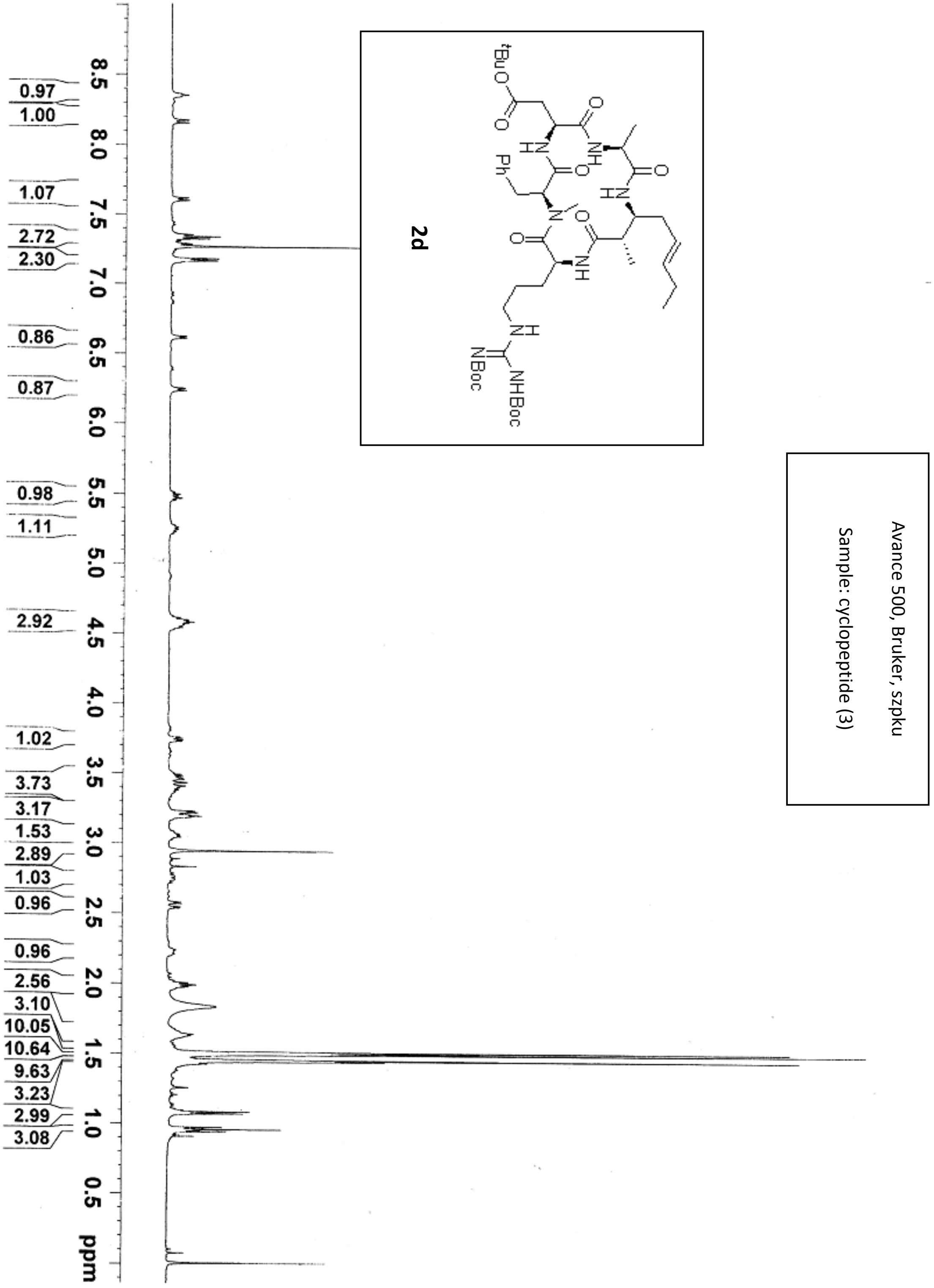

2d

Avance 500, Bruker, szpku
Sample: cyclopeptide (3)

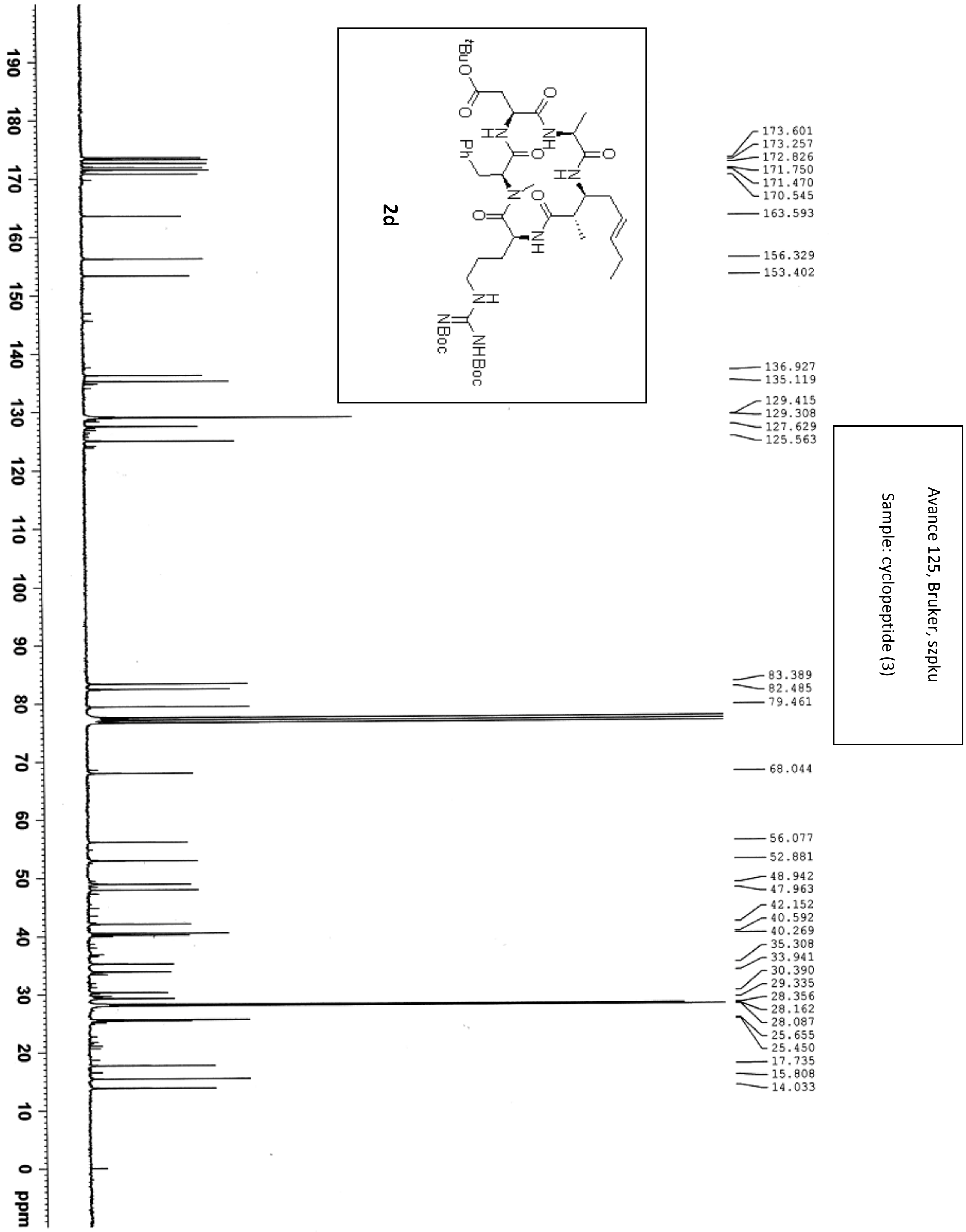
2d
173.601
173.257
172.826
171.750
171.470
170.545
163.593
156.329
153.402
136.927
135.119
129.415
129.308
127.629
125.563
83.389
82.485
79.461
68.044
56.077
52.881
48.942
47.963
42.152
40.592
40.269
35.308
33.941
30.390
29.335
28.356
28.162
28.087
25.655
25.450
17.735
15.808
14.033
Avance 125, Bruker, szpku
Sample: cyclopeptide (3)
ppm

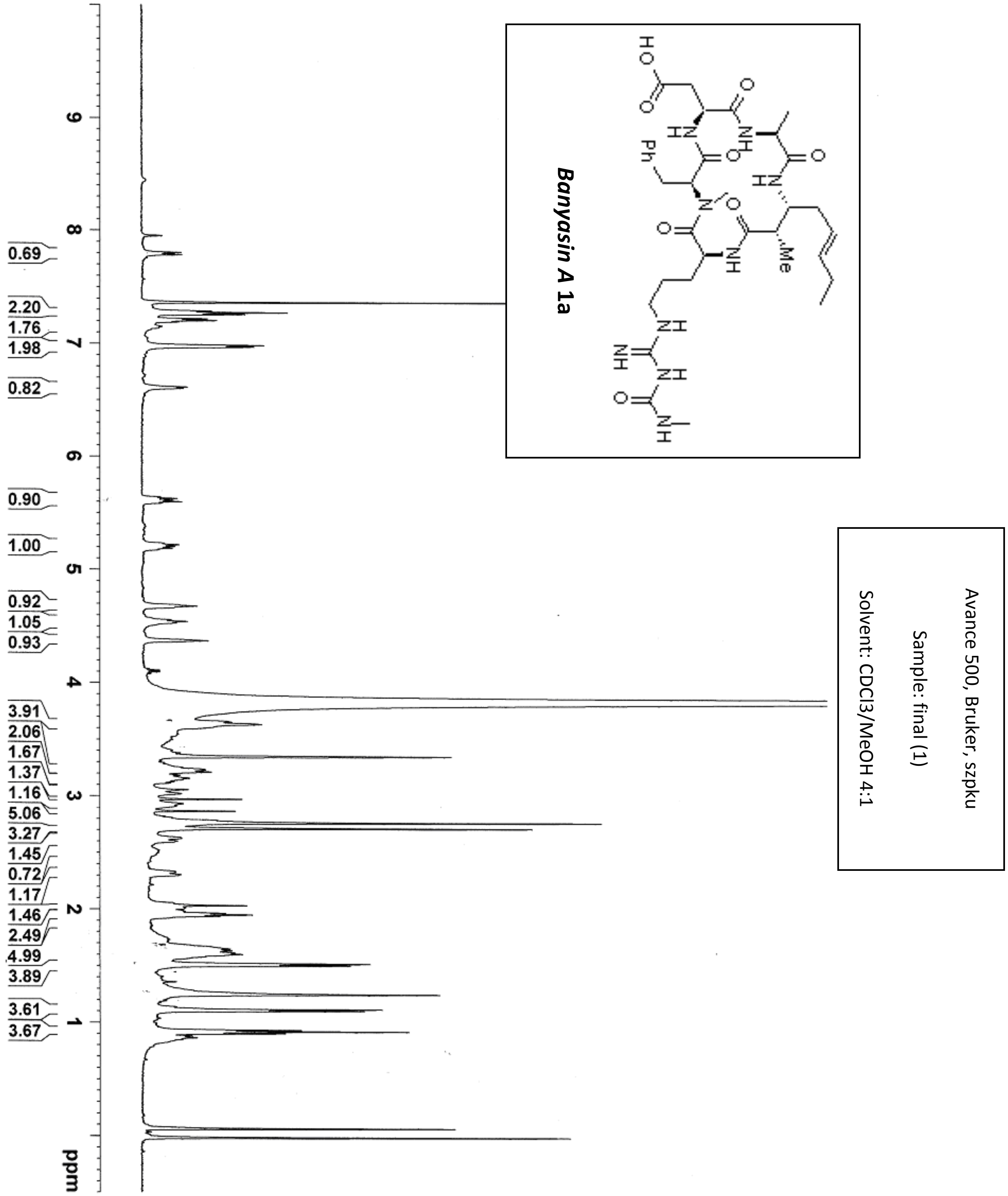
Banyasin A 1a
Avance 500, Bruker, szpku
Sample: final (1)
Solvent: CDCl3/MeOH 4:1
ppm

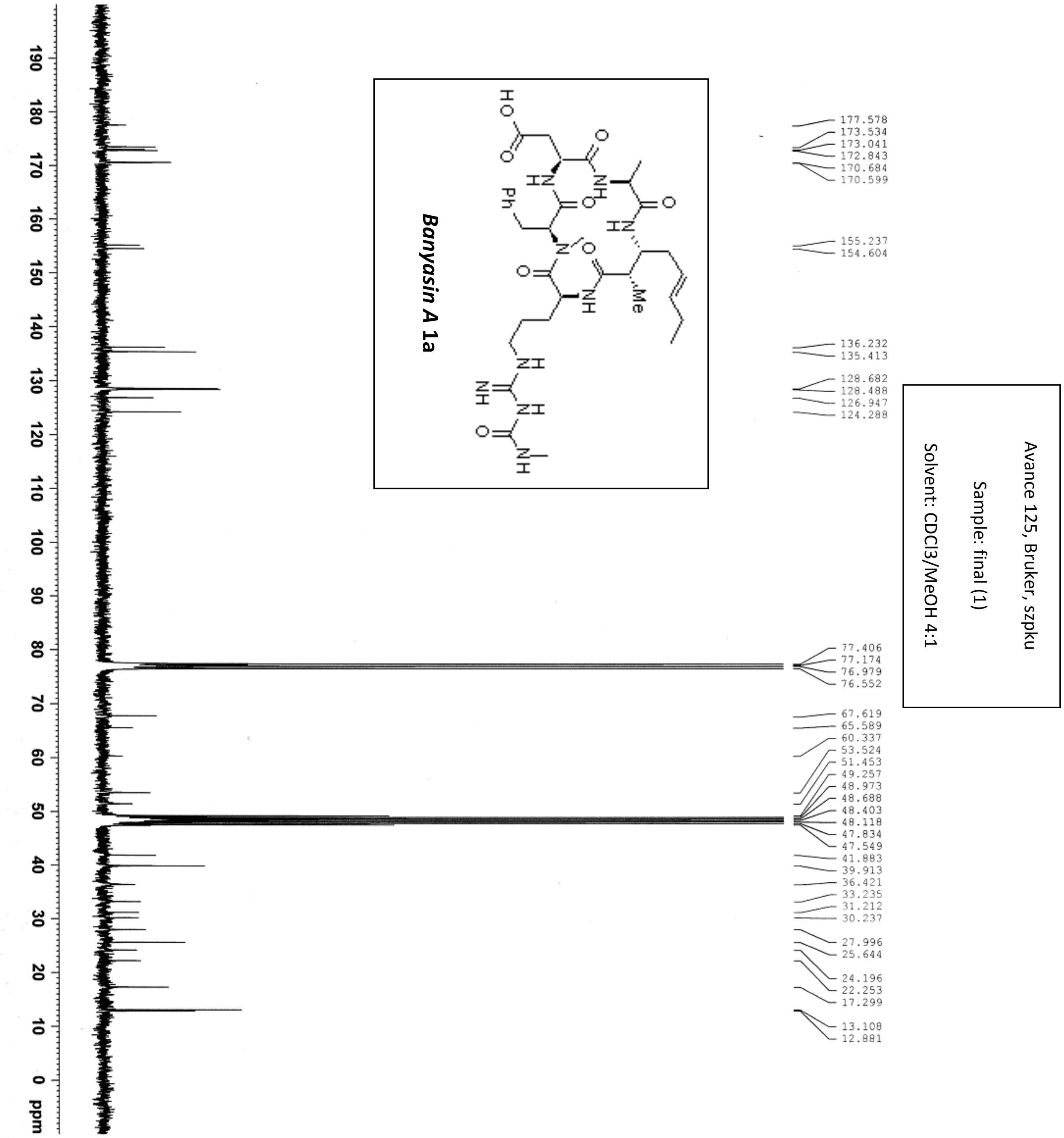

Banyasin A 1a
Avance 125, Bruker, szpku
Sample: final (1)
Solvent: CDCl3/MeOH 4:1
177.578
173.534
173.041
172.843
170.684
170.599
155.237
154.604
136.232
135.413
128.682
128.488
126.947
124.288
77.406
77.174
76.979
76.552
67.619
65.589
60.337
53.524
51.453
49.257
48.973
48.688
48.403
48.118
47.834
47.549
41.883
39.913
36.421
33.235
31.212
30.237
27.996
25.644
24.196
22.253
17.299
13.108
12.881
ppm

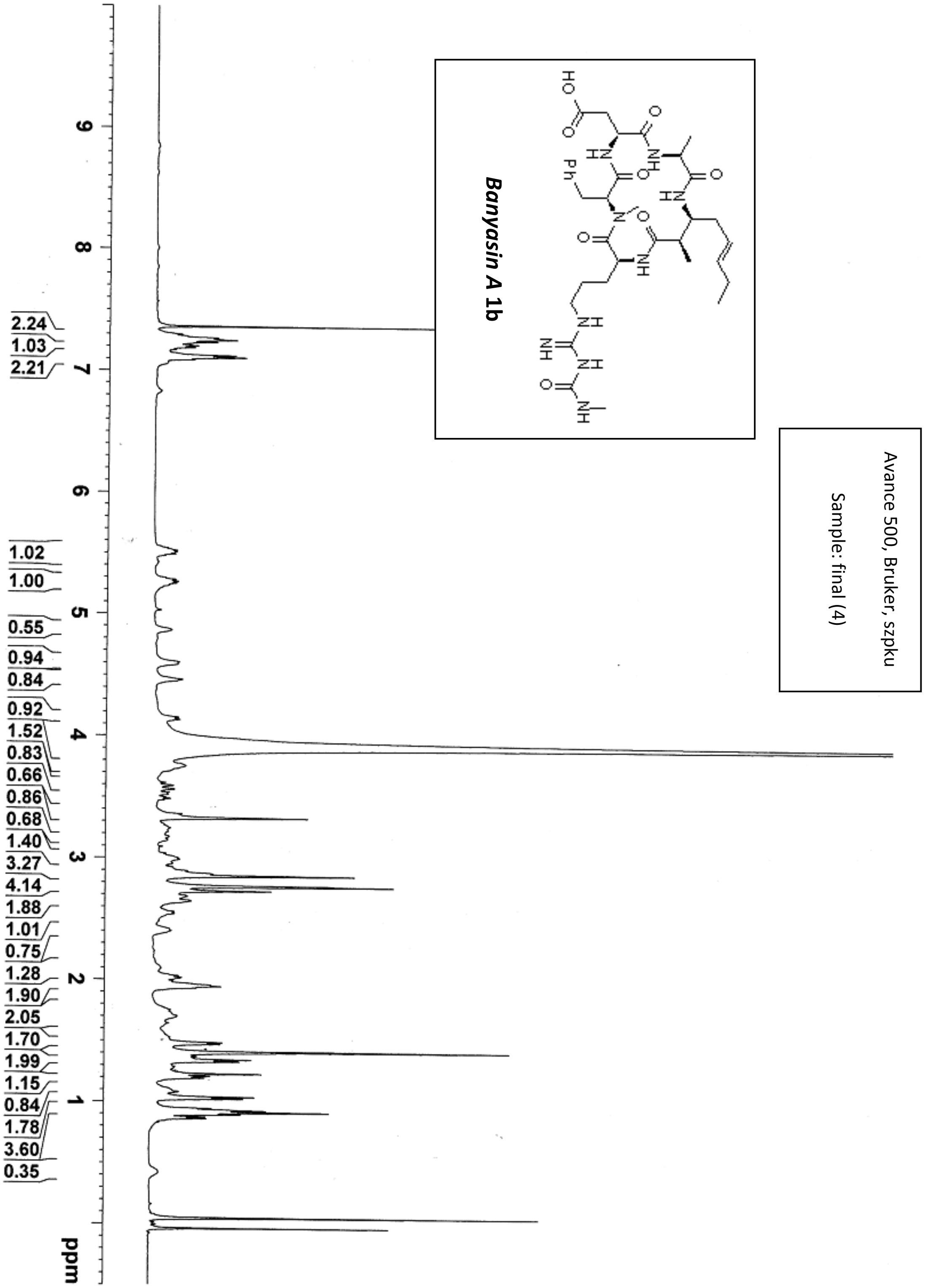
Banyasin A 1b
Avance 500, Bruker, szpku
Sample: final (4)
ppm
2.24
1.03
2.21
1.02
1.00
0.55
0.94
0.84
0.92
1.52
0.83
0.66
0.86
0.68
1.40
3.27
4.14
1.88
1.01
0.75
1.28
1.90
2.05
1.70
1.99
1.15
0.84
1.78
3.60
0.35

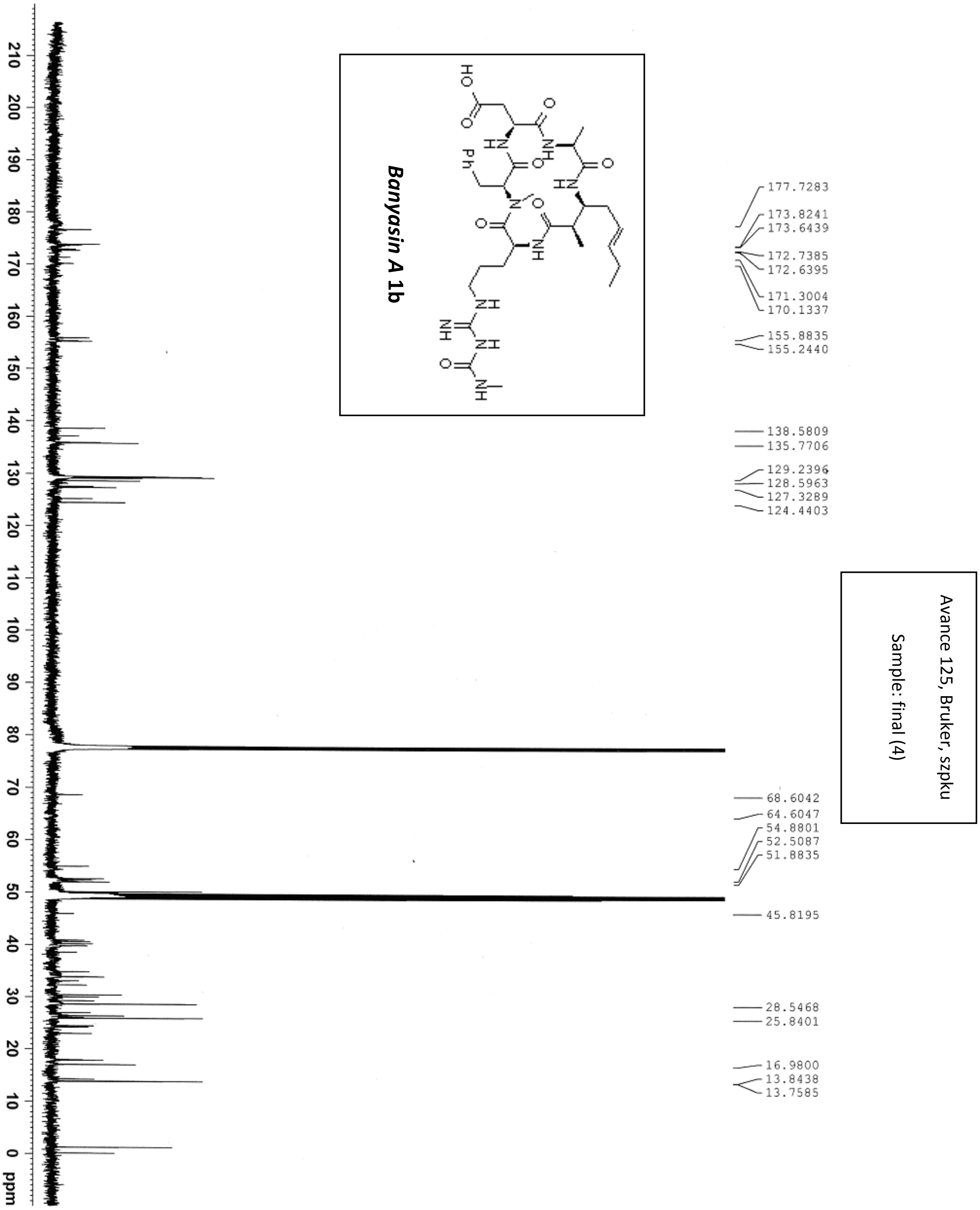
Banyasin A 1b
Avance 125, Bruker, szpku
Sample: final (4)
177.7283
173.8241
173.6439
172.7385
172.6395
171.3004
170.1337
155.8835
155.2440
138.5809
135.7706
129.2396
128.5963
127.3289
124.4403
68.6042
64.6047
54.8801
52.5087
51.8835
45.8195
28.5468
25.8401
16.9800
13.8438
13.7585
ppm

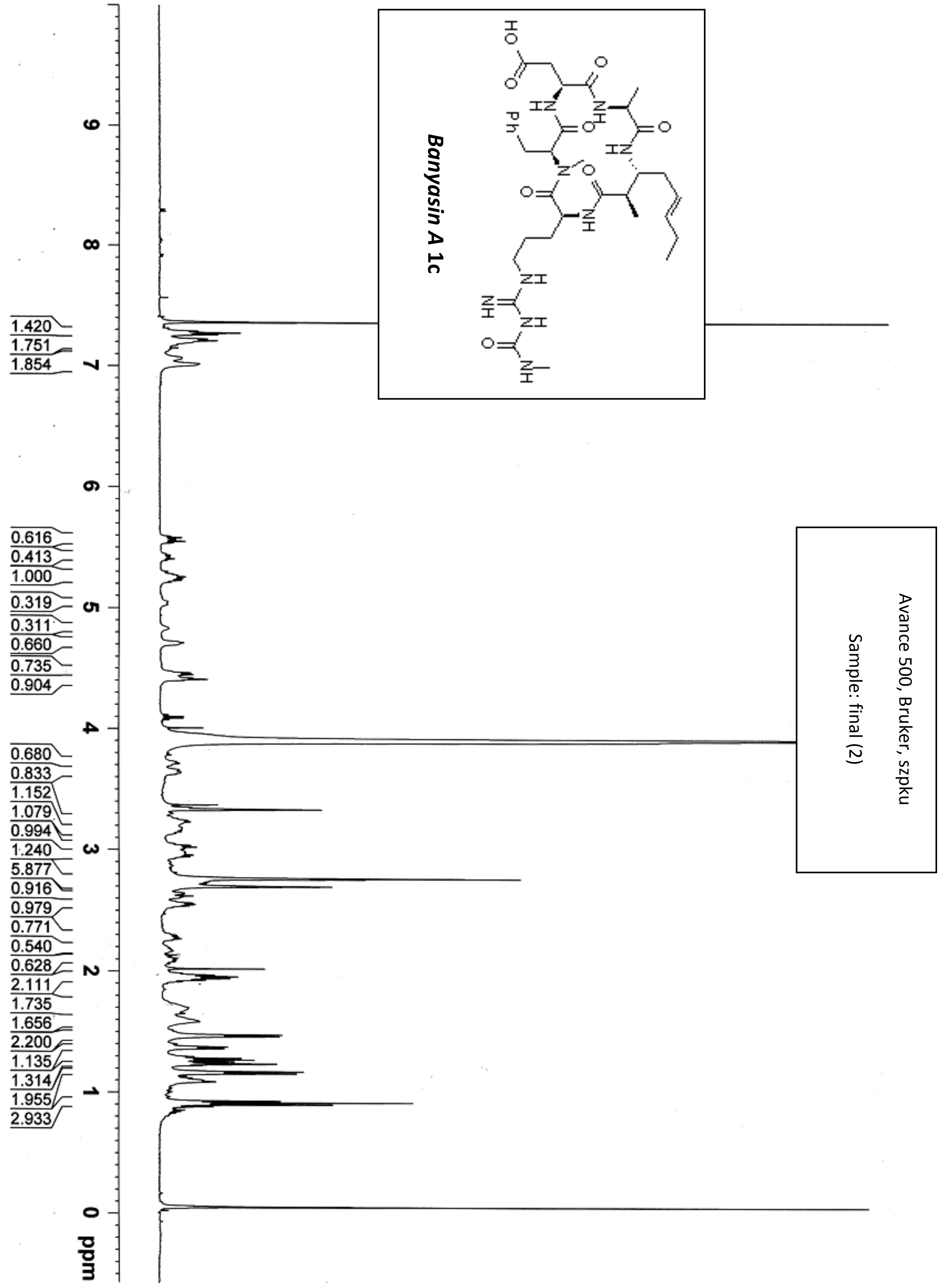
Banyasin A 1c
Avance 500, Bruker, szpku
Sample: final (2)

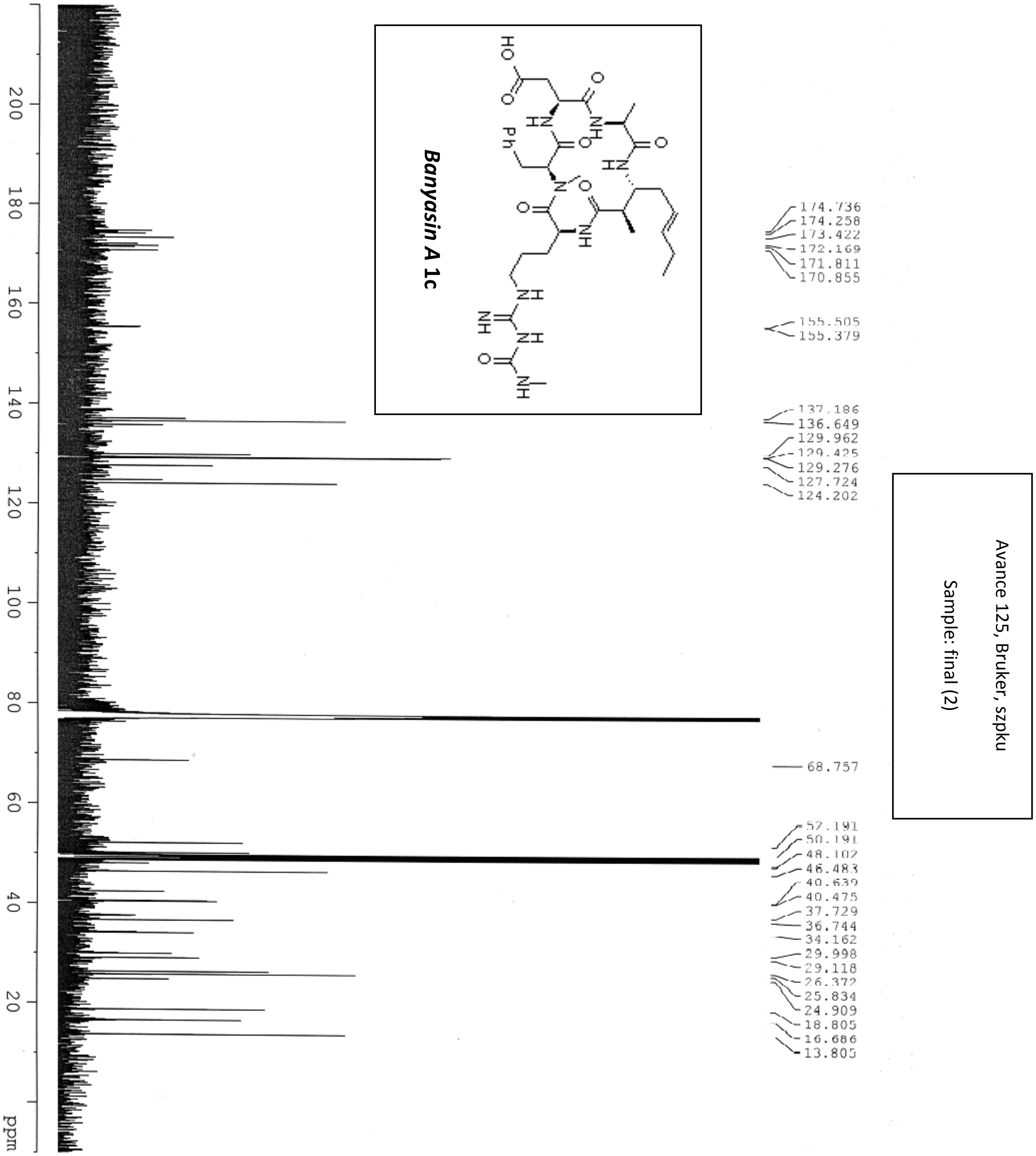
Banyasin A 1c
174.736
174.258
173.422
172.169
171.811
170.855
155.505
155.379
137.186
136.649
129.962
129.425
129.276
127.724
124.202
68.757
57.191
50.191
48.102
46.483
40.639
40.475
37.729
36.744
34.162
29.998
29.118
26.372
25.834
24.909
18.805
16.686
13.805
Avance 125, Bruker, szpku
Sample: final (2)
ppm

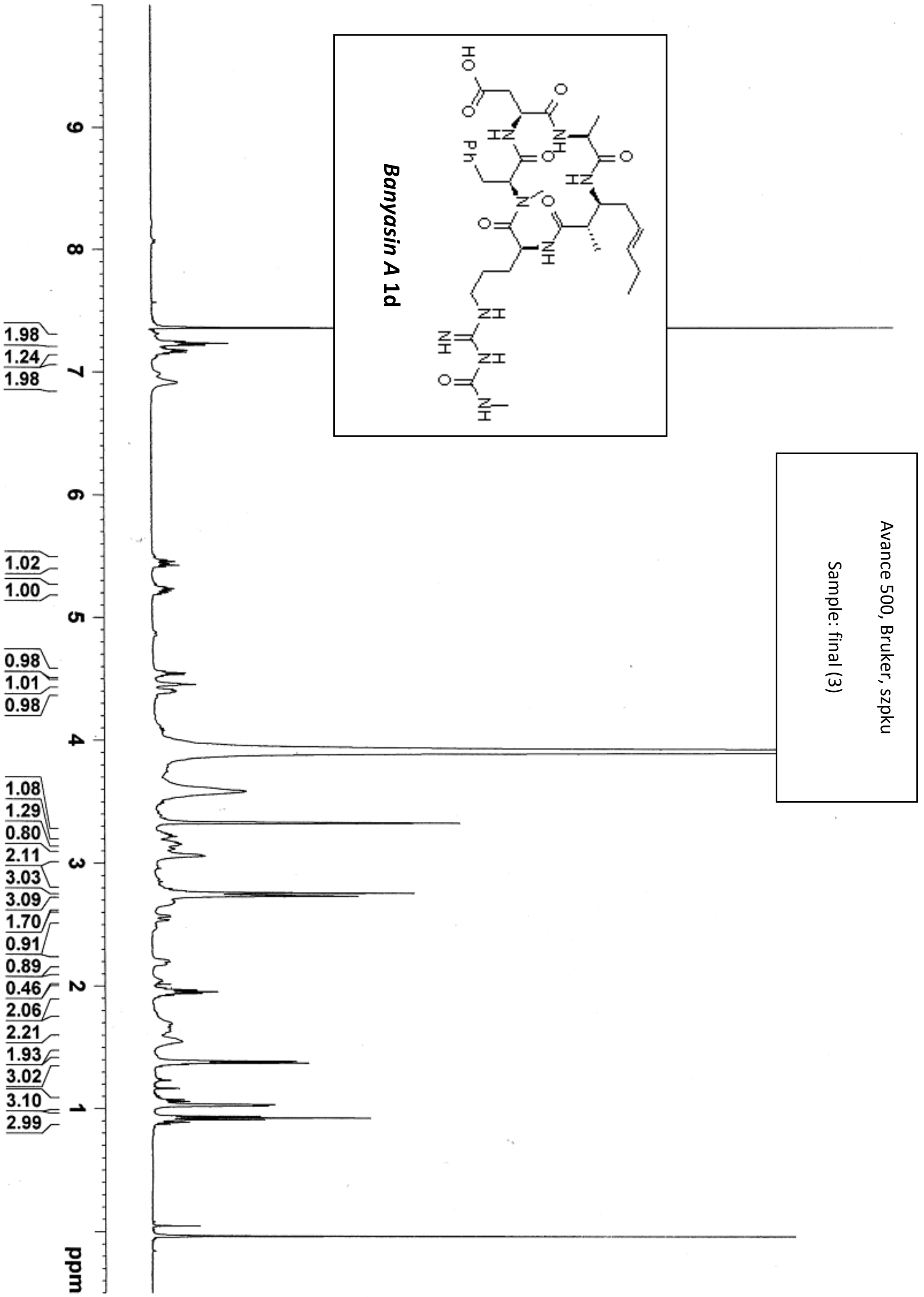
Banyasin A 1d
Avance 500, Bruker, szpku
Sample: final (3)

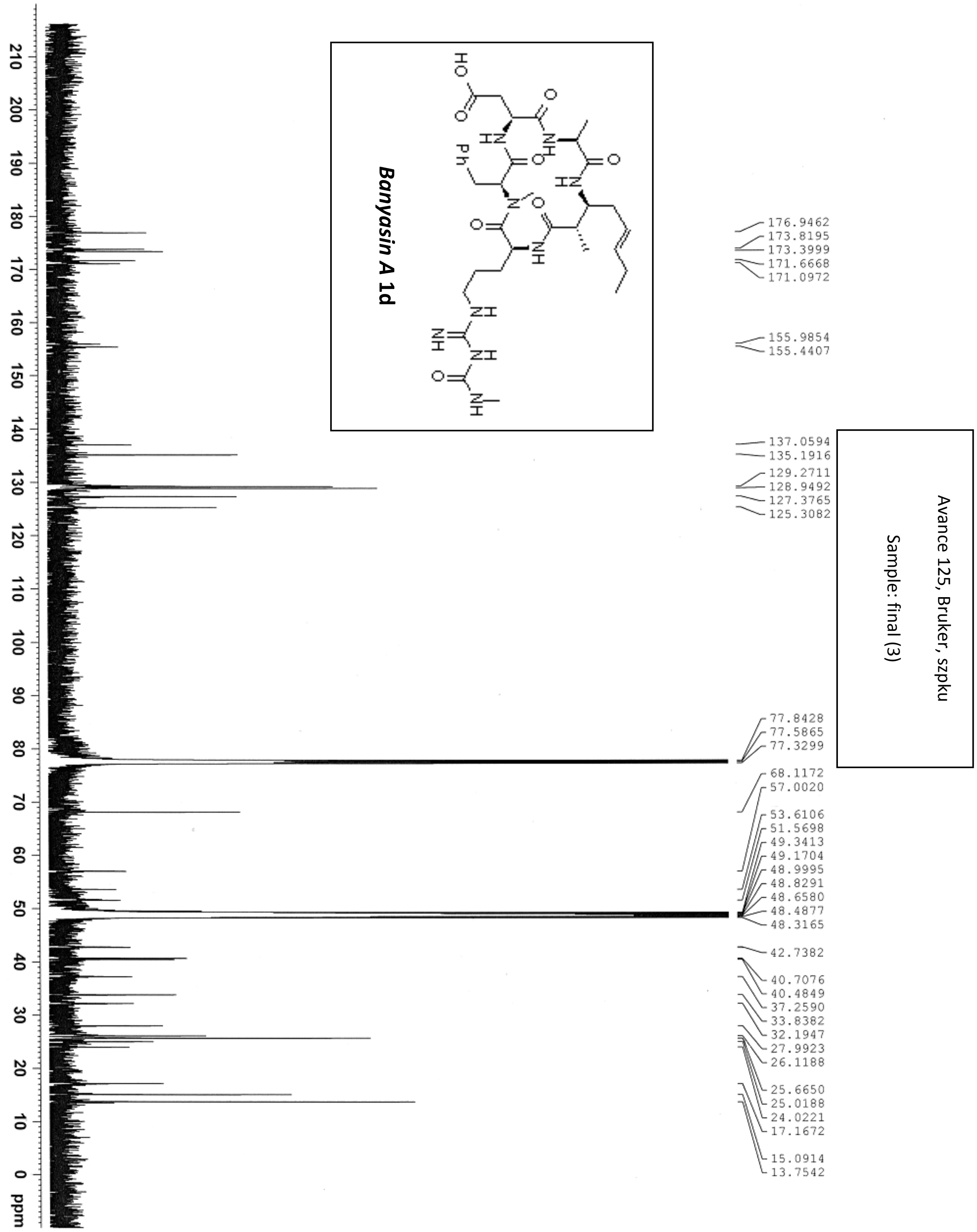
Banyasin A 1d
Avance 125, Bruker, szpku
Sample: final (3)
176.9462
173.8195
173.3999
171.6668
171.0972
155.9854
155.4407
137.0594
135.1916
129.2711
128.9492
127.3765
125.3082
77.8428
77.5865
77.3299
68.1172
57.0020
53.6106
51.5698
49.3413
49.1704
48.9995
48.8291
48.6580
48.4877
48.3165
42.7382
40.7076
40.4849
37.2590
33.8382
32.1947
27.9923
26.1188
25.6650
25.0188
24.0221
17.1672
15.0914
13.7542
ppm
